# Pericyclic reaction benchmarks: hierarchical computations targeting CCSDT(Q)/CBS and analysis of DFT performance†

**DOI:** 10.1039/d2cp02234f

**Published:** 2022-07-21

**Authors:** Pascal Vermeeren, Marco Dalla Tiezza, Mark E. Wolf, Mitchell E. Lahm, Wesley D. Allen, Henry F. Schaefer, Trevor A. Hamlin, F. Matthias Bickelhaupt

**Affiliations:** Department of Theoretical Chemistry, Amsterdam Institute of Molecular and Life Sciences (AIMMS), Amsterdam Center for Multiscale Modeling (ACMM), Vrije Universiteit Amsterdam De Boelelaan 1083 1081 HV Amsterdam The Netherlands t.a.hamlin@vu.nl f.m.bickelhaupt@vu.nl; Center for Computational Quantum Chemistry, University of Georgia Athens GA 30602 USA ccq@uga.edu; Allen Heritage Foundation Dickson TN 37055 USA; Institute for Molecules and Materials (IMM), Radboud University Heyendaalseweg 135 6525 AJ Nijmegen The Netherlands

## Abstract

Hierarchical, convergent *ab initio* benchmark computations were performed followed by a systematic analysis of DFT performance for five pericyclic reactions comprising Diels-Alder, 1,3-dipolar cycloaddition, electrocyclic rearrangement, sigmatropic rearrangement, and double group transfer prototypes. Focal point analyses (FPA) extrapolating to the *ab initio* limit were executed *via* explicit quantum chemical computations with electron correlation treatments through CCSDT(Q) and correlation-consistent Gaussian basis sets up to aug′-cc-pV5Z. Optimized geometric structures and vibrational frequencies of all stationary points were obtained at the CCSD(T)/cc-pVTZ level of theory. The FPA reaction barriers and energies exhibit convergence to within a few tenths of a kcal mol^−1^. The FPA benchmarks were used to evaluate the performance of 60 density functionals (eight dispersion-corrected), covering the local-density approximation (LDA), generalized gradient approximations (GGAs), meta-GGAs, hybrids, meta-hybrids, double-hybrids, and range-separated hybrids. The meta-hybrid M06-2X functional provided the best overall performance [mean absolute error (MAE) of 1.1 kcal mol^−1^] followed closely by the double-hybrids B2K-PLYP, mPW2K-PLYP, and revDSD-PBEP86 [MAE of 1.4–1.5 kcal mol^−1^]. The regularly used GGA functional BP86 gave a higher MAE of 5.8 kcal mol^−1^, but it qualitatively described the trends in reaction barriers and energies. Importantly, we established that accurate yet efficient meta-hybrid or double-hybrid DFT potential energy surfaces can be acquired based on geometries from the computationally efficient and robust BP86/DZP level.

## Abstract 2

A hierarchical *ab initio* benchmark up to CCSDT(Q)/CBS in combination with a DFT performance study reveals meta-hybrid M06-2X functional as the best performing functional for accurately describing pericyclic reactions.

## Graphical abstract

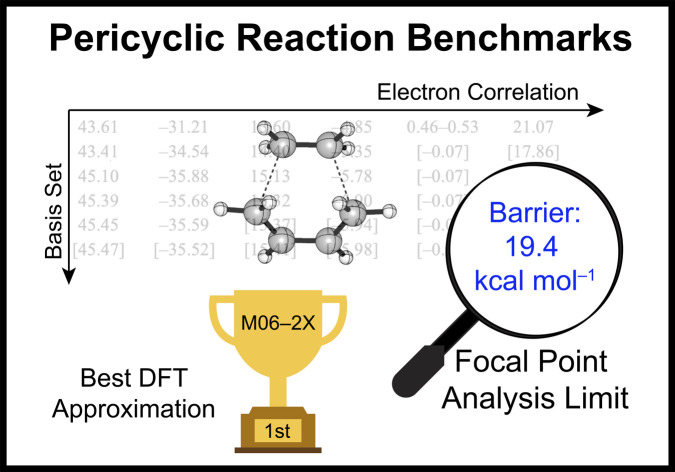

## 1. Introduction

Owing to their robust and versatile nature, pericyclic reactions are highly useful chemical transformations in organic and organometallic chemistry.^1^ A particular advantage of this class of reactions is the ability to increase molecular complexity in a single reaction step, often with high stereoselectivity. For this reason, these reactions have been widely utilized in syntheses of a broad range of target compounds, including complex natural products, as well as molecular species with applications in material sciences and medicinal chemistry.^2^

**Scheme 1:**
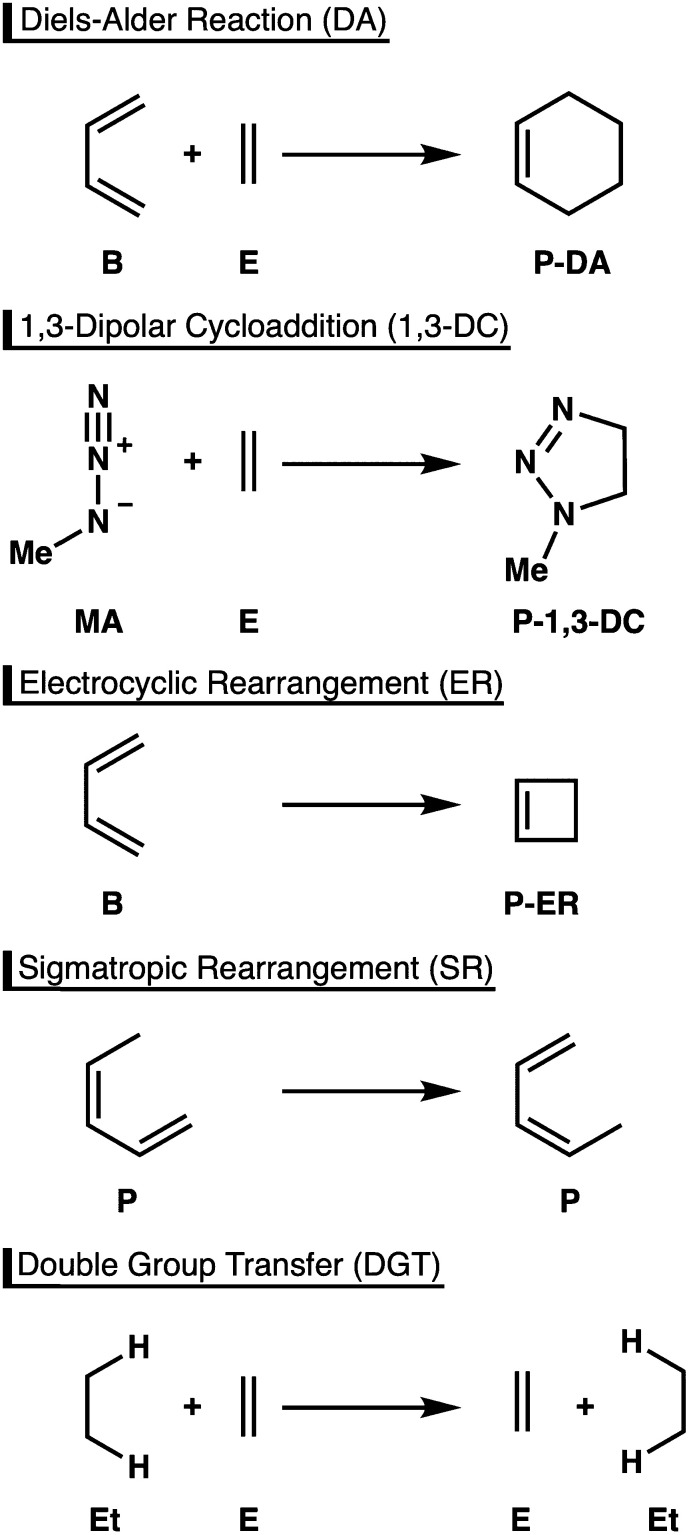
Paradigmatic pericyclic reactions studied in this work.

An important feature of pericyclic reactions, including the herein studied Diels-Alder (DA) reaction, 1,3-dipolar cycloaddition (1,3-DC), electrocyclic rearrangement (ER), sigmatropic rearrangement (SR), and double group transfer (DGT) (Scheme 1), is that they proceed *via* a fully conjugated cyclic transition state.^1^ Density functional theory computations have been paramount for elucidating the nature of such concerted chemical transformations.^3,4^ However, to obtain accurate results an appropriate density functional must be chosen among myriad possibilities, a decision that is hardly transparent and one that requires highly accurate reference data for calibration.

In recent decades, various studies have been dedicated to finding the best density functional approximation for either a large set of various pericyclic reactions^5^ or only a subset of them, such as Diels-Alder^6^ or 1,3-dipolar cycloaddition reactions.^7^ Most of these studies benchmarked density functionals by means of a ‘single-shot’ energy calculation with a composite method of some complete basis set (CBS) type; however, the true quality of the reference data is unknown. In fact, Karton and Goerigk recently discovered that the often-used CBS-QB3 method fails to give accurate reaction barriers for four classes of pericyclic reactions (26 reactions in total),^5*c*^ exhibiting maximum and RMS errors of −4.7 and 2.5 kcal mol^−1^, respectively, relative to the W2-F12 protocol. This demonstrates the necessity of having indubitable reference data for the potential energy surfaces (PESs) of pericyclic reactions. Another study by Karton in 2019^8^ computed the barrier heights of a diverse set of 28 reactions that proceed *via* ring-forming transition states using the W3lite-F12 protocol. As detailed below, part of their investigation overlaps with the DA, ER, and SR reactions studied here; however, our results are more extensive and rigorous, pushing further toward the limits of electronic structure theory.

The first goal of this study is to employ high-level *ab initio* methods to compute definitive structures and energetics for the paradigmatic pericyclic reactions depicted in Scheme 1. We employ the highest-level correlated wave functions [CCSD(T)/cc-pVTZ] used to date to optimize geometric structures for these reactions. A majority of previous studies on pericyclic reactions have utilized DFT geometries.^5*a*,*c*,6,7*a*,*b*^ Nevertheless, a few *ab initio* structures can be found, computed with, for example, CCSD(T)/6-31G(d,p) for DGT,^9*a*^ CCSD/6-311++G(df,p) for ER,^9*b*^ and MP2/6-31G(d,p) for 1,3-DC,^9*c*^ but these are of significantly lower quality than the geometrical structures presented here. To pinpoint reaction energetics, hierarchical focal point analyses (FPA)^10^ are executed to converge toward both the one- and *n*-particle limits of *ab initio* quantum chemistry by means of correlation-consistent Gaussian basis sets [aug′-cc-pV*X*Z, *X* = D, T, Q, 5] of systematically increasing flexibility and polarization conjoined with the high-order coupled-cluster electron correlation series [HF, MP2, CCSD, CCSD(T), CCSDT, CCSDT(Q)].

The second goal of this study is to employ the FPA benchmarks to evaluate the performance of 60 diverse density functionals, including eight with dispersion corrections, implemented with Slater-type TZ2P and QZ4P basis sets. The functionals range in quality from the local-density approximation (LDA) to generalized gradient approximations (GGAs), meta-GGAs, hybrids, meta-hybrids, double-hybrids, and range-separated hybrids. An efficient and accurate protocol is established for the study of pericyclic reactions that relies on the computation of BP86/DZP optimum geometries for final TZ2P single-point energy computations with the meta-hybrid functional M06-2X, or the alternatives B2K-PLYP, mPW2K-PLYP, and revDSD-PBEP86.

## 2. Computational methods

### 2.1 Benchmark geometric structures, vibrational frequencies, and relative energies

Fully optimized geometric structures and harmonic vibrational frequencies of the reactants, products, and transition states on the potential energy surfaces of the pericyclic reactions were computed using coupled-cluster electronic wave functions with single, double, and perturbative triple excitations [CCSD(T)]^11^ in conjunction with the correlation-consistent polarized valence basis set cc-pVTZ.^12^ The character of each stationary point was verified by its vibrational frequencies, and the normal mode associated with the single imaginary frequency of the transition states was inspected to ensure that it pertained to the reaction of interest. The CCSD(T)/cc-pVTZ harmonic vibrational frequencies also provide high-quality zero-point vibrational energies (ZPVEs) and thus are provided in Table S1 (ESI†).

To obtain final energetics, focal point analyses^11^ were executed on the CCSD(T)/cc-pVTZ geometric structures using the following hierarchy of quantum chemical methods: HF, MP2,^13^ CCSD,^14^ CCSD(T),^11^ CCSDT,^15^ and CCSDT(Q)^16^ in conjunction with the cc-pVDZ^9^ and aug′-cc-pV*X*Z (*X* = D, T, Q, 5) basis sets to systematically proceed toward the CBS limit. These computations were carried out using the Molpro 2010 package,^17^ except that Kállay's MRCC^18^ program was employed in the CCSDT and CCSDT(Q) cases. The aug′-cc-pV*X*Z basis sets are composed of aug-cc-pV*X*Z^12*b*^ functions on the heavy atoms and standard Dunning sets (cc-pV*X*Z)^12*a*^ on the hydrogens. The following extrapolation equations were utilized for the Hartree-Fock^19^ (*E*_HF_) and correlation energies^20^ (*ε*):

1 *E*_HF_(*X*) = *E*_HF_(∞) + *A*e^−*bX*^

2 *ε*(*X*) = *ε*_∞_ + *BX*^−3^

where *X* is the cardinal number of the aug′-cc-pV*X*Z basis sets [*X* = (3,4,5) for HF; (4,5) for MP2; and (3,4) for CCSD and CCSD(T)]. Ancillary FPA reaction energies and barrier heights obtained with the non-augmented cc-pV*X*Z series are presented in Table S2 (ESI†); these results differ from the more tightly converged aug′-cc-pV*X*Z predictions by no more than 0.17 kcal mol^−1^, in accord with previous findings.^21^ Increments for the higher-order CCSDT and CCSDT(Q) levels of theory were obtained using the following additivity schemes:

3 *δ*[CCSDT] = Δ*E*(CCSDT/cc-pVDZ) − Δ*E*(CCSD(T)/cc-pVDZ)

4 *δ*[CCSDT(Q)] = Δ*E*(CCSDT(Q)/cc-pVDZ) − Δ*E*(CCSDT/cc-pVDZ)

Numerous studies have demonstrated the effectiveness of such CCSDT and CCSDT(Q) corrections computed with the double-ζ basis set, a consequence of the general basis set insensitivity of high-order correlation increments.^22^

Core-electron correlation effects were quantified by subtracting all-electron (AE) and frozen-core (FC) CCSD(T) energies computed with the cc-pCVTZ basis set:^12*c*^

5 Δ[core] = Δ*E*(AE-CCSD(T)/cc-pCVTZ) − Δ*E*(FC-CCSD(T)/cc-pCVTZ)

A first-order relativistic correction,^23^ Δ(rel), was obtained from the one-electron mass-velocity and Darwin terms at the CCSD(T)/cc-pVTZ level of theory. The diagonal Born-Oppenheimer correction (DBOC)^24^ was computed at the HF/aug-cc-pVTZ level of theory. The Δ(rel) and Δ(DBOC) calculations were performed with the CFOUR 2.0 package.^25^ In all cases the auxiliary Δ corrections are less than 0.25 kcal mol^−1^ in magnitude, confirming the use of frozen-core, nonrelativistic, clamped-nucleus electronic wave functions in the primary FPA determinations. In particular, the minuscule DBOC shifts (<0.08 kcal mol^−1^) indicate that our pericyclic reactions are not complicated by surface crossings near the transition states.

### 2.2 Density functional theory geometric structures and relative energies

The key stationary points on the potential energy surfaces of the pericyclic reactions were optimized with the generalized gradient approximation BP86,^26^ which has been shown to give accurate geometric structures^27^ and is frequently employed for pericyclic reactions.^4*a*,*b*,7*d*^ The BP86 functional was implemented with a hierarchical series of Slater-type (STO) basis sets (DZ, DZP, TZ2P, QZ4P) of double (D)-, triple (T), and quadruple (Q)-ζ quality with one (P), two (2P), or four (4P) sets of polarization functions.^28^ To gauge dispersion effects on the structures, the stationary points were also optimized using Grimme's D3 dispersion correction and Becke-Johnson damping,^29^ as denoted by BP86-D3(BJ)/QZ4P. The accuracies of the fit scheme (Zlm fit)^30*a*^ and the integration grid (Becke grid)^30*b*^ were set to VERYGOOD. As in the benchmark computations, the character of each DFT stationary point was verified by performing a harmonic vibrational analysis,^31^ including careful inspection of the normal mode of imaginary frequency for each transition state. Finally, single-point energies of the stationary points were computed using the TZ2P and QZ4P basis sets in combination with a panoply of density functionals: the local-density approximation (LDA) functional VWN;^32^ the generalized gradient approximation (GGA) functionals BP86,^26^ BLYP,^26*a*,33^ BEE,^34^ PW91,^35^ PBE,^34*b*^ PBEsol,^36^ RPBE,^34*b*,37^ revPBE,^34*b*,38^ mPBE,^34*b*,39^ mPW,^35,40^ HTBS,^34*b*,41^ OLYP,^33,42^ OPBE,^34*b*,42,43^ and XLYP;^33,44^ the meta-GGA functionals M06-L,^45^ MVS,^46^ TPSS,^47^ and revTPSS;^48^ the hybrid functionals B3LYP,^33,49,50^ B3LYP*,^33,49,51^ B1LYP,^33,49,52^ B1PW91,^35,49,52^ BHandH,^53^ BHandHLYP,^53^ KMLYP,^54^ O3LYP,^55^ OPBE0,^43^ PBE0,^56^ mPW1PW,^35,40^ mPW1K,^57^ S12H,^58^ and X3LYP;^44^ the meta-hybrid functionals M06,^45^ M06-2X,^45^ M06-HF,^45^ and TPSSH;^47^ the double-hybrid functionals B2K-PLYP,^59^ B2T-PLYP,^59^ B2-PLYP,^7*b*^ LS1-TPSS,^60^ mPW2K-PLYP,^59^ mPW2-PLYP,^61^ PBE0-DH,^62^ revDSD-BLYP,^63^ revDSD-PBE,^63^ and revDSD-PBEP86;^63^ the Range-Separated Hybrid functionals CAM-B3LYP,^64^ CAMY-B3LYP,^64,65^ ωB97,^66^ ωB97X,^66^ and ωB97X-D;^69^ and the dispersion-corrected functionals BP86-D3(BJ),^26,29^ BLYP-D3(BJ),^26*a*,29,33^ PBE-D3(BJ),^29,34*b*^ OLYP-D3(BJ),^29,33,42^ OPBE-D3(BJ),^29,34*b*,42,43^ B3LYP-D3(BJ),^29,33,49,50^ PBE0-D3(BJ),^29,56^ and M06-2X-D3.^29,45^ All density functional theory (DFT) computations were performed using the Amsterdam Density Functional (ADF) software package.^67^

## 3. Results and discussion

### 3.1 Benchmark pericyclic reaction stationary points

**Fig. 1:**
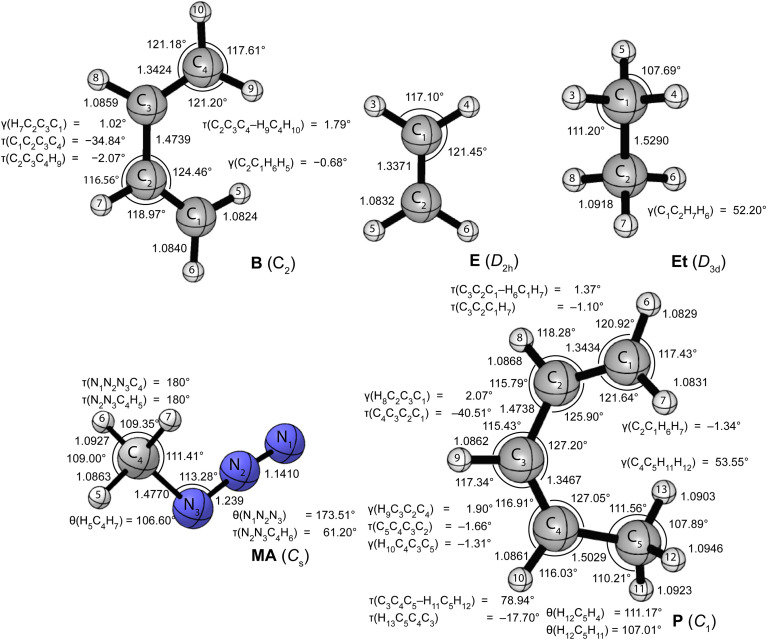
CCSD(T)/cc-pVTZ optimized geometries (in Å, deg) of the reactants of the pericyclic reactions depicted in Scheme 1.

**Fig. 2:**
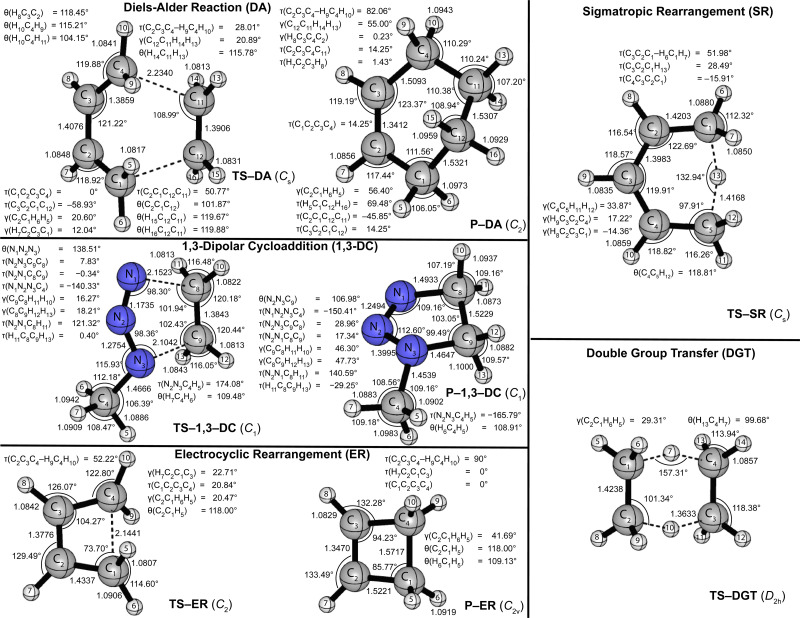
CCSD(T)/cc-pVTZ optimized geometries (in Å, deg) of transition states and products of the pericyclic reactions depicted in Scheme 1.

Fig. 1 and 2 provide the CCSD(T)/cc-pVTZ optimized geometries for the critical stationary points of the Diels-Alder (DA), 1,3-dipolar cycloaddition (1,3-DC), electrocyclic rearrangement (ER), sigmatropic rearrangement (SR), and double group transfer (DGT) reactions. Cartesian coordinates of these structures are given in Table S3 of the ESI.†

The Diels-Alder reaction between 1,3-*cis*-butadiene (B) and ethylene (E) proceeds *via* a concerted synchronous transition state (TS-DA) to the six-membered cycloadduct (P-DA). The transition state is *C*_s_-symmetric with two nascent C⋯C bond distances of 2.234 Å between the terminal carbon atoms of B and E. The C=C double bonds in B and E stretch by 0.167 and 0.194 Å to 1.509 and 1.531 Å, respectively, and become C–C single bonds in the cycloadduct P-DA; at the transition state, 26% and 28% of this evolution has occurred. Simultaneously, the central C–C bond of B shortens from 1.474 Å to 1.341 Å and becomes a C=C double bond in P-DA, this contraction being 50% complete in TS-DA. Twisting and pyramidalization of methylene groups are key motifs of the DA reaction. The twisting is best characterized by the dihedral angle [*τ*_twist_ = *τ*(C_2_C_3_C_4_–H_9_C_4_H_10_) = *τ*(C_1_C_2_C_3_–H_5_C_1_H_6_)] between the terminal CH_2_ plane and that of the connected C–C–C backbone in B. In particular, *τ*_twist_ starts at 1.8° in B and ends at 82.1° in P-DA, while displaying 33% of this change in TS-DA. The pyramidalization is quantified by the angles formed by the C–C bond vectors out of the CH_2_ planes [*γ*_pyr_(B) = *γ*(C_2_C_1_H_6_H_5_) = *γ*(C_3_C_4_H_10_H_9_) and *γ*_pyr_(E) = *γ*(C_12_C_11_H_14_H_13_) = *γ*(C_11_C_12_H_15_H_16_)]. As the terminal carbon atoms hybridizes from sp^2^ to sp^3^, *γ*_pyr_(B) rises from almost 0° in reactant B to 56.4° in P-DA, achieving 37% of this transformation in TS-DA. In remarkable accord, *γ*_pyr_(E) likewise changes from 0° in E to 55.0° in P-DA, exhibiting 38% of this increase in TS-DA. Overall, the key geometric parameters reflective of bond rearrangement consistently indicate that TS-DA is an “early” transition state, nicely consistent with the Hammond Postulate and the large exothermicity (Δ*H*_0_ = −40.6 kcal mol^−1^) of the reaction.

The 1,3-dipolar cycloaddition between methyl azide (MA) and ethylene (E) proceeds, in contrast to the Diels-Alder case, *via* a concerted asynchronous transition state (TS-1,3-DC) to the five-membered cycloadduct (P-1,3-DC). The nascent N_1_–C_8_ and N_3_–C_9_ bonds in the transition state (2.152 and 2.104 Å) differ in length by 0.048 Å, and this marked asymmetry persists in the N–C bonds of the cycloadduct (1.493 and 1.465 Å). The two N⋯C distances in TS-1,3-DC were previously computed by Blavins and Karadakov^9*c*^ with a variety of methods (HF, MP2, B3LYP, CCD, QCISD, MP4) and small Pople basis sets [6-31G(d) or 6-31G(d,p)]. Their N_1_–C_8_ and N_3_–C_9_ results span the ranges 2.102–2.159 and 2.028–2.178 Å, respectively, with the QCISD distances coming within −0.006 and +0.011 Å of our CCSD(T)/cc-pVTZ values. The N_1_–N_2_, N_2_–N_3_, and C=C multiple bonds of MA and E stretch by 0.108, 0.160, and 0.186 Å, respectively, in forming the cycloadduct; the percentage of this elongation found in the transition state is 30%, 23%, and 25%, in order. Regarding pyramidalization of the ethylene moiety, the out-of-plane angles [*γ*(C_9_C_8_H_11_H_12_), *γ*(C_8_C_9_H_12_H_13_)] increase from 0° in the reactant to 46.30° and 47.73°, respectively, in the product, while comprising 35% and 38%, respectively, of this transformation in TS-1,3-DC. Therefore, the key bond distances and pyramidalization angles show uniform trends that characterize TS-1,3-DC as a prototypical “early” transition state in accord with the Hammond Postulate and the reaction exothermicity (Δ*H*_0_ = −23.2 kcal mol^−1^). The azide bending angle is incongruous in this respect, because it contracts from 173.5° in MA to 112.6° in the cycloadduct and exhibits more than half of this deformation in TS-1,3-DC. The spectator methyl substituent of the system actually shows non-monotonic internal rotation, as the torsion angle *τ*(N_2_N_3_C_4_H_5_) rocks from that in MA by −6° in TS-1,3-DC but +14° in P-1,3-DC.

The electrocyclic rearrangement of 1,3-*cis*-butadiene is an intramolecular concerted cyclization in which a new C–C bond is formed between the terminal sp^2^-hybridized carbon atoms of B. The *C*_s_-symmetric transition state (TS-ER) has a nascent C⋯C bond distance of 2.144 Å, which is reduced to 1.572 Å in the strained, four-membered cycloadduct (P-ER, *C*_2v_). These distances are 0.010 and 0.007 Å longer, respectively, than the CCSD/6-311++G(df,p) results of Minkin and coworkers.^9*b*^ During the reaction, the C–C single bond of B contracts from 1.474 Å to become a C=C double bond of length 1.347 Å in P-ER, and 76% of this decrease has occurred in the transition state. Simultaneously, the C=C double bonds of B elongate by 0.180 Å to become C–C single bonds in P-ER, exhibiting 51% of this change in TS-ER. The C–C–C angle changes from 124.5°(B) → 104.3°(TS-ER) → 94.2°(P-ER) during the reaction, exhibiting 67% completion in the transition state. As in the DA reaction, the ER process requires twisting and pyramidalization of the terminal methylene groups in B. The aforementioned *τ*_twist_ angle starts at 1.8° in B, ends at 90° in P-DA, and has achieved 57% of this change in TS-DA. Because the ER reaction is endothermic (Δ*H*_0_ = +9.6 kcal mol^−1^), a “late” transition state is anticipated, consistent with the trends seen in the characteristic geometric parameters.

The SR transformation of (*Z*)-penta-1,3-diene (P) is an intramolecular identity reaction comprising a [1,5]-sigmatropic hydrogen shift between the terminal carbon atoms. The transition state (TS-SR) has *C*_s_ symmetry, with a 1.417 Å distance for both the breaking and forming C⋯H bonds. The reorganization of the π-system causes the backbone bond distances, *i.e.*, C_1_–C_2_, C_2_–C_3_, C_3_–C_4_, C_4_–C_5_, to go from 1.343, 1.474, 1.347, 1.503 Å in the reactant (P), to 1.420, 1.398, 1.398, 1.420 Å in TS-SR, and finally to the re-ordered values 1.503, 1.347, 1.474, 1.343 Å in the identity product (P). The corresponding evolutions of these backbone distances in TS-SR (48%, 59%, 41%, 52%) are symmetrically displaced from the 50% progression simplistically expected for an identity reaction. The backbone bond angles of P, [*θ*(C_1_–C_2_–C_3_), *θ*(C_2_–C_3_–C_4_), *θ*(C_3_–C_4_–C_5_)] = 125.9°, 127.2°, 127.0°, contract by 3.2°, 7.3°, and 4.3° to form TS-SR before relaxing back to the initial values in reverse order. As terminal carbon C_1_ in the reactant transitions from sp^2^ to sp^3^ hybridization, the out-of-plane angle *γ*(C_2_C_1_H_6_H_7_) undergoes the progression −1.3° (P) → 33.9° (TS-SR) → 53.6° (P), whereas the methylene twist angle, *τ*_twist_ = *τ*(C_3_C_2_C_1_–H_6_C_1_H_7_), displays the simultaneous evolution 1.4°(P) → 52.0°(TS-SR) → 78.9°(P). In both the pyramidalization and twisting cases, about 65% of the total reorganization occurs before the transition state is reached. Notably, the expectation of the Hammond Postulate of a central transition state for a thermoneutral reaction is not generally upheld for the various SR geometric transformations.

The DGT identity reaction between ethane (Et) and ethylene (E) proceeds in a concerted and synchronous fashion with two hydrogen atoms migrating simultaneously from Et to E. The corresponding transition state (TS-DGT) of *D*_2h_ symmetry features a six-membered ring in which the breaking C–H bonds are elongated by 0.272 Å to 1.363 Å and the newly forming bonds have the same distance. Comparing with the work from Radom and coworkers^9*a*^ reveals that CCSD(T)/6-31G(d,p) theory gives the C⋯H distance and C⋯H⋯C angle of TS-DGT within 0.003 Å and 0.5°, respectively, of our benchmark values. The DGT switches the C–C and C=C bonds in the fragments, and the dual 1.424 Å distances in the transition state correspond to 55% of the contraction and 45% of the elongation that occurs in the overall reaction. TS-DGT exhibits four equivalent pyramidalizations, which all have a 29.3° angle of the C–C bonds out of adjacent methylene plane; these angles represent 56% of the total shift occurring in the E(sp^2^) → Et(sp^3^) rehybridization. The TS-DGT carbon–carbon distances and pyramidalization angles agree with simple expectations for an identity reaction. However, this accord is not met by the C–H bonds peripheral to the hydrogen exchange; in particular, the corresponding distances and methylene angles in TS-DGT respectively constitute only 29% of the increase and 34% of the decrease involved in the E(sp^2^) → Et(sp^3^) transformation.

### 3.2 Benchmark pericyclic reaction energetics

The focal-point analyses to determine the benchmark reaction energies and barriers for our five paradigmatic pericyclic reactions are presented in Tables 1 and 2. The two-dimensional grids detail the systematic convergence of the energetic predictions toward both the complete basis set (vertical) and electron correlation (horizontal) limits, as approached *via* the aug′-cc-pV*X*Z atomic-orbital series (*X* = D, T, Q, 5) and the HF → MP2 → CCSD → CCSD(T) → CCSDT(Q) wave function hierarchy, respectively. Attesting to the excellent basis set convergence in all eight FPA tables, the explicitly computed aug′-cc-pV5Z results for RHF relative energies and MP2 correlation increments display mean absolute deviations of only 0.02 and 0.09 kcal mol^−1^, respectively, with respect to the extrapolated CBS values. The entry-level RHF/CBS method greatly overestimates the barriers by 13.5–32.3 kcal mol^−1^, while the corresponding reaction energy errors range from +2.4 to +9.0 kcal mol^−1^. The MP2 corrections to the barriers are all negative and overly large in magnitude, to the extent that MP2/CBS theory underestimates the barriers by 2.8–9.4 kcal mol^−1^; concomitantly, the reaction energy errors vary from −5.2 to +3.5 kcal mol^−1^. Oscillations of computed barrier heights continue in the next step of the electron correlation hierarchy, as the CCSD/CBS correlation increments are all positive and this method overestimates the barriers by 3.2–6.4 kcal mol^−1^; however, the corresponding mean absolute error in the reaction energies is now 1.2 kcal mol^−1^. Predictably, the (T) increments for all barriers are negative, reducing the overestimation of the barriers to only 0.08–0.26 kcal mol^−1^ at the CCSD(T)/CBS level of theory; all reaction energies given by this method are accurate to better than 0.8 kcal mol^−1^. The CCSD(T) → CCSDT and CCSDT→ CCSDT(Q) contributions are opposite in sign and largely cancel one another for the barriers, giving very small net *δ*[CCSDT(Q)] corrections between −0.07 and −0.16 kcal mol^−1^ that are shown in brackets in the FPA tables; in contrast, for the reaction energies the CCSD(T) → CCSDT and CCSDT→ CCSDT(Q) terms have the same sign and individually span the interval 0.03–0.65 kcal mol^−1^.

**Table 1:** Focal point analysis of the reaction energies (in kcal mol^−1^) for the pericyclic reactions of Scheme 1^a^

Basis set	Δ*E*_e_(HF)	+*δ*[MP2]	+*δ*[CCSD]	+*δ*[CCSD(T)]	+*δ*[CCSDT(Q)]	NET
Diels-Alder reaction						
cc-pVDZ (134)	−42.71	−11.94	5.26	0.45	0.14 + 0.35	−48.46
aug′-cc-pVDZ (188)	−42.22	−11.66	4.64	0.50	[0.49]	[−48.25]
aug′-cc-pVTZ (416)	−38.82	−13.94	4.44	0.46	[0.49]	[−47.36]
aug′-cc-pVQZ (780)	−38.64	−13.97	4.36	0.44	[0.49]	[−47.33]
aug′-cc-pV5Z (1312)	−38.58	−14.10	[4.33]	[0.43]	[0.49]	[−47.44]
CBS LIMIT	[−38.56]	[−14.24]	[4.30]	[0.42]	[0.49]	[−47.59]
Δ*E*_final_ = Δ*E*_e_(NET/CBS) + Δ(DBOC) + Δ(rel) + Δ(core) = −47.59 + 0.02 + 0.13 − 0.21 = **−47.65 kcal mol^−1^** Δ*E*_0_ = Δ*E*_final_ + Δ(ZPVE) = −47.65 + 6.75 = −40.90 kcal mol^−1^						

1,3-Dipolar cycloaddition						
cc-pVDZ (119)	−30.34	6.08	−5.92	1.85	0.11 + 0.65	−27.56
aug′-cc-pVDZ (173)	−31.13	3.51	−5.70	1.38	[0.76]	[−31.17]
aug′-cc-pVTZ (374)	−26.94	1.68	−5.67	1.55	[0.76]	[−28.61]
aug′-cc-pVQZ (690)	−26.75	1.48	−5.69	1.51	[0.76]	[−28.68]
aug′-cc-pV5Z (1147)	−26.65	1.24	[−5.70]	[1.50]	[0.76]	[−28.83]
CBS LIMIT	[−26.60]	[1.00]	[−5.70]	[1.49]	[0.76]	[−29.05]
Δ*E*_final_ = Δ*E*_e_(NET/CBS) + Δ(DBOC) + Δ(rel) + Δ(core) = −29.05 − 0.02 + 0.17 + 0.04 = **−28.86 kcal mol^−1^**						
Δ*E*_0_ = Δ*E*_final_ + Δ(ZPVE) = −28.86 + 5.28 = −23.58 kcal mol^−1^						

Electrocyclic rearrangement						
cc-pVDZ (86)	10.78	−5.07	2.15	0.32	0.19 + 0.03	8.40
aug′-cc-pVDZ (122)	10.91	−3.99	1.61	0.38	[0.22]	[9.13]
aug′-cc-pVTZ (268)	11.98	−5.39	1.74	0.31	[0.22]	[8.86]
aug′-cc-pVQZ (500)	12.01	−5.64	1.75	0.28	[0.22]	[8.62]
aug′-cc-pV5Z (838)	12.02	−5.77	[1.76]	[0.27]	[0.22]	[8.50]
CBS LIMIT	[12.03]	[−5.91]	[1.76]	[0.26]	[0.22]	[8.36]
Δ*E*_final_ = Δ*E*_e_(NET/CBS) + Δ(DBOC) + Δ(rel) + Δ(core) = 8.36 + 0.01 + 0.05 + 0.03 = **8.45 kcal mol^−1^**						
Δ*E*_0_ = Δ*E*_final_ + Δ(ZPVE) = 8.45 + 1.07 = 9.52 kcal mol^−1^						

a The symbol *δ* denotes the increment in the relative energy with respect to the preceding level of theory in the hierarchy RHF → MP2 → CCSD → CCSD(T) → CCSDT(Q). The total number of contracted Gaussian functions in each basis set is given in parentheses. Square brackets signify results obtained from basis set extrapolations or additivity assumptions, as detailed in the Theoretical methods section. The bracketed CCSD increments are based on differences of corresponding aug′-cc-pV{T,Q}Z extrapolations of MP2 and CCSD correlation energies. The CCSDT(Q) increment is separated into its CCSD(T) → CCSDT and CCSDT → CCSDT(Q) components for the cc-pVDZ basis but entered as a single, bracketed correction in the remaining rows. The sum across each row comprises the NET column entry. Beneath each FPA grid, auxiliary terms (Δ) for the diagonal Born-Oppenheimer correction (DBOC), scalar relativistic effects (rel), and core electron correlation (core) are added to NET/CBS LIMIT to arrive at the final, vibrationless result (Δ*E*_final_; boldfaced) used to calibrate the DFT methods. For completeness, CCSD(T)/cc-pVTZ zero-point vibrational corrections are then appended to obtain Δ*E*_0_ values.

**Table 2:** Focal point analysis of the reaction barriers (in kcal mol^−1^) for the pericyclic reactions of Scheme 1^a^

Basis set	Δ*E*_e_(HF)	+*δ*[MP2]	+*δ*[CCSD]	+*δ*[CCSD(T)]	+*δ*[CCSDT(Q)]	NET
Diels-Alder reaction						
cc-pVDZ (134)	43.61	−31.21	13.60	−4.85	0.46–0.53	21.07
aug′-cc-pVDZ (188)	43.41	−34.54	14.40	−5.35	[−0.07]	[17.86]
aug′-cc-pVTZ (416)	45.10	−35.88	15.13	−5.78	[−0.07]	[18.50]
aug′-cc-pVQZ (780)	45.39	−35.68	15.32	−5.90	[−0.07]	[19.06]
aug′-cc-pV5Z (1312)	45.45	−35.59	[15.39]	[−5.94]	[−0.07]	[19.24]
CBS LIMIT	[45.47]	[−35.49]	[15.47]	[−5.99]	[−0.07]	[19.38]
Δ*E*_final_ = Δ*E*_e_(NET/CBS) + Δ(DBOC) + Δ(rel) + Δ(core) = 19.38 + 0.04 + 0.03 + 0.11 = **19.56 kcal mol^−1^**						
Δ*E*_0_ = Δ*E*_final_ + Δ(ZPVE) = 19.56 + 2.38 = 21.94 kcal mol^−1^						

1,3-Dipolar cycloaddition						
cc-pVDZ (119)	39.31	−27.73	10.95	−4.64	0.70–0.82	17.77
aug′-cc-pVDZ (173)	40.60	−32.84	12.31	−5.49	[−0.12]	[14.47]
aug′-cc-pVTZ (374)	43.22	−33.33	13.12	−5.78	[−0.12]	[17.11]
aug′-cc-pVQZ (690)	43.49	−33.10	13.34	−5.89	[−0.12]	[17.73]
aug′-cc-pV5Z (1147)	43.60	−33.06	[13.42]	[−5.93]	[−0.12]	[17.91]
CBS LIMIT	[43.65]	[−33.03]	[13.50]	[−5.98]	[−0.12]	[18.03]
Δ*E*_final_ = Δ*E*_e_(NET/CBS) + Δ(DBOC) + Δ(rel) + Δ(core) = 18.03 + 0.02 + 0.01 + 0.23 = **18.29 kcal mol^−1^**						
Δ*E*_0_ = Δ*E*_final_ + Δ(ZPVE) = 18.29 + 1.71 = 20.00 kcal mol^−1^						

Electrocyclic rearrangement						
cc-pVDZ (86)	56.42	−15.23	4.93	−2.64	0.16–0.29	43.35
aug′-cc-pVDZ (122)	55.85	−15.60	5.19	−2.75	[−0.13]	[42.56]
aug′-cc-pVTZ (268)	56.55	−16.34	5.79	−3.01	[−0.13]	[42.87]
aug′-cc-pVQZ (500)	56.70	−16.32	5.93	−3.08	[−0.13]	[43.10]
aug′-cc-pV5Z (838)	56.72	−16.32	[5.99]	[−3.10]	[−0.13]	[43.16]
CBS LIMIT	[56.73]	[−16.32]	[6.04]	[−3.13]	[−0.13]	[43.19]
Δ*E*_final_ = Δ*E*_e_(NET/CBS) + Δ(DBOC) + Δ(rel) + Δ(core) = 43.19 + 0.04 + 0.01 + 0.20 = **43.44 kcal mol^−1^**						
Δ*E*_0_ = Δ*E*_final_ + Δ(ZPVE) = 43.44 − 0.78 = 42.66 kcal mol^−1^						

Sigmatropic rearrangement						
cc-pVDZ (110)	53.88	−22.92	9.46	−3.57	0.27–0.41	36.71
aug′-cc-pVDZ (155)	53.71	−23.24	9.77	−3.69	[−0.14]	[36.40]
aug′-cc-pVTZ (342)	54.90	−24.18	10.24	−4.12	[−0.14]	[36.71]
aug′-cc-pVQZ (640)	54.98	−24.32	10.34	−4.21	[−0.14]	[36.64]
aug′-cc-pV5Z (1075)	55.00	−24.34	[10.37]	[−4.25]	[−0.14]	[36.64]
CBS LIMIT	[55.01]	[−24.36]	[10.41]	[−4.29]	[−0.14]	[36.63]
Δ*E*_final_ = Δ*E*_e_(NET/CBS) + Δ(DBOC) + Δ(rel) + Δ(core) = 36.63 + 0.08 + 0.00 + 0.08 = **36.79 kcal mol^−1^**						
Δ*E*_0_ = Δ*E*_final_ + Δ(ZPVE) = 36.79 − 4.23 = 32.56 kcal mol^−1^						

Double group transfer						
cc-pVDZ (106)	80.06	−36.85	12.53	−4.69	0.30–0.56	50.79
aug′-cc-pVDZ (142)	80.08	−38.54	13.03	−5.09	[−0.16]	[49.21]
aug′-cc-pVTZ (324)	82.15	−40.08	13.88	−5.83	[−0.16]	[49.85]
aug′-cc-pVQZ (620)	82.33	−40.16	14.08	−6.00	[−0.16]	[49.99]
aug′-cc-pV5Z (1058)	82.39	−40.13	[14.15]	[−6.06]	[−0.16]	[50.03]
CBS LIMIT	[82.41]	[−40.11]	[14.22]	[−6.12]	[−0.16]	[50.14]
Δ*E*_final_ = Δ*E*_e_(NET/CBS) + Δ(DBOC) + Δ(rel) + Δ(core) = 50.14 + 0.07 + 0.02 + 0.00 = **50.23 kcal mol^−1^**						
Δ*E*_0_ = Δ*E*_final_ + Δ(ZPVE) = 50.23 − 2.44 = 47.79 kcal mol^−1^						

a Please see the footnote of Table 1 for notation.

The ratios of successive correlation increments provide an important gauge of convergence. For the reaction barriers, the CBS values of *δ*[CCSD]/*δ*[MP2] cluster in the interval 0.35–0.44, whereas the corresponding *δ*[CCSD(T)]/*δ*[CCSD] ratios are in the similar range 0.39–0.52. With the cc-pVDZ basis set, the CCSD(T) → CCSDT and CCSDT→ CCSDT(Q) contributions divided by *δ*[CCSD(T)] lie in the ranges 0.05–0.12 and 0.09–0.14, respectively. Considering such indicators, the individual post-CCSDT(Q) increments for the reaction barriers should be substantially less than half the magnitude of the preceding CCSD(T) → CCSDT and CCSDT→ CCSDT(Q) shifts; moreover, favorable cancellations of high-order increments are likely. Accordingly, we surmise that our FPA barriers are converged at least to an accuracy of 0.2 kcal mol^−1^. A similar analysis of the reaction energies suggests somewhat weaker convergence and an error estimate of 0.3–0.4 kcal mol^−1^. An overall conclusion applicable to all of our FPA energetic predictions is that the final residual errors are no larger than a few tenths of a kcal mol^−1^.

Supplementing the Δ*E*_e_(NET/CBS) entries in Tables 1 and 2 with the small Δ(DBOC), Δ(rel), and Δ(core) auxiliary terms yields the boldfaced, vibrationless results (Δ*E*_final_) used to calibrate DFT methods: −47.7 (DA), −28.9 (1,3-DC), 8.5 (ER) kcal mol^−1^ for reaction energies, and 19.6 (DA), 18.3 (1,3-DC), 43.4 (ER), 36.8 (SR), 50.2 (DGT) kcal mol^−1^ for reaction barriers. For comparison with experimental observations and other computational data in the literature, Δ(ZPVE) corrections from our CCSD(T)/cc-pVTZ harmonic vibrational frequencies are appended to the Δ*E*_final_ results to obtain Δ*E*_0_ predictions: −40.9 (DA), −23.6 (1,3-DC), 9.5 (ER) kcal mol^−1^ for reaction energies, and 21.9 (DA), 20.0 (1,3-DC), 42.7 (ER), 32.6 (SR), 47.8 (DGT) kcal mol^−1^ for reaction barriers.

The 2019 paper by Karton^8^ reports W3lite-F12 computations of three reaction barriers (without ZPVE corrections) related to our pericyclic reaction systems: TS-DA arising from the Diels-Alder reaction between E and the *trans*-isomer of B, ring opening reaction (ROR) of P-ER, that is, the reverse reaction of the electrocyclic rearrangement (TS-ER with respect to P-ER), and TS-SR originating from the sigmatropic rearrangement of the *E*-isomer of P. Note, the *trans*-isomer of B and the *E*-isomer of P are not reached by the intrinsic reaction paths connected to TS-DA and TS-SR, but these isomers are lower in energy than their *cis* and *Z* counterparts shown in Fig. 1. We expanded our computations to include B(*trans*) and P(*E*) and re-worked the FPA tables to obtain the following Δ*E*_final_ barrier predictions: 22.53 (TS-DA), 34.99 (TS-ROR), and 39.76 (TS-SR) kcal mol^−1^ (Table S2, ESI†). The corresponding values of Karton are 22.46 (TS-DA), 34.99 (TS-ROR), and 39.85 (TS-SR) kcal mol^−1^, so that the largest difference between the W3lite-F12 and FPA barriers is a mere 0.09 kcal mol^−1^. Notwithstanding this close agreement and the fact that both approaches include correlation effects up to CCSDT(Q)/cc-pVDZ, our FPA computations are more favorable in several respects for the three cases of concern. (1) Geometric structures were optimized at the CCSD(T)/cc-pVTZ level of theory rather than at B3LYP-D3(BJ)/cc-pVTZ. (2) Aug′-cc-pV5Z HF and MP2 energies were used to assist the FPA extrapolations to the CBS limit, as opposed to omitting MP2 in the hierarchy and employing the cc-pVQZ-F12 approach for the largest explicit computations. (3) More complete basis sets with diffuse heavy-atom functions [aug′-cc-pV{T,Q}Z] were utilized to extrapolate CCSD increments *via* an established *X*^−3^ form to the CBS limit, in contrast to evaluating such increments by means of cc-pV{T,Q}-F12 extrapolations with a phenomenological *X*^−5.94^ function. (4) CCSD(T) increments were also obtained from aug′-cc-pV{T,Q}Z extrapolations to the CBS limit rather than by scaling cc-pVTZ-F12 results by an *ad hoc* factor of 0.987. (5) CCSD(T)/cc-pVTZ harmonic vibrational frequencies and ZPVEs of all stationary points were computed as opposed to a complete omission of all vibrational effects. (6) The DBOC terms were evaluated and used to confirm the absence of surface crossing phenomena in the pericyclic reactions. (7) Reaction energies were computed rather than forward barriers alone, thus providing results of the same rigor for the reverse reactions.

### 3.3 Assessment of DFT geometric structures

In this section, geometric structures of the reactants, transition states, and products of each pericyclic reaction were fully optimized with five DFT methods: BP86/DZ, BP86/DZP, BP86/TZ2P, BP86/QZ4P, and BP86-D3(BJ)/QZ4P. Cartesian coordinates of these DFT structures are provided in Tables S4–S8 (ESI†). To assess the quality of these DFT geometries, they were compared to the CCSD(T)/cc-pVTZ benchmark geometries by means of a Cartesian root-mean-square deviation (RMSD) analysis. The procedure rigorously solved for the 3 origin-translation and 3 axis-rotation variables that provide the minimum possible RMSD between the Cartesian coordinates of a given DFT geometry and those of the corresponding CCSD(T)/cc-pVTZ benchmark geometry. The solution to this minimization problem in the translational space is always achieved by making the centroids exactly coincident for the two structures under comparison. However, the RMSD minimization in the rotational space is *not* achieved in the general case by assuming unit masses and aligning the principal axes of inertia, although this approach often yields an excellent approximation. Analytic solutions of the RMSD rotational minimization problem have been reported by Kabsch,^68^ and we have derived improved analytic formulas from the Euler-Rodrigues representation of the associated unitary matrix. Finally, we wrote a *Mathematica* program to execute numerical searches for the desired solutions (see ESI,† for *Mathematica* code CartRMSD). All three approaches gave identical results for every set of molecular structures, making our RMSD minimizations definitive.

**Fig. 3:**
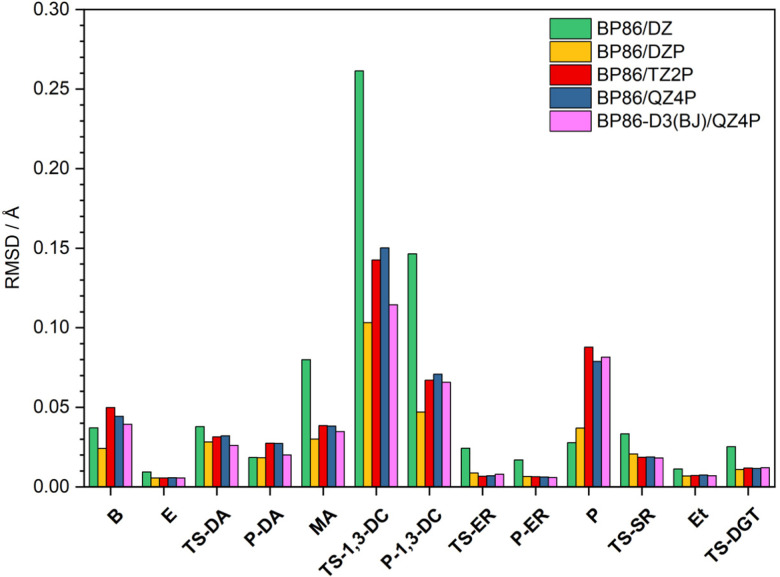
Global-minimum Cartesian root-mean-square deviation (RMSD) values (in Å) of the fully optimized geometries at various DFT levels of theory with respect to the CCSD(T)/cc-pVTZ benchmarks.

The global-minimum Cartesian RMSD values for the five DFT methods are plotted in Fig. 3 for all stationary points of our pericyclic reactions. Note that for all methods the largest deviations occur for the transition state of the 1,3-dipolar cycloaddition reaction (TS-1,3-DC). Interestingly, the BP86/DZP geometries generally display the smallest deviations from the CCSD(T)/cc-pVTZ benchmarks with an average RSMD of 0.027 Å (Table S9, ESI†). Expanding the BP86 basis set to TZ2P and QZ4P generally increases the RMSD and thus does not improve accuracy despite the added computational expense. Moreover, the inclusion of Grimme's D3 dispersion in conjunction with Becke-Johnson damping does not always improve the BP86/QZ4P geometries, and overall BP86-D3(BJ)/QZ4P is still substantially inferior to BP86/DZP.

In order to pinpoint where the DFT geometries exhibit disparities, we considered all geometric parameters identified in Fig. 1 and 2; and calculated the average percentage error of the distances, angles, and dihedral angles with respect to the CCSD(T)/cc-pVTZ benchmarks (Table S10, ESI†). Consistent with Fig. 3, the percentage errors of the distances, angles, and dihedral angles have both the smallest average and the smallest maximum for the BP86/DZP method. Thus, a pragmatic approach to obtain geometries closest to CCSD(T)/cc-pVTZ structures is to optimize stationary points at BP86/DZP. Independent of the DFT level of theory, the largest percentage errors in the internal coordinates are found for dihedral angles, and these errors are responsible for most of the deviations seen in the Cartesian RMSD analysis. In particular, dramatic deficiencies occur for the dihedral angles of the B, TS-1,3-DC, P-1,3-DC, and P stationary points. In contrast, the bond distances and angles for all DFT levels of theory differ on average by less than 1% from the CCSD(T)/cc-pVTZ reference values.

### 3.4 Performance of DFT methods for energetics

Next, we examine the relative energies of the stationary points computed with the density functionals (XC) specified in the Theoretical methods section in conjunction with a QZ4P Slater-type basis set atop the CCSD(T)/cc-pVTZ geometries, denoted as XC/QZ4P//CCSD(T)/cc-pVTZ. The DFT reaction barriers and reaction energies for the five pericyclic reactions are collected in Table 3. The performance of the various density functional approximations is assessed by a systematic comparison of the resulting reaction barriers and energies with our most accurate CCSDT(Q)/CBS benchmark values (Table 1). For all 60 functionals, we have computed the mean error (ME), mean absolute error (MAE), maximum unsigned error (MUE), and standard deviation (SD) of the reaction barriers, reaction energies, as well as the combination of both, relative to the FPA CCSDT(Q)/CBS targeted energy values for all pericyclic reactions (Table 4). Note that the errors in reaction barriers and reaction energies for all stationary points computed at XC/QZ4P//CCSD(T)/cc-pVTZ with respect to CCSDT(Q)/CBS//CCSD(T)/cc-pVTZ can be found in Table S11 (ESI†).

**Table 3:** Reaction barriers (Δ*E*^‡^) and energies (Δ*E*_rxn_) (kcal mol^−1^) for the pericyclic systems of Scheme 1, computed using various density functional approximations at the XC/QZ4P//CCSD(T)/cc-pVTZ level

XC	Diels-Alder reaction		1,3-Dipolar cycloaddition		Electrocyclic rearrangement		Sigmatropic rearrangement		Double group transfer	
	Δ*E*^‡^	Δ*E*_rxn_	Δ*E*^‡^	Δ*E*_rxn_	Δ*E*^‡^	Δ*E*_rxn_	Δ*E*^‡^	Δ*E*_rxn_	Δ*E*^‡^	Δ*E*_rxn_
*LDA*										
VWN	−0.5	−65.4	2.5	−42.3	36.3	2.4	21.7	0.0	21.9	0.0
*GGAs*										
BP86	14.7	−42.3	15.6	−20.4	39.5	8.2	28.1	0.0	38.1	0.0
BLYP	21.3	−30.1	19.8	−10.6	42.6	13.7	31.7	0.0	45.3	0.0
BEE	15.7	−43.1	17.4	−19.6	38.8	6.2	27.9	0.0	38.4	0.0
PW91	12.0	−46.3	13.4	−23.8	39.2	7.3	27.6	0.0	35.7	0.0
PBE	12.4	−46.4	14.0	−23.4	38.9	6.6	27.4	0.0	35.8	0.0
PBEsol	5.8	−57.0	8.5	−33.5	36.6	3.2	23.8	0.0	28.3	0.0
RPBE	18.7	−38.1	19.5	−15.3	39.8	8.1	29.7	0.0	41.7	0.0
revPBE	18.3	−38.9	19.4	−15.9	39.6	7.7	29.3	0.0	41.2	0.0
mPBE	14.1	−44.1	15.5	−21.2	39.2	7.1	28.1	0.0	37.4	0.0
mPW	15.2	−41.9	16.3	−19.4	39.8	8.2	28.7	0.0	38.9	0.0
HTBS	13.2	−47.2	15.5	−23.7	37.4	4.6	25.9	0.0	35.5	0.0
OLYP	23.8	−36.1	24.3	−13.2	40.3	7.0	30.6	0.0	45.3	0.0
OPBE	18.5	−48.7	22.0	−22.1	36.6	−0.1	26.7	0.0	38.8	0.0
XLYP	21.7	−28.9	19.9	−9.7	43.0	14.5	32.4	0.0	46.1	0.0
*Meta-GGAs*										
M06-L	17.2	−46.2	20.7	−16.9	43.6	5.9	33.9	0.0	45.9	0.0
MVS	12.2	−54.5	18.5	−24.8	42.7	5.9	28.2	0.0	36.6	0.0
TPSS	15.4	−40.3	16.2	−19.5	40.1	7.7	30.8	0.0	43.0	0.0
revTPSS	14.4	−42.4	15.2	−22.7	39.6	6.1	31.4	0.0	44.9	0.0
*Hybrids*										
B3LYP	22.8	−37.6	22.0	−18.6	44.8	11.7	35.0	0.0	49.6	0.0
B3LYP*	20.8	−38.3	20.1	−19.0	43.7	11.4	33.4	0.0	46.7	0.0
B1LYP	24.3	−37.3	23.3	−19.0	45.6	12.0	36.4	0.0	52.0	0.0
B1PW91	19.3	−48.7	21.2	−26.7	42.4	5.7	33.0	0.0	46.1	0.0
BHandH	12.3	−66.7	14.2	−48.4	44.2	3.6	34.1	0.0	42.4	0.0
BHandHLYP	27.2	−44.6	26.6	−27.8	48.6	10.3	41.2	0.0	58.5	0.0
KMLYP	19.0	−60.5	20.5	−42.4	46.5	5.1	38.0	0.0	50.1	0.0
O3LYP	−3.3	−76.1	0.4	−53.5	37.3	0.2	22.7	0.0	21.5	0.0
OPBE0	20.5	−54.6	24.2	−29.8	40.0	−0.3	31.5	0.0	45.1	0.0
PBE0	16.1	−52.8	18.2	−30.8	41.8	4.8	32.1	0.0	42.9	0.0
mPW1PW	18.4	−49.1	20.1	−27.5	42.6	6.2	33.2	0.0	45.6	0.0
mPW1K	20.6	−54.2	22.7	−33.6	44.6	4.7	36.5	0.0	50.2	0.0
S12H	16.2	−53.4	19.3	−29.1	42.7	4.5	34.0	0.0	44.0	0.0
X3LYP	22.0	−39.2	21.3	−20.5	44.8	11.5	35.1	0.0	49.2	0.0
*Meta-hybrids*										
M06	19.5	−47.9	22.7	−22.7	43.5	7.0	34.9	0.0	48.4	0.0
M06-2X	17.8	−48.8	20.7	−29.7	44.5	7.9	36.6	0.0	49.0	0.0
M06-HF	13.8	−48.4	17.1	−40.7	42.9	9.5	37.7	0.0	50.7	0.0
TPSSH	16.7	−43.3	17.8	−22.6	41.2	6.8	32.4	0.0	45.2	0.0
*Double-hybrids*										
B2K-PLYP	16.0	−49.2	17.6	−25.8	44.1	8.5	37.4	0.0	49.2	0.0
B2T-PLYP	17.7	−45.6	18.5	−23.2	44.1	9.5	36.7	0.0	49.2	0.0
B2-PLYP	17.9	−43.9	18.4	−21.2	43.7	10.0	35.9	0.0	48.5	0.0
LS1-TPSS	10.6	−56.7	14.4	−30.3	41.9	4.8	36.3	0.0	45.6	0.0
mPW2K-PLYP	15.7	−49.4	17.2	−26.1	44.1	8.7	37.5	0.0	49.1	0.0
mPW2-PLYP	18.4	−44.8	18.8	−23.1	44.6	10.2	36.9	0.0	49.6	0.0
PBE0-DH	15.9	−56.8	18.7	−33.8	42.8	4.0	34.8	0.0	45.9	0.0
revDSD-BLYP	15.2	−49.3	16.8	−25.8	44.3	9.0	38.3	0.0	49.7	0.0
revDSD-PBE	14.5	−52.1	16.8	−27.4	43.1	6.7	37.3	0.0	47.8	0.0
revDSD-PBEP86	16.8	−49.4	18.6	−25.2	43.9	7.6	38.4	0.0	50.0	0.0
*Range-separated hybrids*										
CAM-B3LYP	23.7	−45.4	22.1	−27.8	46.0	9.2	37.3	0.0	51.7	0.0
CAMY-B3LYP	21.7	−44.5	20.7	−26.3	45.3	9.9	36.1	0.0	49.7	0.0
ωB97	23.7	−56.1	22.6	−35.2	46.5	3.2	40.4	0.0	53.0	0.0
ωB97X	23.0	−52.3	22.2	−32.3	46.0	5.4	38.8	0.0	51.7	0.0
ωB97X-D	23.1	−47.7	23.0	−27.9	44.7	6.3	36.4	0.0	50.2	0.0
*Dispersion-corrected*										
BP86-D3(BJ)	8.4	−47.2	11.2	−24.4	39.3	8.4	27.6	0.0	34.7	0.0
BLYP-D3(BJ)	13.2	−36.5	14.1	−15.9	42.2	13.8	31.0	0.0	41.0	0.0
PBE-D3(BJ)	8.4	−49.5	11.1	−26.0	38.7	6.7	27.1	0.0	33.5	0.0
OLYP-D3(BJ)	6.8	−50.5	11.9	−25.8	38.9	6.8	27.4	0.0	36.1	0.0
OPBE-D3(BJ)	1.7	−62.6	9.9	−34.1	35.4	−0.1	24.0	0.0	29.7	0.0
B3LYP-D3(BJ)	16.2	−42.8	17.3	−22.9	44.4	11.8	34.4	0.0	46.0	0.0
PBE0-D3(BJ)	12.6	−55.5	15.7	−33.1	41.7	4.9	31.8	0.0	40.9	0.0
M06-2X-D3	17.6	−48.9	20.6	−29.7	44.5	7.9	36.6	0.0	48.9	0.0

**Table 4:** Mean error (ME), mean absolute error (MAE), maximum unsigned error (MUE) and standard deviation (SD) of reaction barriers and energies (kcal mol^−1^) for the various pericyclic reactions, computed using various density functional approximations at XC/QZ4P//CCSD(T)/cc-pVTZ compared to FPA methods targeting CCSDT(Q)/CBS//CCSD(T)/cc-pVTZ

XC	Δ*E*^‡^				Δ*E*_rxn_				Total			
	ME	MAE	MUE	SD	ME	MAE	MUE	SD	ME	MAE	MUE	SD
*LDA*												
VWN	−17.3	17.3	28.3	6.9	−12.4	12.4	17.8	4.8	−15.5	15.5	28.3	6.7
*GGAs*												
BP86	−6.5	6.5	12.1	3.5	4.5	4.7	8.5	3.4	−2.3	5.8	12.1	3.5
BLYP	−1.5	2.8	5.1	1.8	13.7	13.7	18.3	6.0	4.2	6.9	18.3	6.6
BEE	−6.0	6.0	11.8	3.9	3.9	5.4	9.3	2.9	−2.3	5.8	11.8	3.6
PW91	−8.1	8.1	14.5	3.7	1.8	2.5	5.1	1.8	−4.4	6.0	14.5	4.1
PBE	−8.0	8.0	14.4	3.7	1.6	2.9	5.5	1.9	−4.4	6.0	14.4	4.0
PBEsol	−13.1	13.1	21.9	5.1	−6.4	6.4	9.4	2.1	−10.6	10.6	21.9	5.3
RPBE	−3.8	4.3	8.5	3.1	7.6	7.8	13.6	5.5	0.5	5.6	13.6	4.5
revPBE	−4.1	4.5	9.0	3.2	7.0	7.5	13.0	5.1	0.1	5.6	13.0	4.3
mPBE	−6.8	6.8	12.8	3.6	3.3	4.2	7.7	2.6	−3.0	5.8	12.8	3.5
mPW	−5.9	5.9	11.3	3.4	5.0	5.2	9.5	3.8	−1.8	5.6	11.3	3.6
HTBS	−8.2	8.2	14.7	4.2	0.6	3.2	5.2	2.0	−4.9	6.3	14.7	4.3
OLYP	−0.8	4.9	6.2	1.1	8.6	9.6	15.7	6.0	2.7	6.6	15.7	4.4
OPBE	−5.1	6.6	11.4	3.9	−0.9	5.5	8.6	3.2	−3.6	6.2	11.4	3.7
XLYP	−1.0	2.5	4.4	1.5	14.7	14.7	19.2	6.1	4.8	7.1	19.2	7.0
*Meta-GGAs*												
M06-L	−1.4	2.4	4.3	1.3	3.6	5.3	12.0	4.7	0.5	3.5	12.0	3.4
MVS	−6.0	6.1	13.6	5.1	−1.8	4.5	6.9	1.8	−4.4	5.5	13.6	4.2
TPSS	−4.6	4.6	7.2	1.8	5.3	5.8	9.4	3.7	−0.9	5.0	9.4	2.7
revTPSS	−4.6	4.6	5.4	0.9	3.0	4.6	6.2	1.6	−1.7	4.6	6.2	1.2
*Hybrids*												
B3LYP	1.2	2.1	3.7	1.2	7.9	7.9	10.3	3.3	3.7	4.3	10.3	3.5
B3LYP*	−0.7	2.0	3.5	1.3	7.4	7.4	9.9	3.1	2.3	4.0	9.9	3.4
B1LYP	2.7	2.8	5.0	1.8	7.9	7.9	10.4	3.1	4.6	4.7	10.4	3.4
B1PW91	−1.3	2.4	4.1	1.5	−0.5	2.0	2.8	0.7	−1.0	2.3	4.1	1.3
BHandH	−4.2	4.5	7.8	2.7	−14.5	14.5	19.5	6.8	−8.1	8.3	19.5	6.7
BHandHLYP	6.8	6.8	8.3	1.6	2.0	2.0	3.1	0.8	5.0	5.0	8.3	2.7
KMLYP	1.2	1.4	3.1	1.1	−9.9	9.9	13.5	4.6	−3.0	4.6	13.5	5.1
O3LYP	−17.9	17.9	28.7	7.7	−20.4	20.4	28.5	8.8	−18.9	18.9	28.7	8.2
OPBE0	−1.4	4.1	5.9	1.8	−5.5	5.5	8.8	3.3	−3.0	4.7	8.8	2.6
PBE0	−3.4	3.4	7.3	2.5	−3.6	3.6	5.2	1.3	−3.5	3.5	7.3	2.1
mPW1PW	−1.7	2.4	4.6	1.5	−0.8	1.7	2.3	0.4	−1.3	2.1	4.6	1.2
mPW1K	1.3	1.4	4.4	1.6	−5.0	5.0	6.6	1.2	−1.1	2.7	6.6	2.3
S12H	−2.4	2.8	6.2	2.0	−3.3	3.3	5.8	2.3	−2.8	3.0	6.2	2.1
X3LYP	0.8	1.9	3.0	0.7	6.6	6.6	8.5	2.5	3.0	3.7	8.5	2.8
*Meta-hybrids*												
M06	0.1	1.7	4.4	1.6	1.5	2.6	6.2	2.6	0.6	2.0	6.2	2.1
M06-2X	0.1	1.3	2.4	0.7	−0.8	0.8	1.2	0.2	−0.3	1.1	2.4	0.6
M06-HF	−1.2	1.8	5.8	2.0	−3.8	4.5	11.8	5.2	−2.2	2.8	11.8	3.8
TPSSH	−3.0	3.0	5.0	1.6	3.0	4.1	6.3	1.9	−0.8	3.4	6.3	1.8
*Double-hybrids*												
B2K-PLYP	−0.8	1.3	3.6	1.1	0.5	1.6	3.1	1.2	−0.3	1.4	3.6	1.2
B2T-PLYP	−0.4	0.8	1.9	0.6	2.9	2.9	5.7	2.0	0.8	1.6	5.7	1.7
B2-PLYP	−0.8	0.9	1.7	0.7	4.3	4.3	7.7	2.5	1.1	2.2	7.7	2.3
LS1-TPSS	−3.9	3.9	9.0	2.9	−4.7	4.7	9.1	3.2	−4.2	4.2	9.1	3.1
mPW2K-PLYP	−0.9	1.5	3.9	1.2	0.4	1.6	2.8	1.0	−0.4	1.5	3.9	1.1
mPW2-PLYP	0.0	0.7	1.2	0.4	3.5	3.5	5.8	1.7	1.3	1.7	5.8	1.7
PBE0-DH	−2.0	2.2	4.3	1.6	−6.2	6.2	9.2	2.1	−3.6	3.7	9.2	2.6
revDSD-BLYP	−0.8	1.8	4.4	1.4	0.7	1.8	3.1	1.0	−0.3	1.8	4.4	1.2
revDSD-PBE	−1.8	2.0	5.1	1.7	−1.6	2.6	4.5	1.3	−1.7	2.2	5.1	1.6
revDSD-PBEP86	−0.1	1.1	2.8	1.0	0.4	2.1	3.7	1.2	0.1	1.5	3.7	1.2
*Range-separated hybrids*												
CAM-B3LYP	2.5	2.5	4.1	1.4	1.4	1.4	2.3	0.6	2.1	2.1	4.1	1.3
CAMY-B3LYP	1.0	1.5	2.4	0.8	2.4	2.4	3.2	0.7	1.5	1.8	3.2	0.9
ωB97	3.6	3.6	4.3	0.6	−6.7	6.7	8.5	1.3	−0.3	4.7	8.5	1.8
ωB97X	2.7	2.7	3.9	0.9	−3.7	3.7	4.7	0.7	0.3	3.1	4.7	1.0
ωB97X-D	1.8	2.0	4.7	1.8	−0.4	1.1	2.2	0.9	1.0	1.6	4.7	1.6
*Dispersion-corrected*												
BP86-D3(BJ)	−9.4	9.4	15.5	3.8	1.6	1.7	4.5	2.0	−5.3	6.5	15.5	5.0
BLYP-D3(BJ)	−5.4	5.4	9.2	2.6	9.8	9.8	13.0	3.2	0.3	7.0	13.0	3.6
PBE-D3(BJ)	−9.9	9.9	16.7	4.1	−0.2	2.2	2.9	0.5	−6.3	7.0	16.7	4.9
OLYP-D3(BJ)	−9.4	9.4	14.1	3.6	−0.5	2.5	3.1	0.6	−6.1	6.8	14.1	4.4
OPBE-D3(BJ)	−13.5	13.5	20.5	5.0	−9.6	9.6	15.0	4.0	−12.0	12.0	20.5	5.0
B3LYP-D3(BJ)	−2.0	2.4	4.2	1.3	4.7	4.7	6.0	1.1	0.5	3.3	6.0	1.7
PBE0-D3(BJ)	−5.1	5.1	9.3	2.8	−5.2	5.2	7.9	1.9	−5.2	5.2	9.3	2.5
M06-2X-D3	0.0	1.4	2.3	0.7	−0.9	0.9	1.3	0.3	−0.3	1.2	2.3	0.7

Table 4 and Table S11 (ESI†) show that the LDA functional VWN yields too low reaction barriers with a MAE of 17.3 kcal mol^−1^ and in the worst case of the double group transfer an MUE of 28.3 kcal mol^−1^, which is likely due to the well-known overbinding of this functional. Next to that, VWN also leads to extremely exothermic reactions with a MAE and MUE of 12.4 kcal mol^−1^ and 17.8 kcal mol^−1^, respectively. Additionally, the GGA functional PBEsol and hybrid functionals O3LYP and BHandH perform nearly as poorly as LDA, having a total MAE of 10.6, 18.9, and 8.3 kcal mol^−1^, respectively.

The best overall agreement with our *ab initio* benchmark reaction barriers and reaction energies is obtained by representatives from the meta-hybrid and double-hybrid family, with total MAEs of 1.1 (M06-2X), 1.4 (B2K-PLYP), and 1.5 kcal mol^−1^ (mPW2K-PLYP and revDSD-PBEP86). The best performing density functional approximation, M06-2X, also has the lowest MUE and SD of only 2.4 and 0.6 kcal mol^−1^, respectively. The three best performing double-hybrid functionals (B2K-PLYP, mPW2K-PLYP, and revDSD-PBEP86), on the other hand, have a slightly higher MUE and SD, with a MUE around 4 kcal mol^−1^ and a SD around 1 kcal mol^−1^. Nevertheless, the error distribution of these functionals is not uniform as they under- and overestimate some of the reaction barriers and energies depending on the reaction, *i.e.*, the ME is, due to error cancelation, unequal to the MAE. For instance, mPW2K-PLYP *underestimates*, amongst others, the reaction barrier of the Diels-Alder reaction by 3.9 kcal mol^−1^, but, in contrast, *overestimates*, *e.g.*, the reaction barrier of the electrocyclic rearrangement by 0.7 kcal mol^−1^. We want to highlight that, despite a somewhat higher MAE of 5.8 kcal mol^−1^, the still popular density functional approximation BP86^4*a*,*b*,7*d*^ is able to describe the trends in reaction barriers and reaction energies qualitatively accurately.

Karton and Goerigk proposed PWPB95-D3(BJ) as best performing density functional approximation, with a root mean square deviation in reaction barrier heights of 1.0 kcal mol^−1^ with respect to their W*n*F12 reference data for four classes of pericyclic reactions (26 reactions in total).^5*c*^ The difference between our results and those of Karton and Goerigk could be ascribed to several factors. (1) The density functional approximations in this work are compared to more accurate reference data computed on more accurate geometries (*vide supra*). (2) The density functional approximations are, in contrast to the work of Karton and Goerigk, not only assessed based on their performance of accurately describing the reaction barrier, but also the reaction energies and thus the reverse reaction. (3) Karton and Goerigk exclusively assess the performance of dispersion-corrected density functional approximations, which, as we show later, artificially lower the reaction barrier for pericyclic reaction that involve small reactants with minimal dispersive intermolecular interactions. When solely concentrating on the reaction barriers, as done by Karton and Goerigk, we find three other density functional approximations that outperform, even with respect to our more accurate reference data, the PWPB95-D3(BJ) functional proposed by Karton and Goerigk (*vide infra*). This is, however, not the reason why PWPB95-D3(BJ) is not included in this DFT performance study. The reason why PWPB95-D3(BJ) is not included in this DFT performance study is that this XC functional is not available in the ADF software package.

Evaluating the performance of the functionals in describing the reaction barrier and reaction energy separately reveals that the top three best functionals for accurately describing the reaction barriers are the double-hybrid functionals mPW2-PLYP, B2T-PLYP, and B2-PLYP with a MAE, for all reactions together, of only 0.7, 0.8, and 0.9 kcal mol^−1^, respectively, and a MUE of no more than 2.0 kcal mol^−1^. The best functionals for evaluating the reaction energies are, on the other hand, the meta-hybrid functional M06-2X, with a MAE of only 0.8 kcal mol^−1^ and a MUE of 1.2 kcal mol^−1^, followed by the range-separated hybrid functionals ωB97X-D that has a MAE of 1.1 kcal mol^−1^ and a MUE of around 2.2 kcal mol^−1^ and CAM-B3LYP that has a MAE of 1.4 kcal mol^−1^ and a MUE of around 2.3 kcal mol^−1^. Although the double-hybrid functionals are able to accurately describe the reaction barriers and energies, a disadvantage of these functionals, up to this point, is that, in the context of an STO-based approach, they are computationally less efficient for geometry optimization or transition state search calculations. However, an effective workaround is to perform single-point energy calculations on stationary point geometries calculated using a more computationally more efficient functional (*vide infra*).

Next, we examine how the various DFT approximations perform for each individual one of the five pericyclic reactions (Table S11, ESI†). Interestingly, we find that, despite M06-2X, B2K-PLYP, mPW2K-PLYP, and revDSD-PBEP86 are able to accurately describe pericyclic reactions in general, they are not per definition the best functionals for describing the individual potential energy surfaces of the pericyclic reactions studied in this work. The most accurate energies for the Diels-Alder reaction are obtained using the M06, B1PW91, and OPBE functionals, with an error of less than 1.0 kcal mol^−1^ with respect to the CCSDT(Q)/CBS reference energies. The 1,3-dipolar cycloaddition, on the other hand, is described the best using the functionals S12H, PBE0, and revDSD-PBE, which have an error between 1.0–2.0 kcal mol^−1^. The DFT approximations B2K-PLYP, mPW2K-PLYP, and revDSD-PBEP86 are, with their error of less than 1.1 kcal mol^−1^, the best for describing the electrocyclic rearrangement. The reaction barrier of the sigmatropic rearrangement can be approximated within an error of only a few tenths of a kcal mol^−1^ with B2T-PLYP, mPW2-PLYP, and M06-2X. At last, mPW1K, ωB97X-D, and KMLYP are able to approach the reference energies of the double group transfer with 0.0–0.1 kcal mol^−1^.

Adding Grimme's empirical D3 dispersion correction, in combination with the Becke-Johnson damping, denoted as D3(BJ), results in a significant worsening of the performance of the GGA, hybrid, and meta-hybrid functionals with a maximum increase in total MAE of 6 kcal mol^−1^. The D3(BJ) dispersion correction leads to an additional stabilization of the stationary points, which increases the deviation from the CCSDT(Q)/CBS reference energies when a functional is already underestimating the stationary point energy. For instance, BP86 underestimates the reaction barrier of the pericyclic reaction with errors ranging from −2.7 kcal mol^−1^ for the 1,3-dipolar cycloaddition reaction to −12.1 kcal mol^−1^ for the double group transfer and hence has a MAE, for the reaction barriers, of 6.5 kcal mol^−1^. Adding the D3(BJ) dispersion correction, *i.e.*, BP86-D3(BJ), aggravates the underestimation of the reaction barriers by this functional, increasing the errors to −7.1 kcal mol^−1^ for the 1,3-dipolar cycloaddition reaction and −15.5 kcal mol^−1^ for the double group transfer and, therefore, increases the MAE, for the reaction barriers, to 9.4 kcal mol^−1^. This shows that including dispersion corrections to the evaluation of the reaction barriers of pericyclic reactions involving small reactants that exhibit minimal dispersive intermolecular interactions, such as steric attraction or hydrogen bonds, leads to a worsening (*not improvement*) of the accuracy of the computed energies. The D3(BJ) dispersion correction does, however, have a positive effect on the reaction energies. As these functionals generally underestimate the stability of the adducts, they can be partly compensated by including an additional stabilizing dispersion correction and hence reduces the MAE of the reaction energies. Notably, adding Grimme's D3 dispersion correction to the overall best performing functional M06-2X, *i.e.*, M06-2X-D3, leads to a slight worsening of the accuracy of this functional by increasing the MAE to 1.2 kcal mol^−1^.

Finally, we have additionally evaluated the performance of the 52 functionals and eight dispersion-corrected functionals using (i) a TZ2P basis set on CCSD(T)/cc-pVTZ geometries and (ii) a QZ4P and TZ2P basis set on DFT geometries optimized at BP86/DZ, BP86/DZP, BP86/TZ2P, BP86/QZ4P, BP86-D3(BJ)/QZ4P (*vide supra*). We find that, for the CCSD(T)/cc-pVTZ geometries, there is almost no difference between energies computed with the TZ2P and QZ4P basis set, since the variation in MAE from TZ2P is QZ4P is only 0.2 kcal mol^−1^. Hence, the energies are converged with a TZ2P basis set (Tables S13–S15, ESI†). Notably, the energies evaluated using the density functional approximations in this work do not always have the best agreement with the CCSDT(Q)/CBS benchmark values when employing the best, *i.e.*, largest, basis set. This indicates an increased error cancelation for these functionals with a smaller basis set.

Evaluating the performance of the density functional approximation using DFT geometries optimized at BP86/BS, where BS = DZ, DZP, TZ2P, QZ4P, and BP86-D3(BJ)/QZ4P give results with similar accuracy as the evaluation based on the *ab initio* reference geometries at CCSD(T)/cc-pVTZ (Tables S11–S45, ESI†). The differences in total MAE between the computations performed on the DFT and *ab initio* geometries are within a few tenths of a kcal mol^−1^. Even the earlier established poor BP86/DZ geometries give a total MAE that differs only a few tenths of a kcal mol^−1^ with respect to the very accurate CCSD(T)/cc-pVTZ geometries. In analogy with the evaluation of the functionals on the *ab initio* geometries, we find that for the DFT optimized geometries, the best overall performance is again obtained by members of the meta-hybrid and double-hybrid family, namely, M06-2X, B2K-PLYP, mPW2K-PLYP, and revDSD-PBEP86, having a total MAE of no more than 1.5 kcal mol^−1^ (maximum total MAE for BP86/DZ geometries is 2.3 kcal mol^−1^) and an MUE of a few kcal mol^−1^ with respect to the CCSDT(Q)/CBS reference energy values. These results unequivocally show that one can obtain very accurate pericyclic reaction barriers and reaction energies by optimizing the geometries of the stationary points using an affordable DFT approach, such as BP86/DZP, followed by a refinement of the energy by performing single-point calculations using the more accurate, but also more expensive, hybrid functional M06-2X or a double-hybrid functionals B2K-PLYP, mPW2K-PLYP, and revDSD-PBEP86.

## 4. Conclusions

We have performed a comprehensive *ab initio* benchmark study yielding reference data of unprecedented accuracy for various pericyclic reactions, including the Diels-Alder reaction, 1,3-dipolar cycloaddition, electrocyclic rearrangement, sigmatropic rearrangement, and double group transfer. Our reference data for reaction barriers and reaction energies emerge from a hierarchical series of *ab initio* quantum mechanical methods, up to CCSDT(Q), in combination with a series of correlation-consistent Gaussian-type basis sets, up to the complete basis set (CBS) limit, along which they converge within a few tenths of a kcal mol^−1^.

We have analyzed the performance of 60 density functional approximations for describing the above-mentioned pericyclic reactions against our best *ab initio* benchmark data at CCSDT(Q)/CBS and find that the meta-hybrid functional M06-2X is the best performing density functional approximation, with a mean absolute error (MAE) of only 1.1 kcal mol^−1^ and a maximum unsigned error (MUE) of 2.4 kcal mol^−1^. The mean error (ME) and standard deviation (SD) of M06-2X are also small, only −0.3 and 0.6 kcal mol^−1^, respectively. This meta-hybrid functional is closely followed by representatives from the double-hybrid family, namely, B2K-PLYP (MAE = 1.4 kcal mol^−1^), mPW2K-PLYP, and revDSD-PBEP86 (MAE = 1.5 kcal mol^−1^). The BP86 functional, which has frequently been employed to study pericyclic reactions, has a higher MAE of 5.8 kcal mol^−1^. Inspection, however, shows that BP86 trends in barriers and reaction energies are qualitatively accurate. Furthermore, we found that, for practical application, the energies computed using most of the 60 DFT functionals reach their convergence with the TZ2P basis set. In most cases, the variation in MAE from TZ2P to QZ4P is 0.2 kcal mol^−1^ or less (*e.g.*, 0.1 kcal mol^−1^ for M06-2X and BP86).

Finally, we find that an efficient and still accurate protocol for exploring pericyclic reactions consists of computing BP86/DZP geometries followed by single-point corrections, using the TZ2P basis set, with the more sophisticated density functional approximations M06-2X, B2K-PLYP, mPW2K-PLYP, or revDSD-PBEP86.

## Conflicts of interest

There are no conflicts to declare.

## Supplementary Material

CP-024-D2CP02234F-s001
