# Correction: Pericyclic reaction benchmarks: hierarchical computations targeting CCSDT(Q)/CBS and analysis of DFT performance

**DOI:** 10.1039/d4cp90047b

**Published:** 2024-03-04

**Authors:** Pascal Vermeeren, Marco Dalla Tiezza, Mark E. Wolf, Mitchell E. Lahm, Wesley D. Allen, Henry F. Schaefer, Trevor A. Hamlin, F. Matthias Bickelhaupt

**Affiliations:** a Department of Theoretical Chemistry, Amsterdam Institute of Molecular and Life Sciences (AIMMS), Amsterdam Center for Multiscale Modeling (ACMM), Vrije Universiteit Amsterdam De Boelelaan 1083 1081 HV Amsterdam The Netherlands t.a.hamlin@vu.nl f.m.bickelhaupt@vu.nl; b Center for Computational Quantum Chemistry, University of Georgia Athens GA 30602 USA ccq@uga.edu; c Allen Heritage Foundation Dickson TN 37055 USA; d Institute for Molecules and Materials (IMM), Radboud University Heyendaalseweg 135 6525 AJ Nijmegen The Netherlands

## Abstract

Correction for ‘Pericyclic reaction benchmarks: hierarchical computations targeting CCSDT(Q)/CBS and analysis of DFT performance’ by Pascal Vermeeren *et al.*, *Phys. Chem. Chem. Phys.*, 2022, **24**, 18028–18042, https://doi.org/10.1039/D2CP02234F.

In our paper the performance of 60 density functionals were assessed, including the range-separated hybrid functional ωB97X-D. We have identified a technical issue in the implementation of this functional, that is, the dispersion correction for ωB97X-D in the ADF software package. Therefore, the data for ωB97X-D in both Table 3 and 4 should be disregarded.

The Royal Society of Chemistry apologises for these errors and any consequent inconvenience to authors and readers.

## Supplementary Material

