# Supplementary material for: Pericyclic reaction benchmarks: hierarchical computations targeting CCSDT(Q)/CBS and analysis of DFT performance
Source: Phys Chem Chem Phys. 2022 Jul 21;24(30):18028–42. doi: 10.1039/d2cp02234f (PMC9348522; doi:10.1039/d2cp02234f)
Supplement: CP-024-D2CP02234F-s001 [file CP-024-D2CP02234F-s001.pdf]

Electronic Supplementary Material (ESI) for Physical Chemistry Chemical Physics.
This journal is © the Owner Societies 2022

Supporting Information for

Pericyclic Reactions: Hierarchical Benchmark up to CCSDT(Q)/CBS and DFT Performance Study

Pascal Vermeeren,[+ a] Marco Dalla Tiezza,[+ a] Mark E. Wolf,[+ b] Mitchell E. Lahm,[+ b] Wesley D. Allen,*[b,c] Henry F. Schaefer III,*[b] Trevor A. Hamlin,*[a] F. Matthias Bickelhaupt*[a,d]

[a] Department of Theoretical Chemistry, Amsterdam Institute of Molecular and Life Sciences (AIMMS), Amsterdam Center for Multiscale Modeling (ACMM), Vrije Universiteit Amsterdam, De Boelelaan 1083, 1081 HV Amsterdam, The Netherlands. E-mail: t.a.hamlin@vu.nl; f.m.bickelhaupt@vu.nl

[b] Center for Computational Quantum Chemistry, University of Georgia, Athens, GA 30602, United States. E-mail: ccq@uga.edu

[c] Allen Heritage Foundation, Dickson, TN 37055, United States.

[d] Institute for Molecules and Materials (IMM), Radboud University, Heyendaalseweg 135, 6525 AJ Nijmegen, The Netherlands.

+ These authors contributed equally to this work.

Contents

Table S1. Harmonic vibrational frequencies for all structures at CCSD(T)/cc-pVTZ in cm^{-1}.

Table S2. Focal point analysis in kcal mol^{-1} with values computed atop CCSD(T)/cc-pVTZ geometries.

Table S3. Cartesian coordinates (in Å), energies (in Hartree), and number of imaginary frequencies of all stationary points, computed at CCSD(T)/cc-pVTZ.

Table S4. Cartesian coordinates (in Å), energies (in kcal mol^{-1}), and number of imaginary frequencies of all stationary points, computed at BP86/DZ.

Table S5. Cartesian coordinates (in Å), energies (in kcal mol^{-1}), and number of imaginary frequencies of all stationary points, computed at BP86/DZP.

Table S6. Cartesian coordinates (in Å), energies (in kcal mol^{-1}), and number of imaginary frequencies of all stationary points, computed at BP86/TZ2P.

Table S7. Cartesian coordinates (in Å), energies (in kcal mol^{-1}), and number of imaginary frequencies of all stationary points, computed at BP86/QZ4P.

Table S8. Cartesian coordinates (in Å), energies (in kcal mol^{-1}), and number of imaginary frequencies of all stationary points, computed at BP86-D3(BJ)/QZ4P.

Table S9. Global-minimum Cartesian root-mean-square deviation (RMSD) values (in Å) of the fully optimized geometries at various DFT levels of theory with respect to the CCSD(T)/cc-pVTZ benchmarks.

Table S10. Average percentage errors of all chemically meaningful geometric parameters of the fully optimized DFT geometries with respect to the CCSD(T)/cc-pVTZ benchmarks.

Table S11. Errors in reaction barriers and reaction energies (kcal mol^{-1}) for the various pericyclic reactions, computed with XC/QZ4P//CCSD(T)/cc-pVTZ, compared to CCSDT(Q)/CBS//CCSD(T)/cc-pVTZ.

Table S12. Reaction barriers and reaction energies (kcal mol^{-1}) for the various pericyclic reactions, computed at DFT level using Method/BS, where Method = BP86, BP86-D3(BJ) and BS = DZ, DZP, TZ2P, QZ4P.

Table S13. Reaction barriers and reaction energies (kcal mol^{-1}) for the various pericyclic reactions, computed with XC/TZ2P//CCSD(T)/cc-pVTZ.

Table S14. Errors in reaction barriers and reaction energies (kcal mol^{-1}) for the various pericyclic reactions, computed with XC/TZ2P//CCSD(T)/cc-pVTZ, compared to CCSDT(Q)/CBS//CCSD(T)/cc-pVTZ.

Table S15. Mean error (ME), mean absolute error (MAE), maximum unsigned error (MUE) and standard deviation (SD) of reaction barriers and energies (kcal mol^{-1}) for the various pericyclic reactions, computed with XC/TZ2P//CCSD(T)/cc-pVTZ compared to CCSDT(Q)/CBS//CCSD(T)/cc-pVTZ.

Table S16. Reaction barriers and reaction energies (kcal mol^{-1}) for the various pericyclic reactions, computed with XC/TZ2P//BP86/DZ.

Table S17. Errors in reaction barriers and reaction energies (kcal mol^{-1}) for the various pericyclic reactions, computed with XC/TZ2P//BP86/DZ, compared to CCSDT(Q)/CBS//CCSD(T)/cc-pVTZ.

Table S18. Mean error (ME), mean absolute error (MAE), maximum unsigned error (MUE) and standard deviation (SD) of reaction barriers and energies (kcal mol^{-1}) for the various pericyclic reactions, computed with XC/TZ2P//BP86/DZ compared to CCSDT(Q)/CBS//CCSD(T)/cc-pVTZ.

Table S19. Reaction barriers and reaction energies (kcal mol^{-1}) for the various pericyclic reactions, computed with XC/QZ4P//BP86/DZ.

Table S20. Errors in reaction barriers and reaction energies (kcal mol^{-1}) for the various pericyclic reactions, computed with XC/QZ4P//BP86/DZ, compared to CCSDT(Q)/CBS//CCSD(T)/cc-pVTZ.

Table S21. Mean error (ME), mean absolute error (MAE), maximum unsigned error (MUE) and standard deviation (SD) of reaction barriers and energies (kcal mol^{-1}) for the various pericyclic reactions, computed with XC/QZ4P//BP86/DZ compared to CCSDT(Q)/CBS//CCSD(T)/cc-pVTZ.

Table S22. Reaction barriers and reaction energies (kcal mol^{-1}) for the various pericyclic reactions, computed with XC/TZ2P//BP86/DZP.

Table S23. Errors in reaction barriers and reaction energies (kcal mol^{-1}) for the various pericyclic reactions, computed with XC/TZ2P//BP86/DZP, compared to CCSDT(Q)/CBS//CCSD(T)/cc-pVTZ.

Table S24. Mean error (ME), mean absolute error (MAE), maximum unsigned error (MUE) and standard deviation (SD) of reaction barriers and energies (kcal mol^{-1}) for the various pericyclic reactions, computed with XC/TZ2P//BP86/DZP compared to CCSDT(Q)/CBS//CCSD(T)/cc-pVTZ.

Table S25. Reaction barriers and reaction energies (kcal mol^{-1}) for the various pericyclic reactions, computed with XC/QZ4P//BP86/DZP.

Table S26. Errors in reaction barriers and reaction energies (kcal mol^{-1}) for the various pericyclic reactions, computed with XC/QZ4P//BP86/DZP, compared to CCSDT(Q)/CBS//CCSD(T)/cc-pVTZ.

Table S27. Mean error (ME), mean absolute error (MAE), maximum unsigned error (MUE) and standard deviation (SD) of reaction barriers and energies (kcal mol^{-1}) for the various pericyclic reactions, computed with XC/QZ4P//BP86/DZP compared to CCSDT(Q)/CBS//CCSD(T)/cc-pVTZ.

Table S28. Reaction barriers and reaction energies (kcal mol^{-1}) for the various pericyclic reactions, computed with XC/TZ2P//BP86/TZ2P.

Table S29. Errors in reaction barriers and reaction energies (kcal mol^{-1}) for the various pericyclic reactions, computed with XC/TZ2P//BP86/TZ2P, compared to CCSDT(Q)/CBS//CCSD(T)/cc-pVTZ.

Table S30. Mean error (ME), mean absolute error (MAE), maximum unsigned error (MUE) and standard deviation (SD) of reaction barriers and energies (kcal mol^{-1}) for the various pericyclic reactions, computed with XC/TZ2P//BP86/TZ2P compared to CCSDT(Q)/CBS//CCSD(T)/cc-pVTZ.

Table S31. Reaction barriers and reaction energies (kcal mol^{-1}) for the various pericyclic reactions, computed with XC/QZ4P//BP86/TZ2P.

Table S32. Errors in reaction barriers and reaction energies (kcal mol^{-1}) for the various pericyclic reactions, computed with XC/QZ4P//BP86/TZ2P, compared to CCSDT(Q)/CBS//CCSD(T)/cc-pVTZ.

Table S33. Mean error (ME), mean absolute error (MAE), maximum unsigned error (MUE) and standard deviation (SD) of reaction barriers and energies (kcal mol^{-1}) for the various pericyclic reactions, computed with XC/QZ4P//BP86/TZ2P compared to CCSDT(Q)/CBS//CCSD(T)/cc-pVTZ.

Table S34. Reaction barriers and reaction energies (kcal mol^{-1}) for the various pericyclic reactions, computed with XC/TZ2P//BP86/QZ4P.

Table S35. Errors in reaction barriers and reaction energies (kcal mol^{-1}) for the various pericyclic reactions, computed with XC/TZ2P//BP86/QZ4P, compared to CCSDT(Q)/CBS//CCSD(T)/cc-pVTZ.

Table S36. Mean error (ME), mean absolute error (MAE), maximum unsigned error (MUE) and standard deviation (SD) of reaction barriers and energies (kcal mol^{-1}) for the various pericyclic reactions, computed with XC/TZ2P//BP86/QZ4P compared to CCSDT(Q)/CBS//CCSD(T)/cc-pVTZ.

Table S37. Reaction barriers and reaction energies (kcal mol^{-1}) for the various pericyclic reactions, computed with XC/QZ4P//BP86/QZ4P.

Table S38. Errors in reaction barriers and reaction energies (kcal mol^{-1}) for the various pericyclic reactions, computed with XC/QZ4P//BP86/QZ4P, compared to CCSDT(Q)/CBS//CCSD(T)/cc-pVTZ.

Table S39. Mean error (ME), mean absolute error (MAE), maximum unsigned error (MUE) and standard deviation (SD) of reaction barriers and energies (kcal mol^{-1}) for the various pericyclic reactions, computed with XC/QZ4P//BP86/QZ4P compared to CCSDT(Q)/CBS//CCSD(T)/cc-pVTZ.

Table S40. Reaction barriers and reaction energies (kcal mol^{-1}) for the various pericyclic reactions, computed with XC/TZ2P//BP86-D3(BJ)/QZ4P.

Table S41. Errors in reaction barriers and reaction energies (kcal mol^{-1}) for the various pericyclic reactions, computed with XC/TZ2P//BP86-D3(BJ)/QZ4P, compared to CCSDT(Q)/CBS//CCSD(T)/cc-pVTZ.

Table S42. Mean error (ME), mean absolute error (MAE), maximum unsigned Error (MUE) and standard deviation (SD) of reaction barriers and energies (kcal mol^{-1}) for the various pericyclic reactions, computed with XC/TZ2P//BP86-D3(BJ)/QZ4P compared to CCSDT(Q)/CBS//CCSD(T)/cc-pVTZ.

Table S43. Reaction barriers and reaction energies (kcal mol^{-1}) for the various pericyclic reactions, computed with XC/QZ4P//BP86-D3(BJ)/QZ4P.

Table S44. Errors in reaction barriers and reaction energies (kcal mol^{-1}) for the various pericyclic reactions, computed with XC/TZ2P//BP86-D3(BJ)/QZ4P, compared to CCSDT(Q)/CBS//CCSD(T)/cc-pVTZ.

Table S45. Mean error (ME), mean absolute error (MAE), maximum unsigned error (MUE) and standard deviation (SD) of reaction barriers and energies (kcal mol^{-1}) for the various pericyclic reactions, computed with XC/QZ4P//BP86-D3(BJ)/QZ4P compared to CCSDT(Q)/CBS//CCSD(T)/cc-pVTZ.

Table S46. Multireference diagnostics for each of the pericyclic stationary points at the CCSD(T)/cc-pVTZ level of theory

Table S47. Standard Dunning basis raw electronic energies atop the CCSD(T)/cc-pVTZ structures for use in Focal Point analysis.

Table S48. Aug′ basis raw electronic energies atop the CCSD(T)/cc-pVTZ structures for use in Focal Point analysis.

Table S1. Harmonic vibrational frequencies for all structures at CCSD(T)/cc-pVTZ in cm^{-1}

**Diels-Alder**				**1,3-Dipolar cycloaddition**			**Electrocyclic rearrangement**	
B	E	TS-DA	P-DA	MA	TS-1,3-DC	P-1,3-DC	TS-ER	P-ER
164.8	823.0	618.9i	168.2	111.4	515.2i	167.4	760.0i	302.3
271.9	941.9	132.8	277.3	243.7	106.7	195.6	453.0	644.5
467.0	966.7	206.9	395.1	574.4	153.6	293.1	641.5	856.5
610.1	1047.0	282.9	445.6	669.7	169.6	346.6	674.3	865.0
742.4	1242.1	380.0	492.9	934.2	247.1	547.5	730.8	895.6
886.4	1369.0	399.7	649.5	1111.2	359.0	633.4	875.7	901.9
927.7	1479.0	482.6	727.5	1150.1	452.6	741.9	876.8	916.4
929.6	1671.7	575.4	820.8	1303.7	556.3	876.7	908.8	1002.3
998.6	3139.1	620.3	836.1	1446.6	652.2	886.4	939.0	1029.9
1017.2	3157.1	691.2	892.5	1503.2	663.7	940.9	1008.3	1099.9
1058.6	3219.3	770.1	916.0	1504.7	831.5	976.6	1029.6	1134.8
1098.9	3246.1	790.9	933.8	2179.9	871.9	1021.8	1121.0	1176.5
1301.5		841.9	984.7	3030.0	931.8	1093.8	1163.1	1219.2
1333.3		890.7	1019.8	3097.5	1001.1	1141.4	1232.2	1232.8
1437.3		937.6	1055.0	3162.7	1024.0	1173.6	1381.7	1323.5
1468.4		961.0	1084.4		1115.4	1191.2	1514.5	1470.9
1666.8		966.6	1097.2		1156.4	1265.1	1520.8	1493.9
1674.9		977.5	1155.1		1241.7	1282.2	1533.1	1604.9
3143.1		995.9	1160.1		1268.2	1310.7	3094.1	3052.2
3145.6		1001.3	1248.9		1300.6	1365.8	3094.4	3058.2
3152.1		1091.8	1270.3		1439.6	1445.5	3168.7	3097.0
3164.7		1097.4	1297.0		1472.9	1483.5	3189.6	3111.4
3237.2		1229.3	1350.1		1497.5	1493.8	3218.7	3181.9
3238.9		1242.7	1375.9		1504.3	1511.5	3219.5	3213.7
		1260.7	1378.5		1565.8	1520.4		
		1300.6	1386.1		1884.6	1553.5		
		1427.9	1422.8		3025.0	2982.0		
		1471.5	1479.0		3103.7	2994.3		
		1490.5	1486.6		3141.0	3057.1		
		1541.3	1492.4		3143.4	3099.9		
		1584.8	1502.9		3160.8	3127.1		
		1585.8	1700.8		3227.1	3145.2		
		3142.3	3011.3		3253.8	3151.1		
		3146.6	3011.3					
		3147.7	3022.0					
		3158.5	3026.8					
		3159.3	3057.5					
		3178.3	3057.9					
		3224.8	3078.0					
		3231.0	3080.2					
		3232.9	3144.1					
		3249.9	3169.6					

Sigmatropic rearrangement		Double Group Transfer	
TS-DA	P-DA	Et	TS-DGT
111.1	1690.1i	310.1	1780.0i
177.8	260.4	820.9	170.0
247.2	469.8	820.9	339.3
325.4	507.6	1013.9	494.5
419.0	549.0	1224.8	503.4
583.2	617.3	1224.8	512.1
703.3	738.1	1406.5	550.4
767.6	793.3	1427.5	705.4
886.0	808.4	1510.8	811.0
929.5	968.7	1510.8	836.7
961.6	975.9	1512.5	868.6
985.2	997.3	1512.5	967.7
1016.8	1001.5	3038.0	992.1
1061.2	1005.0	3039.6	1017.1
1078.3	1093.7	3096.9	1107.9
1117.0	1129.7	3096.9	1226.0
1264.4	1176.4	3120.1	1237.8
1322.4	1249.4	3120.1	1245.5
1397.9	1266.5		1380.0
1434.5	1376.1		1387.0
1465.8	1430.5		1414.4
1495.8	1483.5		1414.5
1498.3	1517.6		1417.6
1671.8	1528.4		1461.1
1690.0	1543.9		1503.2
3028.1	1590.7		1513.3
3086.6	3105.7		1525.7
3123.6	3105.9		1545.6
3136.4	3149.5		3111.0
3143.2	3154.0		3111.2
3153.9	3174.2		3123.6
3165.1	3180.3		3125.1
3238.4	3189.5		3185.3
			3185.7
			3205.2
			3207.1

Table S2. Focal point analysis in kcal mol^{-1} with values computed atop CCSD(T)/cc-pVTZ geometries.

Reaction Energies

Diels Alder Reaction (Trans-butadiene + Ethylene → Cycloadduct)

	ΔE_e(HF)	$+\delta$ [MP2]	$+\delta$[CCSD]	$+\delta$ [CCSD(T)]	$+\delta$ [CCSDT(Q)]	NET
cc-pVDZ	–39.40	–12.34	+5.19	+0.53	+0.37+0.15	–45.51
aug'-cc-pVDZ	–38.74	–12.11	+4.54	+0.56	[+0.52]	[–45.24]
aug'-cc–pVTZ	–35.33	–14.53	+4.38	+0.50	[+0.52]	[–44.46]
aug'-cc–pVQZ	–35.14	–14.54	+4.31	+0.47	[+0.52]	[–44.39]
aug'-cc–pV5Z	–35.08	–14.67	[+4.40]	[+0.46]	[+0.52]	[–44.37]
CBS LIMIT	[–35.06]	[–14.80]	[+4.50]	[+0.45]	[+0.52]	[–44.39]

Diels-Alder Reaction

	ΔE_e(HF)	$+\delta$ [MP2]	$+\delta$[CCSD]	$+\delta$ [CCSD(T)]	$+\delta$[CCSDT(Q)]	NET
cc–pVDZ	–42.71	–11.94	+5.26	+0.45	+0.35+0.14	–48.46
cc–pVTZ	–39.29	–13.41	+4.77	+0.42	[+0.49]	[–47.03]
cc–pVQZ	–38.79	–13.91	+4.50	+0.43	[+0.49]	[–47.28]
cc–pV5Z	–38.60	–14.06	[+4.41]	[+0.43]	[+0.49]	[–47.36]
CBS LIMIT	[–38.51]	[–14.23]	[+4.31]	[+0.43]	[+0.49]	[–47.50]

$\Delta E_{\text{final}} = \Delta E_e(\text{FPA}) + \Delta(\text{DBOC}) + \Delta(\text{rel}) + \Delta(\text{core}) + \Delta(\text{aug})$

$\Delta E_{\text{final}} = –47.50 + 0.02 + 0.13 – 0.21 + 0.19 =$ **–47.37 kcal mol^{-1}**

$\Delta E_0 = \Delta E_{\text{final}} + \Delta\text{ZPVE(harm)} = –47.37 + 6.75 = –40.62$ kcal mol^{-1}

1,3-dipolar Cycloaddition

	ΔE_e(HF)	$+\delta$ [MP2]	$+\delta$[CCSD]	$+\delta$ [CCSD(T)]	$+\delta$[CCSDT(Q)]	NET
cc–pVDZ	–30.34	+6.08	–5.92	+1.85	+0.11+0.65	–27.56
cc–pVTZ	–26.87	+2.79	–5.58	+1.60	[+0.76]	[–27.29]
cc–pVQZ	–26.71	+1.87	–5.64	+1.55	[+0.76]	[–28.17]
cc–pV5Z	–26.66	+1.40	[–5.52]	[+1.53]	[+0.76]	[–28.49]
CBS LIMIT	[–26.63]	[+0.90]	[–5.39]	[+1.51]	[+0.76]	[–28.85]

$\Delta E_{\text{final}} = \Delta E_e(\text{FPA}) + \Delta(\text{DBOC}) + \Delta(\text{rel}) + \Delta(\text{core}) + \Delta(\text{aug})$

$\Delta E_{\text{final}} = –28.85 – 0.02 + 0.17 + 0.04 + 0.13 =$ **–28.53 kcal mol^{-1}**

$\Delta E_0 = \Delta E_{\text{final}} + \Delta\text{ZPVE(harm)} = –28.53 + 5.28 = –23.25$ kcal mol^{-1}

Electrocyclic Rearrangement

	ΔE_e(HF)	$+\delta$ [MP2]	$+\delta$[CCSD]	$+\delta$ [CCSD(T)]	$+\delta$[CCSDT(Q)]	NET
cc–pVDZ	+10.78	–5.07	+2.15	+0.32	+0.19+0.03	+8.40
cc–pVTZ	+11.79	–5.40	+1.96	+0.27	[+0.22]	[+8.84]
cc–pVQZ	+11.95	–5.64	+1.83	+0.27	[+0.22]	[+8.63]
cc–pV5Z	+12.01	–5.77	[+1.82]	[+0.28]	[+0.22]	[+8.56]
CBS LIMIT	[+12.05]	[–5.91]	[+1.82]	[+0.28]	[+0.22]	[+8.46]
FUNCTION<br>X (fit points)	$a+be^{-cX}$<br>(3,4,5)	$a+bX^{-3}$<br>(4,5)	$a+bX^{-3}$<br>(3,4)	$a+bX^{-3}$<br>(3,4)	addition	

$\Delta E_{\text{final}} = \Delta E_e(\text{FPA}) + \Delta(\text{DBOC}) + \Delta(\text{rel}) + \Delta(\text{core}) + \Delta(\text{aug})$

$\Delta E_{\text{final}} = 8.46 + 0.01 + 0.05 + 0.03 – 0.02 =$ **+8.53 kcal mol^{-1}**

$\Delta E_0 = \Delta E_{\text{final}} + \Delta\text{ZPVE(harm)} = 8.53 + 1.07 = 9.60$ kcal mol^{-1}

Reaction Barriers

Diels Alder Reaction (Trans-butadiene + Ethylene → TS)

	ΔE_e(HF)	$+\delta$ [MP2]	$+\delta$[CCSD]	$+\delta$ [CCSD(T)]	$+\delta$ [CCSDT(Q)]	NET
cc-pVDZ	+46.92	–31.61	+13.52	–4.77	+0.48–0.53	+24.02
aug'-cc-pVDZ	+46.88	–34.99	+14.30	–5.29	[–0.05]	[+20.86]
aug'-cc–pVTZ	+48.59	–36.47	+15.06	–5.74	[–0.05]	[+21.40]
aug'-cc–pVQZ	+48.89	–36.25	+15.27	–5.86	[–0.05]	[+22.00]
aug'-cc–pV5Z	+48.95	–36.16	[+15.33]	[–5.91]	[–0.05]	[+22.17]
CBS LIMIT	[+48.97]	[–36.06]	[+15.39]	[–5.95]	[–0.05]	[+22.30]

Diels-Alder Reaction

	ΔE_e(HF)	$+\delta$ [MP2]	$+\delta$[CCSD]	$+\delta$ [CCSD(T)]	$+\delta$[CCSDT(Q)]	NET
cc–pVDZ	+43.61	–31.21	+13.60	–4.85	+0.46–0.53	+21.07
cc–pVTZ	+44.97	–34.64	+15.01	–5.64	[–0.07]	[+19.63]
cc–pVQZ	+45.32	–35.24	+15.28	–5.84	[–0.07]	[+19.44]
cc–pV5Z	+45.45	–35.44	[+15.35]	[–5.92]	[–0.07]	[+19.38]
CBS LIMIT	[+45.51]	[–35.65]	[+15.43]	[–5.99]	[–0.07]	[+19.24]

$\Delta E_{\text{final}} = \Delta E_e(\text{FPA}) + \Delta(\text{DBOC}) + \Delta(\text{rel}) + \Delta(\text{core}) + \Delta(\text{aug})$

$\Delta E_{\text{final}} = 19.24 + 0.04 + 0.03 + 0.11 + 0.09 =$ **19.51 kcal mol^{-1}**

$\Delta E_0 = \Delta E_{\text{final}} + \Delta\text{ZPVE(harm)} = 19.51 + 2.38 = 21.89$ kcal mol^{-1}

1,3-dipolar Cycloaddition

	ΔE_e(HF)	$+\delta$ [MP2]	$+\delta$[CCSD]	$+\delta$ [CCSD(T)]	$+\delta$[CCSDT(Q)]	NET
cc–pVDZ	+39.31	–27.73	+10.95	–4.64	+0.70–0.82	+17.77
cc–pVTZ	+42.99	–31.67	+12.86	–5.53	[–0.12]	[+18.53]
cc–pVQZ	+43.42	–32.43	+13.23	–5.79	[–0.12]	[+18.31]
cc–pV5Z	+43.58	–32.71	[+13.37]	[–5.89]	[–0.12]	[+18.24]
CBS LIMIT	[+43.65]	[–32.99]	[+13.51]	[–5.98]	[–0.12]	[+18.07]

$\Delta E_{\text{final}} = \Delta E_e(\text{FPA}) + \Delta(\text{DBOC}) + \Delta(\text{rel}) + \Delta(\text{core}) + \Delta(\text{aug})$

$\Delta E_{\text{final}} = 18.07 + 0.02 + 0.01 + 0.23 + 0.07 =$ **+18.40 kcal mol^{-1}**

$\Delta E_0 = \Delta E_{\text{final}} + \Delta\text{ZPVE(harm)} = 18.40 + 1.71 = 20.11$ kcal mol^{-1}

Electrocyclic Rearrangement

	ΔE_e(HF)	$+\delta$ [MP2]	$+\delta$[CCSD]	$+\delta$ [CCSD(T)]	$+\delta$[CCSDT(Q)]	NET
cc–pVDZ	+56.42	–15.23	+4.93	–2.64	+0.16–0.29	+43.35
cc–pVTZ	+56.64	–16.05	+5.72	–2.98	[–0.13]	[+43.20]
cc–pVQZ	+56.71	–16.19	+5.90	–3.06	[–0.13]	[+43.23]
cc–pV5Z	+56.73	–16.26	[+5.98]	[–3.09]	[–0.13]	[+43.22]
CBS LIMIT	[+56.73]	[–16.34]	[+6.06]	[–3.12]	[–0.13]	[+43.20]

$\Delta E_{\text{final}} = \Delta E_e(\text{FPA}) + \Delta(\text{DBOC}) + \Delta(\text{rel}) + \Delta(\text{core}) + \Delta(\text{aug})$

$\Delta E_{\text{final}} = 43.20 + 0.04 + 0.01 + 0.20 - 0.00 =$ **+43.45 kcal mol^{-1}**

$\Delta E_0 = \Delta E_{\text{final}} + \Delta\text{ZPVE(harm)} = 43.45 - 0.78 = 42.67$ kcal mol^{-1}

Sigmatropic Rearrangement [(*E*)-penta-1,3-diene→ TS]

	ΔE_e(HF)	$+\delta$ [MP2]	$+\delta$[CCSD]	$+\delta$ [CCSD(T)]	$+\delta$ [CCSDT(Q)]	NET
cc-pVDZ	+57.47	–23.73	+9.48	–3.53	+0.31–0.41	+39.59
aug'-cc-pVDZ	+57.43	–24.07	+9.77	–3.67	[–0.10]	[+39.35]
aug'-cc–pVTZ	+58.70	–25.17	+10.30	–4.12	[–0.10]	[+39.61]
aug'-cc–pVQZ	+58.77	–25.28	+10.41	–4.22	[–0.10]	[+39.58]
aug'-cc–pV5Z	+58.80	–25.31	[+10.43]	[–4.25]	[–0.10]	[+39.58]
CBS LIMIT	[+58.82]	[–25.33]	[+10.45]	[–4.29]	[–0.10]	[+39.55]

Sigmatropic Rearrangement

	ΔE_e(HF)	$+\delta$ [MP2]	$+\delta$[CCSD]	$+\delta$ [CCSD(T)]	$+\delta$[CCSDT(Q)]	NET
cc–pVDZ	+53.88	–22.92	+9.46	–3.57	+0.27–0.41	+36.71
cc–pVTZ	+54.95	–24.05	+10.20	–4.09	[–0.14]	[+36.88]
cc–pVQZ	+55.01	–24.29	+10.33	–4.21	[–0.14]	[+36.71]
cc–pV5Z	+55.01	–24.32	[+10.33]	[–4.25]	[–0.14]	[+36.63]
CBS LIMIT	[+55.00]	[–24.35]	[+10.32]	[–4.29]	[–0.14]	[+36.54]

$\Delta E_{\text{final}} = \Delta E_e(\text{FPA}) + \Delta(\text{DBOC}) + \Delta(\text{rel}) + \Delta(\text{core}) + \Delta(\text{aug})$

$\Delta E_{\text{final}} = 36.54 + 0.08 + 0.00 + 0.08 + 0.03 = \mathbf{+36.73\ kcal\ mol^{-1}}$

$\Delta E_0 = \Delta E_{\text{final}} + \Delta\text{ZPVE(harm)} = 36.73 - 4.23 = 32.50\ \text{kcal mol}^{-1}$

Double Group Transfer

	ΔE_e(HF)	$+\delta$ [MP2]	$+\delta$[CCSD]	$+\delta$ [CCSD(T)]	$+\delta$[CCSDT(Q)]	NET
cc–pVDZ	+80.06	–36.85	+12.53	–4.69	+0.30–0.56	+50.79
cc–pVTZ	+82.01	–39.46	+13.82	–5.73	[–0.26]	[+50.38]
cc–pVQZ	+82.29	–40.04	+14.07	–5.98	[–0.26]	[+50.08]
cc–pV5Z	+82.39	–40.08	[+14.00]	[–6.07]	[–0.26]	[+49.98]
CBS LIMIT	[+82.44]	[–40.13]	[+13.92]	[–6.16]	[–0.26]	[+49.81]
FUNCTION<br>X (fit points)	$a+be^{-cX}$<br>(3,4,5)	$a+bX^{-3}$<br>(4,5)	$a+bX^{-3}$<br>(3,4)	$a+bX^{-3}$<br>(3,4)	addition	

$\Delta E_{\text{final}} = \Delta E_e(\text{FPA}) + \Delta(\text{DBOC}) + \Delta(\text{rel}) + \Delta(\text{core}) + \Delta(\text{aug})$

$\Delta E_{\text{final}} = 49.81 + 0.07 + 0.02 + 0.00 + 0.23 = \mathbf{+50.13\ kcal\ mol^{-1}}$

$\Delta E_0 = \Delta E_{\text{final}} + \Delta\text{ZPVE(harm)} = 50.13 - 2.44 = 47.69\ \text{kcal mol}^{-1}$

Table S3. Cartesian coordinates (in Å), energies (in Hartree), and number of imaginary frequencies of all stationary points, computed at CCSD(T)/cc-pVTZ.

Butadiene (B)

E = −155.6913388

N_{imag} = 0

C	0.0780090576	-1.5307847530	0.5103413895
C	-0.1224830096	-0.7267102628	-0.5457694015
C	0.1224830096	0.7267102628	-0.5457694015
C	-0.0780090576	1.5307847530	0.5103413895
H	0.4751302417	-1.1448647296	1.4422287601
H	-0.1385588653	-2.5900298062	0.4577781798
H	-0.4716717238	-1.1602557384	-1.4781716701
H	0.4716717238	1.1602557384	-1.4781716701
H	-0.4751302417	1.1448647296	1.4422287601
H	0.1385588653	2.5900298062	0.4577781798

***trans*-Butadiene**

E = −155.6959336

N_{imag} = 0

C	0.6031763012	1.7499202724	0.0000000000
C	0.6072620711	0.4060073418	0.0000000000
C	-0.6072620711	-0.4060073418	0.0000000000
H	1.5522570424	-0.1299924668	0.0000000000
H	-1.5522570424	0.1299924668	0.0000000000
C	-0.6031763012	-1.7499202724	0.0000000000
H	0.3280364927	-2.3056054391	0.0000000000
H	1.5236702684	2.3188843928	0.0000000000
H	-0.3280364927	2.3056054391	0.0000000000
H	-1.5236702684	-2.3188843928	0.0000000000

Ethylene (E)

E = −78.43880351

N_{imag} = 0

C	0.6685655813	0.0000000000	0.0000000000
C	-0.6685655813	0.0000000000	0.0000000000
H	1.2337633984	0.9240321875	0.0000000000
H	1.2337633984	-0.9240321875	0.0000000000
H	-1.2337633984	0.9240321875	0.0000000000
H	-1.2337633984	-0.9240321875	0.0000000000

TS: DA
E = −234.0987534
N_{imag} = 1,618.9i cm^{-1}

C	-0.4154889803	-1.4230046298	0.4914165794
C	-1.2714421328	-0.7068920916	-0.3302462784
C	-1.2748595730	0.7006702447	-0.3303631561
C	-0.4223986696	1.4210749167	0.4911783485
H	-0.1284754598	-1.0289393722	1.4569991839
H	-0.3497935865	-2.5007558373	0.3945333287
H	-1.7811020584	-1.2250035542	-1.1356241613
H	-1.7870336076	1.2161650530	-1.1358251637
H	-0.1334714665	1.0285808294	1.4568319204
H	-0.3619410862	2.4991169969	0.3941076830
C	1.5821961637	0.6990828245	-0.1805625523
C	1.5855716595	-0.6914587859	-0.1804052311
H	2.0580658056	1.2364955663	0.6305385466
H	1.4952914442	1.2373987055	-1.1142543585
H	2.0640419870	-1.2263684332	0.6308226672
H	1.5013121389	-1.2304088475	-1.1139741963

P: DA
E = −234.2058720
N_{imag} = 0

C	0.1078301460	-1.4969587160	-0.0176383714
C	0.0577077454	-0.6680994266	-1.2779887795
C	-0.0577077454	0.6680994266	-1.2779887795
C	-0.1078301460	1.4969587160	-0.0176383714
H	-0.5253984796	-2.3813778370	-0.1373130930
H	1.1280674294	-1.8722030397	0.1318306047
H	0.1150434038	-1.1945828603	-2.2256951591
H	-0.1150434038	1.1945828603	-2.2256951591
H	-1.1280674294	1.8722030397	0.1318306047
H	0.5253984796	2.3813778370	-0.1373130930
C	0.3348534264	0.6882093345	1.2059578351
C	-0.3348534264	-0.6882093345	1.2059578351
H	0.1023546512	1.2337810408	2.1239820104
H	1.4221818043	0.5547640764	1.1748728185
H	-1.4221818043	-0.5547640764	1.1748728185
H	-0.1023546512	-1.2337810408	2.1239820104

Methylazide (MA)
E = −203.7684651
N_{imag} = 0

N	1.6940531799	0.2636398203	0.0000000000
N	0.6110655338	-0.0955013960	0.0000000000
N	-0.5129188495	-0.6156378581	0.0000000000
C	-1.6124164288	0.3705251895	0.0000000000
H	-2.5384425885	-0.1973521305	0.0000000000
H	-1.5821219680	1.0016430114	-0.8915076049
H	-1.5821219680	1.0016430114	0.8915076049

TS: 1,3-DC
E = −282.1775562
N_{imag} = 1,515.2i cm^{-1}

N	-0.8615904666	-1.3715610485	-0.1281117290
N	0.2397098768	-1.0208060276	0.0748565326
N	0.8474946919	0.0509335256	0.4042230481
C	2.1253621159	0.2972713149	-0.2720273779
H	2.4517576921	1.2879182477	0.0394885503
H	2.0267280064	0.2776100526	-1.3615543912
H	2.8786574489	-0.4282240514	0.0381319994
C	-1.8071946641	0.5608189941	-0.1328772802
C	-0.7371188633	1.4038621723	0.1107110035
H	-2.4840386425	0.2952290384	0.6686360877
H	-2.1710533230	0.4122823877	-1.1401730524
H	-0.5871778424	1.8288863896	1.0935952748
H	-0.2612520155	1.9263151283	-0.7116812868

P: 1,3-DC
E = −282.2519795
N_{imag} = 0

N	-1.2636521870	-0.8558708395	-0.1377172120
N	-0.0484387373	-1.0946189317	0.0273335357
N	0.6880173747	0.0542478231	0.3376083808
C	2.0637178004	-0.0258287505	-0.1258983926
H	2.6484737478	0.7606851972	0.3515026260
H	2.1369685960	0.0879012306	-1.2157850323
H	2.4700918157	-0.9938962776	0.1607419616
C	-1.5038377896	0.6075159015	0.0380327666
C	-0.1089392980	1.2035535734	-0.0974019212
H	-1.9138750406	0.7494265416	1.0420049047
H	-2.2281739337	0.9532950601	-0.6953881326
H	0.0631082301	2.0649981210	0.5448227888
H	0.1252292629	1.4680844828	-1.1390898046

TS: ER
E = −155.6222873
N_{imag} = 1,760.0i cm^{-1}

C	-1.0666140363	-0.6535552579	0.1073104694
C	-0.6824585285	0.7129890786	-0.0936233528
C	0.6823097975	0.7131287592	0.0936368753
C	1.0667503382	-0.6533320659	-0.1073230776
H	-0.8668911855	-1.1003572755	1.0708836185
H	-1.8685554516	-1.1308943621	-0.4569205267
H	-1.3362035640	1.5210005662	-0.4020313330
H	1.3358817170	1.5212692170	0.4020681124
H	0.8671248293	-1.1001553744	-1.0709060115
H	1.8687916458	-1.1305200750	0.4568952543

P: ER

E = −155.6776100

***N*imag** = 0

C	0.7858329668	0.0000000000	-0.7271128451
C	0.6734918648	0.0000000000	0.7908206581
C	-0.6734918648	0.0000000000	0.7908206581
C	-0.7858329668	0.0000000000	-0.7271128451
H	1.2406036241	0.8896182834	-1.1674992689
H	1.2406036241	-0.8896182834	-1.1674992689
H	1.4187784135	0.0000000000	1.5764405464
H	-1.4187784135	0.0000000000	1.5764405464
H	-1.2406036241	-0.8896182834	-1.1674992689
H	-1.2406036241	0.8896182834	-1.1674992689

(*Z*)-penta-1,3-diene (P)

E = −194.9332918

***N*imag** = 0

C	-1.6360350254	0.8505601039	0.1845632501
C	-1.2775280324	-0.3716530045	-0.2424735716
C	-0.0155657702	-1.0614943258	0.0748834466
C	1.2159134053	-0.5196405440	0.1345053410
C	1.5909583114	0.9089741743	-0.1431850908
H	-2.5913775415	1.2786748623	-0.0924530998
H	-0.9981074929	1.4342652575	0.8368313382
H	-1.9836154152	-0.9328111692	-0.8488735534
H	-0.0988818219	-2.1308480370	0.2463388562
H	2.0346055020	-1.1864651801	0.3889073658
H	2.5214994984	0.9508209466	-0.7137551391
H	1.7604619367	1.4609915280	0.7866879860
H	0.8111098128	1.4264092528	-0.7024315521

(*E*)-penta-1,3-diene

E = −194.9378227

***N*imag** = 0

C	-1.9664379752	-0.9229607524	0.0000000000
C	-1.3361140609	0.4407726013	0.0000000000
C	-0.0188623542	0.7255767591	0.0000000000
C	1.0776013191	-0.2399270821	0.0000000000
C	2.3725351048	0.1235673038	0.0000000000
H	-2.0272932162	1.2785494285	0.0000000000
H	0.2775705580	1.7708387100	0.0000000000
H	0.8304224963	-1.2957286992	0.0000000000
H	3.1666073000	-0.6117948613	0.0000000000
H	2.6599882618	1.1693687558	0.0000000000
H	-1.2300469803	-1.7254842137	0.0000000000
H	-2.6049598100	-1.0491298167	0.8788128700
H	-2.6049598100	-1.0491298167	-0.8788128700

TS: SR
$\boldsymbol{E}$ = −194.8743033
$\boldsymbol{N}_{\mathbf{imag}}$ = 1,1690.1i cm^{-1}

C	-1.2993759223	-0.9430169372	0.0054131718
C	-1.2102761001	0.4742672423	0.0315877962
C	0.0004274533	1.1578132068	-0.1175233788
C	1.2106183055	0.4733922158	0.0317305502
C	1.2986886484	-0.9439538441	0.0055515290
H	-2.1671140916	-1.3799842724	0.4951685910
H	-1.0338532234	-1.4667982882	-0.9069115660
H	-2.0425012193	1.0437191605	0.4343446847
H	0.0008166627	2.2373077878	-0.0245940109
H	2.0432108719	1.0422409678	0.4345809623
H	2.1660571733	-1.3815630575	0.4953882069
H	1.0328772200	-1.4675174332	-0.9068131896
H	-0.0004743347	-1.2290693603	0.4936915441

Ethane (Et)
$\boldsymbol{E}$ = −79.6744450
$\boldsymbol{N}_{\mathbf{imag}}$ = 0

C	0.0000005113	0.7644753913	0.0000000000
C	-0.0000005113	-0.7644753913	0.0000000000
H	-0.5089680268	1.1592823746	-0.8815621296
H	-0.5089680268	1.1592823746	0.8815621296
H	1.0179396811	1.1592868837	0.0000000000
H	0.5089680268	-1.1592823746	0.8815621296
H	-1.0179396811	-1.1592868837	0.0000000000
H	0.5089680268	-1.1592823746	-0.8815621296

TS: DGT
$\boldsymbol{E}$ = −158.0325438
$\boldsymbol{N}_{\mathbf{imag}}$ = 1,1780.0i cm^{-1}

C	1.3366737481	0.7118771527	0.0000160548
C	1.3366612865	-0.7118996217	0.0000128249
C	-1.3366726002	-0.7118773510	-0.0000233917
C	-1.3366615093	0.7119000585	-0.0000053194
H	1.6264256070	1.2279009228	0.9102460712
H	1.6264354758	1.2279028481	-0.9102098664
H	0.0000099553	0.9800236631	0.0000093965
H	1.6263978569	-1.2279323045	0.9102424798
H	1.6264209108	-1.2279286393	-0.9102112416
H	-0.0000085634	-0.9800236253	-0.0000078012
H	-1.6264440753	-1.2279155100	0.9101921521
H	-1.6264188561	-1.2278912370	-0.9102605349
H	-1.6264237928	1.2279182694	0.9102246001
H	-1.6264055352	1.2279427732	-0.9102272627

Table S4. Cartesian coordinates (in Å), energies (in kcal mol^{-1}), and number of imaginary frequencies of all stationary points, computed at BP86/DZ.

Butadiene (B)

E = −1254.62
H = −1198.89
G = −1218.75
N_{imag} = 0

C	1.369582	-0.736873	-0.497167
C	0.711233	-0.193600	0.545296
C	-0.711232	0.193602	0.545297
C	-1.369582	0.736877	-0.497166
H	0.860239	-0.975991	-1.434093
H	2.430863	-0.983754	-0.429620
H	1.247159	-0.036264	1.488737
H	-1.247159	0.036262	1.488736
H	-0.860240	0.975991	-1.434093
H	-2.430864	0.983750	-0.429621

Ethylene (E)

E = −709.07
H = −675.19
G = −690.80
N_{imag} = 0

C	0.669518	0.000000	0.000000
C	-0.669518	0.000000	-0.000000
H	1.243549	-0.929860	0.000000
H	1.243549	0.929860	0.000000
H	-1.243549	-0.929860	-0.000000
H	-1.243549	0.929860	-0.000000

TS: DA

E = −1950.85
H = −1860.33
G = −1883.67
N_{imag} = 1,−443.475i cm^{-1}

C	1.800396	-0.553240	-1.014642
C	1.571473	-1.659419	-0.204153
C	0.267348	-2.116291	0.098834
C	-0.871506	-1.489130	-0.393770
H	1.100371	0.282598	-1.029645
H	2.819200	-0.309073	-1.325519
H	2.413315	-2.311205	0.048857
H	0.166332	-3.098391	0.570845
H	-0.885120	-0.412858	-0.568058
H	-1.852537	-1.945294	-0.239836
C	-0.526956	-1.644813	-2.656227
C	0.757435	-1.194614	-2.954996
H	-1.389337	-1.001903	-2.846246
H	-0.735223	-2.714621	-2.623703
H	0.902502	-0.198529	-3.379155

H	1.553173	-1.912479	-3.155973

P: DA

E = −2006.01
H = −1912.18
G = −1934.26
N_{imag} = 0

C	1.921287	-1.072436	-1.535850
C	1.464946	-1.465155	-0.142835
C	0.190227	-1.773008	0.161937
C	-0.931612	-1.800692	-0.859719
H	2.607606	-0.209179	-1.469878
H	2.509313	-1.901487	-1.977151
H	2.229143	-1.492101	0.641441
H	-0.072878	-2.020914	1.195926
H	-1.597476	-0.929933	-0.697649
H	-1.554750	-2.698841	-0.699535
C	-0.389164	-1.786239	-2.308138
C	0.726066	-0.727346	-2.454896
H	-1.208793	-1.594036	-3.020306
H	0.029180	-2.782032	-2.544043
H	0.323155	0.259983	-2.163167
H	1.060239	-0.652310	-3.503055

Methylazide (MA)

E = −897.62
H = −863.98
G = −884.19
N_{imag} = 0

N	1.065184	0.200151	-0.292862
N	-0.063755	-0.034550	-0.074257
N	-1.303718	-0.060728	0.138230
C	-1.949762	-1.392661	0.404572
H	-3.010601	-1.177603	0.554354
H	-1.533545	-1.855565	1.312115
H	-1.826041	-2.067535	-0.455873

TS: 1,3-DC

E = −1600.58
H = −1532.33
G = −1557.08
N_{imag} = 1,−275.361i cm^{-1}

N	-1.453714	-2.606634	-0.087487
N	-2.599452	-2.229607	-0.050277
N	-3.239397	-1.114542	-0.135235
C	-4.663568	-1.005488	0.260525
H	-4.939996	0.040863	0.088083
H	-4.815089	-1.254479	1.322787
H	-5.296848	-1.648281	-0.369303
C	-0.467837	-0.607966	-0.201916
C	-1.537989	0.272357	-0.192562

H	0.011944	-0.886638	-1.140815
H	0.089702	-0.817314	0.711185
H	-1.909113	0.705630	-1.121281
H	-1.835819	0.774023	0.729666

P: 1,3-DC

E = −1629.23

H = −1558.83

G = −1581.93

N_{imag} = 0

N	-1.598594	-2.668826	-0.156446
N	-2.887784	-2.589786	-0.121688
N	-3.346475	-1.254366	-0.192625
C	-4.726462	-0.963061	0.175195
H	-5.138537	-0.190640	-0.490043
H	-4.809841	-0.623480	1.224636
H	-5.302847	-1.888217	0.049423
C	-1.009028	-1.231969	-0.270245
C	-2.218772	-0.291001	-0.044470
H	-0.591224	-1.151836	-1.284259
H	-0.208276	-1.136960	0.470685
H	-2.291774	0.501115	-0.800595
H	-2.226419	0.155042	0.967428

TS: ER

E = −1212.92

H = −1158.56

G = −1177.25

N_{imag} = 1,-673.245i cm^{-1}

C	-1.045530	0.743702	0.036923
C	-0.628557	-0.574515	0.460714
C	0.727013	-0.609946	0.193360
C	1.113580	0.780594	0.103548
H	-0.944719	0.995709	-1.019649
H	-1.810546	1.320043	0.576081
H	-1.257097	-1.324619	0.946848
H	1.373630	-1.472115	0.012785
H	1.002966	1.399558	0.994989
H	1.868132	1.136058	-0.612259

P: ER

E = −1240.65

H = −1184.77

G = −1203.52

N_{imag} = 0

C	-0.757433	0.808095	-0.022342
C	-0.632691	-0.692791	0.293790
C	0.716104	-0.665043	0.417358
C	0.834890	0.840847	0.123679
H	-1.127418	1.050799	-1.030320
H	-1.296021	1.407202	0.727865

H	-1.374458	-1.486907	0.383705
H	1.460933	-1.428581	0.643498
H	1.203143	1.458516	0.957175
H	1.371822	1.102334	-0.801069

(*Z*)-penta-1,3-diene (P)

E = −1622.19

H = −1548.11

G = −1570.61

N_{imag} = 0

C	-1.841787	0.303104	-0.303273
C	-0.897610	1.146778	0.160893
C	0.551998	1.054935	-0.078805
C	1.319966	-0.060104	-0.079178
C	0.877391	-1.470298	0.215269
H	-2.898323	0.461338	-0.076699
H	-1.594729	-0.536209	-0.956361
H	-1.218662	2.016226	0.748238
H	1.055546	2.015759	-0.238093
H	2.389362	0.068990	-0.281817
H	1.634741	-1.984662	0.830094
H	0.763147	-2.059684	-0.711944
H	-0.082319	-1.493643	0.753273

TS: SR

E = −1591.44

H = −1520.33

G = −1541.03

N_{imag} = 1,−1409.899i cm^{-1}

C	-1.531970	-0.014108	-0.091902
C	-0.712590	1.151592	-0.157446
C	0.689599	1.091413	-0.247228
C	1.371146	-0.096734	0.071565
C	0.717745	-1.361863	0.155255
H	-2.520852	0.113194	0.366137
H	-1.567915	-0.676494	-0.964400
H	-1.146292	2.120317	0.114193
H	1.252403	2.027540	-0.265517
H	2.373173	0.011841	0.501058
H	1.209231	-2.121534	0.775924
H	0.295244	-1.792555	-0.759782
H	-0.596006	-0.913636	0.520914

Ethane (Et)

E = −901.35

H = −852.60

G = −868.81

N_{imag} = 0

C	0.628873	-0.360155	0.261911
C	-0.628873	0.360155	-0.261911
H	1.288587	0.337515	0.802102

H	0.359691	-1.174153	0.953784
H	1.207700	-0.798958	-0.566431
H	-1.288554	-0.337500	-0.802162
H	-1.207737	0.798899	0.566436
H	-0.359688	1.174199	-0.953730

TS: DGT

E = -1570.98

H = -1491.95

G = -1512.22

N_{imag} = 1,-1437.768i cm^{-1}

C	1.348528	0.715753	0.000000
C	1.348527	-0.715753	-0.000000
C	-1.348527	-0.715754	0.000000
C	-1.348526	0.715754	-0.000000
H	1.653199	1.233048	0.916967
H	1.653199	1.233048	-0.916967
H	-0.000001	1.011447	-0.000000
H	1.653199	-1.233048	0.916967
H	1.653199	-1.233048	-0.916967
H	0.000000	-1.011447	-0.000000
H	-1.653199	-1.233048	0.916967
H	-1.653199	-1.233048	-0.916967
H	-1.653199	1.233048	0.916967
H	-1.653199	1.233048	-0.916967

Table S5. Cartesian coordinates (in Å), energies (in kcal mol^{-1}), and number of imaginary frequencies of all stationary points, computed at BP86/DZP.

Butadiene (B)

E = −1283.74
H = −1228.37
G = −1248.28
N_{imag} = 0

C	1.364051	-0.728859	-0.496452
C	0.706097	-0.196163	0.543842
C	-0.706097	0.196163	0.543841
C	-1.364051	0.728858	-0.496453
H	0.858973	-0.947056	-1.438429
H	2.421730	-0.980159	-0.426094
H	1.240131	-0.059379	1.490287
H	-1.240131	0.059379	1.490286
H	-0.858973	0.947057	-1.438429
H	-2.421730	0.980160	-0.426094

Ethylene (E)

E = −723.77
H = −690.13
G = −705.75
N_{imag} = 0

C	0.666331	0.000000	-0.000000
C	-0.666331	0.000000	0.000000
H	1.238286	-0.928845	-0.000000
H	1.238286	0.928845	-0.000000
H	-1.238286	-0.928845	0.000000
H	-1.238286	0.928845	0.000000

TS: DA

E = −1994.16
H = −1904.30
G = −1927.78
N_{imag} = 1,−445.460i cm^{-1}

C	1.790955	-0.560187	-1.016420
C	1.566752	-1.655501	-0.200966
C	0.269671	-2.110001	0.100377
C	-0.861451	-1.489429	-0.400091
H	1.086906	0.269789	-1.037488
H	2.807016	-0.310776	-1.325546
H	2.407483	-2.310493	0.042699
H	0.167782	-3.095292	0.562994
H	-0.871327	-0.416211	-0.582109
H	-1.842929	-1.939724	-0.244956
C	-0.521975	-1.644059	-2.649146
C	0.756387	-1.196252	-2.946501
H	-1.380692	-0.998459	-2.834664
H	-0.733549	-2.711499	-2.622828
H	0.899473	-0.199727	-3.364917

H	1.550365	-1.911442	-3.153824

P: DA
E = −2049.89
H = −1956.73
G = −1978.87
N_{imag} = 0

C	1.910760	-1.072173	-1.532797
C	1.459776	-1.464694	-0.150125
C	0.190988	-1.771103	0.153247
C	-0.921054	-1.800929	-0.862542
H	2.596527	-0.210921	-1.465703
H	2.509979	-1.893977	-1.968324
H	2.220077	-1.492092	0.635984
H	-0.067780	-2.018870	1.186890
H	-1.594119	-0.939493	-0.692154
H	-1.543088	-2.697364	-0.701167
C	-0.386920	-1.779007	-2.301502
C	0.726902	-0.736283	-2.450356
H	-1.206087	-1.581513	-3.009842
H	0.019185	-2.775570	-2.548329
H	0.329131	0.255760	-2.173589
H	1.062210	-0.667494	-3.496606

Methylazide (MA)
E = −962.84
H = −928.72
G = −948.81
N_{imag} = 0

N	1.013568	0.118621	-0.271370
N	-0.106814	-0.033107	-0.067297
N	-1.321971	-0.045650	0.135905
C	-1.924995	-1.365532	0.396773
H	-2.992402	-1.195136	0.555621
H	-1.496980	-1.827621	1.297778
H.	-1.792643	-2.040066	-0.461130

TS: 1,3-DC
E = −1674.21
H = −1605.95
G = −1630.09
N_{imag} = 1,−353.398i cm^{-1}

N	-1.489250	-2.576890	-0.144944
N	-2.616130	-2.223637	-0.191263
N	-3.256515	-1.143778	-0.363906
C	-4.564179	-0.985119	0.267331
H	-4.905363	0.026976	0.027347
H	-4.518222	-1.100543	1.361099
H	-5.292113	-1.697296	-0.144953
C	-0.534872	-0.633770	-0.137734
C	-1.609303	0.234548	-0.200042

H	0.038157	-0.862150	-1.035750
H	-0.045809	-0.842471	0.812224
H	-1.883618	0.708646	-1.141010
H	-1.979959	0.717409	0.704970

P: 1,3-DC

E = −1707.51

H = −1636.98

G = −1659.50

N_{imag} = 0

N	-1.622551	-2.609217	-0.020953
N	-2.875531	-2.543293	-0.073359
N	-3.339034	-1.258321	-0.334232
C	-4.676791	-0.971921	0.151185
H	-5.099364	-0.129021	-0.408937
H	-4.681331	-0.726049	1.228727
H	-5.299329	-1.857157	-0.016350
C	-1.048326	-1.241215	-0.255369
C	-2.245397	-0.309705	-0.069523
H	-0.668799	-1.229084	-1.290426
H	-0.211138	-1.068521	0.429621
H	-2.269742	0.526940	-0.777791
H	-2.318699	0.082579	0.964402

TS: ER

E = −1243.88

H = −1189.87

G = −1208.60

N_{imag} = 1,−676.079i cm^{-1}

C	-1.036802	0.735178	0.038978
C	-0.625309	-0.569072	0.459650
C	0.723632	-0.604579	0.192329
C	1.105038	0.771713	0.104771
H	-0.919851	0.994944	-1.011298
H	-1.809657	1.313320	0.563571
H	-1.252238	-1.311688	0.959808
H	1.368572	-1.464945	-0.003928
H	0.978147	1.395204	0.987606
H	1.867340	1.134393	-0.598147

P: ER

E = −1273.70

H = −1218.15

G = −1236.91

N_{imag} = 0

C	-0.744870	0.798200	-0.019133
C	-0.627665	-0.682931	0.292274
C	0.711045	-0.655389	0.414906
C	0.822360	0.830436	0.124588
H	-1.119804	1.046108	-1.023723
H	-1.287517	1.400610	0.725095

H	-1.372865	-1.474862	0.381421
H	1.459291	-1.416601	0.640922
H	1.195566	1.451586	0.952932
H	1.363330	1.097314	-0.795944

(*Z*)-penta-1,3-diene (P)

E = −1658.97

H = −1585.40

G = −1608.03

N_{imag} = 0

C	-1.840642	0.297529	-0.279756
C	-0.894584	1.151533	0.143955
C	0.548319	1.048548	-0.074592
C	1.313818	-0.061541	-0.068670
C	0.877766	-1.467522	0.198233
H	-2.893571	0.478124	-0.064044
H	-1.600303	-0.579534	-0.879899
H	-1.218836	2.050704	0.680519
H	1.062526	2.005043	-0.213889
H	2.384382	0.079132	-0.248572
H	1.621499	-1.986542	0.820577
H	0.793937	-2.044835	-0.737024
H	-0.095591	-1.508106	0.704759

TS: SR

E = −1630.18

H = −1559.65

G = −1580.41

N_{imag} = 1,−1380.954i cm^{-1}

C	-1.523616	-0.014327	-0.092343
C	-0.707986	1.142243	-0.163775
C	0.686285	1.085011	-0.253224
C	1.361905	-0.097745	0.062834
C	0.713919	-1.354742	0.152587
H	-2.513345	0.115176	0.359463
H	-1.554267	-0.690602	-0.950129
H	-1.135138	2.108858	0.121331
H	1.246936	2.021621	-0.249402
H	2.357215	0.016712	0.503680
H	1.209173	-2.114877	0.766904
H	0.273931	-1.785772	-0.750028
H	-0.582099	-0.892582	0.510872

Ethane (Et)

E = −916.98

H = −868.64

G = −884.89

N_{imag} = 0

C	-0.000000	0.000002	0.764569
C	0.000000	-0.000001	-0.764569
H	0.000032	1.023684	1.164870

H	-0.886539	-0.511811	1.164860
H	0.886507	-0.511865	1.164860
H	0.000032	-1.023684	-1.164870
H	-0.886539	0.511811	-1.164861
H	0.886508	0.511865	-1.164861

TS: DGT

E = -1602.75

H = -1524.59

G = -1544.93

N_{imag} = 1,-1419.043i cm^{-1}

C	1.342416	0.710731	0.000000
C	1.342415	-0.710731	0.000000
C	-1.342415	-0.710731	0.000000
C	-1.342414	0.710731	0.000000
H	1.636963	1.232074	0.914924
H	1.636963	1.232074	-0.914924
H	-0.000000	0.993152	-0.000000
H	1.636963	-1.232074	0.914924
H	1.636963	-1.232074	-0.914924
H	-0.000000	-0.993152	-0.000000
H	-1.636963	-1.232074	0.914924
H	-1.636963	-1.232074	-0.914924
H	-1.636963	1.232074	0.914924
H	-1.636963	1.232074	-0.914924

Table S6. Cartesian coordinates (in Å), energies (in kcal mol^{-1}), and number of imaginary frequencies of all stationary points, computed at BP86/TZ2P.

Butadiene (B)
E = −1294.32
H = −1239.01
G = −1258.99
N_{imag} = 0

C	1.372973	-0.713387	-0.499661
C	0.701870	-0.211314	0.547730
C	-0.701870	0.211314	0.547730
C	-1.372973	0.713387	-0.499661
H	0.889690	-0.878529	-1.463676
H	2.423410	-0.989486	-0.417905
H	1.222395	-0.125776	1.506665
H	-1.222395	0.125776	1.506665
H	-0.889690	0.878529	-1.463676
H	-2.423410	0.989486	-0.417905

Ethylene (E)
E = −729.02
H = −695.42
G = −711.04
N_{imag} = 0

C	0.666078	0.000000	0.000000
C	-0.666078	0.000000	-0.000000
H	1.239044	-0.928195	0.000000
H	1.239044	0.928195	0.000000
H	-1.239044	-0.928195	-0.000000
H	-1.239044	0.928195	-0.000000

TS: DA
E = −2008.13
H = −1918.34
G = −1941.87
N_{imag} = 1,-439.759i cm^{-1}

C	1.794194	-0.563001	-1.014172
C	1.568352	-1.655043	-0.196824
C	0.269821	-2.109924	0.104832
C	-0.861121	-1.493055	-0.397314
H	1.085504	0.261248	-1.047841
H	2.808635	-0.311661	-1.323530
H	2.408765	-2.307241	0.051886
H	0.168296	-3.092083	0.572325
H	-0.869154	-0.423362	-0.593514
H	-1.842786	-1.940714	-0.242749
C	-0.522871	-1.642801	-2.654218
C	0.754052	-1.195158	-2.951366
H	-1.381146	-0.995419	-2.829019
H	-0.737809	-2.708400	-2.630317
H	0.899505	-0.195829	-3.359388

H	1.548628	-1.906820	-3.162179

P: DA

E = −2064.96
H = −1971.93
G = −1994.13
N_{imag} = 0

C	1.912368	-1.069606	-1.529800
C	1.459889	-1.463875	-0.147285
C	0.192266	-1.772754	0.155390
C	-0.921135	-1.804274	-0.859978
H	2.595696	-0.207938	-1.462109
H	2.517238	-1.886382	-1.963940
H	2.219264	-1.488375	0.638654
H	-0.066010	-2.023259	1.187615
H	-1.598617	-0.948357	-0.687025
H	-1.540940	-2.700978	-0.699417
C	-0.390748	-1.775792	-2.301437
C	0.729983	-0.739133	-2.453137
H	-1.210151	-1.568271	-3.004507
H	0.003909	-2.771885	-2.560038
H	0.336080	0.256585	-2.191960
H	1.067397	-0.681432	-3.497942

Methylazide (MA)

E = −978.03
H = −943.98
G = −964.08
N_{imag} = 0

N	1.015408	0.132362	-0.273421
N	-0.100404	-0.032138	-0.068465
N	-1.314929	-0.051388	0.135508
C	-1.925774	-1.366367	0.397012
H	-2.992826	-1.189246	0.554925
H	-1.504263	-1.834793	1.298737
H	-1.799451	-2.046922	-0.458016

TS: 1,3-DC

E = −1691.54
H = −1623.37
G = −1647.65
N_{imag} = 1,−351.971i cm^{-1}

N	-1.485579	-2.555466	-0.135033
N	-2.612270	-2.206284	-0.161471
N	-3.261002	-1.131924	-0.317303
C	-4.592925	-0.998233	0.265651
H	-4.938033	0.013869	0.029339
H	-4.590702	-1.127716	1.359436
H	-5.299347	-1.712236	-0.180868
C	-0.520204	-0.638473	-0.151744
C	-1.576424	0.250494	-0.198329

H	0.033394	-0.880706	-1.057233
H	-0.014569	-0.848906	0.788136
H	-1.871669	0.716185	-1.135909
H	-1.927844	0.741320	0.708696

P: 1,3-DC
E = −1727.16
H = −1656.66
G = −1679.22
N_{imag} = 0

N	-1.626675	-2.613453	-0.056594
N	-2.877984	-2.541208	-0.092136
N	-3.340169	-1.257523	-0.313679
C	-4.682239	-0.973279	0.158636
H	-5.111392	-0.143155	-0.416295
H	-4.695694	-0.706449	1.231617
H	-5.296541	-1.866715	0.006296
C	-1.045538	-1.247184	-0.255996
C	-2.242377	-0.308813	-0.070733
H	-0.637353	-1.209667	-1.278234
H	-0.222011	-1.089639	0.448586
H	-2.270309	0.518743	-0.788505
H	-2.307750	0.104356	0.954032

TS: ER
E = −1254.39
H = −1200.45
G = −1219.18
N_{imag} = 1,−685.395i cm^{-1}

C	-1.036503	0.732374	0.040887
C	-0.625730	-0.571044	0.460915
C	0.724102	-0.606863	0.191864
C	1.104818	0.768394	0.104029
H	-0.910924	1.002860	-1.004641
H	-1.810847	1.311699	0.561696
H	-1.252699	-1.310994	0.963110
H	1.369032	-1.465488	-0.007259
H	0.969061	1.399948	0.978546
H	1.868562	1.133585	-0.595806

P: ER
E = −1285.51
H = −1229.94
G = −1248.68
N_{imag} = 0

C	-0.744906	0.796855	-0.018859
C	-0.627538	-0.684895	0.292669
C	0.710926	-0.657355	0.415303
C	0.822408	0.829104	0.124861
H	-1.121102	1.048717	-1.020995
H	-1.288193	1.401906	0.721410

H	-1.373437	-1.475521	0.381474
H	1.459856	-1.417237	0.641136
H	1.196869	1.452938	0.949400
H	1.363987	1.099959	-0.793060

(*Z*)-penta-1,3-diene (P)
E = −1672.19
H = −1598.71
G = −1621.39
N_{imag} = 0

C	-1.845053	0.285954	-0.252571
C	-0.896170	1.163728	0.115941
C	0.550746	1.051029	-0.062207
C	1.319242	-0.057631	-0.049531
C	0.881886	-1.471789	0.177332
H	-2.898361	0.492817	-0.065443
H	-1.611909	-0.638616	-0.778762
H	-1.227650	2.100702	0.576335
H	1.071560	2.006800	-0.173150
H	2.393454	0.089142	-0.192564
H	1.629201	-2.017747	0.770126
H	0.778949	-2.018168	-0.774776
H	-0.087172	-1.523691	0.690867

TS: SR
E = −1643.99
H = −1573.49
G = −1594.24
N_{imag} = 1,−1362.913i cm^{-1}

C	-1.524474	-0.013102	-0.092843
C	-0.707221	1.143481	-0.163880
C	0.687150	1.086946	-0.250375
C	1.362680	-0.096496	0.062836
C	0.715469	-1.354938	0.152398
H	-2.515001	0.114791	0.356003
H	-1.552507	-0.696321	-0.944672
H	-1.137092	2.109219	0.116861
H	1.247188	2.023161	-0.243233
H	2.359130	0.014768	0.499844
H	1.210044	-2.116866	0.763803
H	0.267319	-1.786359	-0.745510
H	-0.579769	-0.889310	0.507538

Ethane (Et)
E = −923.07
H = −874.79
G = −891.06
N_{imag} = 0

C	0.000000	-0.000002	0.765181
C	0.000000	0.000002	-0.765182
H	0.000071	1.022833	1.166039

H	-0.885828	-0.511351	1.166031
H	0.885757	-0.511473	1.166031
H	0.000071	-1.022833	-1.166039
H	-0.885828	0.511351	-1.166031
H	0.885758	0.511474	-1.166032

TS: DGT

E = −1613.98

H = −1535.75

G = −1556.05

N_{imag} = 1,−1402.654i cm^{-1}

C	1.344882	0.711606	0.000000
C	1.344882	-0.711606	0.000000
C	-1.344881	-0.711606	0.000000
C	-1.344881	0.711606	0.000000
H	1.638469	1.233567	0.913816
H	1.638469	1.233567	-0.913816
H	-0.000000	0.989454	-0.000000
H	1.638468	-1.233567	0.913816
H	1.638468	-1.233567	-0.913816
H	-0.000000	-0.989454	-0.000000
H	-1.638469	-1.233567	0.913815
H	-1.638469	-1.233567	-0.913815
H	-1.638469	1.233567	0.913816
H	-1.638469	1.233567	-0.913816

Table S7. Cartesian coordinates (in Å), energies (in kcal mol^{-1}), and number of imaginary frequencies of all stationary points, computed at BP86/QZ4P.

Butadiene (B)

E = −1296.96
H = −1241.69
G = -1261.66
N_{imag} = 0

C	1.372128	-0.715849	-0.498970
C	0.702432	-0.209641	0.547858
C	-0.702432	0.209641	0.547858
C	-1.372128	0.715849	-0.498969
H	0.886617	-0.887995	-1.460041
H	2.423245	-0.988288	-0.420019
H	1.224767	-0.116974	1.504325
H	-1.224767	0.116974	1.504325
H	-0.886617	0.887995	-1.460041
H	-2.423245	0.988288	-0.420020

Ethylene (E)

E = −730.29
H = −696.73
G = −712.35
N_{imag} = 0

C	0.666262	-0.000000	-0.000000
C	-0.666262	-0.000000	0.000000
H	1.239846	-0.927122	-0.000000
H	1.239846	0.927122	-0.000000
H	-1.239846	-0.927122	0.000000
H	-1.239846	0.927122	0.000000

TS: DA

E = −2012.25
H = −1922.51
G = -1946.06
N_{imag} = 1,-437.023i cm^{-1}

C	1.795040	-0.562353	-1.012840
C	1.568817	-1.655407	-0.196807
C	0.269694	-2.110477	0.105013
C	-0.861619	-1.492850	-0.395563
H	1.086496	0.261342	-1.048621
H	2.808866	-0.310416	-1.322323
H	2.408661	-2.307370	0.051503
H	0.168259	-3.092156	0.571990
H	-0.870293	-0.424000	-0.593751
H	-1.843228	-1.939741	-0.241238
C	-0.523015	-1.643271	-2.655920
C	0.753859	-1.195648	-2.953043
H	-1.381540	-0.996397	-2.828752
H	-0.738934	-2.708191	-2.630616
H	0.900574	-0.196317	-3.359442

H	1.549229	-1.906011	-3.162978

P: DA

E = −2069.63
H = −1976.62
G = −1998.81
N_{imag} = 0

C	-0.951522	-1.858615	-1.648114
C	-0.506018	-1.889319	-0.208769
C	0.724085	-1.540870	0.191950
C	1.791603	-1.035562	-0.744061
H	-1.521371	-2.772902	-1.877404
H	-1.666305	-1.028791	-1.792997
H	-1.236337	-2.221353	0.532873
H	0.982934	-1.615124	1.250783
H	2.573920	-1.807155	-0.858697
H	2.302767	-0.172519	-0.289002
C	1.221484	-0.651963	-2.117896
C	0.231821	-1.712826	-2.616687
H	2.036015	-0.509043	-2.842007
H	0.699989	0.314999	-2.034562
H	0.752722	-2.680313	-2.697635
H	-0.129302	-1.464362	-3.624687

Methylazide (MA)

E = −980.32
H = −946.27
G = −966.38
N_{imag} = 0

N	1.015329	0.131679	-0.273371
N	-0.100323	-0.031827	-0.068515
N	-1.315010	-0.050155	0.135430
C	-1.925762	-1.366644	0.397049
H	-2.992374	-1.190010	0.554899
H	-1.504530	-1.834744	1.298473
H	-1.799568	-2.046791	-0.457686

TS: 1,3-DC

E = −1695.19
H = −1627.05
G = −1651.37
N_{imag} = 1,−352.825i cm^{-1}

N	-1.485970	-2.552987	-0.134555
N	-2.611816	-2.201837	-0.156315
N	-3.261432	-1.128254	-0.306705
C	-4.598883	-1.000504	0.266278
H	-4.945052	0.011144	0.031453
H	-4.605148	-1.134112	1.359088
H	-5.299404	-1.714512	-0.188589
C	-0.516529	-0.639111	-0.154053
C	-1.570852	0.252683	-0.198837

H	0.034376	-0.884133	-1.059840
H	-0.008312	-0.849644	0.783855
H	-1.869395	0.716629	-1.135670
H	-1.918758	0.746562	0.707258

P: 1,3-DC

E = −1731.40

H = −1660.92

G = −1683.49

$\boldsymbol{N}_{\mathbf{imag}}$ = 0

N	-1.625729	-2.614444	-0.059777
N	-2.877926	-2.541265	-0.091621
N	-3.339505	-1.257176	-0.307133
C	-4.683886	-0.972897	0.158089
H	-5.113230	-0.147909	-0.423552
H	-4.703285	-0.699158	1.228824
H	-5.295320	-1.868416	0.009054
C	-1.044524	-1.247052	-0.257560
C	-2.240626	-0.308983	-0.067508
H	-0.638027	-1.208198	-1.279888
H	-0.219376	-1.091748	0.444721
H	-2.271789	0.519389	-0.783720
H	-2.302808	0.103872	0.957066

TS: ER

E = −1257.33

H = −1203.40

G = −1222.13

$\boldsymbol{N}_{\mathbf{imag}}$ = 1,-685.650i cm^{-1}

C	-1.036763	0.731313	0.040672
C	-0.626061	-0.571812	0.461060
C	0.724439	-0.607613	0.192022
C	1.105103	0.767499	0.104630
H	-0.909320	1.004804	-1.003423
H	-1.810072	1.311479	0.561112
H	-1.252704	-1.310616	0.964023
H	1.369022	-1.465474	-0.008273
H	0.967447	1.401314	0.976713
H	1.867781	1.133576	-0.595196

P: ER

E = −1288.69

H = −1233.13

G = −1251.86

$\boldsymbol{N}_{\mathbf{imag}}$ = 0

C	-0.744576	0.796404	-0.018731
C	-0.627835	-0.685103	0.292666
C	0.711221	-0.657539	0.415392
C	0.822078	0.828646	0.124928
H	-1.121168	1.048915	-1.020123
H	-1.288073	1.401704	0.720583

H	-1.373695	-1.474884	0.381256
H	1.460103	-1.416571	0.641083
H	1.196967	1.452791	0.948514
H	1.363849	1.100107	-0.792229

(*Z*)-penta-1,3-diene (P)
E = −1288.69
H = −1233.13
G = −1251.86
N_{imag} = 0

C	-1.846434	0.289405	-0.256294
C	-0.896129	1.162395	0.120879
C	0.550312	1.050670	-0.064716
C	1.319004	-0.058293	-0.053583
C	0.883022	-1.471700	0.180356
H	-2.898910	0.491875	-0.062462
H	-1.614949	-0.627321	-0.795872
H	-1.225307	2.092487	0.595085
H	1.069867	2.005839	-0.180676
H	2.391827	0.087759	-0.203298
H	1.625459	-2.011109	0.784682
H	0.791062	-2.026695	-0.767321
H	-0.090102	-1.522781	0.684818

TS: SR
E = −1647.45
H = −1576.99
G = −1597.75
N_{imag} = 1,−1362.402i cm^{-1}

C	-1.524305	-0.013290	-0.092896
C	-0.707176	1.143809	-0.163554
C	0.687645	1.087845	-0.249974
C	1.362888	-0.096270	0.063169
C	0.715241	-1.354894	0.152281
H	-2.514310	0.113744	0.356290
H	-1.553450	-0.696111	-0.944367
H	-1.137261	2.108786	0.117019
H	1.247423	2.023392	-0.244010
H	2.358788	0.014430	0.499974
H	1.208780	-2.116731	0.763872
H	0.267872	-1.787065	-0.745037
H	-0.579220	-0.888671	0.506002

Ethane (Et)
E = −924.59
H = −876.32
G = −892.58
N_{imag} = 0

C	0.000000	-0.000001	0.765249
C	0.000000	0.000001	-0.765249
H	0.000238	1.022102	1.166820

H	0.885040	-0.511253	1.166812
H	-0.885278	-0.510840	1.166811
H	0.000238	-1.022101	-1.166820
H	-0.885278	0.510840	-1.166812
H	0.885040	0.511253	-1.166812

TS: DGT

E = -1616.79

H = -1538.58

G = -1559.30

N_{imag} = 1,-1400.354i cm^{-1}

C	1.344203	0.711756	0.000000
C	1.344203	-0.711756	0.000000
C	-1.344203	-0.711756	0.000000
C	-1.344202	0.711756	0.000000
H	1.638170	1.233852	0.913072
H	1.638170	1.233852	-0.913072
H	-0.000000	0.989347	-0.000000
H	1.638170	-1.233852	0.913071
H	1.638170	-1.233852	-0.913071
H	0.000000	-0.989348	-0.000000
H	-1.638170	-1.233852	0.913071
H	-1.638170	-1.233852	-0.913071
H	-1.638170	1.233852	0.913072
H	-1.638170	1.233852	-0.913072

Table S8. Cartesian coordinates (in Å), energies (in kcal mol^{-1}), and number of imaginary frequencies of all stationary points, computed at BP86-D3(BJ)/QZ4P.

Butadiene (B)

E = −1303.14
H = −1247.85
G = −1267.81
N_{imag} = 0

C	1.367363	-0.713678	-0.499293
C	0.702298	-0.208608	0.550553
C	-0.702299	0.208608	0.550553
C	-1.367363	0.713678	-0.499292
H	0.875452	-0.885408	-1.457044
H	2.419020	-0.984786	-0.426394
H	1.226954	-0.114755	1.505330
H	-1.226954	0.114755	1.505330
H	-0.875452	0.885408	-1.457044
H	-2.419020	0.984786	-0.426394

Ethylene (E)

E = −732.38
H = −698.81
G = −714.44
N_{imag} = 0

C	0.666218	-0.000000	0.000000
C	-0.666218	-0.000000	0.000000
H	1.239598	-0.927165	0.000000
H	1.239598	0.927165	0.000000
H	-1.239598	-0.927165	0.000000
H	-1.239598	0.927165	0.000000

TS: DA

E = −2026.70
H = −1936.90
G = −1960.41
N_{imag} = 1,-421731i cm^{-1}

C	1.792034	-0.558643	-1.009401
C	1.568057	-1.656431	-0.202123
C	0.268798	-2.111612	0.099763
C	-0.860400	-1.487780	-0.392964
H	1.078021	0.259958	-1.046523
H	2.803747	-0.303468	-1.321531
H	2.407460	-2.311339	0.038053
H	0.166325	-3.096488	0.558781
H	-0.862866	-0.419907	-0.595302
H	-1.843445	-1.931339	-0.241420
C	-0.520899	-1.645680	-2.655588
C	0.754098	-1.198918	-2.952151
H	-1.379930	-0.999381	-2.825839
H	-0.735353	-2.710190	-2.619216
H	0.902843	-0.199488	-3.356629

H	1.552375	-1.908558	-3.151298

P: DA

E = −2082.77

H = −1989.69

G = −2011.87

N_{imag} = 0

C	1.910877	-1.071296	-1.531346
C	1.459560	-1.465053	-0.149622
C	0.191464	-1.771040	0.153627
C	-0.920521	-1.802000	-0.861509
H	2.596610	-0.212429	-1.464492
H	2.511047	-1.889240	-1.967968
H	2.218615	-1.490838	0.635233
H	-0.066920	-2.020193	1.185125
H	-1.594946	-0.943793	-0.692671
H	-1.542779	-2.695843	-0.699629
C	-0.387276	-1.778361	-2.300363
C	0.727705	-0.737266	-2.449856
H	-1.204790	-1.578638	-3.006792
H	0.014353	-2.772709	-2.552119
H	0.331045	0.254384	-2.180218
H	1.062444	-0.671411	-3.494315

Methylazide (MA)

E = −983.85

H = −949.79

G = −969.90

N_{imag} = 0

N	1.013554	0.1260680	-0.272376
N	-0.102777	-0.0320520	-0.068082
N	-1.317428	-0.0478320	0.135529
C	-1.925199	-1.3656240	0.396830
H	-2.992050	-1.1918320	0.555080
H	-1.501646	-1.8325870	1.297657
H	-1.796691	-2.0446340	-0.458360

TS: 1,3-DC

E = −1705.05

H = −1636.87

G = −1661.12

N_{imag} = 1,−346.860i cm^{-1}

N	-1.492502	-2.569506	-0.147701
N	-2.613619	-2.211131	-0.187799
N	-3.263994	-1.140557	-0.354275
C	-4.576587	-0.991858	0.267053
H	-4.924283	0.016918	0.023479
H	-4.538313	-1.102204	1.361687
H	-5.298182	-1.711041	-0.143928
C	-0.525688	-0.631857	-0.140571
C	-1.589620	0.245418	-0.199313

H	0.0418270	-0.87017	-1.03758
H	-0.0379240	-0.85123	0.80581
H	-1.8752610	0.71388	-1.13768
H	-1.9630290	0.72527	0.70418

P: 1,3-DC
E = −1740.90
H = −1670.37
G = −1692.93
N_{imag} = 0

N	-1.626377	-2.613068	-0.053768
N	-2.878228	-2.541497	-0.090872
N	-3.339462	-1.258195	-0.314538
C	-4.680387	-0.972717	0.157042
H	-5.108423	-0.143713	-0.419257
H	-4.692552	-0.703516	1.228905
H	-5.294662	-1.865832	0.007011
C	-1.046450	-1.246187	-0.256290
C	-2.243039	-0.309912	-0.069279
H	-0.641900	-1.210598	-1.279320
H	-0.221488	-1.087596	0.444951
H	-2.273068	0.519390	-0.783826
H	-2.309995	0.099456	0.956236

TS: ER
E = −1263.74
H = −1209.83
G = −1228.55
N_{imag} = 1,-675.325i cm^{-1}

C	-1.036692	0.732398	0.039873
C	-0.626090	-0.573051	0.459552
C	0.724490	-0.608211	0.193881
C	1.105001	0.768803	0.104973
H	-0.913258	1.003041	-1.005495
H	-1.805625	1.314749	0.563709
H	-1.253768	-1.313346	0.958587
H	1.370122	-1.466000	-0.002222
H	0.971405	1.400518	0.979266
H	1.863287	1.135569	-0.598783

P: ER
E = −1294.70
H = −1239.12
G = −1257.85
N_{imag} = 0

C	-0.744710	0.796654	-0.018795
C	-0.627907	-0.685209	0.292687
C	0.711293	-0.657645	0.415413
C	0.822211	0.828901	0.124884
H	-1.120734	1.048705	-1.020250
H	-1.287680	1.401593	0.720841

H	-1.373783	-1.474693	0.381228
H	1.460189	-1.416383	0.641040
H	1.196525	1.452640	0.948714
H	1.363466	1.099908	-0.792422

(*Z*)-penta-1,3-diene (P)
E = −1684.91
H = −1611.42
G = −1634.03
N_{imag} = 0

C	-1.829789	0.2747670	-0.256120
C	-0.890880	1.1612690	0.117288
C	0.555237	1.0568980	-0.062892
C	1.319911	-0.0541220	-0.050355
C	0.869268	-1.4627090	0.179720
H	-2.884979	0.4662460	-0.067460
H	-1.584760	-0.6421830	-0.788567
H	-1.229868	2.0910850	0.584257
H	1.074582	2.0120830	-0.175298
H	2.393867	0.0855790	-0.194960
H	1.618314	-2.0209890	0.757179
H	0.738814	-2.0043580	-0.770923
H	-0.090997	-1.5010350	0.709728

TS: SR
E = −1657.21
H = −1586.88
G = −160.66
N_{imag} = 1,−1423.633i cm^{-1}

C	-1.527803	-0.009236	-0.097551
C	-0.706411	1.144037	-0.164418
C	0.688081	1.086329	-0.247776
C	1.362645	-0.097565	0.065700
C	0.718792	-1.357369	0.152126
H	-2.520090	0.120631	0.344697
H	-1.548662	-0.696410	-0.945415
H	-1.134887	2.109915	0.114600
H	1.248178	2.021298	-0.239197
H	2.357906	0.013889	0.503186
H	1.214292	-2.120505	0.759735
H	0.264781	-1.784459	-0.743960
H	-0.583906	-0.891580	0.517039

Ethane (Et)
E = −927.44
H = −879.15
G = −895.42
N_{imag} = 0

C	0.000000	-0.000001	0.765090
C	0.000000	0.000001	-0.765090
H	0.000031	1.022145	1.166307

H	-0.885212	-0.511040	1.166299
H	0.885180	-0.511096	1.166299
H	0.000032	-1.022144	-1.166307
H	-0.885212	0.511041	-1.166299
H	0.885180	0.511096	-1.166299

TS: DGT

E = -1626.16

H = -1545.45

G = -1567.88

N_{imag} = 1,-1533.880i cm^{-1}

C	1.346261	0.711421	0.000000
C	1.346261	-0.711421	0.000000
C	-1.346261	-0.711421	0.000000
C	-1.346260	0.711421	0.000000
H	1.637957	1.233686	0.913453
H	1.637957	1.233686	-0.913453
H	-0.000000	0.990912	0.000000
H	1.637957	-1.233686	0.913453
H	1.637957	-1.233686	-0.913453
H	-0.000000	-0.990912	-0.000000
H	-1.637957	-1.233686	0.913453
H	-1.637957	-1.233686	-0.913453
H	-1.637957	1.233686	0.913453
H	-1.637957	1.233686	-0.913453

Table S9. Global-minimum Cartesian root-mean-square deviation (RMSD) values (in Å) of the fully optimized geometries at various DFT levels of theory with respect to the CCSD(T)/cc-pVTZ benchmarks.

	BP86/DZ	BP86/DZP	BP86/TZ2P	BP86/QZ4P	BP86-D3(BJ)/QZ4P
**B**	0.037158	0.024089	0.049918	0.044318	0.039359
**E**	0.009316	0.005545	0.005675	0.005727	0.005574
**TS-DA**	0.037869	0.028260	0.031486	0.032080	0.026029
**P-DA**	0.018495	0.018281	0.027482	0.027223	0.020090
**MA**	0.079993	0.030022	0.038587	0.038218	0.034785
**TS-1,3-DC**	0.261398	0.103238	0.142519	0.150162	0.114478
**P-1,3-DC**	0.146403	0.046991	0.067095	0.070897	0.065678
**TS-ER**	0.024240	0.008738	0.006690	0.006966	0.007983
**P-ER**	0.016836	0.006537	0.006435	0.006230	0.005990
**P**	0.027782	0.037024	0.087841	0.078892	0.081561
**TS-SR**	0.033426	0.020593	0.018638	0.018828	0.018096
**Et**	0.011221	0.006931	0.007228	0.007463	0.007091
**TS-DGT**	0.025224	0.010974	0.011855	0.011501	0.012013
AVG[a]	0.056105	0.026709	0.038573	0.038347	0.033748
MAX[a]	0.261398	0.103238	0.142519	0.150162	0.114478

[a] AVG = average global-minimum Cartesian root-mean-square deviation; MAX = maximum global-minimum Cartesian root-mean-square deviation.

Table S10. Average percentage errors of all geometric parameters of the fully optimized DFT geometries with respect to the CCSD(T)/cc-pVTZ benchmarks.

	Functional	Basis set	**B**	**E**	**TS-DA**	**P-DA**	**MA**	**TS-1,3-DC**	**P-1,3-DC**	**TS-ER**	**P-ER**[c]	**P**	**TS-SR**	**Et**	**TS-DGT**	AVG[a]	MAX[a]
**DISTANCES**	BP86	DZ	0.6	0.5	0.9	0.8	1.5	1.4	1.3	0.7	1.0	0.7	0.8	0.9	0.9	0.9	1.5
		DZP	0.6	0.5	0.7	0.5	0.5	0.7	0.6	0.4	0.4	0.6	0.5	0.3	0.5	0.5	0.7
		TZ2P	0.5	0.5	0.7	0.4	0.5	0.8	0.6	0.4	0.4	0.6	0.5	0.4	0.5	0.5	0.8
		QZ4P	0.5	0.5	0.7	0.4	0.5	0.8	0.6	0.4	0.4	0.5	0.4	0.3	0.4	0.5	0.8
	BP86-D3(BJ)	QZ4P	0.5	0.5	0.7	0.4	0.5	0.8	0.6	0.4	0.4	0.5	0.5	0.3	0.5	0.5	0.8
**ANGLES**	BP86	DZ	0.4	0.3	0.3	0.2	1.5	0.8	1.1	0.2	0.3	0.2	0.3	0.1	0.6	0.5	1.5
		DZP	0.3	0.2	0.2	0.2	0.6	0.5	0.5	0.1	0.1	0.4	0.3	0.1	0.4	0.3	0.6
		TZ2P	0.6	0.3	0.3	0.3	0.7	0.8	0.7	0.2	0.2	0.6	0.3	0.2	0.3	0.4	0.8
		QZ4P	0.6	0.4	0.3	0.3	0.7	0.8	0.7	0.2	0.2	0.6	0.3	0.2	0.3	0.4	0.8
	BP86-D3(BJ)	QZ4P	0.5	0.4	0.3	0.2	0.6	0.7	0.7	0.2	0.2	0.5	0.3	0.2	0.3	0.4	0.7
**DIHEDRALS**	BP86	DZ	11.3	–	0.9	2.8	0.2	14.5	21.4	1.3	0.1	18.3	1.9	0.0	0.9	6.1	21.4
		DZP	4.4	–	0.8	2.4	0.1	12.9	5.8	1.0	0.0	8.3	1.3	0.0	0.1	3.1	12.9
		TZ2P	4.3	–	1.2	2.9	0.1	28.3	9.6	0.9	0.0	15.8	1.1	0.0	0.1	5.4	28.3
		QZ4P	3.0	–	1.2	3.1	0.1	32.6	9.8	0.9	0.0	14.6	1.2	0.0	0.1	5.6	32.6
	BP86-D3(BJ)	QZ4P	3.2	–	1.1	3.4	0.1	17.6	9.2	1.2	0.0	18.3	1.0	0.0	0.1	4.6	18.3

[a] AVG = total average percentage error; MAX = maximum percentage error.

Table S11. Errors in reaction barriers and reaction energies (kcal mol^{-1}) for the various pericyclic reactions, computed with XC/QZ4P//CCSD(T)/cc-pVTZ, compared to CCSDT(Q)/CBS//CCSD(T)/cc-pVTZ.

XC	Diels-Alder Reaction		1,3-dipolar Cycloaddition		Electrocyclic Rearrangement		Sigmatropic Rearrangement		Double Group Transfer	
	$\Delta E^{\ddagger}$	ΔE_{rxn}	$\Delta E^{\ddagger}$	ΔE_{rxn}	$\Delta E^{\ddagger}$	ΔE_{rxn}	$\Delta E^{\ddagger}$	ΔE_{rxn}	$\Delta E^{\ddagger}$	ΔE_{rxn}
LDA										
VWN	–20.1	–17.8	–15.8	–13.4	–7.1	–6.1	–15.1	0.0	–28.3	0.0
GGAs										
BP86	–4.9	5.4	–2.7	8.5	–3.9	–0.3	–8.7	0.0	–12.1	0.0
BLYP	1.7	17.6	1.5	18.3	–0.8	5.3	–5.1	0.0	–4.9	0.0
BEE	–3.9	4.6	–0.9	9.3	–4.6	–2.3	–8.9	0.0	–11.8	0.0
PW91	–7.6	1.4	–4.9	5.1	–4.2	–1.2	–9.2	0.0	–14.5	0.0
PBE	–7.2	1.3	–4.3	5.5	–4.5	–1.9	–9.4	0.0	–14.4	0.0
PBEsol	–13.8	–9.4	–9.8	–4.6	–6.8	–5.3	–13.0	0.0	–21.9	0.0
RPBE	–0.9	9.6	1.2	13.6	–3.6	–0.4	–7.1	0.0	–8.5	0.0
revPBE	–1.3	8.8	1.1	13.0	–3.8	–0.7	–7.5	0.0	–9.0	0.0
mPBE	–5.5	3.6	–2.8	7.7	–4.2	–1.4	–8.7	0.0	–12.8	0.0
mPW	–4.4	5.8	–2.0	9.5	–3.6	–0.3	–8.1	0.0	–11.3	0.0
HTBS	–6.4	0.4	–2.8	5.2	–6.0	–3.9	–10.9	0.0	–14.7	0.0
OLYP	4.2	11.6	6.0	15.7	–3.1	–1.5	–6.2	0.0	–4.9	0.0
OPBE	–1.1	–1.1	3.7	6.8	–6.8	–8.6	–10.1	0.0	–11.4	0.0
XLYP	2.1	18.8	1.6	19.2	–0.4	6.1	–4.4	0.0	–4.1	0.0
Meta-GGAs										
M06-L	–2.4	1.5	2.4	12.0	0.2	–2.6	–2.9	0.0	–4.3	0.0
MVS	–7.4	–6.9	0.2	4.1	–0.7	–2.6	–8.6	0.0	–13.6	0.0
TPSS	–4.2	7.4	–2.1	9.4	–3.3	–0.7	–6.0	0.0	–7.2	0.0
revTPSS	–5.2	5.3	–3.1	6.2	–3.8	–2.4	–5.4	0.0	–5.3	0.0
Hybrids										
B3LYP	3.2	10.1	3.7	10.3	1.4	3.3	–1.8	0.0	–0.6	0.0
B3LYP*	1.2	9.4	1.8	9.9	0.3	3.0	–3.4	0.0	–3.5	0.0
B1LYP	4.7	10.4	5.0	9.9	2.2	3.6	–0.4	0.0	1.8	0.0
B1PW91	–0.3	–1.1	2.9	2.2	–1.0	–2.8	–3.8	0.0	–4.1	0.0
BHandH	–7.3	–19.1	–4.1	–19.5	0.8	–4.9	–2.7	0.0	–7.8	0.0
BHandHLYP	7.6	3.1	8.3	1.1	5.2	1.9	4.4	0.0	8.3	0.0
KMLYP	–0.6	–12.9	2.2	–13.5	3.1	–3.4	1.2	0.0	–0.1	0.0
O3LYP	–22.9	–28.5	–17.9	–24.6	–6.1	–8.3	–14.1	0.0	–28.7	0.0
OPBE0	0.9	–7.0	5.9	–0.9	–3.4	–8.8	–5.3	0.0	–5.1	0.0
PBE0	–3.5	–5.2	–0.1	–1.9	–1.6	–3.7	–4.7	0.0	–7.3	0.0
mPW1PW	–1.2	–1.5	1.8	1.4	–0.8	–2.3	–3.6	0.0	–4.6	0.0
mPW1K	1.0	–6.6	4.4	–4.7	1.2	–3.8	–0.3	0.0	0.0	0.0
S12H	–3.4	–5.8	1.0	–0.2	–0.7	–4.0	–2.8	0.0	–6.2	0.0
X3LYP	2.4	8.5	3.0	8.4	1.4	3.1	–1.7	0.0	–1.0	0.0
Meta-Hybrids										
M06	–0.1	–0.3	4.4	6.2	0.1	–1.5	–1.9	0.0	–1.8	0.0
M06-2X	–1.8	–1.2	2.4	–0.8	1.1	–0.5	–0.2	0.0	–1.2	0.0
M06-HF	–5.8	–0.8	–1.2	–11.8	–0.5	1.1	0.9	0.0	0.5	0.0
TPSSH	–2.9	4.4	–0.5	6.3	–2.2	–1.7	–4.4	0.0	–5.0	0.0
Double-Hybrids										
B2K-PLYP	–3.6	–1.6	–0.7	3.1	0.7	0.1	0.6	0.0	–1.0	0.0
B2T-PLYP	–1.9	2.1	0.2	5.7	0.7	1.1	–0.1	0.0	–1.0	0.0
B2-PLYP	–1.7	3.8	0.1	7.7	0.3	1.6	–0.9	0.0	–1.7	0.0
LS1-TPSS	–9.0	–9.1	–3.9	–1.4	–1.5	–3.7	–0.5	0.0	–4.6	0.0
mPW2K-PLYP	–3.9	–1.8	–1.1	2.8	0.7	0.3	0.7	0.0	–1.1	0.0
mPW2-PLYP	–1.2	2.9	0.5	5.8	1.2	1.8	0.1	0.0	–0.6	0.0
PBE0-DH	–3.7	–9.2	0.4	–4.9	–0.6	–4.5	–2.0	0.0	–4.3	0.0
revDSD-BLYP	–4.4	–1.7	–1.5	3.1	0.9	0.6	1.5	0.0	–0.5	0.0
revDSD-PBE	–5.1	–4.5	–1.5	1.5	–0.3	–1.8	0.5	0.0	–2.4	0.0
revDSD-PBEP86	–2.8	–1.8	0.3	3.7	0.5	–0.9	1.6	0.0	–0.2	0.0
Range-Separated Hybrids										
CAM-B3LYP	4.1	2.3	3.8	1.1	2.6	0.8	0.5	0.0	1.5	0.0
CAMY-B3LYP	2.1	3.2	2.4	2.6	1.9	1.5	–0.7	0.0	–0.5	0.0
ωB97	4.1	–8.5	4.3	–6.3	3.1	–5.3	3.6	0.0	2.8	0.0
ωB97X	3.4	–4.7	3.9	–3.4	2.6	–3.1	2.0	0.0	1.5	0.0
ωB97X-D	3.5	–0.1	4.7	1.0	1.3	–2.2	–0.4	0.0	0.0	0.0

Table S11 (continued).

XC	Diels-Alder Reaction		1,3-dipolar Cycloaddition		Electrocyclic Rearrangement		Sigmatropic Rearrangement		Double Group Transfer	
	$\Delta E^{\ddagger}$	ΔE_{rxn}	$\Delta E^{\ddagger}$	ΔE_{rxn}	$\Delta E^{\ddagger}$	ΔE_{rxn}	$\Delta E^{\ddagger}$	ΔE_{rxn}	$\Delta E^{\ddagger}$	ΔE_{rxn}
					Dispersion-Corrected					
BP86-D3(BJ)	–11.2	0.4	–7.1	4.5	–4.1	0.0	–9.2	0.0	–15.5	0.0
BLYP-D3(BJ)	–6.4	11.2	–4.2	13.0	–1.2	5.4	–5.8	0.0	–9.2	0.0
PBE-D3(BJ)	–11.2	–1.9	–7.2	2.9	–4.7	–1.8	–9.7	0.0	–16.7	0.0
OLYP-D3(BJ)	–12.8	–2.9	–6.4	3.1	–4.5	–1.7	–9.4	0.0	–14.1	0.0
OPBE-D3(BJ)	–17.9	–15.0	–8.4	–5.2	–8.0	–8.6	–12.8	0.0	–20.5	0.0
B3LYP-D3(BJ)	–3.4	4.9	–1.0	6.0	1.0	3.4	–2.4	0.0	–4.2	0.0
PBE0-D3(BJ)	–7.0	–7.9	–2.6	–4.2	–1.7	–3.6	–5.0	0.0	–9.3	0.0
M06-2X-D3	–2.0	–1.3	2.3	–0.8	1.1	–0.5	–0.2	0.0	–1.3	0.0

Table S12. Reaction barriers and reaction energies (kcal mol^{-1}) for the various pericyclic reactions, computed at DFT level using Method/BS, where Method = BP86, BP86-D3(BJ) and BS = DZ, DZP, TZ2P, QZ4P.

Method/BS	Diels-Alder Reaction		1,3-dipolar Cycloaddition		Electrocyclic rearrangement		Sigmatropic rearrangement		Double Group Transfer	
	$\Delta E^{\ddagger}$	ΔE_{rxn}	$\Delta E^{\ddagger}$	ΔE_{rxn}	$\Delta E^{\ddagger}$	ΔE_{rxn}	$\Delta E^{\ddagger}$	ΔE_{rxn}	$\Delta E^{\ddagger}$	ΔE_{rxn}
BP86/DZ	12.8	–42.3	6.1	–22.5	41.7	14.0	30.8	0.0	39.4	0.0
BP86/DZP	13.3	–42.4	12.4	–20.9	39.9	10.0	28.8	0.0	38.0	0.0
BP86/TZ2P	15.2	–41.6	15.5	–20.1	39.9	8.8	28.2	0.0	38.1	0.0
BP86/QZ4P	15.0	–42.4	15.4	–20.8	39.6	8.3	28.2	0.0	38.2	0.0
BP86-D3(BJ)/QZ4P	8.8	–47.2	11.2	–24.7	39.4	8.4	27.7	0.0	33.7	0.0

Table S13. Reaction barriers and reaction energies (kcal mol^{-1}) for the various pericyclic reactions, computed with XC/TZ2P//CCSD(T)/cc-pVTZ.

XC	Diels-Alder Reaction		1,3-dipolar Cycloaddition		Electrocyclic Rearrangement		Sigmatropic Rearrangement		Double Group Transfer	
	$\Delta E^{\ddagger}$	ΔE_{rxn}	$\Delta E^{\ddagger}$	ΔE_{rxn}	$\Delta E^{\ddagger}$	ΔE_{rxn}	$\Delta E^{\ddagger}$	ΔE_{rxn}	$\Delta E^{\ddagger}$	ΔE_{rxn}
LDA										
VWN	–0.2	–64.8	2.7	–41.6	36.6	2.9	21.7	0.0	21.8	0.0
GGAs										
BP86	14.9	–41.7	15.7	–19.8	39.8	8.7	28.1	0.0	38.1	0.0
BLYP	21.5	–29.6	19.9	–10.2	42.8	14.2	31.8	0.0	45.3	0.0
BEE	16.0	–42.3	17.6	–18.7	39.2	7.0	27.9	0.0	38.4	0.0
PW91	12.2	–45.6	13.5	–23.1	39.5	7.9	27.7	0.0	35.8	0.0
PBE	12.6	–45.7	14.1	–22.7	39.2	7.3	27.5	0.0	35.8	0.0
PBEsol	6.0	–56.4	8.7	–32.7	37.0	3.8	23.8	0.0	28.3	0.0
RPBE	18.9	–37.3	19.7	–14.5	40.3	8.8	29.8	0.0	41.9	0.0
revPBE	18.5	–38.1	19.5	–15.1	40.0	8.4	29.3	0.0	41.3	0.0
mPBE	14.3	–43.3	15.6	–20.5	39.6	7.8	28.1	0.0	37.4	0.0
mPW	15.5	–41.3	16.5	–18.7	40.1	8.8	28.7	0.0	39.0	0.0
HTBS	13.5	–46.4	15.8	–22.7	37.8	5.3	26.0	0.0	35.5	0.0
OLYP	24.0	–35.2	24.6	–12.3	40.8	7.8	30.7	0.0	45.5	0.0
OPBE	18.8	–47.6	22.4	–20.7	37.3	1.1	26.8	0.0	38.9	0.0
XLYP	21.6	–28.3	19.9	–9.3	43.3	15.0	32.4	0.0	46.1	0.0
Meta-GGAs										
M06-L	17.1	–45.1	20.4	–16.5	43.8	6.9	34.1	0.0	46.0	0.0
MVS	11.6	–54.6	17.9	–24.9	43.1	6.5	28.1	0.0	36.5	0.0
TPSS	15.6	–39.6	16.3	–18.8	40.5	8.4	30.8	0.0	43.0	0.0
revTPSS	14.5	–41.8	15.3	–22.1	40.0	6.9	31.3	0.0	44.9	0.0
Hybrids										
B3LYP	22.8	–37.3	22.0	–18.3	45.0	12.1	35.0	0.0	49.5	0.0
B3LYP*	20.8	–37.9	20.1	–18.6	44.0	11.8	33.4	0.0	46.7	0.0
B1LYP	24.3	–37.1	23.2	–18.8	45.9	12.4	36.3	0.0	51.8	0.0
B1PW91	19.3	–48.3	21.1	–26.2	42.7	6.2	32.9	0.0	45.9	0.0
BHandH	12.1	–66.9	14.0	–48.2	44.5	3.9	34.0	0.0	42.2	0.0
BHandHLYP	26.9	–44.7	26.3	–27.7	48.8	10.6	41.1	0.0	58.3	0.0
KMLYP	18.6	–60.8	20.2	–42.3	46.8	5.4	37.8	0.0	49.9	0.0
O3LYP	–3.2	–75.8	0.4	–52.9	37.6	0.7	22.6	0.0	21.4	0.0
OPBE0	20.6	–53.9	24.4	–28.8	40.6	0.6	31.5	0.0	45.1	0.0
PBE0	16.1	–52.4	18.2	–30.3	42.2	5.4	32.0	0.0	42.8	0.0
mPW1PW	18.4	–48.8	20.1	–27.1	43.0	6.7	33.1	0.0	45.5	0.0
mPW1K	20.4	–54.2	22.6	–33.2	44.9	5.2	36.4	0.0	50.0	0.0
S12H	16.2	–52.7	19.2	–28.4	43.1	5.3	34.0	0.0	44.1	0.0
X3LYP	22.0	–38.9	21.2	–20.2	45.1	11.9	35.1	0.0	49.1	0.0
Meta-Hybrids										
M06	19.5	–47.7	22.6	–22.3	43.7	7.4	34.9	0.0	48.3	0.0
M06-2X	17.5	–49.2	20.5	–29.8	44.7	8.2	36.4	0.0	48.7	0.0
M06-HF	14.3	–49.0	17.7	–41.1	43.1	9.4	37.7	0.0	50.9	0.0
TPSSH	16.8	–42.7	17.9	–22.0	41.6	7.6	32.4	0.0	45.2	0.0
Double-Hybrids										
B2K-PLYP	18.1	–49.0	18.4	–25.4	44.9	9.1	35.7	0.0	48.9	0.0
B2T-PLYP	19.2	–45.7	19.1	–22.9	44.8	10.0	35.4	0.0	48.9	0.0
B2-PLYP	19.2	–43.6	19.0	–20.9	44.4	10.5	34.8	0.0	48.2	0.0
LS1-TPSS	13.8	–56.2	15.7	–29.7	43.1	5.6	33.8	0.0	45.2	0.0
mPW2K-PLYP	17.8	–49.1	18.0	–25.7	45.0	9.2	35.8	0.0	48.7	0.0
mPW2-PLYP	19.6	–44.7	19.3	–22.8	45.2	10.7	35.8	0.0	49.4	0.0
PBE0-DH	16.5	–56.7	18.9	–33.5	43.3	4.5	34.1	0.0	45.7	0.0
revDSD-BLYP	17.1	–49.5	17.5	–25.9	45.1	9.4	36.7	0.0	49.1	0.0
revDSD-PBE	16.3	–52.4	17.3	–27.5	43.9	7.2	35.9	0.0	47.1	0.0
revDSD-PBEP86	18.6	–49.7	19.1	–25.3	44.7	8.1	36.9	0.0	49.3	0.0
Range-Separated Hybrids										
CAM-B3LYP	23.6	–45.3	21.9	–27.6	46.2	9.6	37.2	0.0	51.5	0.0
CAMY-B3LYP	21.6	–44.3	20.5	–26.1	45.6	10.3	36.0	0.0	49.6	0.0
ωB97	23.5	–55.6	22.4	–34.8	46.8	4.1	40.4	0.0	53.1	0.0
ωB97X	22.8	–52.0	21.9	–32.0	46.3	6.1	38.8	0.0	51.6	0.0
ωB97X-D	23.0	–47.6	22.8	–27.6	45.0	6.7	36.4	0.0	50.2	0.0

Table S13 (continued).

XC	Diels-Alder Reaction		1,3-dipolar Cycloaddition		Electrocyclic Rearrangement		Sigmatropic Rearrangement		Double Group Transfer	
	$\Delta E^{\ddagger}$	ΔE_{rxn}	$\Delta E^{\ddagger}$	ΔE_{rxn}	$\Delta E^{\ddagger}$	ΔE_{rxn}	$\Delta E^{\ddagger}$	ΔE_{rxn}	$\Delta E^{\ddagger}$	ΔE_{rxn}
Dispersion-Corrected										
BP86-D3(BJ)	8.7	–46.6	11.2	–23.8	39.6	8.9	27.6	0.0	34.7	0.0
BLYP-D3(BJ)	13.4	–36.0	14.1	–15.5	42.4	14.3	31.0	0.0	41.0	0.0
PBE-D3(BJ)	8.6	–48.8	11.3	–25.3	39.1	7.4	27.1	0.0	33.6	0.0
OLYP-D3(BJ)	7.0	–49.6	12.1	–24.9	39.5	7.7	27.6	0.0	36.2	0.0
OPBE-D3(BJ)	2.1	–61.5	10.3	–32.7	36.1	1.0	24.1	0.0	29.9	0.0
B3LYP-D3(BJ)	16.2	–42.5	17.3	–22.6	44.7	12.2	34.4	0.0	45.9	0.0
PBE0-D3(BJ)	12.5	–55.2	15.7	–32.5	42.1	5.5	31.8	0.0	40.9	0.0
M06-2X-D3	17.4	–49.3	20.4	–29.9	44.7	8.2	36.4	0.0	48.6	0.0

Table S14. Errors in reaction barriers and reaction energies (kcal mol^{-1}) for the various pericyclic reactions, computed with XC/TZ2P//CCSD(T)/cc-pVTZ, compared to CCSDT(Q)/CBS//CCSD(T)/cc-pVTZ.

XC	Diels-Alder Reaction		1,3-dipolar Cycloaddition		Electrocyclic Rearrangement		Sigmatropic Rearrangement		Double Group Transfer	
	$\Delta E^{\ddagger}$	ΔE_{rxn}	$\Delta E^{\ddagger}$	ΔE_{rxn}	$\Delta E^{\ddagger}$	ΔE_{rxn}	$\Delta E^{\ddagger}$	ΔE_{rxn}	$\Delta E^{\ddagger}$	ΔE_{rxn}
LDA										
VWN	–19.8	–17.2	–15.6	–12.7	–6.8	–5.6	–15.1	0.0	–28.4	0.0
GGAs										
BP86	–4.7	6.0	–2.6	9.1	–3.6	0.3	–8.7	0.0	–12.1	0.0
BLYP	1.9	18.1	1.6	18.7	–0.6	5.8	–5.0	0.0	–4.9	0.0
BEE	–3.6	5.4	–0.7	10.2	–4.2	–1.5	–8.9	0.0	–11.8	0.0
PW91	–7.4	2.1	–4.8	5.8	–3.9	–0.5	–9.1	0.0	–14.4	0.0
PBE	–7.0	2.0	–4.2	6.2	–4.2	–1.2	–9.3	0.0	–14.4	0.0
PBEsol	–13.6	–8.8	–9.6	–3.8	–6.4	–4.7	–13.0	0.0	–21.9	0.0
RPBE	–0.7	10.4	1.4	14.4	–3.1	0.4	–7.0	0.0	–8.3	0.0
revPBE	–1.1	9.6	1.2	13.8	–3.4	0.0	–7.5	0.0	–8.9	0.0
mPBE	–5.3	4.4	–2.7	8.4	–3.8	–0.6	–8.7	0.0	–12.8	0.0
mPW	–4.1	6.4	–1.8	10.2	–3.3	0.4	–8.1	0.0	–11.2	0.0
HTBS	–6.1	1.3	–2.5	6.2	–5.6	–3.2	–10.8	0.0	–14.7	0.0
OLYP	4.4	12.5	6.3	16.6	–2.6	–0.6	–6.1	0.0	–4.7	0.0
OPBE	–0.8	0.0	4.1	8.2	–6.1	–7.4	–10.0	0.0	–11.3	0.0
XLYP	2.0	19.4	1.6	19.6	–0.1	6.6	–4.4	0.0	–4.1	0.0
Meta-GGAs										
M06-L	–2.5	2.6	2.1	12.4	0.4	–1.6	–2.7	0.0	–4.2	0.0
MVS	–8.0	–7.0	–0.4	4.0	–0.3	–2.0	–8.7	0.0	–13.7	0.0
TPSS	–4.0	8.1	–2.0	10.1	–2.9	0.0	–6.0	0.0	–7.2	0.0
revTPSS	–5.1	5.9	–3.0	6.8	–3.4	–1.6	–5.5	0.0	–5.3	0.0
Hybrids										
B3LYP	3.2	10.4	3.7	10.6	1.6	3.7	–1.8	0.0	–0.7	0.0
B3LYP*	1.2	9.8	1.8	10.3	0.6	3.4	–3.4	0.0	–3.5	0.0
B1LYP	4.7	10.6	4.9	10.1	2.5	4.0	–0.5	0.0	1.6	0.0
B1PW91	–0.3	–0.6	2.8	2.7	–0.7	–2.3	–3.9	0.0	–4.3	0.0
BHandH	–7.5	–19.3	–4.3	–19.3	1.1	–4.6	–2.8	0.0	–8.0	0.0
BHandHLYP	7.3	3.0	8.0	1.2	5.4	2.2	4.3	0.0	8.1	0.0
KMLYP	–1.0	–13.2	1.9	–13.4	3.4	–3.1	1.0	0.0	–0.3	0.0
O3LYP	–22.8	–28.2	–17.9	–24.0	–5.8	–7.8	–14.2	0.0	–28.8	0.0
OPBE0	1.0	–6.3	6.1	0.1	–2.8	–7.9	–5.3	0.0	–5.1	0.0
PBE0	–3.5	–4.8	–0.1	–1.4	–1.2	–3.1	–4.8	0.0	–7.4	0.0
mPW1PW	–1.2	–1.2	1.8	1.8	–0.4	–1.8	–3.7	0.0	–4.7	0.0
mPW1K	0.8	–6.6	4.3	–4.3	1.5	–3.3	–0.4	0.0	–0.2	0.0
S12H	–3.4	–5.1	0.9	0.5	–0.3	–3.2	–2.8	0.0	–6.1	0.0
X3LYP	2.4	8.8	2.9	8.7	1.7	3.5	–1.7	0.0	–1.1	0.0
Meta-Hybrids										
M06	–0.1	–0.1	4.3	6.6	0.3	–1.1	–1.9	0.0	–1.9	0.0
M06-2X	–2.1	–1.6	2.2	–0.9	1.3	–0.3	–0.4	0.0	–1.5	0.0
M06-HF	–5.3	–1.4	–0.6	–12.2	–0.3	1.0	0.9	0.0	0.7	0.0
TPSSH	–2.8	5.0	–0.4	6.9	–1.8	–0.9	–4.4	0.0	–5.0	0.0
Double-Hybrids										
B2K-PLYP	–1.5	–1.4	0.1	3.5	1.5	0.7	–1.1	0.0	–1.3	0.0
B2T-PLYP	–0.4	2.0	0.8	6.0	1.4	1.6	–1.4	0.0	–1.3	0.0
B2-PLYP	–0.4	4.1	0.7	8.0	1.0	2.1	–2.0	0.0	–2.0	0.0
LS1-TPSS	–5.8	–8.6	–2.6	–0.8	–0.3	–2.9	–3.0	0.0	–5.0	0.0
mPW2K-PLYP	–1.8	–1.5	–0.3	3.2	1.6	0.8	–1.0	0.0	–1.5	0.0
mPW2-PLYP	0.0	3.0	1.0	6.1	1.8	2.3	–1.0	0.0	–0.8	0.0
PBE0-DH	–3.1	–9.1	0.6	–4.6	–0.1	–4.0	–2.7	0.0	–4.5	0.0
revDSD-BLYP	–2.5	–1.9	–0.8	3.0	1.7	1.0	–0.1	0.0	–1.1	0.0
revDSD-PBE	–3.3	–4.8	–1.0	1.4	0.5	–1.3	–0.9	0.0	–3.1	0.0
revDSD-PBEP86	–1.0	–2.1	0.8	3.6	1.3	–0.4	0.1	0.0	–0.9	0.0
Range-Separated Hybrids										
CAM-B3LYP	4.0	2.4	3.6	1.3	2.8	1.2	0.4	0.0	1.3	0.0
CAMY-B3LYP	2.0	3.4	2.2	2.8	2.2	1.9	-0.8	0.0	-0.6	0.0
ωB97	3.9	–8.0	4.1	–5.9	3.4	–4.4	3.6	0.0	2.9	0.0
ωB97X	3.2	–4.4	3.6	–3.1	2.9	–2.4	2.0	0.0	1.4	0.0
ωB97X-D	3.4	0.0	4.5	1.3	1.6	–1.8	–0.4	0.0	0.0	0.0

Table S14 (continued).

XC	Diels-Alder Reaction		1,3-dipolar Cycloaddition		Electrocyclic Rearrangement		Sigmatropic Rearrangement		Double Group Transfer	
	$\Delta E^{‡}$	ΔE_{rxn}	$\Delta E^{‡}$	ΔE_{rxn}	$\Delta E^{‡}$	ΔE_{rxn}	$\Delta E^{‡}$	ΔE_{rxn}	$\Delta E^{‡}$	ΔE_{rxn}
Dispersion-Corrected										
BP86-D3(BJ)	–10.9	1.1	–7.1	5.1	–3.8	0.5	–9.2	0.0	–15.5	0.0
BLYP-D3(BJ)	–6.2	11.7	–4.2	13.4	–1.0	5.9	–5.8	0.0	–9.2	0.0
PBE-D3(BJ)	–11.0	–1.2	–7.0	3.6	–4.3	–1.1	–9.7	0.0	–16.6	0.0
OLYP-D3(BJ)	–12.6	–2.0	–6.2	4.0	–3.9	–0.7	–9.2	0.0	–14.0	0.0
OPBE-D3(BJ)	–17.5	–13.9	–8.0	–3.8	–7.3	–7.5	–12.7	0.0	–20.3	0.0
B3LYP-D3(BJ)	–3.4	5.2	–1.0	6.3	1.3	3.8	–2.4	0.0	–4.3	0.0
PBE0-D3(BJ)	–7.1	–7.6	–2.6	–3.6	–1.3	–3.0	–5.0	0.0	–9.3	0.0
M06-2X-D3	–2.2	–1.7	2.1	–1.0	1.3	–0.3	–0.4	0.0	–1.6	0.0

Table S15. Mean error (ME), mean absolute error (MAE), maximum unsigned error (MUE) and standard deviation (SD) of reaction barriers and energies (kcal mol^{-1}) for the various pericyclic reactions, computed with XC/TZ2P//CCSD(T)/cc-pVTZ compared to CCSDT(Q)/CBS//CCSD(T)/cc-pVTZ.

XC	$\Delta E^{\ddagger}$				ΔE_{rxn}				Total			
	ME	MAE	MUE	SD	ME	MAE	MUE	SD	ME	MAE	MUE	SD
						LDA						
VWN	–17.1	17.1	28.4	7.0	–11.8	11.8	17.2	4.8	–15.1	15.1	28.4	6.8
						GGAs						
BP86	–6.3	6.3	12.1	3.6	5.1	5.1	9.1	3.6	–2.1	5.9	12.1	3.6
BLYP	–1.4	2.8	5.0	1.8	14.2	14.2	18.7	5.9	4.4	7.1	18.7	6.7
BEE	–5.8	5.8	11.8	4.0	4.7	5.7	10.2	3.6	–1.9	5.8	11.8	3.8
PW91	–7.9	7.9	14.4	3.7	2.4	2.8	5.8	2.2	–4.0	6.0	14.4	4.1
PBE	–7.8	7.8	14.4	3.8	2.3	3.1	6.2	2.2	–4.0	6.0	14.4	4.0
PBEsol	–12.9	12.9	21.9	5.2	–5.7	5.7	8.8	2.1	–10.2	10.2	21.9	5.5
RPBE	–3.5	4.1	8.3	3.0	8.4	8.4	14.4	5.9	0.9	5.7	14.4	4.8
revPBE	–3.9	4.4	8.9	3.2	7.8	7.8	13.8	5.7	0.4	5.7	13.8	4.6
mPBE	–6.7	6.7	12.8	3.7	4.0	4.5	8.4	3.1	–2.7	5.8	12.8	3.7
mPW	–5.7	5.7	11.2	3.5	5.6	5.6	10.2	4.0	–1.5	5.7	11.2	3.7
HTBS	–7.9	7.9	14.7	4.3	1.4	3.5	6.2	2.0	–4.4	6.3	14.7	4.2
OLYP	–0.5	4.8	6.3	1.3	9.5	9.9	16.6	6.7	3.2	6.7	16.6	4.9
OPBE	–4.8	6.5	11.3	3.9	0.3	5.2	8.2	3.6	–2.9	6.0	11.3	3.8
XLYP	–1.0	2.5	4.4	1.6	15.2	15.2	19.6	6.1	5.1	7.2	19.6	7.3
						Meta–GGAs						
M06-L	–1.4	2.4	4.2	1.2	4.5	5.5	12.4	4.9	0.8	3.5	12.4	3.5
MVS	–6.2	6.2	13.7	5.2	–1.6	4.3	7.0	2.1	–4.5	5.5	13.7	4.4
TPSS	–4.4	4.4	7.2	1.9	6.0	6.1	10.1	4.3	–0.5	5.0	10.1	3.2
revTPSS	–4.5	4.5	5.5	1.0	3.7	4.7	6.8	2.3	–1.4	4.6	6.8	1.6
						Hybrids						
B3LYP	1.2	2.2	3.7	1.1	8.2	8.2	10.6	3.2	3.8	4.4	10.6	3.6
B3LYP*	–0.7	2.1	3.5	1.2	7.8	7.8	10.3	3.1	2.5	4.2	10.3	3.5
B1LYP	2.6	2.8	4.9	1.7	8.2	8.2	10.6	3.0	4.7	4.8	10.6	3.5
B1PW91	–1.3	2.4	4.3	1.6	–0.1	1.9	2.7	0.9	–0.8	2.2	4.3	1.4
BHandH	–4.3	4.7	8.0	2.7	–14.4	14.4	19.3	7.0	–8.1	8.3	19.3	6.7
BHandHLYP	6.6	6.6	8.1	1.5	2.1	2.1	3.0	0.7	4.9	4.9	8.1	2.5
KMLYP	1.0	1.5	3.4	1.1	–9.9	9.9	13.4	4.8	–3.1	4.7	13.4	5.1
O3LYP	–17.9	17.9	28.8	7.8	–20.0	20.0	28.2	8.8	–18.7	18.7	28.8	8.2
OPBE0	–1.2	4.1	6.1	1.9	–4.7	4.7	7.9	3.4	–2.5	4.3	7.9	2.6
PBE0	–3.4	3.4	7.4	2.6	–3.1	3.1	4.8	1.4	–3.3	3.3	7.4	2.2
mPW1PW	–1.6	2.4	4.7	1.6	–0.4	1.6	1.8	0.3	–1.2	2.1	4.7	1.3
mPW1K	1.2	1.4	4.3	1.5	–4.7	4.7	6.6	1.4	–1.0	2.7	6.6	2.1
S12H	–2.3	2.7	6.1	2.0	–2.6	2.9	5.1	1.9	–2.4	2.8	6.1	2.0
X3LYP	0.8	2.0	2.9	0.6	7.0	7.0	8.8	2.5	3.1	3.8	8.8	2.9
						Meta–Hybrids						
M06	0.1	1.7	4.3	1.5	1.8	2.6	6.6	2.9	0.8	2.0	6.6	2.2
M06-2X	–0.1	1.5	2.2	0.6	–0.9	0.9	1.6	0.5	–0.4	1.3	2.2	0.7
M06-HF	–0.9	1.6	5.3	1.9	–4.2	4.8	12.2	5.2	–2.2	2.8	12.2	3.9
TPSSH	–2.9	2.9	5.0	1.7	3.7	4.2	6.9	2.5	–0.4	3.4	6.9	2.1
						Double–Hybrids						
B2K-PLYP	–0.5	1.1	1.5	0.5	0.9	1.8	3.5	1.2	0.1	1.4	3.5	0.9
B2T-PLYP	–0.2	1.1	1.4	0.4	3.2	3.2	6.0	2.0	1.1	1.8	6.0	1.6
B2-PLYP	–0.5	1.2	2.0	0.7	4.7	4.7	8.0	2.5	1.4	2.5	8.0	2.3
LS1-TPSS	–3.3	3.3	5.8	1.9	–4.1	4.1	8.6	3.3	–3.6	3.6	8.6	2.5
mPW2K-PLYP	–0.6	1.2	1.8	0.5	0.8	1.8	3.2	1.0	–0.1	1.4	3.2	0.8
mPW2-PLYP	0.2	0.9	1.8	0.5	3.8	3.8	6.1	1.7	1.5	2.0	6.1	1.8
PBE0-DH	–2.0	2.2	4.5	1.6	–5.9	5.9	9.1	2.3	–3.4	3.6	9.1	2.6
revDSD-BLYP	–0.6	1.2	2.5	0.8	0.7	1.9	3.0	0.8	–0.1	1.5	3.0	0.9
revDSD-PBE	–1.6	1.7	3.3	1.2	–1.5	2.5	4.8	1.6	–1.6	2.0	4.8	1.4
revDSD-PBEP86	0.1	0.8	1.3	0.4	0.4	2.0	3.6	1.3	0.2	1.3	3.6	1.0
						Range-Separated Hybrids						
CAM-B3LYP	2.4	2.4	4.0	1.4	1.6	1.6	2.4	0.5	2.1	2.1	4.0	1.2
CAMY-B3LYP	1.0	1.6	2.2	0.7	2.7	2.7	3.4	0.6	1.6	2.0	3.4	0.9
ωB97	3.6	3.6	4.1	0.4	–6.1	6.1	8.0	1.5	0.0	4.5	8.0	1.5
ωB97X	2.6	2.6	3.6	0.8	–3.3	3.3	4.4	0.8	0.4	2.9	4.4	0.9
ωB97X-D	1.8	2.0	4.5	1.7	–0.1	1.0	1.8	0.7	1.1	1.6	4.5	1.5

Table S15 (continued).

XC	$\Delta E^{\ddagger}$				ΔE_{rxn}				Total			
	ME	MAE	MUE	SD	ME	MAE	MUE	SD	ME	MAE	MUE	SD
					Dispersion–Corrected							
BP86-D3(BJ)	–9.3	9.3	15.5	3.9	2.2	2.2	5.1	2.0	–5.0	6.6	15.5	4.8
BLYP-D3(BJ)	–5.3	5.3	9.2	2.7	10.3	10.3	13.4	3.2	0.6	7.2	13.4	3.8
PBE-D3(BJ)	–9.7	9.7	16.6	4.1	0.5	1.9	3.6	1.2	–5.9	6.8	16.6	5.0
OLYP-D3(BJ)	–9.2	9.2	14.0	3.8	0.4	2.2	4.0	1.3	–5.6	6.6	14.0	4.6
OPBE-D3(BJ)	–13.2	13.2	20.3	5.1	–8.4	8.4	13.9	4.1	–11.4	11.4	20.3	5.3
B3LYP-D3(BJ)	–2.0	2.5	4.3	1.3	5.1	5.1	6.3	1.0	0.7	3.4	6.3	1.7
PBE0-D3(BJ)	–5.1	5.1	9.3	2.9	–4.7	4.7	7.6	2.0	–4.9	4.9	9.3	2.6
M06-2X-D3	–0.2	1.5	2.2	0.7	–1.0	1.0	1.7	0.6	–0.5	1.3	2.2	0.7

Table S16. Reaction barriers and reaction energies (kcal mol^{-1}) for the various pericyclic reactions, computed with XC/TZ2P//BP86/DZ.

XC	Diels-Alder Reaction		1,3-dipolar Cycloaddition		Electrocyclic Rearrangement		Sigmatropic Rearrangement		Double Group Transfer	
	$\Delta E^{\ddagger}$	ΔE_{rxn}	$\Delta E^{\ddagger}$	ΔE_{rxn}	$\Delta E^{\ddagger}$	ΔE_{rxn}	$\Delta E^{\ddagger}$	ΔE_{rxn}	$\Delta E^{\ddagger}$	ΔE_{rxn}
LDA										
VWN	1.2	–64.0	3.7	–38.6	37.1	3.9	22.2	0.0	22.3	0.0
GGAs										
BP86	15.2	–41.5	15.4	–18.6	40.1	9.1	28.3	0.0	38.2	0.0
BLYP	21.1	–29.7	18.9	–9.6	42.9	14.3	31.8	0.0	45.2	0.0
BEE	16.4	–42.2	17.4	–17.4	39.4	7.4	28.2	0.0	38.6	0.0
PW91	12.6	–45.4	13.5	–21.5	39.8	8.4	27.9	0.0	36.0	0.0
PBE	13.1	–45.4	14.1	–21.2	39.5	7.8	27.7	0.0	36.0	0.0
PBEsol	7.0	–55.9	9.4	–30.5	37.4	4.5	24.2	0.0	28.6	0.0
RPBE	18.9	–37.3	19.3	–13.5	40.4	9.1	29.9	0.0	41.9	0.0
revPBE	18.7	–38.1	19.1	–14.1	40.2	8.8	29.5	0.0	41.4	0.0
mPBE	14.6	–43.2	15.4	–19.1	39.8	8.2	28.3	0.0	37.6	0.0
mPW	15.7	–41.1	16.1	–17.5	40.3	9.2	28.9	0.0	39.1	0.0
HTBS	14.2	–46.2	16.0	–21.3	38.1	5.8	26.3	0.0	35.7	0.0
OLYP	24.3	–34.9	24.1	–10.8	41.1	8.5	31.0	0.0	45.8	0.0
OPBE	19.8	–46.9	22.7	–18.5	37.8	2.0	27.4	0.0	39.4	0.0
XLYP	21.3	–28.5	18.9	–8.8	43.4	15.1	32.4	0.0	45.9	0.0
Meta-GGAs										
M06-L	16.8	–44.6	18.6	–14.7	44.1	7.8	34.4	0.0	46.2	0.0
MVS	12.4	–54.0	17.7	–22.2	43.4	7.1	28.7	0.0	37.1	0.0
TPSS	15.8	–39.5	15.9	–17.5	40.7	8.8	31.0	0.0	43.0	0.0
revTPSS	14.8	–41.7	15.0	–20.2	40.2	7.3	31.6	0.0	45.0	0.0
Hybrids										
B3LYP	22.7	–37.1	21.1	–16.9	45.3	12.6	35.2	0.0	49.6	0.0
B3LYP*	20.8	–37.8	19.4	–17.2	44.2	12.3	33.6	0.0	46.8	0.0
B1LYP	24.1	–36.9	22.3	–17.2	46.2	12.9	36.6	0.0	52.0	0.0
B1PW91	19.8	–47.8	20.9	–24.1	43.2	7.0	33.4	0.0	46.3	0.0
BHandH	13.2	–65.8	14.6	–44.2	45.2	5.2	34.7	0.0	42.8	0.0
BHandHLYP	27.0	–44.2	25.5	–25.2	49.3	11.4	41.5	0.0	58.6	0.0
KMLYP	19.4	–59.8	20.2	–38.5	47.5	6.7	38.5	0.0	50.5	0.0
O3LYP	–1.2	–74.3	2.0	–48.5	38.4	2.2	23.5	0.0	22.2	0.0
OPBE0	21.7	–53.1	24.7	–25.8	41.2	1.8	32.2	0.0	45.7	0.0
PBE0	16.7	–51.8	18.2	–27.8	42.6	6.2	32.5	0.0	43.2	0.0
mPW1PW	18.9	–48.2	19.9	–24.8	43.4	7.5	33.6	0.0	45.8	0.0
mPW1K	21.1	–53.4	22.4	–30.3	45.5	6.2	37.0	0.0	50.5	0.0
S12H	16.6	–52.1	19.0	–25.7	43.6	6.3	34.5	0.0	44.5	0.0
X3LYP	22.0	–38.6	20.5	–18.6	45.4	12.4	35.3	0.0	49.2	0.0
Meta-Hybrids										
M06	19.5	–47.1	21.2	–20.2	44.1	8.2	35.3	0.0	48.6	0.0
M06-2X	17.9	–48.8	20.9	–27.6	45.1	8.8	36.8	0.0	49.0	0.0
M06-HF	15.1	–48.8	20.3	–38.7	43.7	9.7	38.2	0.0	51.2	0.0
TPSSH	17.1	–42.5	17.4	–20.4	41.9	8.1	32.7	0.0	45.4	0.0
Double-Hybrids										
B2K-PLYP	18.5	–48.6	18.2	–21.8	45.3	9.8	36.1	0.0	49.3	0.0
B2T-PLYP	19.4	–45.4	18.6	–19.7	45.1	10.6	35.7	0.0	49.3	0.0
B2-PLYP	19.3	–43.4	18.3	–18.0	44.7	11.1	35.0	0.0	48.5	0.0
LS1-TPSS	14.6	–55.8	16.5	–25.4	43.6	6.6	34.2	0.0	45.8	0.0
mPW2K-PLYP	18.1	–48.8	17.8	–22.1	45.4	10.0	36.1	0.0	49.1	0.0
mPW2-PLYP	19.7	–44.4	18.6	–19.9	45.5	11.3	36.1	0.0	49.6	0.0
PBE0-DH	17.2	–56.0	18.9	–30.1	43.8	5.5	34.6	0.0	46.2	0.0
revDSD-BLYP	17.4	–49.2	17.4	–22.3	45.3	10.1	37.0	0.0	49.5	0.0
revDSD-PBE	16.6	–52.1	17.3	–23.9	44.1	7.8	36.2	0.0	47.5	0.0
revDSD-PBEP86	18.8	–49.5	18.9	–21.9	44.9	8.7	37.2	0.0	49.7	0.0
Range-Separated Hybrids										
CAM-B3LYP	23.7	–44.9	21.5	–25.4	46.7	10.3	37.6	0.0	51.8	0.0
CAMY-B3LYP	21.7	–43.9	20.1	–24.1	45.9	10.9	36.4	0.0	49.9	0.0
ωB97	23.4	–55.3	22.0	–32.1	47.3	4.8	40.8	0.0	53.6	0.0
ωB97X	22.9	–51.7	21.5	–29.5	46.8	6.8	39.3	0.0	52.1	0.0
ωB97X-D	23.2	–47.2	22.3	–25.5	45.4	7.5	36.8	0.0	50.5	0.0

Table S16 (continued).

XC	Diels-Alder Reaction		1,3-dipolar Cycloaddition		Electrocyclic Rearrangement		Sigmatropic Rearrangement		Double Group Transfer	
	$\Delta E^{\ddagger}$	ΔE_{rxn}	$\Delta E^{\ddagger}$	ΔE_{rxn}	$\Delta E^{\ddagger}$	ΔE_{rxn}	$\Delta E^{\ddagger}$	ΔE_{rxn}	$\Delta E^{\ddagger}$	ΔE_{rxn}
					Dispersion-Corrected					
BP86-D3(BJ)	9.1	–46.4	11.3	–22.3	39.8	9.3	27.7	0.0	34.8	0.0
BLYP-D3(BJ)	13.2	–36.0	13.6	–14.7	42.5	14.4	30.8	0.0	40.8	0.0
PBE-D3(BJ)	9.2	–48.6	11.4	–23.6	39.3	7.8	27.3	0.0	33.7	0.0
OLYP-D3(BJ)	8.1	–49.0	12.9	–22.4	39.6	8.2	27.6	0.0	36.5	0.0
OPBE-D3(BJ)	3.8	–60.6	11.7	–29.6	36.4	1.8	24.5	0.0	30.3	0.0
B3LYP-D3(BJ)	16.3	–42.2	16.8	–21.0	44.9	12.7	34.5	0.0	46.0	0.0
PBE0-D3(BJ)	9.6	–48.2	11.7	–23.3	39.3	7.9	27.4	0.0	34.0	0.0
M06-2X-D3	17.7	–49.0	20.8	–27.7	45.1	8.7	36.8	0.0	49.4	0.0

Table S17. Errors in reaction barriers and reaction energies (kcal mol^{-1}) for the various pericyclic reactions, computed with XC/TZ2P//BP86/DZ, compared to CCSDT(Q)/CBS//CCSD(T)/cc-pVTZ.

XC	Diels-Alder Reaction		1,3-dipolar Cycloaddition		Electrocyclic Rearrangement		Sigmatropic Rearrangement		Double Group Transfer	
	$\Delta E^{\ddagger}$	ΔE_{rxn}	$\Delta E^{\ddagger}$	ΔE_{rxn}	$\Delta E^{\ddagger}$	ΔE_{rxn}	$\Delta E^{\ddagger}$	ΔE_{rxn}	$\Delta E^{\ddagger}$	ΔE_{rxn}
LDA										
VWN	–18.4	–16.4	–14.6	–9.7	–6.3	–4.6	–14.6	0.0	–27.9	0.0
GGAs										
BP86	–4.4	6.2	–2.9	10.3	–3.3	0.7	–8.5	0.0	–12.0	0.0
BLYP	1.5	18.0	0.6	19.3	–0.5	5.9	–5.0	0.0	–5.0	0.0
BEE	–3.2	5.5	–0.9	11.5	–4.0	–1.1	–8.6	0.0	–11.6	0.0
PW91	–7.0	2.3	–4.8	7.4	–3.6	0.0	–8.9	0.0	–14.2	0.0
PBE	–6.5	2.3	–4.2	7.7	–3.9	–0.6	–9.1	0.0	–14.2	0.0
PBEsol	–12.6	–8.3	–8.9	–1.6	–6.0	–4.0	–12.6	0.0	–21.6	0.0
RPBE	–0.7	10.4	1.0	15.4	–3.0	0.7	–6.9	0.0	–8.3	0.0
revPBE	–0.9	9.6	0.8	14.8	–3.2	0.4	–7.3	0.0	–8.8	0.0
mPBE	–5.0	4.5	–2.9	9.8	–3.6	–0.3	–8.5	0.0	–12.6	0.0
mPW	–3.9	6.6	–2.2	11.4	–3.1	0.8	–7.9	0.0	–11.1	0.0
HTBS	–5.4	1.5	–2.3	7.6	–5.3	–2.7	–10.5	0.0	–14.5	0.0
OLYP	4.7	12.8	5.8	18.1	–2.3	0.1	–5.8	0.0	–4.4	0.0
OPBE	0.2	0.8	4.4	10.4	–5.6	–6.5	–9.4	0.0	–10.8	0.0
XLYP	1.7	19.2	0.6	20.1	0.0	6.7	–4.4	0.0	–4.3	0.0
Meta-GGAs										
M06-L	–2.8	3.1	0.3	14.2	0.7	–0.6	–2.4	0.0	–4.0	0.0
MVS	–7.2	–6.4	–0.6	6.7	0.0	–1.4	–8.1	0.0	–13.1	0.0
TPSS	–3.8	8.2	–2.4	11.4	–2.7	0.4	–5.8	0.0	–7.2	0.0
revTPSS	–4.8	6.0	–3.3	8.7	–3.2	–1.2	–5.2	0.0	–5.2	0.0
Hybrids										
B3LYP	3.1	10.6	2.8	12.0	1.9	4.2	–1.6	0.0	–0.6	0.0
B3LYP*	1.2	9.9	1.1	11.7	0.8	3.9	–3.2	0.0	–3.4	0.0
B1LYP	4.5	10.8	4.0	11.7	2.8	4.5	–0.2	0.0	1.8	0.0
B1PW91	0.2	–0.1	2.6	4.8	–0.2	–1.5	–3.4	0.0	–3.9	0.0
BHandH	–6.4	–18.2	–3.7	–15.3	1.8	–3.3	–2.1	0.0	–7.4	0.0
BHandHLYP	7.4	3.5	7.2	3.7	5.9	3.0	4.7	0.0	8.4	0.0
KMLYP	–0.2	–12.2	1.9	–9.6	4.1	–1.8	1.7	0.0	0.3	0.0
O3LYP	–20.8	–26.7	–16.3	–19.6	–5.0	–6.3	–13.3	0.0	–28.0	0.0
OPBE0	2.1	–5.5	6.4	3.1	–2.2	–6.7	–4.6	0.0	–4.5	0.0
PBE0	–2.9	–4.2	–0.1	1.1	–0.8	–2.3	–4.3	0.0	–7.0	0.0
mPW1PW	–0.7	–0.6	1.6	4.1	0.0	–0.9	–3.2	0.0	–4.4	0.0
mPW1K	1.5	–5.8	4.1	–1.4	2.1	–2.3	0.2	0.0	0.3	0.0
S12H	–3.0	–4.5	0.7	3.2	0.2	–2.2	–2.3	0.0	–5.7	0.0
X3LYP	2.4	9.1	2.2	10.3	2.0	4.0	–1.5	0.0	–1.0	0.0
Meta-Hybrids										
M06	–0.1	0.5	2.9	8.7	0.7	–0.3	–1.5	0.0	–1.6	0.0
M06-2X	–1.7	–1.2	2.6	1.3	1.7	0.4	0.0	0.0	–1.2	0.0
M06-HF	–4.5	–1.2	2.0	–9.8	0.3	1.3	1.4	0.0	1.0	0.0
TPSSH	–2.5	5.2	–0.9	8.5	–1.5	–0.4	–4.1	0.0	–4.8	0.0
Double-Hybrids										
B2K-PLYP	–1.1	–1.0	–0.1	7.1	1.9	1.4	–0.7	0.0	–0.9	0.0
B2T-PLYP	–0.2	2.3	0.3	9.2	1.7	2.2	–1.1	0.0	–0.9	0.0
B2-PLYP	–0.3	4.3	0.0	10.9	1.3	2.7	–1.8	0.0	–1.7	0.0
LS1-TPSS	–5.0	–8.2	–1.8	3.5	0.2	–1.9	–2.6	0.0	–4.4	0.0
mPW2K-PLYP	–1.5	–1.2	–0.5	6.8	2.0	1.6	–0.7	0.0	–1.1	0.0
mPW2-PLYP	0.1	3.3	0.3	9.0	2.1	2.9	–0.7	0.0	–0.6	0.0
PBE0-DH	–2.4	–8.4	0.6	–1.2	0.4	–3.0	–2.2	0.0	–4.0	0.0
revDSD-BLYP	–2.2	–1.6	–0.9	6.6	1.9	1.7	0.2	0.0	–0.7	0.0
revDSD-PBE	–3.0	–4.5	–1.0	5.0	0.7	–0.6	–0.6	0.0	–2.7	0.0
revDSD-PBEP86	–0.8	–1.9	0.6	7.0	1.5	0.3	0.4	0.0	–0.5	0.0
Range-Separated Hybrids										
CAM-B3LYP	4.1	2.8	3.2	3.5	3.3	1.9	0.8	0.0	1.6	0.0
CAMY-B3LYP	2.1	3.8	1.8	4.8	2.5	2.5	–0.4	0.0	–0.3	0.0
ωB97	3.8	–7.7	3.7	–3.2	3.9	–3.7	4.0	0.0	3.4	0.0
ωB97X	3.3	–4.1	3.2	–0.6	3.4	–1.7	2.5	0.0	1.9	0.0
ωB97X-D	3.6	0.4	4.0	3.4	2.0	–0.9	0.0	0.0	0.3	0.0

Table S17 (continued).

XC	Diels-Alder Reaction		1,3-dipolar Cycloaddition		Electrocyclic Rearrangement		Sigmatropic Rearrangement		Double Group Transfer	
	$\Delta E^{‡}$	ΔE_{rxn}	$\Delta E^{‡}$	ΔE_{rxn}	$\Delta E^{‡}$	ΔE_{rxn}	$\Delta E^{‡}$	ΔE_{rxn}	$\Delta E^{‡}$	ΔE_{rxn}
Dispersion-Corrected										
BP86-D3(BJ)	–10.5	1.3	–7.0	6.6	–3.6	0.9	–9.1	0.0	–15.4	0.0
BLYP-D3(BJ)	–6.4	11.7	–4.7	14.2	–0.9	6.0	–6.0	0.0	–9.4	0.0
PBE-D3(BJ)	–10.4	–1.0	–6.9	5.3	–4.1	–0.6	–9.5	0.0	–16.5	0.0
OLYP-D3(BJ)	–11.5	–1.4	–5.4	6.5	–3.8	–0.3	–9.2	0.0	–13.7	0.0
OPBE-D3(BJ)	–15.8	–13.0	–6.6	–0.7	–7.0	–6.7	–12.3	0.0	–19.9	0.0
B3LYP-D3(BJ)	–3.3	5.5	–1.5	7.9	1.5	4.3	–2.3	0.0	–4.2	0.0
PBE0-D3(BJ)	–10.0	–0.6	–6.6	5.6	–4.1	–0.5	–9.4	0.0	–16.2	0.0
M06-2X-D3	–1.9	–1.4	2.5	1.2	1.7	0.3	0.0	0.0	–0.8	0.0

Table S18. Mean error (ME), mean absolute error (MAE), maximum unsigned error (MUE) and standard deviation (SD) of reaction barriers and energies (kcal mol^{-1}) for the various pericyclic reactions, computed with XC/TZ2P//BP86/DZ compared to CCSDT(Q)/CBS//CCSD(T)/cc-pVTZ.

XC	$\Delta E^{\ddagger}$				ΔE_{rxn}				Total			
	ME	MAE	MUE	SD	ME	MAE	MUE	SD	ME	MAE	MUE	SD
					LDA							
VWN	–16.4	16.4	27.9	7.0	–10.2	10.2	16.4	4.8	–14.1	14.1	27.9	6.9
					GGAs							
BP86	–6.2	6.2	12.0	3.5	5.7	5.7	10.3	3.9	–1.8	6.0	12.0	3.7
BLYP	–1.7	2.5	5.0	2.0	14.4	14.4	19.3	6.0	4.3	7.0	19.3	7.0
BEE	–5.7	5.7	11.6	3.9	5.3	6.0	11.5	4.3	–1.6	5.8	11.6	4.0
PW91	–7.7	7.7	14.2	3.7	3.2	3.2	7.4	3.1	–3.6	6.0	14.2	4.1
PBE	–7.6	7.6	14.2	3.8	3.1	3.5	7.7	3.0	–3.6	6.1	14.2	4.0
PBEsol	–12.3	12.3	21.6	5.3	–4.6	4.6	8.3	2.7	–9.4	9.4	21.6	5.8
RPBE	–3.6	4.0	8.3	3.1	8.8	8.8	15.4	6.1	1.1	5.8	15.4	5.0
revPBE	–3.9	4.2	8.8	3.3	8.2	8.2	14.8	6.0	0.7	5.7	14.8	4.9
mPBE	–6.5	6.5	12.6	3.6	4.7	4.8	9.8	3.9	–2.3	5.9	12.6	3.8
mPW	–5.6	5.6	11.1	3.4	6.2	6.2	11.4	4.3	–1.2	5.9	11.4	3.8
HTBS	–7.6	7.6	14.5	4.4	2.1	3.9	7.6	2.6	–4.0	6.2	14.5	4.2
OLYP	–0.4	4.6	5.8	1.3	10.3	10.3	18.1	7.6	3.6	6.7	18.1	5.5
OPBE	–4.2	6.1	10.8	3.8	1.6	5.9	10.4	3.9	–2.1	6.0	10.8	3.8
XLYP	–1.3	2.2	4.4	1.8	15.3	15.3	20.1	6.1	4.9	7.1	20.1	7.5
					Meta–GGAs							
M06-L	–1.6	2.0	4.0	1.4	5.5	6.0	14.2	5.9	1.0	3.5	14.2	4.2
MVS	–5.8	5.8	13.1	4.9	–0.3	4.8	6.7	2.4	–3.8	5.4	13.1	4.2
TPSS	–4.4	4.4	7.2	1.9	6.6	6.6	11.4	4.6	–0.3	5.2	11.4	3.4
revTPSS	–4.3	4.3	5.2	0.9	4.5	5.3	8.7	3.1	–1.0	4.7	8.7	2.1
					Hybrids							
B3LYP	1.1	2.0	3.1	0.9	8.9	8.9	12.0	3.4	4.0	4.6	12.0	4.0
B3LYP*	–0.7	1.9	3.4	1.1	8.5	8.5	11.7	3.3	2.7	4.4	11.7	3.9
B1LYP	2.6	2.7	4.5	1.6	9.0	9.0	11.7	3.2	5.0	5.0	11.7	3.8
B1PW91	–0.9	2.1	3.9	1.6	1.1	2.1	4.8	1.9	–0.2	2.1	4.8	1.7
BHandH	–3.6	4.3	7.4	2.3	–12.2	12.2	18.2	6.5	–6.8	7.3	18.2	5.8
BHandHLYP	6.7	6.7	8.4	1.3	3.4	3.4	3.7	0.3	5.5	5.5	8.4	1.9
KMLYP	1.6	1.6	4.1	1.4	–7.8	7.8	12.2	4.4	–2.0	4.0	12.2	4.2
O3LYP	–16.7	16.7	28.0	7.6	–17.5	17.5	26.7	8.5	–17.0	17.0	28.0	8.0
OPBE0	–0.6	4.0	6.4	1.6	–3.0	5.1	6.7	1.5	–1.5	4.4	6.7	1.7
PBE0	–3.0	3.0	7.0	2.5	–1.8	2.5	4.2	1.3	–2.6	2.8	7.0	2.1
mPW1PW	–1.3	2.0	4.4	1.6	0.9	1.9	4.1	1.6	–0.5	1.9	4.4	1.6
mPW1K	1.6	1.6	4.1	1.4	–3.1	3.1	5.8	1.9	–0.2	2.2	5.8	1.8
S12H	–2.0	2.4	5.7	2.0	–1.1	3.3	4.5	0.9	–1.7	2.7	5.7	1.7
X3LYP	0.8	1.8	2.4	0.5	7.8	7.8	10.3	2.7	3.4	4.0	10.3	3.3
					Meta–Hybrids							
M06	0.1	1.4	2.9	1.0	3.0	3.2	8.7	3.9	1.2	2.0	8.7	2.7
M06-2X	0.3	1.4	2.6	0.8	0.2	0.9	1.3	0.4	0.2	1.2	2.6	0.8
M06-HF	0.0	1.8	4.5	1.4	–3.2	4.1	9.8	4.1	–1.2	2.7	9.8	3.0
TPSSH	–2.8	2.8	4.8	1.5	4.4	4.7	8.5	3.3	–0.1	3.5	8.5	2.5
					Double–Hybrids							
B2K-PLYP	–0.2	0.9	1.9	0.6	2.5	3.1	7.1	2.8	0.8	1.7	7.1	2.1
B2T-PLYP	0.0	0.8	1.7	0.5	4.5	4.5	9.2	3.3	1.7	2.2	9.2	2.7
B2-PLYP	–0.5	1.0	1.8	0.7	5.9	5.9	10.9	3.6	1.9	2.9	10.9	3.3
LS1-TPSS	–2.7	2.8	5.0	1.8	–2.2	4.5	8.2	2.7	–2.5	3.4	8.2	2.3
mPW2K-PLYP	–0.4	1.1	2.0	0.5	2.4	3.2	6.8	2.6	0.7	1.9	6.8	1.9
mPW2-PLYP	0.2	0.8	2.1	0.7	5.0	5.0	9.0	2.8	2.0	2.4	9.0	2.7
PBE0-DH	–1.5	1.9	4.0	1.3	–4.2	4.2	8.4	3.0	–2.5	2.8	8.4	2.4
revDSD-BLYP	–0.3	1.2	2.2	0.7	2.2	3.3	6.6	2.3	0.6	2.0	6.6	1.8
revDSD-PBE	–1.3	1.6	3.0	1.0	0.0	3.4	5.0	1.9	–0.8	2.2	5.0	1.7
revDSD-PBEP86	0.2	0.8	1.5	0.4	1.8	3.0	7.0	2.9	0.8	1.6	7.0	2.1
					Range-Separated Hybrids							
CAM-B3LYP	2.6	2.6	4.1	1.2	2.7	2.7	3.5	0.7	2.6	2.6	4.1	1.0
CAMY-B3LYP	1.1	1.4	2.5	0.9	3.7	3.7	4.8	0.9	2.1	2.3	4.8	1.4
ωB97	3.8	3.8	4.0	0.2	–4.8	4.8	7.7	2.0	0.5	4.2	7.7	1.3
ωB97X	2.9	2.9	3.4	0.6	–2.1	2.1	4.1	1.4	1.0	2.6	4.1	1.1
ωB97X-D	2.0	2.0	4.0	1.7	1.0	1.6	3.4	1.3	1.6	1.8	4.0	1.5

Table S18 (continued).

XC	$\Delta E^{\ddagger}$				ΔE_{rxn}				Total			
	ME	MAE	MUE	SD	ME	MAE	MUE	SD	ME	MAE	MUE	SD
					Dispersion–Corrected							
BP86-D3(BJ)	–9.1	9.1	15.4	3.9	2.9	2.9	6.6	2.6	–4.6	6.8	15.4	4.6
BLYP-D3(BJ)	–5.5	5.5	9.4	2.8	10.6	10.6	14.2	3.4	0.5	7.4	14.2	3.9
PBE-D3(BJ)	–9.5	9.5	16.5	4.1	1.2	2.3	5.3	2.1	–5.5	6.8	16.5	5.0
OLYP-D3(BJ)	–8.7	8.7	13.7	3.7	1.6	2.7	6.5	2.7	–4.8	6.5	13.7	4.4
OPBE-D3(BJ)	–12.3	12.3	19.9	5.1	–6.8	6.8	13.0	5.0	–10.2	10.2	19.9	5.7
B3LYP-D3(BJ)	–2.0	2.5	4.2	1.1	5.9	5.9	7.9	1.5	1.0	3.8	7.9	2.0
PBE0-D3(BJ)	–9.3	9.3	16.2	4.1	1.5	2.2	5.6	2.4	–5.2	6.6	16.2	4.9
M06-2X-D3	0.3	1.4	2.5	0.9	0.0	0.9	1.4	0.5	0.2	1.2	2.5	0.8

Table S19. Reaction barriers and reaction energies (kcal mol^{-1}) for the various pericyclic reactions, computed with XC/QZ4P//BP86/DZ.

XC	Diels-Alder Reaction		1,3-dipolar Cycloaddition		Electrocyclic Rearrangement		Sigmatropic Rearrangement		Double Group Transfer	
	$\Delta E^{\ddagger}$	ΔE_{rxn}	$\Delta E^{\ddagger}$	ΔE_{rxn}	$\Delta E^{\ddagger}$	ΔE_{rxn}	$\Delta E^{\ddagger}$	ΔE_{rxn}	$\Delta E^{\ddagger}$	ΔE_{rxn}
LDA										
VWN	0.9	–64.5	3.6	–39.4	36.8	3.4	22.2	0.0	22.3	0.0
GGAs										
BP86	15.0	–42.2	15.3	–19.3	39.8	8.6	28.3	0.0	38.2	0.0
BLYP	20.9	–30.3	18.8	–10.2	42.7	13.9	31.7	0.0	45.2	0.0
BEE	16.1	–43.0	17.2	–18.5	39.0	6.7	28.1	0.0	38.5	0.0
PW91	12.4	–46.0	13.3	–22.4	39.4	7.8	27.9	0.0	35.9	0.0
PBE	12.9	–46.1	13.9	–22.1	39.1	7.1	27.7	0.0	35.9	0.0
PBEsol	6.8	–56.5	9.2	–31.4	37.0	3.8	24.2	0.0	28.6	0.0
RPBE	18.7	–38.1	19.0	–14.6	40.0	8.4	29.8	0.0	41.7	0.0
revPBE	18.4	–38.9	18.9	–15.1	39.8	8.1	29.4	0.0	41.3	0.0
mPBE	14.4	–43.9	15.2	–20.1	39.4	7.5	28.3	0.0	37.5	0.0
mPW	15.5	–41.8	16.0	–18.4	40.0	8.6	28.9	0.0	39.0	0.0
HTBS	14.0	–46.9	15.6	–22.5	37.6	5.1	26.3	0.0	35.6	0.0
OLYP	24.0	–35.7	23.7	–12.0	40.6	7.6	30.9	0.0	45.5	0.0
OPBE	19.5	–48.0	22.2	–20.2	37.1	0.9	27.3	0.0	39.3	0.0
XLYP	21.2	–29.0	18.8	–9.4	43.1	14.7	32.3	0.0	45.9	0.0
Meta-GGAs										
M06-L	17.0	–45.6	18.8	–15.4	43.9	6.8	34.3	0.0	48.1	0.0
MVS	13.0	–53.9	18.3	–22.5	43.0	6.6	28.8	0.0	37.1	0.0
TPSS	15.6	–40.3	15.7	–18.4	40.3	8.1	31.0	0.0	43.0	0.0
revTPSS	14.7	–42.3	14.9	–21.1	39.8	6.6	31.6	0.0	45.0	0.0
Hybrids										
B3LYP	22.7	–37.4	21.1	–17.3	45.1	12.2	35.2	0.0	49.7	0.0
B3LYP*	20.7	–38.1	19.4	–14.7	44.0	11.9	33.6	0.0	46.8	0.0
B1LYP	24.1	–37.1	22.3	–17.6	46.0	12.5	36.6	0.0	52.1	0.0
B1PW91	19.8	–48.1	20.9	–24.7	42.8	6.4	33.4	0.0	46.4	0.0
BHandH	13.4	–65.6	14.8	–44.5	44.9	4.8	34.8	0.0	43.0	0.0
BHandHLYP	27.2	–44.1	25.7	–25.4	49.1	11.1	41.7	0.0	58.8	0.0
KMLYP	19.8	–59.5	20.5	–38.8	47.2	6.3	38.7	0.0	50.7	0.0
O3LYP	–1.4	–74.6	1.9	–49.2	38.1	1.7	23.5	0.0	22.3	0.0
OPBE0	21.6	–53.7	24.5	–27.1	40.6	0.8	32.2	0.0	45.7	0.0
PBE0	16.8	–52.2	18.2	–28.5	42.3	5.7	32.6	0.0	43.3	0.0
mPW1PW	18.9	–48.6	19.9	–25.5	43.1	7.0	33.6	0.0	45.9	0.0
mPW1K	21.3	–53.5	22.5	–30.8	45.2	5.7	37.1	0.0	50.7	0.0
S12H	16.6	–52.7	19.0	–26.6	43.2	5.4	34.5	0.0	44.5	0.0
X3LYP	22.0	–38.9	20.5	–19.0	45.2	12.0	35.4	0.0	49.3	0.0
Meta-Hybrids										
M06	19.6	–47.3	21.4	–20.7	43.8	7.8	35.3	0.0	48.6	0.0
M06-2X	18.2	–48.5	21.1	–27.6	45.0	8.5	36.9	0.0	49.3	0.0
M06-HF	14.5	–48.3	19.5	–38.3	43.4	9.8	38.1	0.0	51.0	0.0
TPSSH	17.0	–43.1	17.3	–21.2	41.5	7.4	32.7	0.0	45.4	0.0
Double-Hybrids										
B2K-PLYP	18.0	–53.2	17.4	–22.3	44.3	9.2	35.8	0.0	50.5	0.0
B2T-PLYP	19.0	–48.7	17.9	–20.1	44.3	10.1	35.6	0.0	50.2	0.0
B2-PLYP	19.0	–46.3	17.7	–18.4	43.9	10.6	34.9	0.0	49.3	0.0
LS1-TPSS	13.8	–55.6	15.3	–26.0	42.2	5.7	33.9	0.0	47.2	0.0
mPW2K-PLYP	17.7	–53.4	17.0	–22.5	44.4	9.4	35.9	0.0	50.3	0.0
mPW2-PLYP	19.5	–47.0	18.1	–20.2	44.8	10.8	36.0	0.0	50.5	0.0
PBE0-DH	17.2	–57.4	18.7	–30.6	43.2	4.9	34.6	0.0	46.7	0.0
revDSD-BLYP	17.2	–52.9	16.8	–22.3	44.4	9.6	36.8	0.0	50.9	0.0
revDSD-PBE	16.6	–55.3	16.8	–23.9	43.1	7.3	36.0	0.0	48.9	0.0
revDSD-PBEP86	18.8	–52.6	18.4	–21.8	43.9	8.2	37.1	0.0	51.0	0.0
Range-Separated Hybrids										
CAM-B3LYP	23.8	–45.0	21.7	–25.7	46.4	9.9	37.7	0.0	52.0	0.0
CAMY-B3LYP	21.8	–44.1	20.2	–24.4	45.7	10.5	36.5	0.0	50.0	0.0
ωB97	23.6	–55.8	22.2	–32.7	46.9	4.0	40.9	0.0	53.6	0.0
ωB97X	23.0	–51.9	21.8	–30.0	46.4	6.2	39.3	0.0	52.1	0.0
ωB97X-D	23.3	–47.3	22.5	–25.9	45.1	7.0	36.9	0.0	50.6	0.0

Table S19 (continued).

XC	Diels-Alder Reaction		1,3-dipolar Cycloaddition		Electrocyclic Rearrangement		Sigmatropic Rearrangement		Double Group Transfer	
	$\Delta E^{\ddagger}$	ΔE_{rxn}	$\Delta E^{\ddagger}$	ΔE_{rxn}	$\Delta E^{\ddagger}$	ΔE_{rxn}	$\Delta E^{\ddagger}$	ΔE_{rxn}	$\Delta E^{\ddagger}$	ΔE_{rxn}
					Dispersion-Corrected					
BP86-D3(BJ)	8.6	–47.0	11.2	–23.1	39.5	8.7	27.7	0.0	34.8	0.0
BLYP-D3(BJ)	13.1	–36.6	13.5	–15.3	42.2	14.0	30.8	0.0	40.9	0.0
PBE-D3(BJ)	9.0	–49.3	11.3	–24.6	38.9	7.2	27.3	0.0	33.7	0.0
OLYP-D3(BJ)	7.9	–49.9	12.6	–23.6	39.1	7.4	27.5	0.0	36.2	0.0
OPBE-D3(BJ)	3.5	–61.7	11.3	–31.3	35.7	0.7	24.3	0.0	30.2	0.0
B3LYP-D3(BJ)	16.3	–42.6	16.8	–21.4	44.7	12.3	34.5	0.0	46.1	0.0
PBE0-D3(BJ)	9.4	–48.9	11.6	–24.3	39.0	7.2	27.4	0.0	33.9	0.0
M06-2X-D3	18.1	–48.5	21.0	–27.6	45.0	8.5	37.0	0.0	49.2	0.0

Table S20. Errors in reaction barriers and reaction energies (kcal mol^{-1}) for the various pericyclic reactions, computed with XC/QZ4P//BP86/DZ, compared to CCSDT(Q)/CBS//CCSD(T)/cc-pVTZ.

XC	Diels-Alder Reaction		1,3-dipolar Cycloaddition		Electrocyclic Rearrangement		Sigmatropic Rearrangement		Double Group Transfer	
	$\Delta E^{\ddagger}$	ΔE_{rxn}	$\Delta E^{\ddagger}$	ΔE_{rxn}	$\Delta E^{\ddagger}$	ΔE_{rxn}	$\Delta E^{\ddagger}$	ΔE_{rxn}	$\Delta E^{\ddagger}$	ΔE_{rxn}
LDA										
VWN	–18.7	–16.9	–14.7	–10.5	–6.6	–5.1	–14.6	0.0	–27.9	0.0
GGAs										
BP86	–4.6	5.5	–3.0	9.6	–3.6	0.2	–8.5	0.0	–12.0	0.0
BLYP	1.3	17.4	0.5	18.7	–0.7	5.5	–5.1	0.0	–5.0	0.0
BEE	–3.5	4.7	–1.1	10.4	–4.4	–1.8	–8.7	0.0	–11.7	0.0
PW91	–7.2	1.7	–5.0	6.5	–4.0	–0.6	–8.9	0.0	–14.3	0.0
PBE	–6.7	1.6	–4.4	6.8	–4.3	–1.4	–9.1	0.0	–14.3	0.0
PBEsol	–12.8	–8.9	–9.1	–2.5	–6.4	–4.7	–12.6	0.0	–21.6	0.0
RPBE	–0.9	9.6	0.7	14.3	–3.4	0.0	–7.0	0.0	–8.5	0.0
revPBE	–1.2	8.8	0.6	13.8	–3.6	–0.4	–7.4	0.0	–8.9	0.0
mPBE	–5.2	3.8	–3.1	8.8	–4.0	–0.9	–8.5	0.0	–12.7	0.0
mPW	–4.1	5.9	–2.3	10.5	–3.4	0.2	–7.9	0.0	–11.2	0.0
HTBS	–5.6	0.8	–2.7	6.4	–5.8	–3.4	–10.5	0.0	–14.6	0.0
OLYP	4.4	12.0	5.4	16.9	–2.8	–0.9	–5.9	0.0	–4.7	0.0
OPBE	–0.1	–0.4	3.9	8.7	–6.3	–7.6	–9.5	0.0	–10.9	0.0
XLYP	1.6	18.7	0.5	19.5	–0.3	6.3	–4.5	0.0	–4.3	0.0
Meta-GGAs										
M06-L	–2.6	2.1	0.5	13.5	0.5	–1.7	–2.5	0.0	–2.1	0.0
MVS	–6.6	–6.3	0.0	6.4	–0.4	–1.9	–8.0	0.0	–13.1	0.0
TPSS	–4.0	7.4	–2.6	10.5	–3.1	–0.4	–5.8	0.0	–7.2	0.0
revTPSS	–4.9	5.4	–3.4	7.8	–3.6	–1.9	–5.2	0.0	–5.2	0.0
Hybrids										
B3LYP	3.1	10.3	2.8	11.6	1.7	3.8	–1.6	0.0	–0.5	0.0
B3LYP*	1.1	9.6	1.1	14.2	0.6	3.5	–3.2	0.0	–3.4	0.0
B1LYP	4.5	10.6	4.0	11.3	2.6	4.1	–0.2	0.0	1.9	0.0
B1PW91	0.2	–0.5	2.6	4.2	–0.6	–2.1	–3.4	0.0	–3.8	0.0
BHandH	–6.2	–18.0	–3.5	–15.6	1.5	–3.7	–2.0	0.0	–7.2	0.0
BHandHLYP	7.6	3.6	7.4	3.5	5.7	2.7	4.9	0.0	8.6	0.0
KMLYP	0.2	–11.9	2.2	–9.9	3.8	–2.2	1.9	0.0	0.5	0.0
O3LYP	–21.0	–27.0	–16.4	–20.3	–5.3	–6.8	–13.3	0.0	–27.9	0.0
OPBE0	2.0	–6.1	6.2	1.8	–2.8	–7.7	–4.6	0.0	–4.5	0.0
PBE0	–2.8	–4.6	–0.1	0.4	–1.1	–2.8	–4.2	0.0	–6.9	0.0
mPW1PW	–0.7	–1.0	1.6	3.4	–0.3	–1.5	–3.2	0.0	–4.3	0.0
mPW1K	1.7	–5.9	4.2	–1.9	1.8	–2.8	0.3	0.0	0.5	0.0
S12H	–3.0	–5.1	0.7	2.3	–0.2	–3.1	–2.3	0.0	–5.7	0.0
X3LYP	2.4	8.8	2.2	9.9	1.8	3.6	–1.4	0.0	–0.9	0.0
Meta-Hybrids										
M06	0.0	0.4	3.1	8.2	0.4	–0.6	–1.5	0.0	–1.6	0.0
M06-2X	–1.4	–0.9	2.8	1.3	1.6	0.1	0.1	0.0	–0.9	0.0
M06-HF	–5.1	–0.6	1.2	–9.4	0.0	1.4	1.3	0.0	0.8	0.0
TPSSH	–2.6	4.6	–1.0	7.7	–1.9	–1.1	–4.1	0.0	–4.8	0.0
Double-Hybrids										
B2K-PLYP	–1.6	–5.6	–0.9	6.6	0.9	0.8	–1.0	0.0	0.3	0.0
B2T-PLYP	–0.6	–1.1	–0.4	8.8	0.9	1.7	–1.2	0.0	0.0	0.0
B2-PLYP	–0.6	1.4	–0.6	10.5	0.5	2.2	–1.9	0.0	–0.9	0.0
LS1-TPSS	–5.8	–8.0	–3.0	2.9	–1.2	–2.8	–2.9	0.0	–3.0	0.0
mPW2K-PLYP	–1.9	–5.8	–1.3	6.4	1.0	1.0	–0.9	0.0	0.1	0.0
mPW2-PLYP	–0.1	0.6	–0.2	8.7	1.4	2.4	–0.8	0.0	0.3	0.0
PBE0-DH	–2.4	–9.8	0.4	–1.7	–0.2	–3.6	–2.2	0.0	–3.5	0.0
revDSD-BLYP	–2.4	–5.3	–1.5	6.6	1.0	1.2	0.0	0.0	0.7	0.0
revDSD-PBE	–3.0	–7.7	–1.5	5.0	–0.3	–1.2	–0.8	0.0	–1.3	0.0
revDSD-PBEP86	–0.8	–5.0	0.1	7.1	0.5	–0.3	0.3	0.0	0.8	0.0
Range-Separated Hybrids										
CAM-B3LYP	4.2	2.7	3.4	3.2	3.0	1.5	0.9	0.0	1.8	0.0
CAMY-B3LYP	2.2	3.6	1.9	4.5	2.3	2.1	–0.3	0.0	–0.2	0.0
ωB97	4.0	–8.2	3.9	–3.8	3.5	–4.5	4.1	0.0	3.4	0.0
ωB97X	3.4	–4.3	3.5	–1.1	3.0	–2.3	2.5	0.0	1.9	0.0
ωB97X-D	3.7	0.4	4.2	3.0	1.7	–1.5	0.1	0.0	0.4	0.0

Table S20 (continued).

XC	Diels-Alder Reaction		1,3-dipolar Cycloaddition		Electrocyclic Rearrangement		Sigmatropic Rearrangement		Double Group Transfer	
	$\Delta E^{‡}$	ΔE_{rxn}	$\Delta E^{‡}$	ΔE_{rxn}	$\Delta E^{‡}$	ΔE_{rxn}	$\Delta E^{‡}$	ΔE_{rxn}	$\Delta E^{‡}$	ΔE_{rxn}
					Dispersion-Corrected					
BP86-D3(BJ)	–11.0	0.6	–7.1	5.8	–3.9	0.3	–9.1	0.0	–15.4	0.0
BLYP-D3(BJ)	–6.5	11.1	–4.8	13.6	–1.2	5.6	–6.0	0.0	–9.3	0.0
PBE-D3(BJ)	–10.6	–1.7	–7.0	4.3	–4.5	–1.3	–9.5	0.0	–16.5	0.0
OLYP-D3(BJ)	–11.7	–2.3	–5.7	5.3	–4.3	–1.1	–9.3	0.0	–14.0	0.0
OPBE-D3(BJ)	–16.1	–14.1	–7.0	–2.4	–7.7	–7.8	–12.5	0.0	–20.0	0.0
B3LYP-D3(BJ)	–3.3	5.1	–1.5	7.5	1.3	3.9	–2.3	0.0	–4.1	0.0
PBE0-D3(BJ)	–10.2	–1.3	–6.7	4.6	–4.4	–1.3	–9.4	0.0	–16.3	0.0
M06-2X-D3	–1.5	–0.9	2.7	1.3	1.6	0.1	0.2	0.0	–1.0	0.0

Table S21. Mean error (ME), mean absolute error (MAE), maximum unsigned error (MUE) and standard deviation (SD) of reaction barriers and energies (kcal mol^{-1}) for the various pericyclic reactions, computed with XC/QZ4P//BP86/DZ compared to CCSDT(Q)/CBS//CCSD(T)/cc-pVTZ.

XC	$\Delta E^{\ddagger}$				ΔE_{rxn}				Total			
	ME	MAE	MUE	SD	ME	MAE	MUE	SD	ME	MAE	MUE	SD
					LDA							
VWN	–16.5	16.5	27.9	6.9	–10.8	10.8	16.9	4.8	–14.4	14.4	27.9	6.8
					GGAs							
BP86	–6.3	6.3	12.0	3.4	5.1	5.1	9.6	3.9	–2.1	5.9	12.0	3.6
BLYP	–1.8	2.5	5.1	2.1	13.8	13.8	18.7	5.9	4.1	6.8	18.7	6.8
BEE	–5.9	5.9	11.7	3.8	4.4	5.6	10.4	3.6	–2.0	5.8	11.7	3.7
PW91	–7.9	7.9	14.3	3.6	2.5	2.9	6.5	2.5	–4.0	6.0	14.3	4.1
PBE	–7.8	7.8	14.3	3.7	2.3	3.2	6.8	2.5	–4.0	6.1	14.3	4.0
PBEsol	–12.5	12.5	21.6	5.1	–5.3	5.3	8.9	2.6	–9.8	9.8	21.6	5.6
RPBE	–3.8	4.1	8.5	3.2	7.9	8.0	14.3	5.9	0.6	5.5	14.3	4.8
revPBE	–4.1	4.3	8.9	3.3	7.4	7.6	13.8	5.5	0.2	5.6	13.8	4.6
mPBE	–6.7	6.7	12.7	3.5	3.9	4.5	8.8	3.2	–2.7	5.9	12.7	3.6
mPW	–5.8	5.8	11.2	3.3	5.5	5.5	10.5	4.2	–1.6	5.7	11.2	3.7
HTBS	–7.8	7.8	14.6	4.2	1.3	3.5	6.4	2.3	–4.4	6.2	14.6	4.2
OLYP	–0.7	4.7	5.9	1.0	9.3	9.9	16.9	6.7	3.0	6.6	16.9	4.9
OPBE	–4.6	6.1	10.9	3.9	0.3	5.5	8.7	3.7	–2.8	5.9	10.9	3.8
XLYP	–1.4	2.3	4.5	1.8	14.8	14.8	19.5	6.0	4.7	7.0	19.5	7.2
					Meta–GGAs							
M06-L	–1.2	1.6	2.6	0.9	4.6	5.7	13.5	5.5	1.0	3.2	13.5	4.0
MVS	–5.6	5.6	13.1	4.9	–0.6	4.8	6.4	2.1	–3.7	5.3	13.1	4.1
TPSS	–4.5	4.5	7.2	1.7	5.8	6.1	10.5	4.2	–0.7	5.1	10.5	3.0
revTPSS	–4.5	4.5	5.2	0.8	3.8	5.0	7.8	2.4	–1.4	4.7	7.8	1.6
					Hybrids							
B3LYP	1.1	1.9	3.1	0.9	8.5	8.5	11.6	3.4	3.9	4.4	11.6	3.9
B3LYP*	–0.8	1.9	3.4	1.2	9.1	9.1	14.2	4.4	2.9	4.6	14.2	4.5
B1LYP	2.6	2.6	4.5	1.6	8.6	8.6	11.3	3.2	4.8	4.9	11.3	3.7
B1PW91	–1.0	2.1	3.8	1.4	0.6	2.2	4.2	1.5	–0.4	2.2	4.2	1.5
BHandH	–3.5	4.1	7.2	2.3	–12.4	12.4	18.0	6.3	–6.8	7.2	18.0	5.9
BHandHLYP	6.8	6.8	8.6	1.3	3.2	3.2	3.6	0.4	5.5	5.5	8.6	2.1
KMLYP	1.7	1.7	3.8	1.3	–8.0	8.0	11.9	4.2	–1.9	4.1	11.9	4.1
O3LYP	–16.8	16.8	27.9	7.5	–18.0	18.0	27.0	8.4	–17.2	17.2	27.9	7.9
OPBE0	–0.7	4.0	6.2	1.5	–4.0	5.2	7.7	2.5	–2.0	4.5	7.7	2.0
PBE0	–3.0	3.0	6.9	2.4	–2.3	2.6	4.6	1.7	–2.8	2.8	6.9	2.2
mPW1PW	–1.4	2.0	4.3	1.5	0.3	1.9	3.4	1.0	–0.7	2.0	4.3	1.4
mPW1K	1.7	1.7	4.2	1.4	–3.5	3.5	5.9	1.7	–0.3	2.4	5.9	1.7
S12H	–2.1	2.4	5.7	1.9	–1.9	3.5	5.1	1.2	–2.0	2.8	5.7	1.8
X3LYP	0.8	1.7	2.4	0.5	7.4	7.4	9.9	2.8	3.3	3.9	9.9	3.2
					Meta–Hybrids							
M06	0.1	1.3	3.1	1.1	2.6	3.1	8.2	3.6	1.0	2.0	8.2	2.5
M06-2X	0.4	1.4	2.8	0.9	0.2	0.7	1.3	0.5	0.3	1.1	2.8	0.8
M06-HF	–0.4	1.7	5.1	1.7	–2.9	3.8	9.4	4.0	–1.3	2.5	9.4	3.0
TPSSH	–2.9	2.9	4.8	1.4	3.7	4.4	7.7	2.7	–0.4	3.5	7.7	2.1
					Double–Hybrids							
B2K-PLYP	–0.5	0.9	1.6	0.4	0.6	4.3	6.6	2.5	–0.1	2.2	6.6	2.3
B2T-PLYP	–0.3	0.6	1.2	0.4	3.1	3.8	8.8	3.5	1.0	1.8	8.8	2.7
B2-PLYP	–0.7	0.9	1.9	0.5	4.7	4.7	10.5	4.1	1.3	2.3	10.5	3.1
LS1-TPSS	–3.2	3.2	5.8	1.5	–2.6	4.5	8.0	2.4	–3.0	3.7	8.0	2.0
mPW2K-PLYP	–0.6	1.0	1.9	0.6	0.5	4.4	6.4	2.4	–0.2	2.3	6.4	2.2
mPW2-PLYP	0.1	0.5	1.4	0.5	3.9	3.9	8.7	3.4	1.5	1.8	8.7	2.7
PBE0-DH	–1.6	1.7	3.5	1.2	–5.0	5.0	9.8	3.4	–2.9	3.0	9.8	2.8
revDSD-BLYP	–0.4	1.1	2.4	0.8	0.8	4.3	6.6	2.3	0.0	2.3	6.6	2.2
revDSD-PBE	–1.4	1.4	3.0	0.9	–1.3	4.6	7.7	2.7	–1.3	2.6	7.7	2.4
revDSD-PBEP86	0.2	0.5	0.8	0.3	0.6	4.1	7.1	2.8	0.3	1.8	7.1	2.5
					Range-Separated Hybrids							
CAM-B3LYP	2.7	2.7	4.2	1.2	2.4	2.4	3.2	0.7	2.6	2.6	4.2	1.0
CAMY-B3LYP	1.2	1.4	2.3	0.9	3.4	3.4	4.5	1.0	2.0	2.1	4.5	1.3
ωB97	3.8	3.8	4.1	0.3	-5.5	5.5	8.2	1.9	0.3	4.4	8.2	1.4
ωB97X	2.9	2.9	3.5	0.6	-2.5	2.5	4.3	1.3	0.8	2.7	4.3	0.9
ωB97X-D	2.0	2.0	4.2	1.7	0.6	1.6	3.0	1.1	1.5	1.9	4.2	1.5

Table S21 (continued).

XC	$\Delta E^{\ddagger}$				ΔE_{rxn}				Total			
	ME	MAE	MUE	SD	ME	MAE	MUE	SD	ME	MAE	MUE	SD
					Dispersion–Corrected							
BP86-D3(BJ)	–9.3	9.3	15.4	3.8	2.2	2.2	5.8	2.5	–5.0	6.6	15.4	4.8
BLYP-D3(BJ)	–5.6	5.6	9.3	2.6	10.1	10.1	13.6	3.3	0.3	7.2	13.6	3.6
PBE-D3(BJ)	–9.6	9.6	16.5	4.0	0.5	2.4	4.3	1.3	–5.8	6.9	16.5	4.8
OLYP-D3(BJ)	–9.0	9.0	14.0	3.6	0.7	2.9	5.3	1.8	–5.4	6.7	14.0	4.3
OPBE-D3(BJ)	–12.7	12.7	20.0	4.9	–8.1	8.1	14.1	4.7	–10.9	10.9	20.0	5.4
B3LYP-D3(BJ)	–2.0	2.5	4.1	1.1	5.5	5.5	7.5	1.5	0.8	3.6	7.5	1.9
PBE0-D3(BJ)	–9.4	9.4	16.3	4.0	0.7	2.4	4.6	1.6	–5.6	6.8	16.3	4.8
M06-2X-D3	0.4	1.4	2.7	0.8	0.2	0.7	1.3	0.5	0.3	1.1	2.7	0.8

Table S22. Reaction barriers and reaction energies (kcal mol^{-1}) for the various pericyclic reactions, computed with XC/TZ2P//BP86/DZP.

XC	Diels-Alder Reaction		1,3-dipolar Cycloaddition		Electrocyclic Rearrangement		Sigmatropic Rearrangement		Double Group Transfer	
	$\Delta E^{\ddagger}$	ΔE_{rxn}	$\Delta E^{\ddagger}$	ΔE_{rxn}	$\Delta E^{\ddagger}$	ΔE_{rxn}	$\Delta E^{\ddagger}$	ΔE_{rxn}	$\Delta E^{\ddagger}$	ΔE_{rxn}
LDA										
VWN	0.9	–64.6	3.3	–41.3	36.6	2.9	21.9	0.0	22.0	0.0
GGAs										
BP86	15.2	–41.6	15.5	–20.1	39.9	8.8	28.2	0.0	38.1	0.0
BLYP	21.2	–29.6	19.4	–10.6	42.9	14.3	31.8	0.0	45.3	0.0
BEE	16.3	–42.3	17.4	–19.0	39.3	7.0	28.1	0.0	38.5	0.0
PW91	12.5	–45.6	13.5	–23.2	39.6	8.0	27.8	0.0	35.8	0.0
PBE	13.0	–45.6	14.1	–22.9	39.3	7.3	27.6	0.0	35.9	0.0
PBEsol	6.8	–56.2	9.0	–32.7	37.1	3.8	24.0	0.0	28.4	0.0
RPBE	19.0	–37.3	19.4	–14.9	40.3	8.9	29.9	0.0	41.8	0.0
revPBE	18.6	–38.1	19.3	–15.5	40.1	8.5	29.4	0.0	41.3	0.0
mPBE	14.6	–43.3	15.5	–20.7	39.6	7.8	28.3	0.0	37.5	0.0
mPW	15.7	–41.2	16.3	–18.9	40.2	8.9	28.8	0.0	39.0	0.0
HTBS	14.1	–46.4	15.8	–23.0	37.9	5.3	26.1	0.0	35.5	0.0
OLYP	24.2	–35.1	24.3	–12.5	40.9	8.0	30.9	0.0	45.6	0.0
OPBE	19.6	–47.3	22.4	–20.8	37.4	1.2	27.1	0.0	39.1	0.0
XLYP	21.5	–28.4	19.5	–9.7	43.4	15.1	32.4	0.0	46.0	0.0
Meta-GGAs										
M06-L	16.9	–44.9	19.9	–16.5	43.8	6.8	34.2	0.0	46.0	0.0
MVS	12.3	–54.3	18.1	–24.6	43.1	6.5	28.3	0.0	36.7	0.0
TPSS	15.7	–39.5	16.1	–19.0	40.5	8.4	30.9	0.0	42.9	0.0
revTPSS	14.7	–41.7	15.2	–22.2	40.0	6.9	31.5	0.0	44.8	0.0
Hybrids										
B3LYP	22.7	–37.1	21.8	–18.5	45.1	12.2	35.0	0.0	49.5	0.0
B3LYP*	20.8	–37.8	19.9	–18.8	44.0	11.9	33.4	0.0	46.6	0.0
B1LYP	24.1	–36.9	23.0	–18.9	46.0	12.5	36.4	0.0	51.8	0.0
B1PW91	19.7	–48.1	21.2	–26.3	42.8	6.3	33.0	0.0	46.0	0.0
BHandH	13.0	–66.4	14.7	–47.8	44.5	4.0	34.2	0.0	42.3	0.0
BHandHLYP	26.9	–44.4	26.4	–27.7	48.9	10.7	41.1	0.0	58.3	0.0
KMLYP	19.3	–60.3	20.8	–41.9	46.8	5.5	37.9	0.0	50.0	0.0
O3LYP	–1.6	–75.2	1.4	–52.2	37.7	0.7	23.0	0.0	21.7	0.0
OPBE0	21.5	–53.6	24.6	–28.7	40.7	0.7	31.7	0.0	45.3	0.0
PBE0	16.6	–52.2	18.4	–30.3	42.2	5.5	32.2	0.0	43.0	0.0
mPW1PW	18.8	–48.5	20.2	–27.1	43.0	6.8	33.3	0.0	45.5	0.0
mPW1K	20.9	–53.8	22.8	–33.1	45.0	5.3	36.5	0.0	50.1	0.0
S12H	16.5	–52.4	19.4	–28.3	43.2	5.4	34.2	0.0	44.2	0.0
X3LYP	22.0	–38.7	21.1	–20.3	45.2	12.0	35.1	0.0	49.1	0.0
Meta-Hybrids										
M06	19.5	–47.4	22.4	–22.3	43.7	7.4	35.0	0.0	48.3	0.0
M06-2X	17.8	–48.9	20.9	–29.9	44.7	8.2	36.3	0.0	48.7	0.0
M06-HF	14.8	–48.7	19.0	–41.1	43.1	9.4	37.6	0.0	50.8	0.0
TPSSH	17.1	–42.6	17.8	–22.2	41.6	7.6	32.5	0.0	45.2	0.0
Double-Hybrids										
B2K-PLYP	18.4	–48.7	18.5	–25.0	44.9	9.1	35.7	0.0	48.9	0.0
B2T-PLYP	19.3	–45.4	19.1	–22.6	44.8	10.0	35.4	0.0	49.0	0.0
B2-PLYP	19.3	–43.4	18.8	–20.6	44.4	10.6	34.8	0.0	48.2	0.0
LS1-TPSS	14.3	–55.9	16.1	–29.1	43.1	5.7	33.8	0.0	45.3	0.0
mPW2K-PLYP	18.0	–48.8	18.1	–25.3	45.0	9.3	35.8	0.0	48.8	0.0
mPW2-PLYP	19.6	–44.5	19.2	–22.6	45.2	10.7	35.8	0.0	49.4	0.0
PBE0-DH	17.1	–56.3	19.2	–33.2	43.4	4.6	34.2	0.0	45.8	0.0
revDSD-BLYP	17.2	–49.3	17.6	–25.5	45.1	9.5	36.7	0.0	49.1	0.0
revDSD-PBE	16.5	–52.2	17.5	–27.1	43.9	7.2	35.8	0.0	47.1	0.0
revDSD-PBEP86	18.7	–49.5	19.2	–25.0	44.7	8.1	36.9	0.0	49.3	0.0
Range-Separated Hybrids										
CAM-B3LYP	23.7	–45.1	22.0	–27.6	46.3	9.7	37.3	0.0	51.5	0.0
CAMY-B3LYP	21.7	–44.1	20.6	–26.2	45.6	10.3	36.1	0.0	49.6	0.0
ωB97	23.3	–55.4	22.5	–34.7	46.8	4.0	40.3	0.0	53.3	0.0
ωB97X	22.8	–51.8	22.1	–32.0	46.3	6.1	38.8	0.0	51.8	0.0
ωB97X-D	23.2	–47.4	22.9	–27.6	45.0	6.8	36.4	0.0	50.2	0.0

Table S22 (continued).

XC	Diels-Alder Reaction		1,3-dipolar Cycloaddition		Electrocyclic Rearrangement		Sigmatropic Rearrangement		Double Group Transfer	
	$\Delta E^{\ddagger}$	ΔE_{rxn}	$\Delta E^{\ddagger}$	ΔE_{rxn}	$\Delta E^{\ddagger}$	ΔE_{rxn}	$\Delta E^{\ddagger}$	ΔE_{rxn}	$\Delta E^{\ddagger}$	ΔE_{rxn}
					Dispersion-Corrected					
BP86-D3(BJ)	9.0	–46.5	11.3	–23.9	39.7	9.0	27.7	0.0	34.7	0.0
BLYP-D3(BJ)	13.3	–36.0	13.9	–15.8	42.5	14.3	31.0	0.0	40.9	0.0
PBE-D3(BJ)	9.1	–48.8	11.4	–25.4	39.1	7.4	27.2	0.0	33.6	0.0
OLYP-D3(BJ)	7.8	–49.4	12.4	–24.8	39.5	7.7	27.6	0.0	36.3	0.0
OPBE-D3(BJ)	3.4	–61.2	10.9	–32.5	36.2	1.0	24.3	0.0	30.0	0.0
B3LYP-D3(BJ)	16.3	–42.3	17.3	–22.8	44.7	12.3	34.4	0.0	45.9	0.0
PBE0-D3(BJ)	9.5	–48.4	11.7	–25.1	39.2	7.4	27.3	0.0	33.9	0.0
M06-2X-D3	17.7	–49.0	20.8	–29.9	44.7	8.2	36.4	0.0	48.8	0.0

Table S23. Errors in reaction barriers and reaction energies (kcal mol^{-1}) for the various pericyclic reactions, computed with XC/TZ2P//BP86/DZP, compared to CCSDT(Q)/CBS//CCSD(T)/cc-pVTZ.

XC	Diels-Alder Reaction		1,3-dipolar Cycloaddition		Electrocyclic Rearrangement		Sigmatropic Rearrangement		Double Group Transfer	
	$\Delta E^{‡}$	ΔE_{rxn}	$\Delta E^{‡}$	ΔE_{rxn}	$\Delta E^{‡}$	ΔE_{rxn}	$\Delta E^{‡}$	ΔE_{rxn}	$\Delta E^{‡}$	ΔE_{rxn}
LDA										
VWN	–18.7	–17.0	–15.0	–12.4	–6.8	–5.6	–14.9	0.0	–28.2	0.0
GGAs										
BP86	–4.4	6.1	–2.8	8.8	–3.5	0.4	–8.6	0.0	–12.1	0.0
BLYP	1.6	18.1	1.1	18.3	–0.5	5.9	–5.0	0.0	–4.9	0.0
BEE	–3.3	5.4	–0.9	9.9	–4.1	–1.5	–8.7	0.0	–11.7	0.0
PW91	–7.1	2.1	–4.8	5.7	–3.8	–0.4	–9.0	0.0	–14.4	0.0
PBE	–6.6	2.1	–4.2	6.0	–4.1	–1.2	–9.2	0.0	–14.3	0.0
PBEsol	–12.8	–8.6	–9.3	–3.8	–6.3	–4.7	–12.8	0.0	–21.8	0.0
RPBE	–0.6	10.4	1.1	14.0	–3.1	0.5	–6.9	0.0	–8.4	0.0
revPBE	–1.0	9.6	1.0	13.4	–3.3	0.1	–7.4	0.0	–8.9	0.0
mPBE	–5.0	4.4	–2.8	8.2	–3.8	–0.6	–8.5	0.0	–12.7	0.0
mPW	–3.9	6.5	–2.0	10.0	–3.2	0.5	–8.0	0.0	–11.2	0.0
HTBS	–5.5	1.3	–2.5	5.9	–5.5	–3.2	–10.7	0.0	–14.7	0.0
OLYP	4.6	12.6	6.0	16.4	–2.5	–0.4	–5.9	0.0	–4.6	0.0
OPBE	0.0	0.4	4.1	8.1	–6.0	–7.3	–9.7	0.0	–11.1	0.0
XLYP	1.9	19.3	1.2	19.2	0.0	6.7	–4.4	0.0	–4.2	0.0
Meta-GGAs										
M06-L	–2.7	2.8	1.6	12.4	0.4	–1.7	–2.6	0.0	–4.2	0.0
MVS	–7.3	–6.7	–0.2	4.3	–0.3	–2.0	–8.5	0.0	–13.5	0.0
TPSS	–3.9	8.2	–2.2	9.9	–2.9	0.0	–5.9	0.0	–7.3	0.0
revTPSS	–4.9	6.0	–3.1	6.7	–3.4	–1.6	–5.3	0.0	–5.4	0.0
Hybrids										
B3LYP	3.1	10.6	3.5	10.4	1.7	3.8	–1.8	0.0	–0.7	0.0
B3LYP*	1.2	9.9	1.6	10.1	0.6	3.5	–3.4	0.0	–3.6	0.0
B1LYP	4.5	10.8	4.7	10.0	2.6	4.1	–0.4	0.0	1.6	0.0
B1PW91	0.1	–0.5	2.9	2.6	–0.6	–2.2	–3.8	0.0	–4.2	0.0
BHandH	–6.6	–18.8	–3.6	–18.9	1.1	–4.5	–2.6	0.0	–7.9	0.0
BHandHLYP	7.3	3.3	8.1	1.2	5.5	2.3	4.3	0.0	8.1	0.0
KMLYP	–0.3	–12.7	2.5	–13.0	3.4	–3.0	1.1	0.0	–0.2	0.0
O3LYP	–21.2	–27.6	–16.9	–23.3	–5.7	–7.8	–13.8	0.0	–28.5	0.0
OPBE0	1.9	–6.0	6.3	0.2	–2.7	–7.8	–5.1	0.0	–4.9	0.0
PBE0	–3.0	–4.6	0.1	–1.4	–1.2	–3.0	–4.6	0.0	–7.2	0.0
mPW1PW	–0.8	–0.9	1.9	1.8	–0.4	–1.7	–3.5	0.0	–4.7	0.0
mPW1K	1.3	–6.2	4.5	–4.2	1.6	–3.2	–0.3	0.0	–0.1	0.0
S12H	–3.1	–4.8	1.1	0.6	–0.2	–3.1	–2.6	0.0	–6.0	0.0
X3LYP	2.4	9.0	2.8	8.6	1.8	3.6	–1.7	0.0	–1.1	0.0
Meta-Hybrids										
M06	–0.1	0.3	4.1	6.6	0.3	–1.1	–1.8	0.0	–1.9	0.0
M06-2X	–1.8	–1.3	2.6	–1.0	1.3	–0.3	–0.5	0.0	–1.5	0.0
M06-HF	–4.8	–1.1	0.7	–12.2	–0.3	1.0	0.8	0.0	0.6	0.0
TPSSH	–2.5	5.1	–0.5	6.7	–1.8	–0.9	–4.3	0.0	–5.0	0.0
Double-Hybrids										
B2K-PLYP	–1.2	–1.1	0.2	3.9	1.5	0.7	–1.1	0.0	–1.3	0.0
B2T-PLYP	–0.3	2.3	0.8	6.3	1.4	1.6	–1.4	0.0	–1.2	0.0
B2-PLYP	–0.3	4.3	0.5	8.3	1.0	2.2	–2.0	0.0	–2.0	0.0
LS1-TPSS	–5.3	–8.3	–2.2	–0.2	–0.3	–2.8	–3.0	0.0	–4.9	0.0
mPW2K-PLYP	–1.6	–1.2	–0.2	3.6	1.6	0.9	–1.0	0.0	–1.4	0.0
mPW2-PLYP	0.0	3.2	0.9	6.3	1.8	2.3	–1.0	0.0	–0.8	0.0
PBE0-DH	–2.5	–8.7	0.9	–4.3	0.0	–3.9	–2.6	0.0	–4.4	0.0
revDSD-BLYP	–2.4	–1.7	–0.7	3.4	1.7	1.1	–0.1	0.0	–1.1	0.0
revDSD-PBE	–3.1	–4.6	–0.8	1.8	0.5	–1.3	–1.0	0.0	–3.1	0.0
revDSD-PBEP86	–0.9	–1.9	0.9	3.9	1.3	–0.4	0.1	0.0	–0.9	0.0
Range-Separated Hybrids										
CAM-B3LYP	4.1	2.6	3.7	1.3	2.9	1.3	0.5	0.0	1.3	0.0
CAMY-B3LYP	2.1	3.6	2.3	2.7	2.2	1.9	–0.7	0.0	–0.6	0.0
ωB97	3.7	–7.8	4.2	–5.8	3.4	–4.5	3.5	0.0	3.1	0.0
ωB97X	3.2	–4.2	3.8	–3.1	2.9	–2.4	2.0	0.0	1.6	0.0
ωB97X-D	3.6	0.3	4.6	1.3	1.6	–1.7	–0.4	0.0	0.0	0.0

Table S23 (continued).

XC	Diels-Alder Reaction		1,3-dipolar Cycloaddition		Electrocyclic Rearrangement		Sigmatropic Rearrangement		Double Group Transfer	
	$\Delta E^{\ddagger}$	ΔE_{rxn}	$\Delta E^{\ddagger}$	ΔE_{rxn}	$\Delta E^{\ddagger}$	ΔE_{rxn}	$\Delta E^{\ddagger}$	ΔE_{rxn}	$\Delta E^{\ddagger}$	ΔE_{rxn}
Dispersion-Corrected										
BP86-D3(BJ)	–10.6	1.2	–7.0	5.0	–3.7	0.6	–9.1	0.0	–15.5	0.0
BLYP-D3(BJ)	–6.3	11.7	–4.4	13.1	–0.9	5.9	–5.8	0.0	–9.3	0.0
PBE-D3(BJ)	–10.5	–1.2	–6.9	3.5	–4.3	–1.1	–9.6	0.0	–16.6	0.0
OLYP-D3(BJ)	–11.8	–1.8	–5.9	4.1	–3.9	–0.7	–9.2	0.0	–13.9	0.0
OPBE-D3(BJ)	–16.2	–13.6	–7.4	–3.6	–7.2	–7.5	–12.5	0.0	–20.2	0.0
B3LYP-D3(BJ)	–3.3	5.4	–1.0	6.1	1.3	3.9	–2.4	0.0	–4.3	0.0
PBE0-D3(BJ)	–10.1	–0.8	–6.6	3.8	–4.2	–1.1	–9.5	0.0	–16.3	0.0
M06-2X-D3	–1.9	–1.4	2.5	–1.0	1.3	–0.3	–0.4	0.0	–1.4	0.0

Table S24. Mean error (ME), mean absolute error (MAE), maximum unsigned error (MUE) and standard deviation (SD) of reaction barriers and energies (kcal mol^{-1}) for the various pericyclic reactions, computed with XC/TZ2P//BP86/DZP compared to CCSDT(Q)/CBS//CCSD(T)/cc-pVTZ.

XC	$\Delta E^{\ddagger}$				ΔE_{rxn}				Total			
	ME	MAE	MUE	SD	ME	MAE	MUE	SD	ME	MAE	MUE	SD
					LDA							
VWN	–16.7	16.7	28.2	6.9	–11.6	11.6	17.0	4.7	–14.8	14.8	28.2	6.7
					GGAs							
BP86	–6.3	6.3	12.1	3.5	5.1	5.1	8.8	3.5	–2.0	5.8	12.1	3.6
BLYP	–1.5	2.6	5.0	1.9	14.1	14.1	18.3	5.8	4.3	6.9	18.3	6.7
BEE	–5.7	5.7	11.7	3.9	4.6	5.6	9.9	3.4	–1.9	5.7	11.7	3.7
PW91	–7.8	7.8	14.4	3.8	2.4	2.7	5.7	2.2	–4.0	5.9	14.4	4.1
PBE	–7.7	7.7	14.3	3.8	2.3	3.1	6.0	2.1	–3.9	5.9	14.3	4.0
PBEsol	–12.6	12.6	21.8	5.2	–5.7	5.7	8.6	2.1	–10.0	10.0	21.8	5.5
RPBE	–3.6	4.0	8.4	3.1	8.3	8.3	14.0	5.7	0.9	5.6	14.0	4.7
revPBE	–3.9	4.3	8.9	3.3	7.7	7.7	13.4	5.6	0.4	5.6	13.4	4.6
mPBE	–6.6	6.6	12.7	3.6	4.0	4.4	8.2	3.1	–2.6	5.7	12.7	3.6
mPW	–5.7	5.7	11.2	3.4	5.6	5.6	10.0	3.9	–1.4	5.6	11.2	3.6
HTBS	–7.8	7.8	14.7	4.4	1.3	3.4	5.9	1.9	–4.4	6.1	14.7	4.2
OLYP	–0.5	4.7	6.0	1.2	9.5	9.8	16.4	6.8	3.3	6.6	16.4	4.9
OPBE	–4.5	6.2	11.1	4.0	0.4	5.2	8.1	3.5	–2.7	5.8	11.1	3.8
XLYP	–1.1	2.4	4.4	1.7	15.0	15.0	19.3	5.9	4.9	7.1	19.3	7.2
					Meta–GGAs							
M06-L	–1.5	2.3	4.2	1.3	4.5	5.6	12.4	4.8	0.7	3.5	12.4	3.5
MVS	–6.0	6.0	13.5	5.1	–1.4	4.3	6.7	1.9	–4.3	5.3	13.5	4.3
TPSS	–4.4	4.4	7.3	1.9	6.0	6.0	9.9	4.3	–0.5	5.0	9.9	3.1
revTPSS	–4.4	4.4	5.4	1.0	3.7	4.7	6.7	2.3	–1.4	4.5	6.7	1.6
					Hybrids							
B3LYP	1.2	2.2	3.5	1.0	8.2	8.2	10.6	3.2	3.8	4.4	10.6	3.6
B3LYP*	–0.7	2.1	3.6	1.2	7.8	7.8	10.1	3.1	2.5	4.2	10.1	3.5
B1LYP	2.6	2.8	4.7	1.7	8.3	8.3	10.8	3.0	4.7	4.8	10.8	3.5
B1PW91	–1.1	2.3	4.2	1.7	0.0	1.7	2.6	0.9	–0.7	2.1	4.2	1.5
BHandH	–3.9	4.3	7.9	2.5	–14.0	14.0	18.9	6.8	–7.7	8.0	18.9	6.6
BHandHLYP	6.7	6.7	8.1	1.5	2.2	2.2	3.3	0.9	5.0	5.0	8.1	2.5
KMLYP	1.3	1.5	3.4	1.2	–9.5	9.5	13.0	4.7	–2.8	4.5	13.0	4.9
O3LYP	–17.2	17.2	28.5	7.6	–19.5	19.5	27.6	8.5	–18.1	18.1	28.5	8.0
OPBE0	–0.9	4.2	6.3	1.6	–4.5	4.6	7.8	3.2	–2.3	4.4	7.8	2.4
PBE0	–3.2	3.2	7.2	2.5	–3.0	3.0	4.6	1.3	–3.1	3.1	7.2	2.1
mPW1PW	–1.5	2.3	4.7	1.6	–0.2	1.4	1.8	0.4	–1.0	1.9	4.7	1.4
mPW1K	1.4	1.6	4.5	1.6	–4.5	4.5	6.2	1.2	–0.8	2.7	6.2	2.0
S12H	–2.2	2.6	6.0	2.0	–2.4	2.8	4.8	1.7	–2.3	2.7	6.0	1.9
X3LYP	0.8	2.0	2.8	0.6	7.0	7.0	9.0	2.5	3.2	3.9	9.0	2.9
					Meta–Hybrids							
M06	0.1	1.6	4.1	1.5	1.9	2.6	6.6	2.8	0.8	2.0	6.6	2.1
M06-2X	0.0	1.5	2.6	0.7	–0.8	0.8	1.3	0.4	–0.3	1.3	2.6	0.7
M06-HF	–0.6	1.4	4.8	1.7	–4.1	4.7	12.2	5.3	–1.9	2.7	12.2	3.9
TPSSH	–2.8	2.8	5.0	1.6	3.6	4.2	6.7	2.4	–0.4	3.3	6.7	2.1
					Double–Hybrids							
B2K-PLYP	–0.4	1.1	1.5	0.4	1.2	1.9	3.9	1.4	0.2	1.4	3.9	1.0
B2T-PLYP	–0.1	1.0	1.4	0.4	3.4	3.4	6.3	2.1	1.2	1.9	6.3	1.7
B2-PLYP	–0.6	1.2	2.0	0.7	4.9	4.9	8.3	2.5	1.5	2.6	8.3	2.5
LS1-TPSS	–3.1	3.1	5.3	1.8	–3.7	3.7	8.3	3.3	–3.4	3.4	8.3	2.5
mPW2K-PLYP	–0.5	1.1	1.6	0.5	1.1	1.9	3.6	1.2	0.1	1.4	3.6	0.9
mPW2-PLYP	0.2	0.9	1.8	0.5	3.9	3.9	6.3	1.7	1.6	2.0	6.3	1.8
PBE0-DH	–1.7	2.1	4.4	1.5	–5.6	5.6	8.7	2.2	–3.2	3.4	8.7	2.5
revDSD-BLYP	–0.5	1.2	2.4	0.8	0.9	2.0	3.4	1.0	0.0	1.5	3.4	1.0
revDSD-PBE	–1.5	1.7	3.1	1.2	–1.3	2.5	4.6	1.5	–1.4	2.0	4.6	1.3
revDSD-PBEP86	0.1	0.8	1.3	0.4	0.6	2.0	3.9	1.4	0.3	1.3	3.9	1.1
					Range-Separated Hybrids							
CAM-B3LYP	2.5	2.5	4.1	1.4	1.7	1.7	2.6	0.6	2.2	2.2	4.1	1.2
CAMY-B3LYP	1.1	1.6	2.3	0.8	2.7	2.7	3.6	0.7	1.7	2.0	3.6	0.9
ωB97	3.6	3.6	4.2	0.4	–6.0	6.0	7.8	1.4	0.0	4.5	7.8	1.5
ωB97X	2.7	2.7	3.8	0.8	–3.2	3.2	4.2	0.7	0.5	2.9	4.2	0.8
ωB97X-D	1.9	2.0	4.6	1.8	0.0	1.1	1.7	0.6	1.2	1.7	4.6	1.5

Table S24 (continued).

XC	$\Delta E^{\ddagger}$				ΔE_{rxn}				Total			
	ME	MAE	MUE	SD	ME	MAE	MUE	SD	ME	MAE	MUE	SD
					Dispersion–Corrected							
BP86-D3(BJ)	–9.2	9.2	15.5	3.9	2.2	2.2	5.0	2.0	–4.9	6.6	15.5	4.7
BLYP-D3(BJ)	–5.3	5.3	9.3	2.7	10.2	10.2	13.1	3.1	0.5	7.2	13.1	3.7
PBE-D3(BJ)	–9.6	9.6	16.6	4.1	0.4	1.9	3.5	1.1	–5.8	6.7	16.6	5.0
OLYP-D3(BJ)	–8.9	8.9	13.9	3.7	0.5	2.2	4.1	1.4	–5.4	6.4	13.9	4.5
OPBE-D3(BJ)	–12.7	12.7	20.2	5.0	–8.2	8.2	13.6	4.1	–11.0	11.0	20.2	5.2
B3LYP-D3(BJ)	–1.9	2.4	4.3	1.2	5.1	5.1	6.1	0.9	0.7	3.4	6.1	1.7
PBE0-D3(BJ)	–9.3	9.3	16.3	4.1	0.7	1.9	3.8	1.4	–5.6	6.5	16.3	4.9
M06-2X-D3	0.0	1.5	2.5	0.7	–0.9	0.9	1.4	0.5	–0.3	1.3	2.5	0.7

Table S25. Reaction barriers and reaction energies (kcal mol–1) for the various pericyclic reactions, computed with XC/QZ4P//BP86/DZP.

XC	Diels-Alder Reaction		1,3-dipolar Cycloaddition		Electrocyclic Rearrangement		Sigmatropic Rearrangement		Double Group Transfer	
	$\Delta E^{\ddagger}$	ΔE_{rxn}	$\Delta E^{\ddagger}$	ΔE_{rxn}	$\Delta E^{\ddagger}$	ΔE_{rxn}	$\Delta E^{\ddagger}$	ΔE_{rxn}	$\Delta E^{\ddagger}$	ΔE_{rxn}
LDA										
VWN	0.7	–65.1	3.1	–42.1	36.3	2.4	21.9	0.0	22.0	0.0
GGAs										
BP86	15.1	–42.3	15.4	–20.7	39.6	8.2	28.2	0.0	38.1	0.0
BLYP	21.0	–30.2	19.3	–11.0	42.7	13.8	31.8	0.0	45.2	0.0
BEE	16.0	–43.1	17.2	–19.9	38.8	6.3	28.0	0.0	38.4	0.0
PW91	12.3	–46.2	13.3	–24.0	39.2	7.3	27.8	0.0	35.8	0.0
PBE	12.8	–46.3	13.9	–23.7	38.9	6.7	27.6	0.0	35.8	0.0
PBEsol	6.6	–56.9	8.8	–33.6	36.7	3.2	24.0	0.0	28.4	0.0
RPBE	18.7	–38.1	19.2	–15.7	39.9	8.2	29.8	0.0	41.7	0.0
revPBE	18.4	–39.0	19.1	–16.3	39.7	7.8	29.3	0.0	41.2	0.0
mPBE	14.4	–44.1	15.3	–21.5	39.2	7.1	28.2	0.0	37.4	0.0
mPW	15.5	–41.9	16.2	–19.7	39.8	8.3	28.8	0.0	39.0	0.0
HTBS	13.8	–47.1	15.5	–24.0	37.4	4.6	26.1	0.0	35.5	0.0
OLYP	24.0	–36.0	24.0	–13.5	40.4	7.1	30.7	0.0	45.4	0.0
OPBE	19.3	–48.5	22.0	–22.3	36.7	0.0	26.9	0.0	38.9	0.0
XLYP	21.3	–28.9	19.4	–10.2	43.1	14.6	32.4	0.0	46.0	0.0
Meta-GGAs										
M06-L	17.0	–45.9	20.1	–17.1	43.6	5.9	34.0	0.0	45.9	0.0
MVS	12.8	–54.2	18.6	–24.6	42.7	5.9	28.3	0.0	36.7	0.0
TPSS	15.6	–40.3	16.0	–19.8	40.2	7.7	30.9	0.0	42.9	0.0
revTPSS	14.6	–42.3	15.1	–22.9	39.6	6.1	31.5	0.0	44.9	0.0
Hybrids										
B3LYP	22.7	–37.5	21.8	–18.9	44.8	11.8	35.1	0.0	49.6	0.0
B3LYP*	20.8	–38.2	19.9	–19.2	43.8	11.5	33.5	0.0	46.7	0.0
B1LYP	24.2	–37.2	23.0	–19.2	45.7	12.1	36.4	0.0	51.9	0.0
B1PW91	19.7	–48.4	21.3	–26.8	42.4	5.7	33.1	0.0	46.1	0.0
BHandH	13.2	–66.2	14.9	–48.0	44.3	3.6	34.3	0.0	42.5	0.0
BHandHLYP	27.2	–44.3	26.6	–27.8	48.7	10.4	41.3	0.0	58.5	0.0
KMLYP	19.6	–60.1	21.0	–42.1	46.6	5.2	38.1	0.0	50.2	0.0
O3LYP	–1.7	–75.5	1.4	–52.8	37.4	0.3	23.0	0.0	21.8	0.0
OPBE0	21.4	–54.2	24.5	–29.8	40.0	–0.2	31.7	0.0	45.3	0.0
PBE0	16.6	–52.5	18.4	–30.8	41.8	4.9	32.2	0.0	43.0	0.0
mPW1PW	18.8	–48.8	20.2	–27.6	42.7	6.3	33.3	0.0	45.6	0.0
mPW1K	21.1	–53.9	23.0	–33.5	44.6	4.8	36.6	0.0	50.3	0.0
S12H	16.5	–53.1	19.4	–29.1	42.8	4.5	34.2	0.0	44.1	0.0
X3LYP	22.0	–39.0	21.1	–20.7	44.9	11.6	35.2	0.0	49.2	0.0
Meta-Hybrids										
M06	19.6	–47.6	22.6	–22.7	43.4	7.0	35.0	0.0	48.4	0.0
M06-2X	18.0	–48.6	21.1	–29.7	44.5	7.9	36.5	0.0	49.0	0.0
M06-HF	14.3	–48.2	18.4	–40.7	42.9	9.6	37.6	0.0	50.7	0.0
TPSSH	17.0	–43.2	17.7	–22.8	41.2	6.9	32.5	0.0	45.2	0.0
Double-Hybrids										
B2K-PLYP	17.9	–48.9	17.7	–25.2	44.1	8.5	35.3	0.0	49.2	0.0
B2T-PLYP	18.9	–45.7	18.4	–22.8	44.1	9.5	35.1	0.0	49.3	0.0
B2-PLYP	18.9	–43.7	18.3	–20.8	43.8	10.1	34.5	0.0	48.5	0.0
LS1-TPSS	13.6	–56.4	14.9	–29.5	42.0	4.8	33.2	0.0	45.5	0.0
mPW2K-PLYP	17.6	–49.1	17.3	–25.5	44.2	8.7	35.4	0.0	49.0	0.0
mPW2-PLYP	19.4	–44.6	18.7	–22.8	44.6	10.2	35.6	0.0	49.6	0.0
PBE0-DH	17.0	–56.5	18.9	–33.6	42.8	4.0	34.1	0.0	46.1	0.0
revDSD-BLYP	17.1	–49.1	17.0	–25.3	44.3	9.0	36.3	0.0	49.6	0.0
revDSD-PBE	16.4	–51.9	17.0	–26.9	43.1	6.7	35.5	0.0	47.8	0.0
revDSD-PBEP86	18.6	–49.2	18.7	–24.7	43.9	7.6	36.6	0.0	50.0	0.0
Range-Separated Hybrids										
CAM-B3LYP	23.8	–45.2	22.1	–27.8	46.0	9.3	37.3	0.0	51.7	0.0
CAMY-B3LYP	21.8	–44.3	20.7	–26.4	45.3	10.0	36.1	0.0	49.8	0.0
ωB97	23.5	–36.0	22.7	–35.3	46.4	3.2	40.3	0.0	53.2	0.0
ωB97X	23.8	–45.2	22.1	–27.8	46.0	9.3	37.3	0.0	51.7	0.0
ωB97X-D	23.2	–47.5	23.0	–28.0	44.6	6.4	36.5	0.0	50.3	0.0

Table S25 (continued).

XC	Diels-Alder Reaction		1,3-dipolar Cycloaddition		Electrocyclic Rearrangement		Sigmatropic Rearrangement		Double Group Transfer	
	$\Delta E^{\ddagger}$	ΔE_{rxn}	$\Delta E^{\ddagger}$	ΔE_{rxn}	$\Delta E^{\ddagger}$	ΔE_{rxn}	$\Delta E^{\ddagger}$	ΔE_{rxn}	$\Delta E^{\ddagger}$	ΔE_{rxn}
Dispersion-Corrected										
BP86-D3(BJ)	8.8	–47.2	11.2	–24.6	39.4	8.4	27.7	0.0	34.7	0.0
BLYP-D3(BJ)	13.1	–36.6	13.8	–16.2	42.3	13.9	31.0	0.0	40.9	0.0
PBE-D3(BJ)	8.9	–49.5	11.2	–26.2	38.7	6.8	27.2	0.0	33.6	0.0
OLYP-D3(BJ)	7.6	–50.2	12.2	–25.8	39.0	6.9	27.5	0.0	36.1	0.0
OPBE-D3(BJ)	3.1	–62.3	10.4	–34.0	35.5	–0.1	24.2	0.0	29.8	0.0
B3LYP-D3(BJ)	16.2	–42.7	17.3	–23.1	44.5	11.9	34.4	0.0	46.0	0.0
PBE0-D3(BJ)	9.3	–49.1	11.5	–25.9	38.8	6.8	27.3	0.0	33.8	0.0
M06-2X-D3	17.9	–48.7	21.0	–29.8	44.5	7.9	36.6	0.0	48.9	0.0

Table S26. Errors in reaction barriers and reaction energies (kcal mol^{-1}) for the various pericyclic reactions, computed with XC/QZ4P//BP86/DZP, compared to CCSDT(Q)/CBS//CCSD(T)/cc-pVTZ.

XC	Diels-Alder Reaction		1,3-dipolar Cycloaddition		Electrocyclic Rearrangement		Sigmatropic Rearrangement		Double Group Transfer	
	$\Delta E^{\ddagger}$	ΔE_{rxn}	$\Delta E^{\ddagger}$	ΔE_{rxn}	$\Delta E^{\ddagger}$	ΔE_{rxn}	$\Delta E^{\ddagger}$	ΔE_{rxn}	$\Delta E^{\ddagger}$	ΔE_{rxn}
LDA										
VWN	–18.9	–17.5	–15.2	–13.2	–7.1	–6.1	–14.9	0.0	–28.2	0.0
GGAs										
BP86	–4.5	5.4	–2.9	8.2	–3.8	–0.3	–8.6	0.0	–12.1	0.0
BLYP	1.4	17.5	1.0	17.9	–0.7	5.4	–5.0	0.0	–5.0	0.0
BEE	–3.6	4.6	–1.1	9.0	–4.6	–2.2	–8.8	0.0	–11.8	0.0
PW91	–7.3	1.5	–5.0	4.9	–4.2	–1.2	–9.0	0.0	–14.4	0.0
PBE	–6.8	1.4	–4.4	5.2	–4.5	–1.8	–9.2	0.0	–14.4	0.0
PBEsol	–13.0	–9.3	–9.5	–4.7	–6.7	–5.3	–12.8	0.0	–21.8	0.0
RPBE	–0.9	9.6	0.9	13.2	–3.5	–0.3	–7.0	0.0	–8.5	0.0
revPBE	–1.2	8.7	0.8	12.6	–3.7	–0.6	–7.5	0.0	–9.0	0.0
mPBE	–5.2	3.6	–3.0	7.4	–4.2	–1.4	–8.6	0.0	–12.8	0.0
mPW	–4.1	5.8	–2.1	9.2	–3.6	–0.1	–8.0	0.0	–11.2	0.0
HTBS	–5.8	0.5	–2.8	4.9	–6.0	–3.9	–10.7	0.0	–14.7	0.0
OLYP	4.4	11.7	5.7	15.4	–3.0	–1.4	–6.1	0.0	–4.8	0.0
OPBE	–0.3	–0.9	3.7	6.6	–6.7	–8.5	–9.9	0.0	–11.3	0.0
XLYP	1.7	18.8	1.1	18.7	–0.3	6.2	–4.4	0.0	–4.2	0.0
Meta-GGAs										
M06-L	–2.6	1.8	1.8	11.8	0.2	–2.6	–2.8	0.0	–4.3	0.0
MVS	–6.8	–6.6	0.3	4.3	–0.7	–2.6	–8.5	0.0	–13.5	0.0
TPSS	–4.0	7.4	–2.3	9.1	–3.2	–0.7	–5.9	0.0	–7.3	0.0
revTPSS	–5.0	5.4	–3.2	6.0	–3.8	–2.4	–5.3	0.0	–5.3	0.0
Hybrids										
B3LYP	3.1	10.2	3.5	10.0	1.4	3.4	–1.7	0.0	–0.6	0.0
B3LYP*	1.2	9.5	1.6	9.7	0.4	3.1	–3.3	0.0	–3.5	0.0
B1LYP	4.6	10.5	4.7	9.7	2.3	3.7	–0.4	0.0	1.7	0.0
B1PW91	0.1	–0.8	3.0	2.1	–1.0	–2.8	–3.7	0.0	–4.1	0.0
BHandH	–6.4	–18.6	–3.4	–19.1	0.9	–4.9	–2.5	0.0	–7.7	0.0
BHandHLYP	7.6	3.4	8.3	1.1	5.3	2.0	4.5	0.0	8.3	0.0
KMLYP	0.0	–12.5	2.7	–13.2	3.2	–3.3	1.3	0.0	0.0	0.0
O3LYP	–21.3	–27.9	–16.9	–23.9	–6.0	–8.2	–13.8	0.0	–28.4	0.0
OPBE0	1.8	–6.6	6.2	–0.9	–3.4	–8.7	–5.1	0.0	–4.9	0.0
PBE0	–3.0	–4.9	0.1	–1.9	–1.6	–3.6	–4.6	0.0	–7.2	0.0
mPW1PW	–0.8	–1.2	1.9	1.3	–0.7	–2.2	–3.5	0.0	–4.6	0.0
mPW1K	1.5	–6.3	4.7	–4.6	1.2	–3.7	–0.2	0.0	0.1	0.0
S12H	–3.1	–5.5	1.1	–0.2	–0.6	–4.0	–2.6	0.0	–6.1	0.0
X3LYP	2.4	8.7	2.8	8.2	1.5	3.2	–1.6	0.0	–1.0	0.0
Meta-Hybrids										
M06	0.0	0.0	4.3	6.2	0.0	–1.5	–1.8	0.0	–1.8	0.0
M06-2X	–1.6	–1.0	2.8	–0.8	1.1	–0.5	–0.3	0.0	–1.2	0.0
M06-HF	–5.3	–0.6	0.1	–11.8	–0.5	1.2	0.8	0.0	0.5	0.0
TPSSH	–2.6	4.5	–0.6	6.1	–2.2	–1.6	–4.3	0.0	–5.0	0.0
Double-Hybrids										
B2K-PLYP	–1.7	–1.3	–0.6	3.7	0.7	0.1	–1.5	0.0	–1.0	0.0
B2T-PLYP	–0.7	2.0	0.1	6.1	0.7	1.1	–1.7	0.0	–0.9	0.0
B2-PLYP	–0.7	4.0	0.0	8.1	0.4	1.7	–2.3	0.0	–1.7	0.0
LS1-TPSS	–6.0	–8.8	–3.4	–0.6	–1.4	–3.7	–3.6	0.0	–4.7	0.0
mPW2K-PLYP	–2.0	–1.5	–1.0	3.4	0.8	0.3	–1.4	0.0	–1.2	0.0
mPW2-PLYP	–0.2	3.1	0.4	6.1	1.2	1.8	–1.2	0.0	–0.6	0.0
PBE0-DH	–2.6	–8.9	0.6	–4.7	–0.6	–4.5	–2.7	0.0	–4.1	0.0
revDSD-BLYP	–2.5	–1.5	–1.3	3.6	0.9	0.6	–0.5	0.0	–0.6	0.0
revDSD-PBE	–3.2	–4.3	–1.3	2.0	–0.3	–1.8	–1.3	0.0	–2.4	0.0
revDSD-PBEP86	–1.0	–1.6	0.4	4.2	0.5	–0.9	–0.2	0.0	–0.2	0.0
Range-Separated Hybrids										
CAM-B3LYP	4.2	2.5	3.8	1.1	2.6	0.9	0.5	0.0	1.5	0.0
CAMY-B3LYP	2.2	3.4	2.4	2.5	1.9	1.6	–0.7	0.0	–0.4	0.0
ωB97	3.9	11.7	4.4	–6.4	3.0	–5.3	3.5	0.0	3.0	0.0
ωB97X	4.2	2.5	3.8	1.1	2.6	0.9	0.5	0.0	1.5	0.0
ωB97X-D	3.6	0.1	4.7	0.9	1.2	–2.1	–0.3	0.0	0.1	0.0

Table S26 (continued).

XC	Diels-Alder Reaction		1,3-dipolar Cycloaddition		Electrocyclic Rearrangement		Sigmatropic Rearrangement		Double Group Transfer	
	$\Delta E^{‡}$	ΔE_{rxn}	$\Delta E^{‡}$	ΔE_{rxn}	$\Delta E^{‡}$	ΔE_{rxn}	$\Delta E^{‡}$	ΔE_{rxn}	$\Delta E^{‡}$	ΔE_{rxn}
Dispersion-Corrected										
BP86-D3(BJ)	–10.8	0.4	–7.1	4.3	–4.0	0.0	–9.1	0.0	–15.5	0.0
BLYP-D3(BJ)	–6.5	11.1	–4.5	12.7	–1.1	5.5	–5.8	0.0	–9.3	0.0
PBE-D3(BJ)	–10.7	–1.9	–7.1	2.7	–4.7	–1.7	–9.6	0.0	–16.6	0.0
OLYP-D3(BJ)	–12.0	–2.6	–6.1	3.1	–4.4	–1.6	–9.3	0.0	–14.1	0.0
OPBE-D3(BJ)	–16.5	–14.7	–7.9	–5.1	–7.9	–8.6	–12.6	0.0	–20.4	0.0
B3LYP-D3(BJ)	–3.4	5.0	–1.0	5.8	1.1	3.5	–2.4	0.0	–4.2	0.0
PBE0-D3(BJ)	–10.3	–1.5	–6.8	3.0	–4.6	–1.7	–9.5	0.0	–16.4	0.0
M06-2X-D3	–1.7	–1.1	2.7	–0.9	1.1	–0.5	–0.2	0.0	–1.3	0.0

Table S27. Mean error (ME), mean absolute error (MAE), maximum unsigned error (MUE) and standard deviation (SD) of reaction barriers and energies (kcal mol^{-1}) for the various pericyclic reactions, computed with XC/QZ4P//BP86/DZP compared to CCSDT(Q)/CBS//CCSD(T)/cc-pVTZ.

XC	$\Delta E^{\ddagger}$				ΔE_{rxn}				Total			
	ME	MAE	MUE	SD	ME	MAE	MUE	SD	ME	MAE	MUE	SD
						LDA						
VWN	–16.9	16.9	28.2	6.8	–12.2	12.2	17.5	4.7	–15.1	15.1	28.2	6.5
						GGAs						
BP86	–6.4	6.4	12.1	3.5	4.4	4.6	8.2	3.3	–2.3	5.7	12.1	3.5
BLYP	–1.7	2.6	5.0	1.9	13.6	13.6	17.9	5.8	4.0	6.7	17.9	6.5
BEE	–6.0	6.0	11.8	3.8	3.8	5.2	9.0	2.8	–2.3	5.7	11.8	3.5
PW91	–8.0	8.0	14.4	3.6	1.7	2.5	4.9	1.7	–4.3	5.9	14.4	4.0
PBE	–7.9	7.9	14.4	3.7	1.6	2.8	5.2	1.7	–4.3	5.9	14.4	4.0
PBEsol	–12.8	12.8	21.8	5.1	–6.4	6.4	9.3	2.0	–10.4	10.4	21.8	5.2
RPBE	–3.8	4.2	8.5	3.1	7.5	7.7	13.2	5.4	0.4	5.5	13.2	4.5
revPBE	–4.1	4.4	9.0	3.3	6.9	7.3	12.6	5.0	0.0	5.5	12.6	4.2
mPBE	–6.8	6.8	12.8	3.6	3.2	4.1	7.4	2.5	–3.0	5.8	12.8	3.5
mPW	–5.8	5.8	11.2	3.3	4.9	5.0	9.2	3.7	–1.8	5.5	11.2	3.5
HTBS	–8.0	8.0	14.7	4.2	0.5	3.1	4.9	1.8	–4.8	6.2	14.7	4.2
OLYP	–0.8	4.8	6.1	1.1	8.6	9.5	15.4	5.9	2.7	6.6	15.4	4.3
OPBE	–4.9	6.4	11.3	4.0	–0.9	5.3	8.5	3.2	–3.4	6.0	11.3	3.8
XLYP	–1.2	2.4	4.4	1.7	14.5	14.5	18.8	5.9	4.7	6.9	18.8	7.0
						Meta–GGAs						
M06-L	–1.5	2.3	4.3	1.4	3.7	5.4	11.8	4.5	0.4	3.5	11.8	3.3
MVS	–5.8	6.0	13.5	5.0	–1.6	4.5	6.6	1.6	–4.3	5.4	13.5	4.1
TPSS	–4.5	4.5	7.3	1.8	5.2	5.7	9.1	3.6	–0.9	5.0	9.1	2.7
revTPSS	–4.5	4.5	5.3	0.9	3.0	4.6	6.0	1.6	–1.7	4.5	6.0	1.2
						Hybrids						
B3LYP	1.1	2.1	3.5	1.1	7.8	7.8	10.2	3.2	3.6	4.2	10.2	3.5
B3LYP*	–0.7	2.0	3.5	1.2	7.4	7.4	9.7	3.1	2.3	4.0	9.7	3.4
B1LYP	2.6	2.7	4.7	1.7	7.9	7.9	10.5	3.0	4.6	4.7	10.5	3.4
B1PW91	–1.1	2.4	4.1	1.5	–0.5	1.9	2.8	0.8	–0.9	2.2	4.1	1.4
BHandH	–3.8	4.2	7.7	2.5	–14.2	14.2	19.1	6.6	–7.7	7.9	19.1	6.6
BHandHLYP	6.8	6.8	8.3	1.6	2.1	2.1	3.4	0.9	5.0	5.0	8.3	2.7
KMLYP	1.4	1.5	3.2	1.3	–9.6	9.6	13.2	4.5	–2.7	4.5	13.2	5.0
O3LYP	–17.3	17.3	28.4	7.5	–20.0	20.0	27.9	8.5	–18.3	18.3	28.4	8.0
OPBE0	–1.1	4.3	6.2	1.5	–5.4	5.4	8.7	3.3	–2.7	4.7	8.7	2.4
PBE0	–3.3	3.3	7.2	2.5	–3.4	3.4	4.9	1.2	–3.3	3.4	7.2	2.1
mPW1PW	–1.5	2.3	4.6	1.5	–0.7	1.5	2.2	0.4	–1.2	2.0	4.6	1.3
mPW1K	1.5	1.5	4.7	1.7	–4.8	4.8	6.3	1.1	–0.9	2.8	6.3	2.2
S12H	–2.3	2.7	6.1	1.9	–3.2	3.2	5.5	2.2	–2.6	2.9	6.1	2.0
X3LYP	0.8	1.9	2.8	0.7	6.7	6.7	8.7	2.5	3.0	3.7	8.7	2.8
						Meta–Hybrids						
M06	0.1	1.6	4.3	1.6	1.6	2.6	6.2	2.6	0.7	2.0	6.2	2.1
M06-2X	0.2	1.4	2.8	0.8	–0.8	0.8	1.0	0.2	–0.2	1.2	2.8	0.7
M06-HF	–0.9	1.4	5.3	1.9	–3.7	4.5	11.8	5.2	–2.0	2.6	11.8	3.8
TPSSH	–2.9	2.9	5.0	1.6	3.0	4.0	6.1	1.9	–0.7	3.3	6.1	1.8
						Double–Hybrids						
B2K-PLYP	–0.8	1.1	1.7	0.4	0.8	1.7	3.7	1.5	–0.2	1.3	3.7	1.0
B2T-PLYP	–0.5	0.8	1.7	0.5	3.0	3.0	6.1	2.2	0.8	1.6	6.1	1.8
B2-PLYP	–0.9	1.0	2.3	0.9	4.6	4.6	8.1	2.7	1.2	2.3	8.1	2.5
LS1-TPSS	–3.8	3.8	6.0	1.5	–4.3	4.3	8.8	3.3	–4.0	4.0	8.8	2.4
mPW2K-PLYP	–1.0	1.3	2.0	0.4	0.7	1.7	3.4	1.3	–0.3	1.4	3.4	0.9
mPW2-PLYP	–0.1	0.7	1.2	0.4	3.6	3.6	6.1	1.8	1.3	1.8	6.1	1.8
PBE0-DH	–1.9	2.1	4.1	1.3	–6.0	6.0	8.9	2.0	–3.4	3.6	8.9	2.5
revDSD-BLYP	–0.8	1.1	2.5	0.7	0.9	1.9	3.6	1.3	–0.2	1.4	3.6	1.0
revDSD-PBE	–1.7	1.7	3.2	1.0	–1.3	2.7	4.3	1.1	–1.6	2.1	4.3	1.1
revDSD-PBEP86	–0.1	0.4	1.0	0.3	0.6	2.2	4.2	1.4	0.2	1.1	4.2	1.2
						Range-Separated Hybrids						
CAM-B3LYP	2.5	2.5	4.2	1.4	1.5	1.5	2.5	0.7	2.1	2.1	4.2	1.3
CAMY-B3LYP	1.1	1.5	2.4	0.8	2.5	2.5	3.4	0.7	1.6	1.9	3.4	0.9
ωB97	3.6	3.6	4.4	0.6	0.0	7.8	11.7	2.8	2.2	5.1	11.7	2.7
ωB97X	2.5	2.5	4.2	1.4	1.5	1.5	2.5	0.7	2.1	2.1	4.2	1.3
ωB97X-D	1.9	2.0	4.7	1.9	–0.3	1.0	2.1	0.8	1.0	1.6	4.7	1.6

Table S27 (continued).

XC	$\Delta E^{\ddagger}$				ΔE_{rxn}				Total			
	ME	MAE	MUE	SD	ME	MAE	MUE	SD	ME	MAE	MUE	SD
					Dispersion–Corrected							
BP86-D3(BJ)	–9.3	9.3	15.5	3.8	1.6	1.6	4.3	1.9	–5.2	6.4	15.5	4.9
BLYP-D3(BJ)	–5.4	5.4	9.3	2.7	9.7	9.7	12.7	3.1	0.2	7.0	12.7	3.5
PBE-D3(BJ)	–9.7	9.7	16.6	4.0	–0.3	2.1	2.7	0.4	–6.2	6.9	16.6	4.9
OLYP-D3(BJ)	–9.2	9.2	14.1	3.6	–0.3	2.4	3.1	0.6	–5.9	6.6	14.1	4.4
OPBE-D3(BJ)	–13.1	13.1	20.4	4.9	–9.4	9.4	14.7	3.9	–11.7	11.7	20.4	4.9
B3LYP-D3(BJ)	–2.0	2.4	4.2	1.3	4.7	4.7	5.8	1.0	0.5	3.3	5.8	1.6
PBE0-D3(BJ)	–9.5	9.5	16.4	4.0	0.0	2.0	3.0	0.7	–6.0	6.7	16.4	4.8
M06-2X-D3	0.1	1.4	2.7	0.8	–0.8	0.8	1.1	0.2	–0.2	1.2	2.7	0.7

Table S28. Reaction barriers and reaction energies (kcal mol^{-1}) for the various pericyclic reactions, computed with XC/TZ2P//BP86/TZ2P.

XC	Diels-Alder Reaction		1,3-dipolar Cycloaddition		Electrocyclic Rearrangement		Sigmatropic Rearrangement		Double Group Transfer	
	$\Delta E^{\ddagger}$	ΔE_{rxn}	$\Delta E^{\ddagger}$	ΔE_{rxn}	$\Delta E^{\ddagger}$	ΔE_{rxn}	$\Delta E^{\ddagger}$	ΔE_{rxn}	$\Delta E^{\ddagger}$	ΔE_{rxn}
LDA										
VWN	1.0	–64.5	3.3	–41.5	36.6	3.0	22.0	0.0	22.1	0.0
GGAs										
BP86	15.2	–41.6	15.5	–20.1	39.9	8.8	28.2	0.0	38.1	0.0
BLYP	21.2	–29.6	19.4	–10.6	43.0	14.3	31.9	0.0	45.2	0.0
BEE	16.4	–42.3	17.4	–19.1	39.3	7.1	28.1	0.0	38.5	0.0
PW91	12.6	–45.5	13.5	–23.3	39.6	8.0	27.9	0.0	35.8	0.0
PBE	13.1	–45.6	14.1	–22.9	39.3	7.4	27.6	0.0	35.9	0.0
PBEsol	6.9	–56.1	9.1	–32.8	37.1	3.8	24.0	0.0	28.4	0.0
RPBE	19.0	–37.3	19.4	–14.9	40.1	9.0	29.9	0.0	41.8	0.0
revPBE	18.7	–38.1	19.2	–15.5	40.1	8.5	29.5	0.0	41.3	0.0
mPBE	14.6	–43.3	15.5	–20.8	39.6	7.9	28.3	0.0	37.5	0.0
mPW	15.7	–41.2	16.3	–19.0	40.2	8.9	28.9	0.0	39.0	0.0
HTBS	14.2	–46.4	15.8	–23.1	37.9	5.4	26.1	0.0	35.5	0.0
OLYP	24.3	–35.1	24.2	–12.6	41.0	8.0	30.9	0.0	45.6	0.0
OPBE	19.8	–47.3	22.4	–21.0	37.4	1.2	27.1	0.0	39.1	0.0
XLYP	21.4	–28.4	19.4	–9.7	43.4	15.1	32.5	0.0	45.9	0.0
Meta-GGAs										
M06-L	16.8	–44.8	19.7	–16.6	43.8	6.9	34.2	0.0	46.1	0.0
MVS	12.2	–54.2	18.1	–24.5	43.1	6.5	28.2	0.0	36.7	0.0
TPSS	15.7	–39.5	16.1	–19.0	40.6	8.5	31.0	0.0	42.9	0.0
revTPSS	14.7	–41.7	15.1	–22.1	40.0	6.9	31.5	0.0	44.8	0.0
Hybrids										
B3LYP	22.6	–37.2	21.7	–18.6	45.1	12.2	35.1	0.0	49.5	0.0
B3LYP*	20.7	–37.8	19.9	–18.9	44.1	11.9	33.5	0.0	46.6	0.0
B1LYP	24.0	–37.0	22.9	–19.0	46.0	12.5	36.4	0.0	51.8	0.0
B1PW91	19.6	–48.1	21.2	–26.4	42.8	6.3	33.0	0.0	46.0	0.0
BHandH	12.8	–66.4	14.8	–47.9	44.4	3.9	34.0	0.0	42.4	0.0
BHandHLYP	26.6	–44.5	26.4	–27.7	48.8	10.6	41.0	0.0	58.3	0.0
KMLYP	19.0	–60.4	20.8	–42.0	46.7	5.4	37.8	0.0	50.1	0.0
O3LYP	–1.6	–75.1	1.5	–52.4	37.6	0.7	22.9	0.0	21.8	0.0
OPBE0	21.5	–53.6	24.6	–28.9	40.7	0.7	31.6	0.0	45.3	0.0
PBE0	16.6	–52.2	18.4	–30.3	42.2	5.5	32.1	0.0	43.0	0.0
mPW1PW	18.7	–48.5	20.2	–27.2	43.0	6.8	33.2	0.0	45.6	0.0
mPW1K	20.7	–53.9	22.8	–33.2	44.9	5.3	36.4	0.0	50.2	0.0
S12H	16.4	–52.5	19.4	–28.4	43.2	5.3	34.1	0.0	44.2	0.0
X3LYP	21.9	–38.8	21.0	–20.4	45.2	12.0	35.1	0.0	49.1	0.0
Meta-Hybrids										
M06	19.3	–47.4	22.3	–22.3	43.7	7.4	34.9	0.0	48.4	0.0
M06-2X	17.6	–49.0	21.0	–29.8	44.6	8.1	36.2	0.0	48.7	0.0
M06-HF	14.5	–48.9	19.4	–41.0	42.9	9.2	37.3	0.0	50.8	0.0
TPSSH	17.0	–42.6	17.7	–22.2	41.6	7.6	32.5	0.0	45.2	0.0
Double-Hybrids										
B2K-PLYP	18.1	–48.8	18.5	–25.1	44.8	8.6	35.6	0.0	49.0	0.0
B2T-PLYP	19.1	–45.5	19.0	–22.6	44.7	9.6	35.3	0.0	49.0	0.0
B2-PLYP	19.1	–43.5	18.8	–20.7	44.4	10.3	34.7	0.0	48.2	0.0
LS1-TPSS	14.2	–56.0	16.2	–29.2	43.0	5.0	33.6	0.0	45.3	0.0
mPW2K-PLYP	17.8	–48.9	18.1	–25.3	44.9	8.8	35.7	0.0	48.8	0.0
mPW2-PLYP	19.4	–44.5	19.1	–22.6	45.2	10.4	35.8	0.0	49.4	0.0
PBE0-DH	16.9	–56.4	19.1	–33.3	43.3	4.4	34.1	0.0	45.8	0.0
revDSD-BLYP	17.0	–49.3	17.6	–25.5	45.0	9.0	36.5	0.0	49.1	0.0
revDSD-PBE	16.3	–52.3	17.5	–27.1	43.8	6.8	35.7	0.0	47.1	0.0
revDSD-PBEP86	18.5	–49.6	19.2	–25.0	44.6	7.7	36.8	0.0	49.3	0.0
Range-Separated Hybrids										
CAM-B3LYP	23.4	–45.2	22.9	–27.6	46.2	9.6	37.2	0.0	51.6	0.0
CAMY-B3LYP	21.5	–44.2	20.6	–26.2	45.5	10.3	36.0	0.0	49.6	0.0
ωB97	23.0	–55.5	22.5	–34.7	46.7	4.0	40.1	0.0	53.3	0.0
ωB97X	22.5	–51.9	22.1	–32.0	46.2	6.0	38.7	0.0	51.8	0.0
ωB97X-D	23.0	–47.5	22.9	–27.7	45.0	6.8	36.4	0.0	50.3	0.0

Table S28 (continued).

XC	Diels-Alder Reaction		1,3-dipolar Cycloaddition		Electrocyclic Rearrangement		Sigmatropic Rearrangement		Double Group Transfer	
	$\Delta E^{\ddagger}$	ΔE_{rxn}	$\Delta E^{\ddagger}$	ΔE_{rxn}	$\Delta E^{\ddagger}$	ΔE_{rxn}	$\Delta E^{\ddagger}$	ΔE_{rxn}	$\Delta E^{\ddagger}$	ΔE_{rxn}
					Dispersion-Corrected					
BP86-D3(BJ)	9.0	–46.5	11.3	–24.0	39.7	9.0	27.7	0.0	34.7	0.0
BLYP-D3(BJ)	13.3	–36.0	13.9	–15.7	42.6	14.4	31.0	0.0	40.8	0.0
PBE-D3(BJ)	9.1	–48.7	11.4	–25.5	39.1	7.5	27.3	0.0	33.6	0.0
OLYP-D3(BJ)	7.9	–49.3	12.5	–24.9	39.5	7.8	27.7	0.0	36.3	0.0
OPBE-D3(BJ)	3.6	–61.1	11.0	–32.6	36.2	1.1	24.3	0.0	30.0	0.0
B3LYP-D3(BJ)	16.2	–42.4	17.3	–22.8	44.7	12.3	34.4	0.0	45.9	0.0
PBE0-D3(BJ)	13.1	–54.9	16.0	–32.6	42.1	5.6	31.9	0.0	41.0	0.0
M06-2X-D3	17.5	–49.1	20.9	–29.9	44.6	8.1	36.2	0.0	48.6	0.0

Table S29. Errors in reaction barriers and reaction energies (kcal mol^{-1}) for the various pericyclic reactions, computed with XC/TZ2P//BP86/TZ2P, compared to CCSDT(Q)/CBS//CCSD(T)/cc-pVTZ.

XC	Diels-Alder Reaction		1,3-dipolar Cycloaddition		Electrocyclic Rearrangement		Sigmatropic Rearrangement		Double Group Transfer	
	$\Delta E^{‡}$	ΔE_{rxn}	$\Delta E^{‡}$	ΔE_{rxn}	$\Delta E^{‡}$	ΔE_{rxn}	$\Delta E^{‡}$	ΔE_{rxn}	$\Delta E^{‡}$	ΔE_{rxn}
					LDA					
VWN	–18.6	–16.9	–15.0	–12.6	–6.8	–5.5	–14.8	0.0	–28.1	0.0
					GGAs					
BP86	–4.4	6.1	–2.8	8.8	–3.5	0.4	–8.6	0.0	–12.1	0.0
BLYP	1.6	18.1	1.1	18.3	–0.4	5.9	–4.9	0.0	–5.0	0.0
BEE	–3.2	5.4	–0.9	9.8	–4.1	–1.4	–9.7	0.0	–11.7	0.0
PW91	–7.0	2.2	–4.8	5.6	–3.8	–0.4	–8.9	0.0	–14.4	0.0
PBE	–6.5	2.1	–4.2	6.0	–4.1	–1.1	–9.2	0.0	–14.3	0.0
PBEsol	–12.7	–8.5	–9.2	–3.9	–6.3	–4.7	–12.8	0.0	–21.8	0.0
RPBE	–0.6	10.4	1.1	14.0	–3.3	0.6	–6.9	0.0	–8.4	0.0
revPBE	–0.9	9.6	0.9	13.4	–3.3	0.1	–7.3	0.0	–8.9	0.0
mPBE	–5.0	4.4	–2.8	8.1	–3.8	–0.5	–8.5	0.0	–12.7	0.0
mPW	–3.9	6.5	–2.0	9.9	–3.2	0.5	–7.9	0.0	–11.2	0.0
HTBS	–5.4	1.3	–2.5	5.8	–5.5	–3.1	–10.7	0.0	–14.7	0.0
OLYP	4.7	12.6	5.9	16.3	–2.4	–0.4	–5.9	0.0	–4.6	0.0
OPBE	0.2	0.4	4.1	7.9	–6.0	–7.3	–9.7	0.0	–11.1	0.0
XLYP	1.8	19.3	1.1	19.2	0.0	6.7	–4.3	0.0	–4.3	0.0
					Meta-GGAs					
M06-L	–2.8	2.9	1.4	12.3	0.4	–1.6	–2.6	0.0	–4.1	0.0
MVS	–7.4	–6.6	–0.2	4.4	–0.3	–2.0	–8.6	0.0	–13.5	0.0
TPSS	–3.9	8.2	–2.2	9.9	–2.8	0.1	–5.8	0.0	–7.3	0.0
revTPSS	–4.9	6.0	–3.2	6.8	–3.4	–1.6	–5.3	0.0	–5.4	0.0
					Hybrids					
B3LYP	3.0	10.5	3.4	10.3	1.7	3.8	–1.7	0.0	–0.7	0.0
B3LYP*	1.1	9.9	1.6	10.0	0.7	3.5	–3.3	0.0	–3.6	0.0
B1LYP	4.4	10.7	4.6	9.9	2.6	4.1	–0.4	0.0	1.6	0.0
B1PW91	0.0	–0.5	2.9	2.5	–0.6	–2.2	–3.8	0.0	–4.2	0.0
BHandH	–6.8	–18.8	–3.5	–19.0	1.0	–4.6	–2.8	0.0	–7.8	0.0
BHandHLYP	7.0	3.2	8.1	1.2	5.4	2.2	4.2	0.0	8.1	0.0
KMLYP	–0.6	–12.8	2.5	–13.1	3.3	–3.1	1.0	0.0	–0.1	0.0
O3LYP	–21.2	–27.5	–16.8	–23.5	–5.8	–7.8	–13.9	0.0	–28.4	0.0
OPBE0	1.9	–6.0	6.3	0.0	–2.7	–7.8	–5.2	0.0	–4.9	0.0
PBE0	–3.0	–4.6	0.1	–1.4	–1.2	–3.0	–4.7	0.0	–7.2	0.0
mPW1PW	–0.9	–0.9	1.9	1.7	–0.4	–1.7	–3.6	0.0	–4.6	0.0
mPW1K	1.1	–6.3	4.5	–4.3	1.5	–3.2	–0.4	0.0	0.0	0.0
S12H	–3.2	–4.9	1.1	0.5	–0.2	–3.2	–2.7	0.0	–6.0	0.0
X3LYP	2.3	8.9	2.7	8.5	1.8	3.6	–1.7	0.0	–1.1	0.0
					Meta-Hybrids					
M06	–0.3	0.3	4.0	6.6	0.3	–1.1	–1.9	0.0	–1.8	0.0
M06-2X	–2.0	–1.4	2.7	–0.9	1.2	–0.4	–0.6	0.0	–1.5	0.0
M06-HF	–5.1	–1.3	1.1	–12.1	–0.5	0.8	0.5	0.0	0.6	0.0
TPSSH	–2.6	5.1	–0.6	6.7	–1.8	–0.9	–4.3	0.0	–5.0	0.0
					Double-Hybrids					
B2K-PLYP	–1.5	–1.2	0.2	3.8	1.4	0.2	–1.2	0.0	–1.2	0.0
B2T-PLYP	–0.5	2.2	0.7	6.3	1.3	1.2	–1.5	0.0	–1.2	0.0
B2-PLYP	–0.5	4.2	0.5	8.2	1.0	1.9	–2.1	0.0	–2.0	0.0
LS1-TPSS	–5.4	–8.4	–2.1	–0.3	–0.4	–3.5	–3.2	0.0	–4.9	0.0
mPW2K-PLYP	–1.8	–1.3	–0.2	3.6	1.5	0.4	–1.1	0.0	–1.4	0.0
mPW2-PLYP	–0.2	3.2	0.8	6.3	1.8	2.0	–1.0	0.0	–0.8	0.0
PBE0-DH	–2.7	–8.8	0.8	–4.4	–0.1	–4.1	–2.7	0.0	–4.4	0.0
revDSD-BLYP	–2.6	–1.7	–0.7	3.4	1.6	0.6	–0.3	0.0	–1.1	0.0
revDSD-PBE	–3.3	–4.7	–0.8	1.8	0.4	–1.7	–1.1	0.0	–3.1	0.0
revDSD-PBEP86	–1.1	–2.0	0.9	3.9	1.2	–0.7	0.0	0.0	–0.9	0.0
					Range-Separated Hybrids					
CAM-B3LYP	3.8	2.5	4.6	1.3	2.8	1.2	0.4	0.0	1.4	0.0
CAMY-B3LYP	1.9	3.5	2.3	2.7	2.1	1.9	–0.8	0.0	–0.6	0.0
ωB97	3.4	–7.9	4.2	–5.8	3.3	–4.5	3.3	0.0	3.1	0.0
ωB97X	2.9	–4.3	3.8	–3.1	2.8	–2.5	1.9	0.0	1.6	0.0
ωB97X-D	3.4	0.1	4.6	1.2	1.6	–1.7	–0.4	0.0	0.1	0.0

Table S29 (continued).

XC	Diels-Alder Reaction		1,3-dipolar Cycloaddition		Electrocyclic Rearrangement		Sigmatropic Rearrangement		Double Group Transfer	
	$\Delta E^{‡}$	ΔE_{rxn}	$\Delta E^{‡}$	ΔE_{rxn}	$\Delta E^{‡}$	ΔE_{rxn}	$\Delta E^{‡}$	ΔE_{rxn}	$\Delta E^{‡}$	ΔE_{rxn}
					Dispersion-Corrected					
BP86-D3(BJ)	–10.6	1.2	–7.0	4.9	–3.7	0.6	–9.1	0.0	–15.5	0.0
BLYP-D3(BJ)	–6.3	11.7	–4.4	13.2	–0.8	6.0	–5.8	0.0	–9.4	0.0
PBE-D3(BJ)	–10.5	–1.1	–6.9	3.4	–4.3	–0.9	–9.5	0.0	–16.6	0.0
OLYP-D3(BJ)	–11.7	–1.7	–5.8	4.0	–3.9	–0.6	–9.1	0.0	–13.9	0.0
OPBE-D3(BJ)	–16.0	–13.5	–7.3	–3.7	–7.2	–7.4	–12.5	0.0	–20.2	0.0
B3LYP-D3(BJ)	–3.4	5.3	–1.0	6.1	1.3	3.9	–2.4	0.0	–4.3	0.0
PBE0-D3(BJ)	–6.5	–7.3	–2.3	–3.7	–1.3	–2.9	–4.9	0.0	–9.2	0.0
M06-2X-D3	–2.1	–1.5	2.6	–1.0	1.2	–0.4	–0.6	0.0	–1.6	0.0

Table S30. Mean error (ME), mean absolute error (MAE), maximum unsigned error (MUE) and standard deviation (SD) of reaction barriers and energies (kcal mol^{-1}) for the various pericyclic reactions, computed with XC/TZ2P//BP86/TZ2P compared to CCSDT(Q)/CBS//CCSD(T)/cc-pVTZ.

XC	$\Delta E^{\ddagger}$				ΔE_{rxn}				Total			
	ME	MAE	MUE	SD	ME	MAE	MUE	SD	ME	MAE	MUE	SD
						LDA						
VWN	–16.7	16.7	28.1	6.9	–11.6	11.6	16.9	4.7	–14.8	14.8	28.1	6.6
						GGAs						
BP86	–6.3	6.3	12.1	3.5	5.1	5.1	8.8	3.5	–2.0	5.8	12.1	3.6
BLYP	–1.5	2.6	5.0	1.9	14.1	14.1	18.3	5.8	4.3	6.9	18.3	6.8
BEE	–5.9	5.9	11.7	4.1	4.6	5.5	9.8	3.4	–2.0	5.8	11.7	3.9
PW91	–7.8	7.8	14.4	3.8	2.4	2.7	5.6	2.1	–4.0	5.9	14.4	4.1
PBE	–7.7	7.7	14.3	3.8	2.3	3.0	6.0	2.1	–3.9	5.9	14.3	4.0
PBEsol	–12.6	12.6	21.8	5.2	–5.7	5.7	8.5	2.0	–10.0	10.0	21.8	5.4
RPBE	–3.6	4.1	8.4	3.1	8.3	8.3	14.0	5.7	0.8	5.6	14.0	4.7
revPBE	–3.9	4.3	8.9	3.3	7.7	7.7	13.4	5.6	0.4	5.5	13.4	4.6
mPBE	–6.6	6.6	12.7	3.6	4.0	4.3	8.1	3.1	–2.6	5.7	12.7	3.6
mPW	–5.6	5.6	11.2	3.4	5.6	5.6	9.9	3.9	–1.4	5.6	11.2	3.6
HTBS	–7.8	7.8	14.7	4.4	1.3	3.4	5.8	1.9	–4.4	6.1	14.7	4.2
OLYP	–0.5	4.7	5.9	1.3	9.5	9.8	16.3	6.8	3.3	6.6	16.3	4.9
OPBE	–4.5	6.2	11.1	3.9	0.3	5.2	7.9	3.4	–2.7	5.8	11.1	3.8
XLYP	–1.1	2.3	4.3	1.7	15.0	15.0	19.3	5.9	4.9	7.1	19.3	7.3
						Meta–GGAs						
M06-L	–1.5	2.3	4.1	1.3	4.5	5.6	12.3	4.8	0.7	3.5	12.3	3.5
MVS	–6.0	6.0	13.5	5.1	–1.4	4.3	6.6	1.9	–4.3	5.4	13.5	4.3
TPSS	–4.4	4.4	7.3	1.9	6.0	6.0	9.9	4.3	–0.5	5.0	9.9	3.1
revTPSS	–4.4	4.4	5.4	0.9	3.7	4.8	6.8	2.3	–1.4	4.6	6.8	1.6
						Hybrids						
B3LYP	1.1	2.1	3.4	1.0	8.2	8.2	10.5	3.1	3.8	4.4	10.5	3.6
B3LYP*	–0.7	2.1	3.6	1.2	7.8	7.8	10.0	3.0	2.5	4.2	10.0	3.5
B1LYP	2.6	2.7	4.6	1.6	8.2	8.2	10.7	2.9	4.7	4.8	10.7	3.5
B1PW91	–1.1	2.3	4.2	1.7	0.0	1.7	2.5	0.9	–0.7	2.1	4.2	1.5
BHandH	–4.0	4.4	7.8	2.6	–14.1	14.1	19.0	6.8	–7.8	8.0	19.0	6.6
BHandHLYP	6.6	6.6	8.1	1.5	2.2	2.2	3.2	0.8	4.9	4.9	8.1	2.5
KMLYP	1.2	1.5	3.3	1.2	–9.6	9.6	13.1	4.7	–2.9	4.6	13.1	5.0
O3LYP	–17.2	17.2	28.4	7.5	–19.6	19.6	27.5	8.5	–18.1	18.1	28.4	8.0
OPBE0	–0.9	4.2	6.3	1.6	–4.6	4.6	7.8	3.3	–2.3	4.4	7.8	2.4
PBE0	–3.2	3.2	7.2	2.5	–3.0	3.0	4.6	1.3	–3.1	3.1	7.2	2.1
mPW1PW	–1.5	2.3	4.6	1.6	–0.3	1.4	1.7	0.4	–1.1	1.9	4.6	1.4
mPW1K	1.3	1.5	4.5	1.6	–4.6	4.6	6.3	1.3	–0.9	2.7	6.3	2.1
S12H	–2.2	2.6	6.0	2.0	–2.5	2.8	4.9	1.8	–2.3	2.7	6.0	1.9
X3LYP	0.8	1.9	2.7	0.5	7.0	7.0	8.9	2.4	3.1	3.8	8.9	2.9
						Meta–Hybrids						
M06	0.1	1.7	4.0	1.4	1.9	2.6	6.6	2.8	0.8	2.0	6.6	2.1
M06-2X	0.0	1.6	2.7	0.7	–0.9	0.9	1.4	0.4	–0.4	1.3	2.7	0.7
M06-HF	–0.7	1.6	5.1	1.8	–4.2	4.7	12.1	5.3	–2.0	2.7	12.1	3.8
TPSSH	–2.9	2.9	5.0	1.6	3.6	4.2	6.7	2.4	–0.4	3.4	6.7	2.1
						Double–Hybrids						
B2K-PLYP	–0.5	1.1	1.5	0.5	0.9	1.7	3.8	1.5	0.1	1.3	3.8	1.0
B2T-PLYP	–0.2	1.0	1.5	0.4	3.2	3.2	6.3	2.2	1.0	1.8	6.3	1.7
B2-PLYP	–0.6	1.2	2.1	0.7	4.7	4.7	8.2	2.6	1.4	2.5	8.2	2.4
LS1-TPSS	–3.2	3.2	5.4	1.8	–4.0	4.0	8.4	3.3	–3.5	3.5	8.4	2.5
mPW2K-PLYP	–0.6	1.2	1.8	0.5	0.9	1.7	3.6	1.4	0.0	1.4	3.6	1.0
mPW2-PLYP	0.1	0.9	1.8	0.5	3.8	3.8	6.3	1.8	1.5	2.0	6.3	1.8
PBE0-DH	–1.8	2.1	4.4	1.5	–5.7	5.7	8.8	2.1	–3.3	3.5	8.8	2.5
revDSD-BLYP	–0.6	1.2	2.6	0.8	0.8	1.9	3.4	1.2	–0.1	1.5	3.4	1.0
revDSD-PBE	–1.6	1.7	3.3	1.2	–1.5	2.7	4.7	1.4	–1.6	2.1	4.7	1.4
revDSD-PBEP86	0.0	0.8	1.2	0.4	0.4	2.2	3.9	1.3	0.2	1.3	3.9	1.1
						Range-Separated Hybrids						
CAM-B3LYP	2.6	2.6	4.6	1.5	1.6	1.6	2.5	0.6	2.2	2.2	4.6	1.4
CAMY-B3LYP	1.0	1.5	2.3	0.7	2.7	2.7	3.5	0.7	1.6	2.0	3.5	0.9
ωB97	3.5	3.5	4.2	0.4	-6.0	6.0	7.9	1.4	-0.1	4.4	7.9	1.5
ωB97X	2.6	2.6	3.8	0.8	–3.3	3.3	4.3	0.7	0.4	2.9	4.3	0.8
ωB97X-D	1.9	2.0	4.6	1.8	–0.1	1.0	1.7	0.6	1.1	1.6	4.6	1.5

Table S30 (continued).

XC	$\Delta E^{\ddagger}$				ΔE_{rxn}				Total			
	ME	MAE	MUE	SD	ME	MAE	MUE	SD	ME	MAE	MUE	SD
					Dispersion–Corrected							
BP86-D3(BJ)	–9.2	9.2	15.5	3.9	2.2	2.2	4.9	1.9	–4.9	6.6	15.5	4.7
BLYP-D3(BJ)	–5.3	5.3	9.4	2.8	10.3	10.3	13.2	3.1	0.5	7.2	13.2	3.8
PBE-D3(BJ)	–9.6	9.6	16.6	4.1	0.5	1.8	3.4	1.1	–5.8	6.6	16.6	5.0
OLYP-D3(BJ)	–8.9	8.9	13.9	3.7	0.6	2.1	4.0	1.4	–5.3	6.3	13.9	4.5
OPBE-D3(BJ)	–12.6	12.6	20.2	5.0	–8.2	8.2	13.5	4.0	–11.0	11.0	20.2	5.1
B3LYP-D3(BJ)	–2.0	2.5	4.3	1.3	5.1	5.1	6.1	0.9	0.7	3.4	6.1	1.7
PBE0-D3(BJ)	–4.8	4.8	9.2	2.9	–4.6	4.6	7.3	1.9	–4.8	4.8	9.2	2.5
M06-2X-D3	–0.1	1.6	2.6	0.7	–0.9	0.9	1.5	0.5	–0.4	1.4	2.6	0.7

Table S31. Reaction barriers and reaction energies (kcal mol^{-1}) for the various pericyclic reactions, computed with XC/QZ4P//BP86/TZ2P.

XC	Diels-Alder Reaction		1,3-dipolar Cycloaddition		Electrocyclic Rearrangement		Sigmatropic Rearrangement		Double Group Transfer	
	$\Delta E^{\ddagger}$	ΔE_{rxn}	$\Delta E^{\ddagger}$	ΔE_{rxn}	$\Delta E^{\ddagger}$	ΔE_{rxn}	$\Delta E^{\ddagger}$	ΔE_{rxn}	$\Delta E^{\ddagger}$	ΔE_{rxn}
LDA										
VWN	0.8	–65.0	3.2	–42.2	36.3	2.4	21.9	0.0	22.1	0.0
GGAs										
BP86	15.0	–42.3	15.4	–20.8	39.6	8.2	28.2	0.0	38.1	0.0
BLYP	21.0	–30.2	19.3	–11.1	42.7	13.8	31.9	0.0	45.2	0.0
BEE	16.1	–43.1	17.2	–20.0	38.9	6.3	28.0	0.0	38.4	0.0
PW91	12.4	–46.2	13.3	–24.1	39.3	7.4	27.8	0.0	35.8	0.0
PBE	12.8	–46.3	13.9	–23.7	38.9	6.7	27.6	0.0	35.8	0.0
PBEsol	6.7	–56.8	8.8	–33.7	36.6	3.2	24.0	0.0	28.4	0.0
RPBE	18.8	–38.1	19.2	–15.8	40.0	8.2	29.8	0.0	41.7	0.0
revPBE	18.5	–39.0	19.0	–16.4	39.7	7.8	29.4	0.0	41.2	0.0
mPBE	14.4	–44.1	15.3	–21.6	39.2	7.2	28.2	0.0	37.4	0.0
mPW	15.5	–41.9	16.1	–19.8	39.9	8.3	28.8	0.0	38.9	0.0
HTBS	13.9	–47.1	15.5	–24.1	37.4	4.7	26.1	0.0	35.5	0.0
OLYP	24.0	–36.0	23.9	–13.6	40.4	7.1	30.8	0.0	45.4	0.0
OPBE	19.4	–48.5	21.9	–22.5	36.7	0.1	26.9	0.0	39.0	0.0
XLYP	21.3	–28.9	19.3	–10.1	43.2	14.7	32.5	0.0	45.9	0.0
Meta-GGAs										
M06-L	16.9	–45.9	19.8	–17.2	43.6	5.9	34.1	0.0	46.0	0.0
MVS	12.8	–54.2	18.6	–24.6	42.7	5.9	28.2	0.0	36.7	0.0
TPSS	15.5	–40.3	15.9	–19.8	40.2	7.7	30.9	0.0	42.9	0.0
revTPSS	14.6	–42.3	15.0	–22.9	39.6	6.2	31.5	0.0	44.8	0.0
Hybrids										
B3LYP	22.6	–37.5	21.7	–18.9	44.8	11.8	35.1	0.0	49.6	0.0
B3LYP*	20.6	–38.3	19.9	–19.3	43.8	11.5	33.5	0.0	46.7	0.0
B1LYP	24.0	–37.3	23.0	–19.2	45.7	12.1	36.4	0.0	51.9	0.0
B1PW91	19.6	–48.5	21.2	–26.9	42.4	5.7	33.0	0.0	46.2	0.0
BHandH	12.9	–66.3	14.9	–48.1	44.1	3.5	34.1	0.0	42.6	0.0
BHandHLYP	26.9	–44.5	26.5	–27.9	48.6	10.3	41.2	0.0	58.5	0.0
KMLYP	19.3	–60.2	21.0	–42.2	46.4	5.1	37.9	0.0	50.3	0.0
O3LYP	–1.8	–75.5	1.4	–53.1	37.3	0.2	23.0	0.0	21.9	0.0
OPBE0	21.4	–54.3	24.4	–30.0	40.0	–0.3	31.6	0.0	45.3	0.0
PBE0	16.5	–52.6	18.4	–30.9	41.8	4.9	32.2	0.0	43.1	0.0
mPW1PW	18.7	–48.9	20.2	–27.7	42.6	6.2	33.2	0.0	45.7	0.0
mPW1K	20.9	–54.0	22.9	–33.6	44.6	4.7	36.5	0.0	50.4	0.0
S12H	16.4	–53.1	19.4	–29.2	42.7	4.5	34.1	0.0	44.2	0.0
X3LYP	21.9	–39.1	21.1	–20.7	44.9	11.6	35.2	0.0	49.2	0.0
Meta-Hybrids										
M06	19.4	–47.6	22.4	–22.7	43.4	7.0	34.9	0.0	48.4	0.0
M06-2X	17.8	–48.7	21.2	–29.7	44.4	7.8	36.4	0.0	49.0	0.0
M06-HF	13.9	–48.4	18.7	–40.6	42.5	9.7	37.3	0.0	50.7	0.0
TPSSH	16.9	–43.2	17.6	–22.9	41.2	6.9	32.5	0.0	45.2	0.0
Double-Hybrids										
B2K-PLYP	18.2	–48.5	17.7	–25.2	44.3	7.7	35.4	0.0	49.2	0.0
B2T-PLYP	19.2	–45.3	19.2	–22.8	44.3	9.0	35.1	0.0	49.3	0.0
B2-PLYP	19.1	–43.4	18.2	–20.9	43.9	9.6	34.5	0.0	48.5	0.0
LS1-TPSS	14.2	–55.7	15.1	–29.5	42.3	3.9	33.3	0.0	45.5	0.0
mPW2K-PLYP	17.9	–48.7	17.3	–25.5	44.4	7.9	35.4	0.0	49.0	0.0
mPW2-PLYP	19.5	–44.4	18.6	–22.8	44.8	9.8	35.6	0.0	49.6	0.0
PBE0-DH	17.1	–56.3	18.9	–33.7	42.8	3.8	34.0	0.0	46.1	0.0
revDSD-BLYP	17.5	–48.5	17.0	–25.2	44.6	8.4	36.3	0.0	46.9	0.0
revDSD-PBE	16.9	–51.4	17.0	–26.9	43.4	6.3	35.5	0.0	47.8	0.0
revDSD-PBEP86	19.0	–48.7	18.7	–24.7	44.2	7.1	36.6	0.0	50.0	0.0
Range-Separated Hybrids										
CAM-B3LYP	23.5	–45.3	22.1	–27.8	45.9	9.2	37.2	0.0	51.7	0.0
CAMY-B3LYP	21.6	–44.4	20.6	–26.5	45.3	9.9	36.1	0.0	49.8	0.0
ωB97	23.2	–56.1	22.7	–35.3	46.3	3.1	40.1	0.0	53.2	0.0
ωB97X	22.7	–52.2	22.3	–32.4	45.8	5.4	38.7	0.0	51.8	0.0
ωB97X-D	23.0	–47.6	23.0	–28.0	44.6	6.3	36.4	0.0	50.3	0.0

Table S31 (continued).

XC	Diels-Alder Reaction		1,3-dipolar Cycloaddition		Electrocyclic Rearrangement		Sigmatropic Rearrangement		Double Group Transfer	
	$\Delta E^{\ddagger}$	ΔE_{rxn}	$\Delta E^{\ddagger}$	ΔE_{rxn}	$\Delta E^{\ddagger}$	ΔE_{rxn}	$\Delta E^{\ddagger}$	ΔE_{rxn}	$\Delta E^{\ddagger}$	ΔE_{rxn}
Dispersion-Corrected										
BP86-D3(BJ)	8.8	–47.2	11.2	–24.7	39.4	8.4	27.7	0.0	34.7	0.0
BLYP-D3(BJ)	13.1	–36.6	13.8	–16.2	42.3	13.9	31.0	0.0	40.8	0.0
PBE-D3(BJ)	8.9	–49.4	11.2	–26.3	38.8	6.8	27.2	0.0	33.5	0.0
OLYP-D3(BJ)	7.7	–50.2	12.2	–25.9	39.0	6.9	27.5	0.0	36.1	0.0
OPBE-D3(BJ)	3.2	–62.2	10.5	–34.1	35.5	–0.1	24.1	0.0	29.9	0.0
B3LYP-D3(BJ)	16.1	–42.7	17.3	–23.1	44.5	11.9	34.4	0.0	46.0	0.0
PBE0-D3(BJ)	13.0	–55.3	16.0	–33.2	41.7	4.9	31.9	0.0	41.1	0.0
M06-2X-D3	17.7	–48.8	21.1	–29.8	44.4	7.8	36.4	0.0	48.9	0.0

Table S32. Errors in reaction barriers and reaction energies (kcal mol^{-1}) for the various pericyclic reactions, computed with XC/QZ4P//BP86/TZ2P, compared to CCSDT(Q)/CBS//CCSD(T)/cc-pVTZ.

XC	Diels-Alder Reaction		1,3-dipolar Cycloaddition		Electrocyclic Rearrangement		Sigmatropic Rearrangement		Double Group Transfer	
	$\Delta E^{\ddagger}$	ΔE_{rxn}	$\Delta E^{\ddagger}$	ΔE_{rxn}	$\Delta E^{\ddagger}$	ΔE_{rxn}	$\Delta E^{\ddagger}$	ΔE_{rxn}	$\Delta E^{\ddagger}$	ΔE_{rxn}
LDA										
VWN	–18.8	–17.4	–15.1	–13.3	–7.1	–6.1	–14.9	0.0	–28.1	0.0
GGAs										
BP86	–4.6	5.4	–2.9	8.1	–3.8	–0.3	–8.6	0.0	–12.1	0.0
BLYP	1.4	17.5	1.0	17.8	–0.7	5.4	–4.9	0.0	–5.0	0.0
BEE	–3.5	4.6	–1.1	8.9	–4.5	–2.2	–8.8	0.0	–11.8	0.0
PW91	–7.2	1.5	–5.0	4.8	–4.1	–1.1	–9.0	0.0	–14.4	0.0
PBE	–6.8	1.4	–4.4	5.2	–4.5	–1.8	–9.2	0.0	–14.4	0.0
PBEsol	–12.9	–9.2	–9.5	–4.8	–6.8	–5.3	–12.8	0.0	–21.8	0.0
RPBE	–0.8	9.6	0.9	13.1	–3.4	–0.3	–7.0	0.0	–8.5	0.0
revPBE	–1.1	8.7	0.7	12.5	–3.7	–0.6	–7.4	0.0	–9.0	0.0
mPBE	–5.2	3.6	–3.0	7.3	–4.2	–1.3	–8.6	0.0	–12.8	0.0
mPW	–4.1	5.8	–2.2	9.1	–3.5	–0.1	–8.0	0.0	–11.3	0.0
HTBS	–5.7	0.5	–2.8	4.8	–6.0	–3.8	–10.7	0.0	–14.7	0.0
OLYP	4.4	11.7	5.6	15.3	–3.0	–1.4	–6.0	0.0	–4.8	0.0
OPBE	–0.2	–0.9	3.6	6.4	–6.7	–8.4	–9.9	0.0	–11.2	0.0
XLYP	1.7	18.8	1.0	18.8	–0.2	6.3	–4.3	0.0	–4.3	0.0
Meta-GGAs										
M06-L	–2.7	1.8	1.5	11.7	0.2	–2.6	–2.7	0.0	–4.2	0.0
MVS	–6.8	–6.6	0.3	4.3	–0.7	–2.6	–8.6	0.0	–13.5	0.0
TPSS	–4.1	7.4	–2.4	9.1	–3.2	–0.7	–5.9	0.0	–7.3	0.0
revTPSS	–5.0	5.4	–3.3	6.0	–3.8	–2.3	–5.3	0.0	–5.4	0.0
Hybrids										
B3LYP	3.0	10.2	3.4	10.0	1.4	3.4	–1.7	0.0	–0.6	0.0
B3LYP*	1.0	9.4	1.6	9.6	0.4	3.1	–3.3	0.0	–3.5	0.0
B1LYP	4.4	10.4	4.7	9.7	2.3	3.7	–0.4	0.0	1.7	0.0
B1PW91	0.0	–0.9	2.9	2.0	–1.0	–2.8	–3.8	0.0	–4.0	0.0
BHandH	–6.7	–18.7	–3.4	–19.2	0.7	–5.0	–2.7	0.0	–7.6	0.0
BHandHLYP	7.3	3.2	8.2	1.0	5.2	1.9	4.4	0.0	8.3	0.0
KMLYP	–0.3	–12.6	2.7	–13.3	3.0	–3.4	1.1	0.0	0.1	0.0
O3LYP	–21.4	–27.9	–16.9	–24.2	–6.1	–8.3	–13.8	0.0	–28.3	0.0
OPBE0	1.8	–6.7	6.1	–1.1	–3.4	–8.8	–5.2	0.0	–4.9	0.0
PBE0	–3.1	–5.0	0.1	–2.0	–1.6	–3.6	–4.6	0.0	–7.1	0.0
mPW1PW	–0.9	–1.3	1.9	1.2	–0.8	–2.3	–3.6	0.0	–4.5	0.0
mPW1K	1.3	–6.4	4.6	–4.7	1.2	–3.8	–0.3	0.0	0.2	0.0
S12H	–3.2	–5.5	1.1	–0.3	–0.7	–4.0	–2.7	0.0	–6.0	0.0
X3LYP	2.3	8.6	2.8	8.2	1.5	3.2	–1.6	0.0	–1.0	0.0
Meta-Hybrids										
M06	–0.2	0.0	4.1	6.2	0.0	–1.5	–1.9	0.0	–1.8	0.0
M06-2X	–1.8	–1.1	2.9	–0.8	1.0	–0.6	–0.4	0.0	–1.2	0.0
M06-HF	–5.7	–0.8	0.4	–11.7	–0.9	1.3	0.5	0.0	0.5	0.0
TPSSH	–2.7	4.5	–0.7	6.0	–2.2	–1.6	–4.3	0.0	–5.0	0.0
Double-Hybrids										
B2K-PLYP	–1.4	–0.9	–0.6	3.7	0.9	–0.7	–1.4	0.0	–1.0	0.0
B2T-PLYP	–0.4	2.4	0.9	6.1	0.9	0.6	–1.7	0.0	–0.9	0.0
B2-PLYP	–0.5	4.3	–0.1	8.0	0.5	1.2	–2.3	0.0	–1.7	0.0
LS1-TPSS	–5.4	–8.1	–3.2	–0.6	–1.1	–4.6	–3.5	0.0	–4.7	0.0
mPW2K-PLYP	–1.7	–1.1	–1.0	3.4	1.0	–0.5	–1.4	0.0	–1.2	0.0
mPW2-PLYP	–0.1	3.3	0.3	6.1	1.4	1.4	–1.2	0.0	–0.6	0.0
PBE0-DH	–2.5	–8.7	0.6	–4.8	–0.6	–4.7	–2.8	0.0	–4.1	0.0
revDSD-BLYP	–2.1	–0.9	–1.3	3.7	1.2	0.0	–0.5	0.0	–3.3	0.0
revDSD-PBE	–2.7	–3.8	–1.3	2.0	0.0	–2.2	–1.3	0.0	–2.4	0.0
revDSD-PBEP86	–0.6	–1.1	0.4	4.2	0.8	–1.4	–0.2	0.0	–0.2	0.0
Range-Separated Hybrids										
CAM-B3LYP	3.9	2.4	3.8	1.1	2.5	0.8	0.4	0.0	1.5	0.0
CAMY-B3LYP	2.0	3.3	2.3	2.4	1.9	1.5	–0.7	0.0	–0.4	0.0
ωB97	3.6	–8.5	4.4	–6.4	2.9	–5.4	3.3	0.0	3.0	0.0
ωB97X	3.1	–4.6	4.0	–3.5	2.4	–3.1	1.9	0.0	1.6	0.0
ωB97X-D	3.4	0.0	4.7	0.9	1.2	–2.2	–0.4	0.0	0.1	0.0

Table S32 (continued).

XC	Diels-Alder Reaction		1,3-dipolar Cycloaddition		Electrocyclic Rearrangement		Sigmatropic Rearrangement		Double Group Transfer	
	$\Delta E^{\ddagger}$	ΔE_{rxn}	$\Delta E^{\ddagger}$	ΔE_{rxn}	$\Delta E^{\ddagger}$	ΔE_{rxn}	$\Delta E^{\ddagger}$	ΔE_{rxn}	$\Delta E^{\ddagger}$	ΔE_{rxn}
					Dispersion-Corrected					
BP86-D3(BJ)	–10.8	0.4	–7.1	4.2	–4.0	0.0	–9.1	0.0	–15.5	0.0
BLYP-D3(BJ)	–6.5	11.1	–4.5	12.7	–1.1	5.5	–5.8	0.0	–9.4	0.0
PBE-D3(BJ)	–10.7	–1.8	–7.1	2.6	–4.6	–1.7	–9.6	0.0	–16.7	0.0
OLYP-D3(BJ)	–11.9	–2.6	–6.1	3.0	–4.4	–1.6	–9.3	0.0	–14.1	0.0
OPBE-D3(BJ)	–16.4	–14.6	–7.8	–5.2	–7.9	–8.6	–12.7	0.0	–20.3	0.0
B3LYP-D3(BJ)	–3.5	5.0	–1.0	5.8	1.1	3.5	–2.4	0.0	–4.2	0.0
PBE0-D3(BJ)	–6.6	–7.7	–2.3	–4.3	–1.7	–3.6	–4.9	0.0	–9.1	0.0
M06-2X-D3	–1.9	–1.2	2.8	–0.9	1.0	–0.6	–0.4	0.0	–1.3	0.0

Table S33. Mean error (ME), mean absolute error (MAE), maximum unsigned error (MUE) and standard deviation (SD) of reaction barriers and energies (kcal mol^{-1}) for the various pericyclic reactions, computed with XC/QZ4P//BP86/TZ2P compared to CCSDT(Q)/CBS//CCSD(T)/cc-pVTZ.

XC	$\Delta E^{\ddagger}$				ΔE_{rxn}				Total			
	ME	MAE	MUE	SD	ME	MAE	MUE	SD	ME	MAE	MUE	SD
LDA												
VWN	–16.8	16.8	28.1	6.8	–12.2	12.2	17.4	4.7	–15.1	15.1	28.1	6.5
GGAs												
BP86	–6.4	6.4	12.1	3.5	4.4	4.6	8.1	3.2	–2.4	5.7	12.1	3.5
BLYP	–1.6	2.6	5.0	1.9	13.5	13.5	17.8	5.8	4.0	6.7	17.8	6.5
BEE	–5.9	5.9	11.8	3.9	3.8	5.2	8.9	2.8	–2.3	5.7	11.8	3.5
PW91	–7.9	7.9	14.4	3.7	1.7	2.4	4.8	1.7	–4.3	5.9	14.4	4.1
PBE	–7.9	7.9	14.4	3.7	1.6	2.8	5.2	1.7	–4.3	5.9	14.4	4.0
PBEsol	–12.8	12.8	21.8	5.1	–6.4	6.4	9.2	1.9	–10.4	10.4	21.8	5.2
RPBE	–3.8	4.1	8.5	3.2	7.5	7.6	13.1	5.4	0.4	5.4	13.1	4.5
revPBE	–4.1	4.4	9.0	3.3	6.8	7.3	12.5	4.9	0.0	5.5	12.5	4.2
mPBE	–6.8	6.8	12.8	3.6	3.2	4.0	7.3	2.5	–3.0	5.7	12.8	3.5
mPW	–5.8	5.8	11.3	3.4	4.9	5.0	9.1	3.7	–1.8	5.5	11.3	3.5
HTBS	–8.0	8.0	14.7	4.2	0.5	3.0	4.8	1.8	–4.8	6.1	14.7	4.3
OLYP	–0.8	4.8	6.0	1.0	8.5	9.4	15.3	5.9	2.7	6.5	15.3	4.3
OPBE	–4.9	6.3	11.2	4.1	–0.9	5.2	8.4	3.2	–3.4	5.9	11.2	3.8
XLYP	–1.2	2.3	4.3	1.7	14.6	14.6	18.8	5.9	4.7	6.9	18.8	7.1
Meta–GGAs												
M06-L	–1.6	2.3	4.2	1.4	3.6	5.3	11.7	4.5	0.4	3.4	11.7	3.3
MVS	–5.9	6.0	13.5	5.0	–1.6	4.5	6.6	1.6	–4.3	5.4	13.5	4.1
TPSS	–4.6	4.6	7.3	1.8	5.2	5.7	9.1	3.6	–0.9	5.0	9.1	2.7
revTPSS	–4.6	4.6	5.4	0.8	3.0	4.5	6.0	1.6	–1.7	4.5	6.0	1.2
Hybrids												
B3LYP	1.1	2.0	3.4	1.0	7.8	7.8	10.2	3.2	3.6	4.2	10.2	3.5
B3LYP*	–0.8	2.0	3.5	1.2	7.3	7.3	9.6	3.0	2.3	4.0	9.6	3.3
B1LYP	2.5	2.7	4.7	1.7	7.9	7.9	10.4	3.0	4.5	4.6	10.4	3.4
B1PW91	–1.2	2.4	4.0	1.6	–0.5	1.9	2.8	0.8	–0.9	2.2	4.0	1.3
BHandH	–3.9	4.2	7.6	2.6	–14.3	14.3	19.2	6.6	–7.8	8.0	19.2	6.7
BHandHLYP	6.7	6.7	8.3	1.6	2.0	2.0	3.2	0.9	4.9	4.9	8.3	2.7
KMLYP	1.3	1.4	3.0	1.2	–9.7	9.7	13.3	4.5	–2.8	4.5	13.3	5.0
O3LYP	–17.3	17.3	28.3	7.4	–20.1	20.1	27.9	8.5	–18.4	18.4	28.3	8.0
OPBE0	–1.1	4.3	6.1	1.5	–5.5	5.5	8.8	3.2	–2.8	4.8	8.8	2.4
PBE0	–3.3	3.3	7.1	2.4	–3.5	3.5	5.0	1.2	–3.4	3.4	7.1	2.1
mPW1PW	–1.6	2.3	4.5	1.5	–0.8	1.6	2.3	0.5	–1.3	2.0	4.5	1.3
mPW1K	1.4	1.5	4.6	1.6	–4.9	4.9	6.4	1.1	–1.0	2.8	6.4	2.2
S12H	–2.3	2.7	6.0	1.9	–3.2	3.2	5.5	2.1	–2.7	2.9	6.0	2.0
X3LYP	0.8	1.8	2.8	0.6	6.6	6.6	8.6	2.5	3.0	3.6	8.6	2.8
Meta–Hybrids												
M06	0.0	1.6	4.1	1.5	1.6	2.6	6.2	2.6	0.6	2.0	6.2	2.0
M06-2X	0.1	1.5	2.9	0.9	–0.8	0.8	1.1	0.2	–0.3	1.2	2.9	0.7
M06-HF	–1.0	1.6	5.7	2.0	–3.7	4.6	11.7	5.1	–2.1	2.7	11.7	3.8
TPSSH	–3.0	3.0	5.0	1.5	3.0	4.0	6.0	1.8	–0.8	3.4	6.0	1.7
Double–Hybrids												
B2K-PLYP	–0.7	1.0	1.4	0.3	0.7	1.8	3.7	1.3	–0.2	1.3	3.7	0.9
B2T-PLYP	–0.2	0.9	1.7	0.4	3.0	3.0	6.1	2.3	1.0	1.7	6.1	1.7
B2-PLYP	–0.8	1.0	2.3	0.8	4.5	4.5	8.0	2.8	1.2	2.3	8.0	2.5
LS1-TPSS	–3.6	3.6	5.4	1.5	–4.4	4.4	8.1	3.0	–3.9	3.9	8.1	2.2
mPW2K-PLYP	–0.9	1.2	1.7	0.3	0.6	1.7	3.4	1.2	–0.3	1.4	3.4	0.8
mPW2-PLYP	0.0	0.7	1.4	0.5	3.6	3.6	6.1	1.9	1.3	1.8	6.1	1.9
PBE0-DH	–1.9	2.1	4.1	1.3	–6.0	6.0	8.7	1.8	–3.4	3.6	8.7	2.5
revDSD-BLYP	–1.2	1.7	3.3	1.0	0.9	1.5	3.7	1.5	–0.4	1.6	3.7	1.2
revDSD-PBE	–1.5	1.5	2.7	0.9	–1.3	2.6	3.8	0.8	–1.5	1.9	3.8	1.0
revDSD-PBEP86	0.0	0.4	0.8	0.2	0.6	2.2	4.2	1.4	0.2	1.1	4.2	1.2
Range-Separated Hybrids												
CAM-B3LYP	2.4	2.4	3.9	1.4	1.4	1.4	2.4	0.7	2.0	2.0	3.9	1.3
CAMY-B3LYP	1.0	1.5	2.3	0.8	2.4	2.4	3.3	0.7	1.5	1.8	3.3	0.9
ωB97	3.4	3.4	4.4	0.6	–6.7	6.7	8.5	1.3	–0.4	4.7	8.5	1.8
ωB97X	2.6	2.6	4.0	0.9	–3.7	3.7	4.6	0.6	0.2	3.0	4.6	1.0
ωB97X-D	1.8	2.0	4.7	1.8	–0.4	1.0	2.2	0.9	1.0	1.6	4.7	1.6

Table S33 (continued).

XC	$\Delta E^{\ddagger}$				ΔE_{rxn}				Total			
	ME	MAE	MUE	SD	ME	MAE	MUE	SD	ME	MAE	MUE	SD
					Dispersion–Corrected							
BP86-D3(BJ)	–9.3	9.3	15.5	3.8	1.5	1.6	4.2	1.9	–5.2	6.4	15.5	5.0
BLYP-D3(BJ)	–5.5	5.5	9.4	2.7	9.7	9.7	12.7	3.1	0.2	7.1	12.7	3.5
PBE-D3(BJ)	–9.7	9.7	16.7	4.1	–0.3	2.0	2.6	0.4	–6.2	6.8	16.7	5.0
OLYP-D3(BJ)	–9.2	9.2	14.1	3.6	–0.4	2.4	3.0	0.6	–5.9	6.6	14.1	4.4
OPBE-D3(BJ)	–13.0	13.0	20.3	4.9	–9.4	9.4	14.6	3.9	–11.7	11.7	20.3	4.8
B3LYP-D3(BJ)	–2.0	2.4	4.2	1.3	4.7	4.7	5.8	1.0	0.5	3.3	5.8	1.6
PBE0-D3(BJ)	–4.9	4.9	9.1	2.7	–5.2	5.2	7.7	1.8	–5.0	5.0	9.1	2.4
M06-2X-D3	0.0	1.5	2.8	0.8	–0.9	0.9	1.2	0.2	–0.3	1.3	2.8	0.7

Table S34. Reaction barriers and reaction energies (kcal mol^{-1}) for the various pericyclic reactions, computed with XC/TZ2P//BP86/QZ4P.

XC	Diels-Alder Reaction		1,3-dipolar Cycloaddition		Electrocyclic Rearrangement		Sigmatropic Rearrangement		Double Group Transfer	
	$\Delta E^{\ddagger}$	ΔE_{rxn}	$\Delta E^{\ddagger}$	ΔE_{rxn}	$\Delta E^{\ddagger}$	ΔE_{rxn}	$\Delta E^{\ddagger}$	ΔE_{rxn}	$\Delta E^{\ddagger}$	ΔE_{rxn}
LDA										
VWN	1.0	–64.5	3.3	–41.5	36.6	2.9	21.9	0.0	22.1	0.0
GGAs										
BP86	15.2	–41.6	15.5	–20.1	39.9	8.8	28.2	0.0	38.1	0.0
BLYP	21.1	–29.6	19.4	–10.6	43.0	14.3	31.9	0.0	45.2	0.0
BEE	16.4	–42.3	17.4	–19.1	39.3	7.1	28.1	0.0	38.5	0.0
PW91	12.6	–45.5	13.5	–23.3	39.6	8.0	27.8	0.0	35.8	0.0
PBE	13.1	–45.6	14.1	–22.9	39.3	7.4	27.6	0.0	35.8	0.0
PBEsol	7.0	–56.2	9.1	–32.8	37.1	3.8	24.0	0.0	28.4	0.0
RPBE	19.0	–37.3	19.4	–14.9	40.4	9.0	29.9	0.0	41.8	0.0
revPBE	18.7	–38.1	19.2	–15.5	40.1	8.5	29.5	0.0	41.3	0.0
mPBE	14.6	–43.3	15.5	–20.8	39.6	7.9	28.3	0.0	37.5	0.0
mPW	15.7	–41.2	16.3	–19.0	40.2	8.9	28.8	0.0	39.0	0.0
HTBS	14.2	–46.4	15.8	–23.1	37.9	5.4	26.1	0.0	35.5	0.0
OLYP	24.3	–35.1	24.2	–12.6	41.0	8.0	30.9	0.0	45.6	0.0
OPBE	19.8	–47.8	22.4	–21.0	37.4	1.2	27.1	0.0	39.1	0.0
XLYP	21.4	–28.4	19.4	–9.7	43.4	15.1	32.5	0.0	45.9	0.0
Meta-GGAs										
M06-L	16.7	–44.9	19.6	–16.6	43.8	6.9	34.2	0.0	46.0	0.0
MVS	12.2	–54.3	18.1	–24.5	43.1	6.5	28.2	0.0	36.7	0.0
TPSS	15.7	–39.5	16.1	–19.0	40.6	8.5	31.0	0.0	42.9	0.0
revTPSS	14.7	–41.7	15.1	–22.1	40.0	6.9	31.5	0.0	44.8	0.0
Hybrids										
B3LYP	22.6	–37.2	21.7	–18.5	45.0	12.2	35.0	0.0	49.5	0.0
B3LYP*	20.7	–37.8	19.9	–18.8	44.0	11.9	33.5	0.0	46.6	0.0
B1LYP	23.9	–37.0	23.0	–18.9	45.8	12.5	36.4	0.0	51.8	0.0
B1PW91	19.6	–48.1	21.2	–26.3	42.7	6.3	33.0	0.0	46.0	0.0
BHandH	12.8	–66.4	14.8	–47.8	44.4	3.9	34.1	0.0	42.4	0.0
BHandHLYP	26.6	–44.5	26.4	–27.6	48.8	10.6	41.1	0.0	58.3	0.0
KMLYP	19.0	–60.4	20.8	–41.9	46.7	5.4	37.8	0.0	50.1	0.0
O3LYP	–1.6	–75.2	1.5	–52.4	37.6	0.7	22.9	0.0	21.8	0.0
OPBE0	21.5	–53.6	24.6	–28.9	40.6	0.7	31.7	0.0	45.3	0.0
PBE0	16.6	–52.2	18.4	–30.3	42.1	5.5	32.1	0.0	43.0	0.0
mPW1PW	18.7	–48.6	20.2	–27.1	42.9	6.8	33.2	0.0	45.6	0.0
mPW1K	20.7	–53.9	22.8	–33.1	44.9	5.2	36.4	0.0	50.2	0.0
S12H	16.4	–52.5	19.4	–28.4	43.1	5.3	34.1	0.0	44.2	0.0
X3LYP	21.8	–38.8	21.0	–20.3	45.1	12.0	35.1	0.0	49.1	0.0
Meta-Hybrids										
M06	19.3	–47.4	22.3	–22.3	43.6	7.4	34.9	0.0	48.4	0.0
M06-2X	17.6	–49.0	21.1	–29.7	44.6	8.1	36.2	0.0	48.7	0.0
M06-HF	14.5	–48.9	19.5	–40.9	42.9	9.3	37.4	0.0	50.8	0.0
TPSSH	17.0	–42.6	17.7	–22.2	41.6	7.6	32.5	0.0	45.2	0.0
Double-Hybrids										
B2K-PLYP	18.1	–48.8	18.5	–25.0	44.8	8.8	35.6	0.0	49.0	0.0
B2T-PLYP	19.1	–45.5	19.0	–22.6	44.7	9.8	35.3	0.0	49.0	0.0
B2-PLYP	19.1	–43.5	18.8	–20.6	44.4	10.4	34.7	0.0	48.2	0.0
LS1-TPSS	14.2	–55.9	16.2	–29.1	43.0	5.3	33.7	0.0	45.3	0.0
mPW2K-PLYP	17.8	–48.9	18.1	–25.3	44.9	9.0	35.7	0.0	48.8	0.0
mPW2-PLYP	19.4	–44.5	19.1	–22.6	45.2	10.5	35.8	0.0	49.4	0.0
PBE0-DH	16.9	–56.4	19.1	–33.2	43.3	4.5	34.1	0.0	45.8	0.0
revDSD-BLYP	17.0	–49.3	17.6	–25.4	45.0	9.2	36.6	0.0	49.1	0.0
revDSD-PBE	16.3	–52.3	17.5	–27.1	43.8	7.0	35.7	0.0	47.1	0.0
revDSD-PBEP86	18.4	–49.6	19.2	–24.9	44.6	7.9	36.8	0.0	49.3	0.0
Range-Separated Hybrids										
CAM-B3LYP	23.4	–45.2	22.0	–27.6	46.2	9.6	37.2	0.0	51.6	0.0
CAMY-B3LYP	21.5	–44.2	20.6	–26.1	45.5	10.3	36.0	0.0	49.6	0.0
ωB97	23.0	–55.6	22.5	–34.7	46.7	4.0	40.2	0.0	53.3	0.0
ωB97X	22.5	–51.9	22.1	–31.9	46.2	6.0	38.7	0.0	51.8	0.0
ωB97X-D	22.9	–47.5	22.9	–27.9	45.0	6.8	36.4	0.0	50.3	0.0

Table S34 (continued).

XC	Diels-Alder Reaction		1,3-dipolar Cycloaddition		Electrocyclic Rearrangement		Sigmatropic Rearrangement		Double Group Transfer	
	$\Delta E^{\ddagger}$	ΔE_{rxn}	$\Delta E^{\ddagger}$	ΔE_{rxn}	$\Delta E^{\ddagger}$	ΔE_{rxn}	$\Delta E^{\ddagger}$	ΔE_{rxn}	$\Delta E^{\ddagger}$	ΔE_{rxn}
Dispersion-Corrected										
BP86-D3(BJ)	9.0	–46.5	11.3	–24.0	39.7	9.0	27.7	0.0	34.7	0.0
BLYP-D3(BJ)	13.2	–35.9	14.0	–15.7	42.6	14.4	31.0	0.0	40.9	0.0
PBE-D3(BJ)	9.0	–46.5	11.3	–24.0	39.7	9.0	44.5	0.0	34.7	0.0
OLYP-D3(BJ)	7.9	–49.3	12.6	–24.8	39.5	7.8	27.7	0.0	36.3	0.0
OPBE-D3(BJ)	3.6	–61.1	11.0	–32.6	36.2	1.1	24.3	0.0	30.0	0.0
B3LYP-D3(BJ)	16.1	–42.4	17.3	–22.7	44.7	12.3	34.4	0.0	45.9	0.0
PBE0-D3(BJ)	13.1	–55.0	16.0	–32.5	42.0	5.5	31.9	0.0	41.0	0.0
M06-2X-D3	17.5	–49.1	21.0	–29.8	44.6	8.1	36.3	0.0	48.6	0.0

Table S35. Errors in reaction barriers and reaction energies (kcal mol^{-1}) for the various pericyclic reactions, computed with XC/TZ2P//BP86/QZ4P, compared to CCSDT(Q)/CBS//CCSD(T)/cc-pVTZ.

XC	Diels-Alder Reaction		1,3-dipolar Cycloaddition		Electrocyclic Rearrangement		Sigmatropic Rearrangement		Double Group Transfer	
	$\Delta E^{\ddagger}$	ΔE_{rxn}	$\Delta E^{\ddagger}$	ΔE_{rxn}	$\Delta E^{\ddagger}$	ΔE_{rxn}	$\Delta E^{\ddagger}$	ΔE_{rxn}	$\Delta E^{\ddagger}$	ΔE_{rxn}
LDA										
VWN	–18.6	–16.9	–15.0	–12.6	–6.8	–5.6	–14.9	0.0	–28.1	0.0
GGAs										
BP86	–4.4	6.1	–2.8	8.8	–3.5	0.4	–8.6	0.0	–12.1	0.0
BLYP	1.5	18.1	1.1	18.3	–0.4	5.9	–4.9	0.0	–5.0	0.0
BEE	–3.2	5.4	–0.9	9.8	–4.1	–1.4	–8.7	0.0	–11.7	0.0
PW91	–7.0	2.2	–4.8	5.6	–3.8	–0.4	–9.0	0.0	–14.4	0.0
PBE	–6.5	2.1	–4.2	6.0	–4.1	–1.1	–9.2	0.0	–14.4	0.0
PBEsol	–12.6	–8.6	–9.2	–3.9	–6.3	–4.7	–12.8	0.0	–21.8	0.0
RPBE	–0.6	10.4	1.1	14.0	–3.0	0.6	–6.9	0.0	–8.4	0.0
revPBE	–0.9	9.6	0.9	13.4	–3.3	0.1	–7.3	0.0	–8.9	0.0
mPBE	–5.0	4.4	–2.8	8.1	–3.8	–0.5	–8.5	0.0	–12.7	0.0
mPW	–3.9	6.5	–2.0	9.9	–3.2	0.5	–8.0	0.0	–11.2	0.0
HTBS	–5.4	1.3	–2.5	5.8	–5.5	–3.1	–10.7	0.0	–14.7	0.0
OLYP	4.7	12.6	5.9	16.3	–2.4	–0.4	–5.9	0.0	–4.6	0.0
OPBE	0.2	–0.1	4.1	7.9	–6.0	–7.3	–9.7	0.0	–11.1	0.0
XLYP	1.8	19.3	1.1	19.2	0.0	6.7	–4.3	0.0	–4.3	0.0
Meta-GGAs										
M06-L	–2.9	2.8	1.3	12.3	0.4	–1.6	–2.6	0.0	–4.2	0.0
MVS	–7.4	–6.7	–0.2	4.4	–0.3	–2.0	–8.6	0.0	–13.5	0.0
TPSS	–3.9	8.2	–2.2	9.9	–2.8	0.1	–5.8	0.0	–7.3	0.0
revTPSS	–4.9	6.0	–3.2	6.8	–3.4	–1.6	–5.3	0.0	–5.4	0.0
Hybrids										
B3LYP	3.0	10.5	3.4	10.4	1.6	3.8	–1.8	0.0	–0.7	0.0
B3LYP*	1.1	9.9	1.6	10.1	0.6	3.5	–3.3	0.0	–3.6	0.0
B1LYP	4.3	10.7	4.7	10.0	2.4	4.1	–0.4	0.0	1.6	0.0
B1PW91	0.0	–0.5	2.9	2.6	–0.7	–2.2	–3.8	0.0	–4.2	0.0
BHandH	–6.8	–18.8	–3.5	–18.9	1.0	–4.6	–2.7	0.0	–7.8	0.0
BHandHLYP	7.0	3.2	8.1	1.3	5.4	2.2	4.3	0.0	8.1	0.0
KMLYP	–0.6	–12.8	2.5	–13.0	3.3	–3.1	1.0	0.0	–0.1	0.0
O3LYP	–21.2	–27.6	–16.8	–23.5	–5.8	–7.8	–13.9	0.0	–28.4	0.0
OPBE0	1.9	–6.0	6.3	0.0	–2.8	–7.8	–5.1	0.0	–4.9	0.0
PBE0	–3.0	–4.6	0.1	–1.4	–1.3	–3.0	–4.7	0.0	–7.2	0.0
mPW1PW	–0.9	–1.0	1.9	1.8	–0.5	–1.7	–3.6	0.0	–4.6	0.0
mPW1K	1.1	–6.3	4.5	–4.2	1.5	–3.3	–0.4	0.0	0.0	0.0
S12H	–3.2	–4.9	1.1	0.5	–0.3	–3.2	–2.7	0.0	–6.0	0.0
X3LYP	2.2	8.9	2.7	8.6	1.7	3.6	–1.7	0.0	–1.1	0.0
Meta-Hybrids										
M06	–0.3	0.3	4.0	6.6	0.2	–1.1	–1.9	0.0	–1.8	0.0
M06-2X	–2.0	–1.4	2.8	–0.8	1.2	–0.4	–0.6	0.0	–1.5	0.0
M06-HF	–5.1	–1.3	1.2	–12.0	–0.5	0.9	0.6	0.0	0.6	0.0
TPSSH	–2.6	5.1	–0.6	6.7	–1.8	–0.9	–4.3	0.0	–5.0	0.0
Double-Hybrids										
B2K-PLYP	–1.5	–1.2	0.2	3.9	1.4	0.4	–1.2	0.0	–1.2	0.0
B2T-PLYP	–0.5	2.2	0.7	6.3	1.3	1.4	–1.5	0.0	–1.2	0.0
B2-PLYP	–0.5	4.2	0.5	8.3	1.0	2.0	–2.1	0.0	–2.0	0.0
LS1-TPSS	–5.4	–8.3	–2.1	–0.2	–0.4	–3.2	–3.1	0.0	–4.9	0.0
mPW2K-PLYP	–1.8	–1.3	–0.2	3.6	1.5	0.6	–1.1	0.0	–1.4	0.0
mPW2-PLYP	–0.2	3.2	0.8	6.3	1.8	2.1	–1.0	0.0	–0.8	0.0
PBE0-DH	–2.7	–8.8	0.8	–4.3	–0.1	–4.0	–2.7	0.0	–4.4	0.0
revDSD-BLYP	–2.6	–1.7	–0.7	3.5	1.6	0.8	–0.2	0.0	–1.1	0.0
revDSD-PBE	–3.3	–4.7	–0.8	1.8	0.4	–1.5	–1.1	0.0	–3.1	0.0
revDSD-PBEP86	–1.2	–2.0	0.9	4.0	1.2	–0.5	0.0	0.0	–0.9	0.0
Range-Separated Hybrids										
CAM-B3LYP	3.8	2.5	3.7	1.3	2.8	1.2	0.4	0.0	1.4	0.0
CAMY-B3LYP	1.9	3.5	2.3	2.8	2.1	1.9	–0.8	0.0	–0.6	0.0
ωB97	3.4	–8.0	4.2	–5.8	3.3	–4.5	3.4	0.0	3.1	0.0
ωB97X	2.9	–4.3	3.8	–3.0	2.8	–2.5	1.9	0.0	1.6	0.0
ωB97X-D	3.3	0.1	4.6	1.0	1.6	–1.7	–0.4	0.0	0.1	0.0

Table S35 (continued).

XC	Diels-Alder Reaction		1,3-dipolar Cycloaddition		Electrocyclic Rearrangement		Sigmatropic Rearrangement		Double Group Transfer	
	$\Delta E^{\ddagger}$	ΔE_{rxn}	$\Delta E^{\ddagger}$	ΔE_{rxn}	$\Delta E^{\ddagger}$	ΔE_{rxn}	$\Delta E^{\ddagger}$	ΔE_{rxn}	$\Delta E^{\ddagger}$	ΔE_{rxn}
					Dispersion-Corrected					
BP86-D3(BJ)	–10.6	1.2	–7.0	4.9	–3.7	0.6	–9.1	0.0	–15.5	0.0
BLYP-D3(BJ)	–6.4	11.8	–4.3	13.2	–0.8	6.0	–5.8	0.0	–9.3	0.0
PBE-D3(BJ)	–10.6	1.2	–7.0	4.9	–3.7	0.6	7.7	0.0	–15.5	0.0
OLYP-D3(BJ)	–11.7	–1.7	–5.7	4.1	–3.9	–0.6	–9.1	0.0	–13.9	0.0
OPBE-D3(BJ)	–16.0	–13.5	–7.3	–3.7	–7.2	–7.4	–12.5	0.0	–20.2	0.0
B3LYP-D3(BJ)	–3.5	5.3	–1.0	6.2	1.3	3.9	–2.4	0.0	–4.3	0.0
PBE0-D3(BJ)	–6.5	–7.4	–2.3	–3.6	–1.4	–3.0	–4.9	0.0	–9.2	0.0
M06-2X-D3	–2.1	–1.5	2.7	–0.9	1.2	–0.4	–0.5	0.0	–1.6	0.0

Table S36. Mean error (ME), mean absolute error (MAE), maximum unsigned error (MUE) and standard deviation (SD) of reaction barriers and energies (kcal mol^{-1}) for the various pericyclic reactions, computed with XC/TZ2P//BP86/QZ4P compared to CCSDT(Q)/CBS//CCSD(T)/cc-pVTZ.

XC	$\Delta E^{\ddagger}$				ΔE_{rxn}				Total			
	ME	MAE	MUE	SD	ME	MAE	MUE	SD	ME	MAE	MUE	SD
					LDA							
VWN	–16.7	16.7	28.1	6.9	–11.7	11.7	16.9	4.7	–14.8	14.8	28.1	6.6
					GGAs							
BP86	–6.3	6.3	12.1	3.5	5.1	5.1	8.8	3.5	–2.0	5.8	12.1	3.6
BLYP	–1.5	2.6	5.0	2.0	14.1	14.1	18.3	5.8	4.3	6.9	18.3	6.8
BEE	–5.7	5.7	11.7	3.9	4.6	5.5	9.8	3.4	–1.9	5.6	11.7	3.8
PW91	–7.8	7.8	14.4	3.8	2.4	2.7	5.6	2.1	–4.0	5.9	14.4	4.1
PBE	–7.7	7.7	14.4	3.8	2.3	3.0	6.0	2.1	–3.9	5.9	14.4	4.0
PBEsol	–12.5	12.5	21.8	5.2	–5.7	5.7	8.6	2.0	–10.0	10.0	21.8	5.4
RPBE	–3.6	4.0	8.4	3.1	8.3	8.3	14.0	5.7	0.9	5.6	14.0	4.7
revPBE	–3.9	4.3	8.9	3.3	7.7	7.7	13.4	5.6	0.4	5.5	13.4	4.6
mPBE	–6.6	6.6	12.7	3.6	4.0	4.3	8.1	3.1	–2.6	5.7	12.7	3.6
mPW	–5.7	5.7	11.2	3.4	5.6	5.6	9.9	3.9	–1.4	5.6	11.2	3.6
HTBS	–7.8	7.8	14.7	4.4	1.3	3.4	5.8	1.9	–4.4	6.1	14.7	4.2
OLYP	–0.5	4.7	5.9	1.3	9.5	9.8	16.3	6.8	3.3	6.6	16.3	4.9
OPBE	–4.5	6.2	11.1	3.9	0.2	5.1	7.9	3.5	–2.8	5.8	11.1	3.8
XLYP	–1.1	2.3	4.3	1.7	15.0	15.0	19.3	5.9	4.9	7.1	19.3	7.3
					Meta–GGAs							
M06-L	–1.6	2.3	4.2	1.3	4.5	5.5	12.3	4.8	0.7	3.5	12.3	3.5
MVS	–6.0	6.0	13.5	5.1	–1.4	4.3	6.7	1.9	–4.3	5.4	13.5	4.3
TPSS	–4.4	4.4	7.3	1.9	6.0	6.0	9.9	4.3	–0.5	5.0	9.9	3.1
revTPSS	–4.4	4.4	5.4	0.9	3.7	4.8	6.8	2.3	–1.4	4.6	6.8	1.6
					Hybrids							
B3LYP	1.1	2.1	3.4	1.0	8.2	8.2	10.5	3.1	3.8	4.4	10.5	3.6
B3LYP*	–0.7	2.0	3.6	1.2	7.8	7.8	10.1	3.1	2.5	4.2	10.1	3.5
B1LYP	2.5	2.7	4.7	1.6	8.2	8.2	10.7	3.0	4.7	4.8	10.7	3.5
B1PW91	–1.2	2.3	4.2	1.7	0.0	1.7	2.6	0.9	–0.7	2.1	4.2	1.5
BHandH	–4.0	4.3	7.8	2.6	–14.1	14.1	18.9	6.7	–7.8	8.0	18.9	6.6
BHandHLYP	6.6	6.6	8.1	1.5	2.2	2.2	3.2	0.8	4.9	4.9	8.1	2.5
KMLYP	1.2	1.5	3.3	1.2	–9.6	9.6	13.0	4.6	–2.8	4.5	13.0	4.9
O3LYP	–17.2	17.2	28.4	7.5	–19.6	19.6	27.6	8.5	–18.1	18.1	28.4	8.0
OPBE0	–0.9	4.2	6.3	1.6	–4.6	4.6	7.8	3.3	–2.3	4.4	7.8	2.4
PBE0	–3.2	3.3	7.2	2.5	–3.0	3.0	4.6	1.3	–3.1	3.2	7.2	2.1
mPW1PW	–1.5	2.3	4.6	1.6	–0.3	1.5	1.8	0.4	–1.1	2.0	4.6	1.3
mPW1K	1.3	1.5	4.5	1.6	–4.6	4.6	6.3	1.2	–0.9	2.7	6.3	2.1
S12H	–2.2	2.7	6.0	2.0	–2.5	2.8	4.9	1.8	–2.3	2.7	6.0	1.9
X3LYP	0.8	1.9	2.7	0.5	7.0	7.0	8.9	2.4	3.1	3.8	8.9	2.9
					Meta–Hybrids							
M06	0.0	1.6	4.0	1.4	1.9	2.6	6.6	2.8	0.7	2.0	6.6	2.1
M06-2X	0.0	1.6	2.8	0.7	–0.8	0.8	1.4	0.4	–0.3	1.3	2.8	0.7
M06-HF	–0.6	1.6	5.1	1.7	–4.1	4.7	12.0	5.2	–2.0	2.8	12.0	3.8
TPSSH	–2.9	2.9	5.0	1.6	3.6	4.2	6.7	2.4	–0.4	3.4	6.7	2.1
					Double–Hybrids							
B2K-PLYP	–0.5	1.1	1.5	0.5	1.0	1.8	3.9	1.5	0.1	1.4	3.9	1.0
B2T-PLYP	–0.2	1.0	1.5	0.4	3.3	3.3	6.3	2.2	1.1	1.9	6.3	1.7
B2-PLYP	–0.6	1.2	2.1	0.7	4.8	4.8	8.3	2.6	1.4	2.6	8.3	2.4
LS1-TPSS	–3.2	3.2	5.4	1.8	–3.9	3.9	8.3	3.3	–3.4	3.4	8.3	2.5
mPW2K-PLYP	–0.6	1.2	1.8	0.5	1.0	1.8	3.6	1.3	0.0	1.4	3.6	0.9
mPW2-PLYP	0.1	0.9	1.8	0.5	3.8	3.8	6.3	1.8	1.5	2.0	6.3	1.8
PBE0-DH	–1.8	2.1	4.4	1.5	–5.7	5.7	8.8	2.2	–3.3	3.5	8.8	2.5
revDSD-BLYP	–0.6	1.2	2.6	0.8	0.9	2.0	3.5	1.1	–0.1	1.5	3.5	1.0
revDSD-PBE	–1.6	1.7	3.3	1.2	–1.4	2.6	4.7	1.4	–1.5	2.1	4.7	1.4
revDSD-PBEP86	0.0	0.8	1.2	0.4	0.5	2.2	4.0	1.4	0.2	1.3	4.0	1.1
					Range-Separated Hybrids							
CAM-B3LYP	2.4	2.4	3.8	1.3	1.6	1.6	2.5	0.6	2.1	2.1	3.8	1.2
CAMY-B3LYP	1.0	1.5	2.3	0.7	2.7	2.7	3.5	0.7	1.6	2.0	3.5	0.9
ωB97	3.5	3.5	4.2	0.4	–6.1	6.1	8.0	1.4	–0.1	4.5	8.0	1.6
ωB97X	2.6	2.6	3.8	0.8	–3.2	3.2	4.3	0.7	0.4	2.8	4.3	0.8
ωB97X-D	1.8	2.0	4.6	1.7	–0.2	0.9	1.7	0.6	1.1	1.6	4.6	1.5

Table S36 (continued).

XC	$\Delta E^{‡}$				ΔE_{rxn}				Total			
	ME	MAE	MUE	SD	ME	MAE	MUE	SD	ME	MAE	MUE	SD
					Dispersion–Corrected							
BP86-D3(BJ)	–9.2	9.2	15.5	3.9	2.2	2.2	4.9	1.9	–4.9	6.6	15.5	4.7
BLYP-D3(BJ)	–5.3	5.3	9.3	2.8	10.3	10.3	13.2	3.1	0.5	7.2	13.2	3.8
PBE-D3(BJ)	–5.8	8.9	15.5	4.0	2.2	2.2	4.9	1.9	–2.8	6.4	15.5	4.7
OLYP-D3(BJ)	–8.9	8.9	13.9	3.7	0.6	2.1	4.1	1.4	–5.3	6.3	13.9	4.5
OPBE-D3(BJ)	–12.6	12.6	20.2	5.0	–8.2	8.2	13.5	4.0	–11.0	11.0	20.2	5.1
B3LYP-D3(BJ)	–2.0	2.5	4.3	1.3	5.1	5.1	6.2	1.0	0.7	3.5	6.2	1.7
PBE0-D3(BJ)	–4.9	4.9	9.2	2.8	–4.6	4.6	7.4	1.9	–4.8	4.8	9.2	2.5
M06-2X-D3	–0.1	1.6	2.7	0.8	–0.9	0.9	1.5	0.4	–0.4	1.3	2.7	0.7

Table S37. Reaction barriers and reaction energies (kcal mol^{-1}) for the various pericyclic reactions, computed with XC/QZ4P//BP86/QZ4P.

XC	Diels-Alder Reaction		1,3-dipolar Cycloaddition		Electrocyclic Rearrangement		Sigmatropic Rearrangement		Double Group Transfer	
	$\Delta E^{\ddagger}$	ΔE_{rxn}	$\Delta E^{\ddagger}$	ΔE_{rxn}	$\Delta E^{\ddagger}$	ΔE_{rxn}	$\Delta E^{\ddagger}$	ΔE_{rxn}	$\Delta E^{\ddagger}$	ΔE_{rxn}
LDA										
VWN	0.8	–65.1	3.2	–42.3	36.3	2.4	21.9	0.0	22.1	0.0
GGAs										
BP86	15.0	–42.4	15.4	–20.8	39.6	8.3	28.2	0.0	38.1	0.0
BLYP	21.0	–30.2	19.2	–11.1	42.7	13.8	31.9	0.0	45.2	0.0
BEE	16.1	–43.1	17.2	–20.0	38.9	6.3	28.0	0.0	38.4	0.0
PW91	12.4	–46.2	13.3	–24.1	39.3	7.4	27.8	0.0	35.7	0.0
PBE	12.8	–46.3	13.9	–23.7	38.9	6.7	27.6	0.0	35.8	0.0
PBEsol	6.7	–56.8	8.8	–33.7	36.7	3.2	24.0	0.0	28.4	0.0
RPBE	18.8	–38.1	19.2	–15.8	40.0	8.2	29.8	0.0	41.7	0.0
revPBE	18.5	–39.0	19.0	–16.4	39.7	7.8	29.4	0.0	41.2	0.0
mPBE	14.4	–44.0	15.3	–21.6	39.3	7.2	28.2	0.0	37.4	0.0
mPW	15.5	–41.9	16.1	–19.8	39.9	8.3	28.8	0.0	38.9	0.0
HTBS	13.9	–47.1	15.5	–24.1	37.5	4.7	26.1	0.0	35.5	0.0
OLYP	24.0	–36.0	23.9	–13.6	40.4	7.1	30.8	0.0	45.4	0.0
OPBE	19.4	–48.5	21.9	–22.5	36.7	0.1	26.9	0.0	39.0	0.0
XLYP	21.2	–28.9	19.3	–10.1	43.2	14.7	32.5	0.0	45.9	0.0
Meta-GGAs										
M06-L	16.8	–45.9	19.8	–17.2	43.6	5.9	34.1	0.0	46.0	0.0
MVS	12.8	–54.2	18.6	–24.6	43.7	5.8	28.2	0.0	36.7	0.0
TPSS	15.5	–40.3	15.9	–19.8	40.2	7.7	30.9	0.0	42.9	0.0
revTPSS	14.9	–42.3	15.0	–22.8	39.6	6.3	31.5	0.0	44.8	0.0
Hybrids										
B3LYP	22.6	–37.5	21.7	–18.9	44.8	11.8	35.1	0.0	49.6	0.0
B3LYP*	20.6	–38.3	19.9	–19.2	43.8	11.4	33.5	0.0	46.7	0.0
B1LYP	24.0	–37.2	23.0	–19.2	45.7	12.1	36.4	0.0	51.9	0.0
B1PW91	19.6	–48.5	21.2	–26.9	42.4	5.7	33.0	0.0	46.2	0.0
BHandH	13.0	–66.3	14.9	–48.1	44.1	3.5	34.2	0.0	42.6	0.0
BHandHLYP	26.8	–44.4	26.5	–27.8	48.6	10.3	41.2	0.0	58.5	0.0
KMLYP	19.3	–60.2	21.0	–42.1	46.4	5.0	38.0	0.0	50.3	0.0
O3LYP	–1.7	–75.5	1.4	–53.0	37.2	0.2	22.9	0.0	21.8	0.0
OPBE0	21.4	–54.3	24.4	–30.0	40.0	–0.3	31.6	0.0	45.3	0.0
PBE0	16.6	–52.6	18.4	–30.9	41.8	4.8	32.2	0.0	43.1	0.0
mPW1PW	18.7	–48.9	20.2	–27.2	42.6	6.2	33.2	0.0	45.7	0.0
mPW1K	20.9	–54.0	22.9	–33.5	44.6	4.7	36.5	0.0	50.4	0.0
S12H	16.4	–53.2	19.4	–29.2	42.7	4.5	34.1	0.0	44.2	0.0
X3LYP	21.8	–39.1	21.1	–20.7	44.9	11.6	35.2	0.0	49.2	0.0
Meta-Hybrids										
M06	19.3	–47.6	22.4	–22.7	43.4	7.0	34.9	0.0	48.4	0.0
M06-2X	17.8	–48.7	21.2	–29.6	44.4	7.8	36.4	0.0	49.0	0.0
M06-HF	14.0	–48.4	18.8	–40.4	42.7	9.7	37.3	0.0	50.7	0.0
TPSSH	16.9	–43.2	17.6	–22.8	41.2	6.9	32.5	0.0	45.2	0.0
Double-Hybrids										
B2K-PLYP	17.7	–49.1	17.7	–25.2	43.9	7.9	35.3	0.0	49.2	0.0
B2T-PLYP	18.7	–45.8	18.4	–22.7	44.0	9.1	35.1	0.0	49.3	0.0
B2-PLYP	18.8	–43.7	18.2	–20.8	43.7	9.7	34.5	0.0	48.5	0.0
LS1-TPSS	13.4	–56.5	15.1	–29.4	41.6	4.1	33.2	0.0	45.5	0.0
mPW2K-PLYP	17.3	–49.2	17.3	–25.5	44.0	8.1	35.4	0.0	49.0	0.0
mPW2-PLYP	19.2	–44.7	18.6	–22.7	44.5	9.9	35.6	0.0	49.7	0.0
PBE0-DH	16.9	–56.5	18.9	–33.6	42.7	3.8	34.0	0.0	46.1	0.0
revDSD-BLYP	16.8	–49.2	17.0	–25.2	44.1	8.5	36.3	0.0	49.7	0.0
revDSD-PBE	16.2	–52.0	17.0	–26.8	42.9	6.2	35.5	0.0	47.8	0.0
revDSD-PBEP86	18.4	–49.3	18.7	–24.6	43.7	7.1	36.5	0.0	50.0	0.0
Range-Separated Hybrids										
CAM-B3LYP	23.5	–45.3	22.1	–27.8	45.9	9.2	37.2	0.0	51.7	0.0
CAMY-B3LYP	21.5	–44.4	20.7	–26.4	45.3	9.9	36.1	0.0	49.8	0.0
ωB97	23.1	–56.1	22.7	–35.2	46.3	3.1	40.2	0.0	53.2	0.0
ωB97X	22.6	–56.1	22.3	–32.3	45.8	5.4	38.7	0.0	51.8	0.0
ωB97X-D	23.0	–47.6	23.0	–28.0	44.6	6.3	36.4	0.0	50.3	0.0

Table S37 (continued).

XC	Diels-Alder Reaction		1,3-dipolar Cycloaddition		Electrocyclic Rearrangement		Sigmatropic Rearrangement		Double Group Transfer	
	$\Delta E^{\ddagger}$	ΔE_{rxn}	$\Delta E^{\ddagger}$	ΔE_{rxn}	$\Delta E^{\ddagger}$	ΔE_{rxn}	$\Delta E^{\ddagger}$	ΔE_{rxn}	$\Delta E^{\ddagger}$	ΔE_{rxn}
					Dispersion-Corrected					
BP86-D3(BJ)	8.8	–47.2	11.2	–24.7	39.4	8.4	27.7	0.0	34.7	0.0
BLYP-D3(BJ)	13.1	–36.5	13.8	–16.2	42.3	13.9	31.0	0.0	40.8	0.0
PBE-D3(BJ)	8.9	–49.4	11.2	–26.3	38.8	6.8	27.2	0.0	33.5	0.0
OLYP-D3(BJ)	7.7	–50.2	12.2	–25.9	39.0	6.9	27.5	0.0	36.1	0.0
OPBE-D3(BJ)	3.3	–62.3	10.6	–34.1	35.5	–0.1	24.1	0.0	29.9	0.0
B3LYP-D3(BJ)	16.1	–42.7	17.3	–23.1	44.5	11.9	34.4	0.0	46.0	0.0
PBE0-D3(BJ)	13.1	–55.3	16.0	–33.1	41.7	4.9	31.9	0.0	41.1	0.0
M06-2X-D3	17.7	–48.8	21.1	–29.7	44.4	7.8	36.4	0.0	48.9	0.0

Table S38. Errors in reaction barriers and reaction energies (kcal mol^{-1}) for the various pericyclic reactions, computed with XC/QZ4P//BP86/QZ4P, compared to CCSDT(Q)/CBS//CCSD(T)/cc-pVTZ.

XC	Diels-Alder Reaction		1,3-dipolar Cycloaddition		Electrocyclic Rearrangement		Sigmatropic Rearrangement		Double Group Transfer	
	$\Delta E^{\ddagger}$	ΔE_{rxn}	$\Delta E^{\ddagger}$	ΔE_{rxn}	$\Delta E^{\ddagger}$	ΔE_{rxn}	$\Delta E^{\ddagger}$	ΔE_{rxn}	$\Delta E^{\ddagger}$	ΔE_{rxn}
LDA										
VWN	–18.8	–17.5	–15.1	–13.4	–7.1	–6.1	–14.9	0.0	–28.1	0.0
GGAs										
BP86	–4.6	5.3	–2.9	8.1	–3.8	–0.1	–8.6	0.0	–12.1	0.0
BLYP	1.4	17.5	0.9	17.8	–0.7	5.4	–4.9	0.0	–5.0	0.0
BEE	–3.5	4.6	–1.1	8.9	–4.5	–2.2	–8.8	0.0	–11.8	0.0
PW91	–7.2	1.5	–5.0	4.8	–4.1	–1.1	–9.0	0.0	–14.5	0.0
PBE	–6.8	1.4	–4.4	5.2	–4.5	–1.8	–9.2	0.0	–14.4	0.0
PBEsol	–12.9	–9.2	–9.5	–4.8	–6.7	–5.3	–12.8	0.0	–21.8	0.0
RPBE	–0.8	9.6	0.9	13.1	–3.4	–0.3	–7.0	0.0	–8.5	0.0
revPBE	–1.1	8.7	0.7	12.5	–3.7	–0.6	–7.4	0.0	–9.0	0.0
mPBE	–5.2	3.7	–3.0	7.3	–4.1	–1.3	–8.6	0.0	–12.8	0.0
mPW	–4.1	5.8	–2.2	9.1	–3.5	–0.1	–8.0	0.0	–11.3	0.0
HTBS	–5.7	0.5	–2.8	4.8	–5.9	–3.8	–10.7	0.0	–14.7	0.0
OLYP	4.4	11.7	5.6	15.3	–3.0	–1.4	–6.0	0.0	–4.8	0.0
OPBE	–0.2	–0.9	3.6	6.4	–6.7	–8.4	–9.9	0.0	–11.2	0.0
XLYP	1.6	18.8	1.0	18.8	–0.2	6.3	–4.3	0.0	–4.3	0.0
Meta-GGAs										
M06-L	–2.8	1.8	1.5	11.7	0.2	–2.6	–2.7	0.0	–4.2	0.0
MVS	–6.8	–6.6	0.3	4.3	0.3	–2.7	–8.6	0.0	–13.5	0.0
TPSS	–4.1	7.4	–2.4	9.1	–3.2	–0.7	–5.9	0.0	–7.3	0.0
revTPSS	–4.7	5.4	–3.3	6.1	–3.8	–2.2	–5.3	0.0	–5.4	0.0
Hybrids										
B3LYP	3.0	10.2	3.4	10.0	1.4	3.4	–1.7	0.0	–0.6	0.0
B3LYP*	1.0	9.4	1.6	9.7	0.4	3.0	–3.3	0.0	–3.5	0.0
B1LYP	4.4	10.5	4.7	9.7	2.3	3.7	–0.4	0.0	1.7	0.0
B1PW91	0.0	–0.9	2.9	2.0	–1.0	–2.8	–3.8	0.0	–4.0	0.0
BHandH	–6.6	–18.7	–3.4	–19.2	0.7	–5.0	–2.6	0.0	–7.6	0.0
BHandHLYP	7.2	3.3	8.2	1.1	5.2	1.9	4.4	0.0	8.3	0.0
KMLYP	–0.3	–12.6	2.7	–13.2	3.0	–3.5	1.2	0.0	0.1	0.0
O3LYP	–21.3	–27.9	–16.9	–24.1	–6.2	–8.3	–13.9	0.0	–28.4	0.0
OPBE0	1.8	–6.7	6.1	–1.1	–3.4	–8.8	–5.2	0.0	–4.9	0.0
PBE0	–3.0	–5.0	0.1	–2.0	–1.6	–3.7	–4.6	0.0	–7.1	0.0
mPW1PW	–0.9	–1.3	1.9	1.7	–0.8	–2.3	–3.6	0.0	–4.5	0.0
mPW1K	1.3	–6.4	4.6	–4.6	1.2	–3.8	–0.3	0.0	0.2	0.0
S12H	–3.2	–5.6	1.1	–0.3	–0.7	–4.0	–2.7	0.0	–6.0	0.0
X3LYP	2.2	8.6	2.8	8.2	1.5	3.2	–1.6	0.0	–1.0	0.0
Meta-Hybrids										
M06	–0.3	0.0	4.1	6.2	0.0	–1.5	–1.9	0.0	–1.8	0.0
M06-2X	–1.8	–1.1	2.9	–0.7	1.0	–0.6	–0.4	0.0	–1.2	0.0
M06-HF	–5.6	–0.8	0.5	–11.5	–0.7	1.3	0.5	0.0	0.5	0.0
TPSSH	–2.7	4.5	–0.7	6.1	–2.2	–1.6	–4.3	0.0	–5.0	0.0
Double-Hybrids										
B2K-PLYP	–1.9	–1.5	–0.6	3.7	0.5	–0.5	–1.5	0.0	–1.0	0.0
B2T-PLYP	–0.9	1.9	0.1	6.2	0.6	0.7	–1.7	0.0	–0.9	0.0
B2-PLYP	–0.8	4.0	–0.1	8.1	0.3	1.3	–2.3	0.0	–1.7	0.0
LS1-TPSS	–6.2	–8.9	–3.2	–0.5	–1.8	–4.4	–3.6	0.0	–4.7	0.0
mPW2K-PLYP	–2.3	–1.6	–1.0	3.4	0.6	–0.4	–1.4	0.0	–1.2	0.0
mPW2-PLYP	–0.4	3.0	0.3	6.2	1.1	1.5	–1.2	0.0	–0.5	0.0
PBE0-DH	–2.7	–8.9	0.6	–4.7	–0.7	–4.7	–2.8	0.0	–4.1	0.0
revDSD-BLYP	–2.8	–1.6	–1.3	3.7	0.7	0.1	–0.5	0.0	–0.5	0.0
revDSD-PBE	–3.4	–4.4	–1.3	2.1	–0.5	–2.3	–1.3	0.0	–2.4	0.0
revDSD-PBEP86	–1.2	–1.7	0.4	4.3	0.3	–1.4	–0.3	0.0	–0.2	0.0
Range-Separated Hybrids										
CAM-B3LYP	3.9	2.4	3.8	1.1	2.5	0.8	0.4	0.0	1.5	0.0
CAMY-B3LYP	1.9	3.3	2.4	2.5	1.9	1.5	–0.7	0.0	–0.4	0.0
ωB97	3.5	–8.5	4.4	–6.3	2.9	–5.4	3.4	0.0	3.0	0.0
ωB97X	3.0	–8.5	4.0	–3.4	2.4	–3.1	1.9	0.0	1.6	0.0
ωB97X-D	3.4	0.0	4.7	0.9	1.2	–2.2	–0.4	0.0	0.1	0.0

Table S38 (continued).

XC	Diels-Alder Reaction		1,3-dipolar Cycloaddition		Electrocyclic Rearrangement		Sigmatropic Rearrangement		Double Group Transfer	
	$\Delta E^{\ddagger}$	ΔE_{rxn}	$\Delta E^{\ddagger}$	ΔE_{rxn}	$\Delta E^{\ddagger}$	ΔE_{rxn}	$\Delta E^{\ddagger}$	ΔE_{rxn}	$\Delta E^{\ddagger}$	ΔE_{rxn}
					Dispersion-Corrected					
BP86-D3(BJ)	–10.8	0.4	–7.1	4.2	–4.0	0.0	–9.1	0.0	–15.5	0.0
BLYP-D3(BJ)	–6.5	11.2	–4.5	12.7	–1.1	5.5	–5.8	0.0	–9.4	0.0
PBE-D3(BJ)	–10.7	–1.8	–7.1	2.6	–4.6	–1.7	–9.6	0.0	–16.7	0.0
OLYP-D3(BJ)	–11.9	–2.6	–6.1	3.0	–4.4	–1.6	–9.3	0.0	–14.1	0.0
OPBE-D3(BJ)	–16.3	–14.7	–7.7	–5.2	–7.9	–8.6	–12.7	0.0	–20.3	0.0
B3LYP-D3(BJ)	–3.5	5.0	–1.0	5.8	1.1	3.5	–2.4	0.0	–4.2	0.0
PBE0-D3(BJ)	–6.5	–7.7	–2.3	–4.2	–1.7	–3.6	–4.9	0.0	–9.1	0.0
M06-2X-D3	–1.9	–1.2	2.8	–0.8	1.0	–0.6	–0.4	0.0	–1.3	0.0

Table S39. Mean error (ME), mean absolute error (MAE), maximum unsigned error (MUE) and standard deviation (SD) of reaction barriers and energies (kcal mol^{-1}) for the various pericyclic reactions, computed with XC/QZ4P//BP86/QZ4P compared to CCSDT(Q)/CBS//CCSD(T)/cc-pVTZ.

XC	$\Delta E^{\ddagger}$				ΔE_{rxn}				Total			
	ME	MAE	MUE	SD	ME	MAE	MUE	SD	ME	MAE	MUE	SD
					LDA							
VWN	–16.8	16.8	28.1	6.8	–12.3	12.3	17.5	4.7	–15.1	15.1	28.1	6.5
					GGAs							
BP86	–6.4	6.4	12.1	3.5	4.4	4.5	8.1	3.3	–2.4	5.7	12.1	3.5
BLYP	–1.7	2.6	5.0	1.9	13.5	13.5	17.8	5.8	4.0	6.7	17.8	6.5
BEE	–5.9	5.9	11.8	3.9	3.8	5.2	8.9	2.8	–2.3	5.7	11.8	3.5
PW91	–8.0	8.0	14.5	3.7	1.7	2.4	4.8	1.7	–4.3	5.9	14.5	4.1
PBE	–7.9	7.9	14.4	3.7	1.6	2.8	5.2	1.7	–4.3	5.9	14.4	4.0
PBEsol	–12.7	12.7	21.8	5.1	–6.4	6.4	9.2	1.9	–10.4	10.4	21.8	5.2
RPBE	–3.8	4.1	8.5	3.2	7.5	7.6	13.1	5.4	0.4	5.4	13.1	4.5
revPBE	–4.1	4.4	9.0	3.3	6.8	7.3	12.5	4.9	0.0	5.5	12.5	4.2
mPBE	–6.7	6.7	12.8	3.6	3.2	4.1	7.3	2.5	–3.0	5.7	12.8	3.5
mPW	–5.8	5.8	11.3	3.4	4.9	5.0	9.1	3.7	–1.8	5.5	11.3	3.5
HTBS	–8.0	8.0	14.7	4.2	0.5	3.0	4.8	1.8	–4.8	6.1	14.7	4.3
OLYP	–0.8	4.8	6.0	1.0	8.5	9.4	15.3	5.9	2.7	6.5	15.3	4.3
OPBE	–4.9	6.3	11.2	4.1	–0.9	5.2	8.4	3.2	–3.4	5.9	11.2	3.8
XLYP	–1.2	2.3	4.3	1.7	14.6	14.6	18.8	5.9	4.7	6.9	18.8	7.1
					Meta–GGAs							
M06-L	–1.6	2.3	4.2	1.4	3.6	5.3	11.7	4.5	0.4	3.4	11.7	3.3
MVS	–5.7	5.9	13.5	5.1	–1.6	4.5	6.6	1.6	–4.2	5.4	13.5	4.2
TPSS	–4.6	4.6	7.3	1.8	5.2	5.7	9.1	3.6	–0.9	5.0	9.1	2.7
revTPSS	–4.5	4.5	5.4	0.8	3.1	4.5	6.1	1.7	–1.7	4.5	6.1	1.2
					Hybrids							
B3LYP	1.1	2.0	3.4	1.0	7.8	7.8	10.2	3.2	3.6	4.2	10.2	3.5
B3LYP*	–0.8	2.0	3.5	1.2	7.3	7.3	9.7	3.1	2.3	4.0	9.7	3.4
B1LYP	2.5	2.7	4.7	1.7	7.9	7.9	10.5	3.0	4.6	4.7	10.5	3.4
B1PW91	–1.2	2.4	4.0	1.6	–0.5	1.9	2.8	0.8	–0.9	2.2	4.0	1.3
BHandH	–3.9	4.2	7.6	2.6	–14.3	14.3	19.2	6.6	–7.8	8.0	19.2	6.7
BHandHLYP	6.7	6.7	8.3	1.6	2.1	2.1	3.3	0.9	4.9	4.9	8.3	2.6
KMLYP	1.3	1.4	3.0	1.2	–9.7	9.7	13.2	4.5	–2.8	4.6	13.2	5.0
O3LYP	–17.3	17.3	28.4	7.4	–20.1	20.1	27.9	8.5	–18.4	18.4	28.4	7.9
OPBE0	–1.1	4.3	6.1	1.5	–5.5	5.5	8.8	3.2	–2.8	4.8	8.8	2.4
PBE0	–3.2	3.3	7.1	2.4	–3.5	3.5	5.0	1.2	–3.4	3.4	7.1	2.1
mPW1PW	–1.6	2.3	4.5	1.5	–0.6	1.7	2.3	0.4	–1.2	2.1	4.5	1.2
mPW1K	1.4	1.5	4.6	1.6	–4.9	4.9	6.4	1.1	–1.0	2.8	6.4	2.2
S12H	–2.3	2.7	6.0	1.9	–3.3	3.3	5.6	2.2	–2.7	2.9	6.0	2.0
X3LYP	0.8	1.8	2.8	0.6	6.6	6.6	8.6	2.5	3.0	3.6	8.6	2.8
					Meta–Hybrids							
M06	0.0	1.6	4.1	1.5	1.6	2.6	6.2	2.6	0.6	2.0	6.2	2.0
M06-2X	0.1	1.5	2.9	0.9	–0.8	0.8	1.1	0.2	–0.2	1.2	2.9	0.7
M06-HF	–1.0	1.6	5.6	2.0	–3.7	4.5	11.5	5.0	–2.0	2.7	11.5	3.7
TPSSH	–3.0	3.0	5.0	1.5	3.0	4.0	6.1	1.9	–0.7	3.4	6.1	1.7
					Double–Hybrids							
B2K-PLYP	–0.9	1.1	1.9	0.5	0.6	1.9	3.7	1.3	–0.4	1.4	3.7	1.0
B2T-PLYP	–0.6	0.8	1.7	0.5	2.9	2.9	6.2	2.4	0.7	1.6	6.2	1.8
B2-PLYP	–0.9	1.0	2.3	0.9	4.4	4.4	8.1	2.8	1.1	2.3	8.1	2.5
LS1-TPSS	–3.9	3.9	6.2	1.5	–4.6	4.6	8.9	3.4	–4.2	4.2	8.9	2.4
mPW2K-PLYP	–1.1	1.3	2.3	0.6	0.5	1.8	3.4	1.2	–0.5	1.5	3.4	0.9
mPW2-PLYP	–0.1	0.7	1.2	0.4	3.5	3.5	6.2	2.0	1.2	1.8	6.2	1.8
PBE0-DH	–1.9	2.2	4.1	1.3	–6.1	6.1	8.9	2.0	–3.5	3.6	8.9	2.5
revDSD-BLYP	–0.9	1.1	2.8	0.9	0.7	1.8	3.7	1.5	–0.3	1.4	3.7	1.2
revDSD-PBE	–1.8	1.8	3.4	1.0	–1.5	2.9	4.4	1.0	–1.7	2.2	4.4	1.1
revDSD-PBEP86	–0.2	0.5	1.2	0.4	0.4	2.4	4.3	1.3	0.0	1.2	4.3	1.3
					Range-Separated Hybrids							
CAM-B3LYP	2.4	2.4	3.9	1.4	1.4	1.4	2.4	0.7	2.0	2.0	3.9	1.3
CAMY-B3LYP	1.0	1.5	2.4	0.8	2.4	2.4	3.3	0.7	1.5	1.8	3.3	0.9
ωB97	3.4	3.4	4.4	0.5	–6.7	6.7	8.5	1.3	–0.4	4.7	8.5	1.8
ωB97X	2.6	2.6	4.0	0.9	–5.0	5.0	8.5	2.5	–0.3	3.5	8.5	2.0
ωB97X-D	1.8	2.0	4.7	1.8	–0.4	1.0	2.2	0.9	1.0	1.6	4.7	1.6

Table S39 (continued).

XC	$\Delta E^{\ddagger}$				ΔE_{rxn}				Total			
	ME	MAE	MUE	SD	ME	MAE	MUE	SD	ME	MAE	MUE	SD
					Dispersion–Corrected							
BP86-D3(BJ)	–9.3	9.3	15.5	3.8	1.5	1.6	4.2	1.9	–5.2	6.4	15.5	5.0
BLYP-D3(BJ)	–5.5	5.5	9.4	2.7	9.8	9.8	12.7	3.1	0.2	7.1	12.7	3.5
PBE-D3(BJ)	–9.7	9.7	16.7	4.1	–0.3	2.0	2.6	0.4	–6.2	6.8	16.7	5.0
OLYP-D3(BJ)	–9.2	9.2	14.1	3.6	–0.4	2.4	3.0	0.6	–5.9	6.6	14.1	4.4
OPBE-D3(BJ)	–13.0	13.0	20.3	4.9	–9.5	9.5	14.7	3.9	–11.7	11.7	20.3	4.8
B3LYP-D3(BJ)	–2.0	2.4	4.2	1.3	4.7	4.7	5.8	1.0	0.5	3.3	5.8	1.6
PBE0-D3(BJ)	–4.9	4.9	9.1	2.7	–5.1	5.1	7.7	1.8	–5.0	5.0	9.1	2.4
M06-2X-D3	0.0	1.5	2.8	0.8	–0.9	0.9	1.2	0.2	–0.3	1.2	2.8	0.7

Table S40. Reaction barriers and reaction energies (kcal mol^{-1}) for the various pericyclic reactions, computed with XC/TZ2P//BP86-D3(BJ)/QZ4P.

XC	Diels-Alder Reaction		1,3-dipolar Cycloaddition		Electrocyclic Rearrangement		Sigmatropic Rearrangement		Double Group Transfer	
	$\Delta E^{\ddagger}$	ΔE_{rxn}	$\Delta E^{\ddagger}$	ΔE_{rxn}	$\Delta E^{\ddagger}$	ΔE_{rxn}	$\Delta E^{\ddagger}$	ΔE_{rxn}	$\Delta E^{\ddagger}$	ΔE_{rxn}
LDA										
VWN	1.0	–64.6	3.3	–41.5	36.7	3.0	22.2	0.0	22.1	0.0
GGAs										
BP86	15.2	–41.6	15.5	–20.1	39.9	8.8	28.2	0.0	38.1	0.0
BLYP	21.1	–29.6	19.4	–10.5	43.0	14.3	31.8	0.0	45.2	0.0
BEE	16.4	–42.3	17.4	–19.1	39.3	7.1	28.1	0.0	38.5	0.0
PW91	12.6	–45.5	13.5	–23.3	39.6	8.0	27.9	0.0	35.8	0.0
PBE	13.1	–45.6	14.1	–22.9	39.3	7.4	27.6	0.0	35.9	0.0
PBEsol	7.0	–56.2	9.1	–32.8	37.1	3.8	24.1	0.0	28.5	0.0
RPBE	19.0	–37.3	19.4	–14.9	40.3	8.9	29.8	0.0	41.8	0.0
revPBE	18.7	–38.1	19.3	–15.5	40.1	8.5	29.4	0.0	41.3	0.0
mPBE	14.6	–43.3	15.5	–20.8	39.6	7.9	28.3	0.0	37.5	0.0
mPW	15.7	–41.2	16.3	–19.0	40.2	8.9	28.9	0.0	39.0	0.0
HTBS	14.2	–46.4	15.8	–23.1	37.9	5.4	26.1	0.0	35.5	0.0
OLYP	24.3	–35.1	24.2	–12.6	40.9	8.0	30.8	0.0	45.6	0.0
OPBE	19.8	–47.4	22.4	–21.0	37.4	1.2	27.1	0.0	39.1	0.0
XLYP	21.4	–28.3	19.4	–9.7	43.4	15.1	32.4	0.0	45.9	0.0
Meta-GGAs										
M06-L	16.5	–44.9	19.5	–16.6	43.9	6.9	34.3	0.0	46.1	0.0
MVS	12.1	–54.3	17.9	–24.6	43.1	6.5	28.4	0.0	36.8	0.0
TPSS	15.6	–39.5	16.1	–19.0	40.6	8.5	31.0	0.0	42.9	0.0
revTPSS	14.6	–41.7	15.1	–22.1	40.0	6.9	31.5	0.0	44.8	0.0
Hybrids										
B3LYP	22.5	–37.2	21.6	–18.5	45.1	12.2	35.0	0.0	49.5	0.0
B3LYP*	20.6	–37.9	19.8	–18.8	44.1	11.9	33.4	0.0	46.6	0.0
B1LYP	23.8	–37.0	22.8	–18.9	46.0	12.5	36.4	0.0	51.8	0.0
B1PW91	19.5	–48.2	21.1	–26.4	42.8	6.3	33.1	0.0	46.0	0.0
BHandH	12.5	–66.6	14.6	–47.9	44.5	4.0	34.2	0.0	42.4	0.0
BHandHLYP	26.4	–44.6	26.2	–27.7	48.9	10.7	41.1	0.0	58.3	0.0
KMLYP	18.7	–60.5	20.6	–42.0	46.8	5.5	38.0	0.0	50.1	0.0
O3LYP	–1.7	–75.3	1.4	–52.5	37.8	0.8	23.3	0.0	21.8	0.0
OPBE0	21.4	–53.7	24.5	–28.9	40.7	0.7	31.7	0.0	45.3	0.0
PBE0	16.5	–52.3	18.3	–30.4	42.3	5.5	32.2	0.0	43.0	0.0
mPW1PW	18.6	–48.6	20.1	–27.2	43.1	6.8	33.3	0.0	45.6	0.0
mPW1K	20.6	–54.0	22.7	–33.2	45.0	5.3	36.5	0.0	50.2	0.0
S12H	16.3	–52.5	19.2	–28.4	43.2	5.4	34.2	0.0	44.3	0.0
X3LYP	21.7	–38.8	21.0	–20.3	45.2	12.0	35.1	0.0	49.1	0.0
Meta-Hybrids										
M06	19.1	–47.5	22.1	–22.3	43.8	7.4	35.0	0.0	48.4	0.0
M06-2X	17.5	–49.0	20.8	–29.8	44.7	8.2	36.3	0.0	48.7	0.0
M06-HF	14.4	–48.9	19.1	–40.9	43.0	9.3	37.3	0.0	50.8	0.0
TPSSH	16.9	–42.6	17.7	–22.2	41.7	7.6	32.5	0.0	45.2	0.0
Double-Hybrids										
B2K-PLYP	18.0	–48.8	18.3	–25.1	44.8	9.1	36.0	0.0	49.0	0.0
B2T-PLYP	18.9	–45.6	18.8	–22.6	44.8	10.0	35.6	0.0	49.0	0.0
B2-PLYP	19.0	–43.5	18.6	–20.7	44.4	10.6	35.0	0.0	48.3	0.0
LS1-TPSS	14.0	–56.1	15.8	–29.2	42.9	5.6	34.2	0.0	45.4	0.0
mPW2K-PLYP	17.6	–49.0	17.9	–25.4	44.9	9.2	36.1	0.0	48.8	0.0
mPW2-PLYP	19.3	–44.6	18.9	–22.6	45.2	10.7	36.0	0.0	49.4	0.0
PBE0-DH	16.8	–56.5	19.0	–33.3	43.3	4.6	34.3	0.0	45.9	0.0
revDSD-BLYP	16.8	–49.4	17.3	–25.5	45.0	9.4	36.9	0.0	49.2	0.0
revDSD-PBE	16.1	–52.3	17.2	–27.1	43.8	7.2	36.0	0.0	47.2	0.0
revDSD-PBEP86	18.3	–49.6	18.9	–25.0	44.6	8.1	37.0	0.0	49.4	0.0
Range-Separated Hybrids										
CAM-B3LYP	23.2	–45.2	21.9	–27.6	46.3	9.6	37.2	0.0	51.6	0.0
CAMY-B3LYP	21.3	–44.2	20.5	–26.2	45.6	10.3	36.1	0.0	49.7	0.0
ωB97	22.7	–55.6	22.3	–34.7	46.8	4.0	40.2	0.0	53.3	0.0
ωB97X	22.3	–52.0	21.8	–32.0	46.3	6.0	38.7	0.0	51.8	0.0
ωB97X-D	22.8	–47.5	22.7	–27.7	45.0	6.8	36.4	0.0	50.3	0.0

Table S40 (continued).

XC	Diels-Alder Reaction		1,3-dipolar Cycloaddition		Electrocyclic Rearrangement		Sigmatropic Rearrangement		Double Group Transfer	
	$\Delta E^{\ddagger}$	ΔE_{rxn}	$\Delta E^{\ddagger}$	ΔE_{rxn}	$\Delta E^{\ddagger}$	ΔE_{rxn}	$\Delta E^{\ddagger}$	ΔE_{rxn}	$\Delta E^{\ddagger}$	ΔE_{rxn}
					Dispersion-Corrected					
BP86-D3(BJ)	9.0	–46.5	11.3	–24.0	39.7	9.0	27.7	0.0	34.7	0.0
BLYP-D3(BJ)	13.2	–35.9	13.9	–15.7	42.5	14.4	31.0	0.0	40.8	0.0
PBE-D3(BJ)	9.1	–48.7	11.4	–25.5	39.2	7.5	27.3	0.0	33.6	0.0
OLYP-D3(BJ)	7.9	–49.4	12.4	–24.9	39.6	7.8	27.7	0.0	36.3	0.0
OPBE-D3(BJ)	3.6	–61.2	10.9	–32.7	36.2	1.1	24.4	0.0	30.0	0.0
B3LYP-D3(BJ)	16.0	–42.4	17.2	–22.8	44.8	12.3	34.4	0.0	45.9	0.0
PBE0-D3(BJ)	13.0	–55.0	15.9	–32.6	42.1	5.6	31.9	0.0	41.0	0.0
M06-2X-D3	17.3	–49.2	20.7	–29.9	44.7	8.2	36.3	0.0	48.6	0.0

Table S41. Errors in reaction barriers and reaction energies (kcal mol^{-1}) for the various pericyclic reactions, computed with XC/TZ2P//BP86-D3(BJ)/QZ4P, compared to CCSDT(Q)/CBS//CCSD(T)/cc-pVTZ.

XC	Diels-Alder Reaction		1,3-dipolar Cycloaddition		Electrocyclic Rearrangement		Sigmatropic Rearrangement		Double Group Transfer	
	$\Delta E^{\ddagger}$	ΔE_{rxn}	$\Delta E^{\ddagger}$	ΔE_{rxn}	$\Delta E^{\ddagger}$	ΔE_{rxn}	$\Delta E^{\ddagger}$	ΔE_{rxn}	$\Delta E^{\ddagger}$	ΔE_{rxn}
LDA										
VWN	–18.8	–17.5	–15.1	–13.4	–7.1	–6.0	–14.7	0.0	–28.1	0.0
GGAs										
BP86	–4.6	0.4	–2.9	8.1	–3.8	–0.3	–8.6	0.0	–12.1	0.0
BLYP	1.4	17.6	0.9	17.9	–0.7	5.4	–5.0	0.0	–5.0	0.0
BEE	–3.4	4.6	–1.1	8.9	–4.5	–2.2	–8.8	0.0	–11.8	0.0
PW91	–7.2	1.5	–5.0	4.8	–4.1	–1.1	–9.0	0.0	–14.4	0.0
PBE	–6.8	1.4	–4.4	5.1	–4.5	–1.8	–9.2	0.0	–11.3	0.0
PBEsol	–12.9	–9.3	–9.4	–4.8	–6.7	–5.3	–12.7	0.0	–21.8	0.0
RPBE	–0.8	9.6	0.9	13.1	–3.5	–0.3	–7.0	0.0	–8.5	0.0
revPBE	–1.1	8.8	0.7	12.5	–3.7	–0.6	–7.5	0.0	–9.0	0.0
mPBE	–5.2	3.7	–3.0	7.3	–4.2	–1.3	–8.6	0.0	–12.8	0.0
mPW	–4.1	5.8	–2.2	9.1	–3.5	–0.1	–8.0	0.0	–11.3	0.0
HTBS	–5.6	0.5	–2.8	4.8	–6.0	–3.9	–10.7	0.0	–14.7	0.0
OLYP	4.4	11.7	5.6	–7.1	–3.0	–1.4	–6.1	0.0	–4.8	0.0
OPBE	–0.2	–0.9	3.7	6.4	–6.7	–8.5	–9.9	0.0	–11.2	0.0
XLYP	1.6	18.8	1.0	18.8	–0.2	6.2	–4.4	0.0	–4.3	0.0
Meta-GGAs										
M06-L	–2.9	1.7	1.4	11.7	0.3	–2.5	–2.6	0.0	–4.2	0.0
MVS	–6.9	–6.7	0.1	4.2	–0.7	–2.6	–8.4	0.0	–13.4	0.0
TPSS	–4.1	7.4	–2.4	9.1	–3.2	–0.7	–5.9	0.0	–7.3	0.0
revTPSS	–5.1	5.4	–3.3	6.0	–3.8	–2.3	–5.3	0.0	–5.4	0.0
Hybrids										
B3LYP	2.9	10.2	3.4	10.0	1.4	3.4	–1.8	0.0	–0.6	0.0
B3LYP*	1.0	9.4	1.5	9.7	0.4	3.1	–3.3	0.0	–3.5	0.0
B1LYP	4.3	10.4	4.6	9.7	2.3	3.7	–0.4	0.0	1.7	0.0
B1PW91	–0.1	–0.9	2.8	2.0	–1.0	–2.8	–3.7	0.0	–4.0	0.0
BHandH	–6.8	–18.8	–3.6	–19.3	0.9	–4.9	–2.5	0.0	–7.6	0.0
BHandHLYP	7.0	3.2	8.1	1.1	5.3	1.9	4.4	0.0	8.3	0.0
KMLYP	–0.6	–12.7	2.5	–13.3	3.2	–3.4	1.3	0.0	0.1	0.0
O3LYP	–21.5	–28.1	–17.0	–24.2	–5.9	–7.2	–13.5	0.0	–28.3	0.0
OPBE0	1.7	–6.8	6.1	–1.1	–3.3	–8.8	–5.1	0.0	–4.9	0.0
PBE0	–3.1	–5.0	0.0	–2.0	–1.5	–3.6	–4.6	0.0	–7.1	0.0
mPW1PW	–1.0	–1.4	1.8	1.2	–0.7	–2.2	–3.5	0.0	–4.5	0.0
mPW1K	–3.1	–5.0	0.0	–2.0	–1.5	–3.6	–4.6	0.0	–7.1	0.0
S12H	–3.3	–5.6	0.9	–0.3	–0.6	–4.0	–2.6	0.0	–6.0	0.0
X3LYP	2.1	8.6	2.7	8.2	1.5	3.2	–1.7	0.0	–1.0	0.0
Meta-Hybrids										
M06	–0.4	–0.1	3.9	6.2	0.1	–1.5	–1.8	0.0	–1.7	0.0
M06-2X	–1.9	–1.1	2.7	–0.8	1.1	–0.6	0.6	0.0	–1.2	0.0
M06-HF	–5.8	–0.8	0.1	–11.6	–0.6	1.0	0.5	0.0	0.4	0.0
TPSSH	–2.8	4.5	–0.7	6.0	–2.1	–1.6	–4.3	0.0	–5.0	0.0
Double-Hybrids										
B2K-PLYP	–1.6	–1.0	–0.9	3.6	0.9	0.6	1.8	0.0	–1.0	0.0
B2T-PLYP	–0.6	2.3	–0.1	6.1	0.9	1.5	0.6	0.0	–0.9	0.0
B2-PLYP	–0.6	4.3	–0.3	8.0	0.5	2.0	–0.3	0.0	–1.7	0.0
LS1-TPSS	–5.5	–8.2	–3.6	–0.6	–1.2	–3.0	1.9	0.0	–4.6	0.0
mPW2K-PLYP	–1.9	–1.1	–1.3	3.3	1.0	0.8	1.8	0.0	–1.2	0.0
mPW2-PLYP	–0.2	3.3	0.2	6.1	1.4	2.2	0.7	0.0	–0.5	0.0
PBE0-DH	–2.7	–8.8	0.5	–4.8	–0.5	–4.3	–1.7	0.0	–4.1	0.0
revDSD-BLYP	–2.3	–1.0	–1.6	3.6	1.2	1.2	2.4	0.0	–0.6	0.0
revDSD-PBE	–2.9	–3.8	–1.6	2.0	0.0	–1.2	1.4	0.0	–2.4	0.0
revDSD-PBEP86	–0.7	–1.1	0.1	4.2	0.8	–0.3	2.4	0.0	–0.2	0.0
Range-Separated Hybrids										
CAM-B3LYP	3.6	2.5	3.6	1.3	2.9	1.2	0.4	0.0	1.4	0.0
CAMY-B3LYP	1.7	3.5	2.2	2.7	2.2	1.9	–0.7	0.0	–0.5	0.0
ωB97	3.1	–8.0	4.0	–5.8	3.4	–4.5	3.4	0.0	3.1	0.0
ωB97X	2.7	–4.4	3.5	–3.1	2.9	–2.5	1.9	0.0	1.6	0.0
ωB97X-D	3.2	0.1	4.4	1.2	1.6	–1.7	–0.4	0.0	0.1	0.0

Table S41 (continued).

XC	Diels-Alder Reaction		1,3-dipolar Cycloaddition		Electrocyclic Rearrangement		Sigmatropic Rearrangement		Double Group Transfer	
	$\Delta E^{\ddagger}$	ΔE_{rxn}	$\Delta E^{\ddagger}$	ΔE_{rxn}	$\Delta E^{\ddagger}$	ΔE_{rxn}	$\Delta E^{\ddagger}$	ΔE_{rxn}	$\Delta E^{\ddagger}$	ΔE_{rxn}
Dispersion-Corrected										
BP86-D3(BJ)	–10.8	0.4	–7.1	4.2	–4.0	0.0	–9.1	0.0	–15.5	0.0
BLYP-D3(BJ)	–6.6	11.2	–4.5	12.7	–1.1	5.5	–5.9	0.0	–9.4	0.0
PBE-D3(BJ)	–10.7	–1.9	–7.1	2.6	–4.6	–1.7	–9.6	0.0	–16.6	0.0
OLYP-D3(BJ)	–12.0	–2.7	–6.2	3.0	–4.4	–1.6	–9.3	0.0	–14.1	0.0
OPBE-D3(BJ)	–15.4	–13.7	–7.8	–5.3	–6.9	–7.6	–12.6	0.0	–20.3	0.0
B3LYP-D3(BJ)	–3.6	5.0	–1.1	5.8	1.1	3.5	–2.4	0.0	–4.2	0.0
PBE0-D3(BJ)	–6.6	–7.8	–2.4	–4.3	–1.7	–3.5	–4.8	0.0	–9.1	0.0
M06-2X-D3	–2.0	–1.2	2.6	–0.8	1.1	–0.6	–0.3	0.0	–1.3	0.0

Table S42. Mean error (ME), mean absolute error (MAE), maximum unsigned error (MUE) and standard deviation (SD) of reaction barriers and energies (kcal mol^{-1}) for the various pericyclic reactions, computed with XC/TZ2P//BP86-D3(BJ)/QZ4P compared to CCSDT(Q)/CBS//CCSD(T)/cc-pVTZ.

XC	$\Delta E^{\ddagger}$				ΔE_{rxn}				Total			
	ME	MAE	MUE	SD	ME	MAE	MUE	SD	ME	MAE	MUE	SD
					LDA							
VWN	–16.6	16.6	28.1	6.9	–11.7	11.7	17.0	4.7	–14.8	14.8	28.1	6.6
					GGAs							
BP86	–6.3	6.3	12.1	3.5	5.1	5.1	8.8	3.5	–2.0	5.8	12.1	3.6
BLYP	–1.6	2.6	5.0	2.0	14.1	14.1	18.4	5.8	4.3	6.9	18.4	6.8
BEE	–5.7	5.7	11.7	3.9	4.6	5.5	9.8	3.4	–1.9	5.6	11.7	3.8
PW91	–7.8	7.8	14.4	3.8	2.4	2.7	5.6	2.1	–4.0	5.9	14.4	4.1
PBE	–7.7	7.7	14.3	3.8	2.3	3.0	6.0	2.1	–3.9	5.9	14.3	4.0
PBEsol	–12.5	12.5	21.7	5.2	–5.7	5.7	8.6	2.0	–10.0	10.0	21.7	5.4
RPBE	–3.6	4.0	8.4	3.1	8.3	8.3	14.0	5.7	0.8	5.6	14.0	4.8
revPBE	–3.9	4.3	8.9	3.3	7.7	7.7	13.4	5.6	0.4	5.6	13.4	4.6
mPBE	–6.6	6.6	12.7	3.6	4.0	4.3	8.1	3.1	–2.6	5.7	12.7	3.6
mPW	–5.6	5.6	11.2	3.4	5.6	5.6	9.9	3.9	–1.4	5.6	11.2	3.6
HTBS	–7.8	7.8	14.7	4.4	1.3	3.4	5.8	1.9	–4.4	6.1	14.7	4.2
OLYP	–0.5	4.8	6.0	1.2	9.5	9.8	16.3	6.8	3.2	6.6	16.3	4.9
OPBE	–4.5	6.2	11.1	3.9	0.3	5.1	7.9	3.5	–2.7	5.8	11.1	3.8
XLYP	–1.2	2.3	4.4	1.7	15.1	15.1	19.4	5.9	4.9	7.1	19.4	7.3
					Meta–GGAs							
M06-L	–1.6	2.3	4.1	1.3	4.5	5.5	12.3	4.8	0.7	3.5	12.3	3.5
MVS	–6.0	6.0	13.4	5.0	–1.4	4.3	6.7	1.9	–4.3	5.4	13.4	4.2
TPSS	–4.4	4.4	7.3	1.9	6.0	6.0	9.9	4.3	–0.5	5.0	9.9	3.1
revTPSS	–4.5	4.5	5.4	1.0	3.7	4.8	6.8	2.3	–1.4	4.6	6.8	1.6
					Hybrids							
B3LYP	1.1	2.1	3.3	0.9	8.2	8.2	10.5	3.1	3.7	4.4	10.5	3.6
B3LYP*	–0.8	2.0	3.6	1.2	7.8	7.8	10.1	3.0	2.4	4.2	10.1	3.5
B1LYP	2.5	2.7	4.5	1.6	8.2	8.2	10.7	3.0	4.6	4.7	10.7	3.5
B1PW91	–1.2	2.3	4.2	1.7	–0.1	1.7	2.5	0.8	–0.8	2.1	4.2	1.4
BHandH	–4.0	4.4	7.8	2.6	–14.1	14.1	19.0	6.9	–7.8	8.1	19.0	6.6
BHandHLYP	6.5	6.5	8.1	1.4	2.2	2.2	3.1	0.8	4.9	4.9	8.1	2.4
KMLYP	1.2	1.6	3.4	1.1	–9.6	9.6	13.1	4.7	–2.9	4.6	13.1	4.9
O3LYP	–17.1	17.1	28.4	7.6	–19.6	19.6	27.7	8.6	–18.1	18.1	28.4	8.1
OPBE0	–0.9	4.2	6.2	1.6	–4.6	4.6	7.8	3.3	–2.3	4.3	7.8	2.4
PBE0	–3.2	3.2	7.2	2.6	–3.0	3.0	4.7	1.3	–3.1	3.1	7.2	2.2
mPW1PW	–1.5	2.2	4.6	1.6	–0.3	1.4	1.7	0.3	–1.1	1.9	4.6	1.3
mPW1K	1.3	1.5	4.4	1.6	–4.6	4.6	6.4	1.3	–0.9	2.6	6.4	2.1
S12H	–2.2	2.6	5.9	2.0	–2.5	2.8	4.9	1.8	–2.3	2.7	5.9	1.9
X3LYP	0.8	1.9	2.7	0.5	7.0	7.0	8.9	2.4	3.1	3.8	8.9	2.9
					Meta–Hybrids							
M06	0.0	1.7	3.8	1.2	1.9	2.6	6.6	2.8	0.7	2.0	6.6	2.0
M06-2X	–0.1	1.6	2.5	0.7	–0.8	0.8	1.4	0.5	–0.4	1.3	2.5	0.7
M06-HF	–0.7	1.5	5.2	1.8	–4.1	4.7	12.0	5.2	–2.0	2.7	12.0	3.8
TPSSH	–2.9	2.9	5.0	1.6	3.6	4.2	6.7	2.4	–0.4	3.4	6.7	2.1
					Double–Hybrids							
B2K-PLYP	–0.4	1.0	1.6	0.6	1.1	1.9	3.8	1.4	0.1	1.3	3.8	1.0
B2T-PLYP	–0.2	1.0	1.4	0.3	3.3	3.3	6.3	2.1	1.1	1.9	6.3	1.7
B2-PLYP	–0.6	1.1	1.9	0.6	4.8	4.8	8.2	2.5	1.4	2.5	8.2	2.4
LS1-TPSS	–3.2	3.2	5.6	1.8	–3.9	3.9	8.5	3.4	–3.5	3.5	8.5	2.5
mPW2K-PLYP	–0.6	1.2	2.0	0.6	1.0	1.9	3.5	1.2	0.0	1.4	3.5	0.9
mPW2-PLYP	0.1	0.9	1.8	0.5	3.9	3.9	6.3	1.7	1.5	2.0	6.3	1.8
PBE0-DH	–1.8	2.1	4.3	1.5	–5.7	5.7	8.9	2.2	–3.3	3.4	8.9	2.5
revDSD-BLYP	–0.6	1.3	2.8	0.9	0.9	2.0	3.4	1.0	–0.1	1.6	3.4	1.0
revDSD-PBE	–1.6	1.7	3.5	1.3	–1.4	2.6	4.7	1.5	–1.5	2.0	4.7	1.4
revDSD-PBEP86	0.0	0.8	1.3	0.4	0.5	2.1	3.9	1.4	0.2	1.3	3.9	1.1
					Range-Separated Hybrids							
CAM-B3LYP	2.4	2.4	3.6	1.3	1.6	1.6	2.5	0.6	2.1	2.1	3.6	1.1
CAMY-B3LYP	1.0	1.5	2.2	0.7	2.7	2.7	3.5	0.7	1.6	1.9	3.5	0.9
ωB97	3.4	3.4	4.0	0.3	–6.1	6.1	8.0	1.4	–0.2	4.4	8.0	1.6
ωB97X	2.5	2.5	3.5	0.7	–3.3	3.3	4.4	0.8	0.3	2.8	4.4	0.8
ωB97X-D	1.8	1.9	4.4	1.7	–0.1	1.0	1.7	0.6	1.1	1.6	4.4	1.4

Table S42 (continued).

XC	$\Delta E^{\ddagger}$				ΔE_{rxn}				Total			
	ME	MAE	MUE	SD	ME	MAE	MUE	SD	ME	MAE	MUE	SD
					Dispersion–Corrected							
BP86-D3(BJ)	–9.2	9.2	15.5	3.9	2.2	2.2	4.9	1.9	–4.9	6.6	15.5	4.7
BLYP-D3(BJ)	–5.4	5.4	9.4	2.8	10.3	10.3	13.2	3.1	0.5	7.2	13.2	3.8
PBE-D3(BJ)	–9.5	9.5	16.6	4.2	0.5	1.8	3.4	1.1	–5.8	6.6	16.6	5.0
OLYP-D3(BJ)	–8.9	8.9	13.9	3.7	0.5	2.1	4.0	1.4	–5.4	6.3	13.9	4.5
OPBE-D3(BJ)	–12.6	12.6	20.2	5.0	–8.2	8.2	13.6	4.0	–11.0	11.0	20.2	5.1
B3LYP-D3(BJ)	–2.0	2.5	4.3	1.2	5.1	5.1	6.1	0.9	0.6	3.5	6.1	1.7
PBE0-D3(BJ)	–4.9	4.9	9.2	2.8	–4.6	4.6	7.4	1.9	–4.8	4.8	9.2	2.5
M06-2X-D3	–0.1	1.6	2.4	0.7	–0.9	0.9	1.6	0.5	–0.4	1.4	2.4	0.7

Table S43. Reaction barriers and reaction energies (kcal mol^{-1}) for the various pericyclic reactions, computed with XC/QZ4P//BP86-D3(BJ)/QZ4P.

XC	Diels-Alder Reaction		1,3-dipolar Cycloaddition		Electrocyclic Rearrangement		Sigmatropic Rearrangement		Double Group Transfer	
	$\Delta E^{\ddagger}$	ΔE_{rxn}	$\Delta E^{\ddagger}$	ΔE_{rxn}	$\Delta E^{\ddagger}$	ΔE_{rxn}	$\Delta E^{\ddagger}$	ΔE_{rxn}	$\Delta E^{\ddagger}$	ΔE_{rxn}
					LDA					
VWN	0.8	–65.1	3.2	–42.3	36.3	2.5	22.1	0.0	22.1	0.0
					GGAs					
BP86	15.0	–47.2	15.4	–20.8	39.6	8.2	28.2	0.0	38.1	0.0
BLYP	21.0	–30.1	19.2	–11.0	42.7	13.8	31.8	0.0	45.2	0.0
BEE	16.2	–43.1	17.2	–20.0	38.9	6.3	28.0	0.0	38.4	0.0
PW91	12.4	–46.2	13.3	–24.1	39.3	7.4	27.8	0.0	35.8	0.0
PBE	12.8	–46.3	13.9	–23.8	38.9	6.7	27.6	0.0	38.9	0.0
PBEsol	6.7	–56.9	8.9	–33.7	36.7	3.2	24.1	0.0	28.4	0.0
RPBE	18.8	–38.1	19.2	–15.8	39.9	8.2	29.8	0.0	41.7	0.0
revPBE	18.5	–38.9	19.0	–16.4	39.7	7.8	29.3	0.0	41.2	0.0
mPBE	14.4	–44.0	15.3	–21.6	39.2	7.2	28.2	0.0	37.4	0.0
mPW	15.5	–41.9	16.1	–19.8	39.9	8.3	28.8	0.0	38.9	0.0
HTBS	14.0	–47.1	15.5	–24.1	37.4	4.6	26.1	0.0	35.5	0.0
OLYP	24.0	–36.0	23.9	–36.0	40.4	7.1	30.7	0.0	45.4	0.0
OPBE	19.4	–48.5	22.0	–22.5	36.7	0.0	26.9	0.0	39.0	0.0
XLYP	21.2	–28.9	19.3	–10.1	43.2	14.6	32.4	0.0	45.9	0.0
					Meta-GGAs					
M06-L	16.7	–46.0	19.7	–17.2	43.7	6.0	34.2	0.0	46.0	0.0
MVS	12.7	–54.3	18.4	–24.7	42.7	5.9	28.4	0.0	36.8	0.0
TPSS	15.5	–40.3	15.9	–19.8	40.2	7.7	30.9	0.0	42.9	0.0
revTPSS	14.5	–42.3	15.0	–22.9	39.6	6.2	31.5	0.0	44.8	0.0
					Hybrids					
B3LYP	22.5	–37.5	21.7	–18.9	44.8	11.8	35.0	0.0	49.6	0.0
B3LYP*	20.6	–38.3	19.8	–19.2	43.8	11.5	33.5	0.0	46.7	0.0
B1LYP	23.9	–37.3	22.9	–19.2	45.7	12.1	36.4	0.0	51.9	0.0
B1PW91	19.5	–48.5	21.1	–26.9	42.4	5.7	33.1	0.0	46.2	0.0
BHandH	12.8	–66.4	14.7	–48.2	44.3	3.6	34.3	0.0	42.6	0.0
BHandHLYP	26.6	–44.5	26.4	–27.8	48.7	10.3	41.2	0.0	58.5	0.0
KMLYP	19.0	–60.3	20.8	–42.2	46.6	5.1	38.1	0.0	50.3	0.0
O3LYP	–1.9	–75.7	1.3	–53.1	37.5	1.3	23.3	0.0	21.9	0.0
OPBE0	21.3	–54.4	24.4	–30.0	40.1	–0.3	31.7	0.0	45.3	0.0
PBE0	16.5	–52.6	18.3	–30.9	41.9	4.9	32.2	0.0	43.1	0.0
mPW1PW	18.6	–49.0	20.1	–27.7	42.7	6.3	33.3	0.0	45.7	0.0
mPW1K	16.5	–52.6	18.3	–30.9	41.9	4.9	32.2	0.0	43.1	0.0
S12H	16.3	–53.2	19.2	–29.2	42.8	4.5	34.2	0.0	44.2	0.0
X3LYP	21.7	–39.1	21.0	–20.7	44.9	11.6	35.1	0.0	49.2	0.0
					Meta-Hybrids					
M06	19.2	–47.7	22.2	–22.7	43.5	7.0	35.0	0.0	48.5	0.0
M06-2X	17.7	–48.7	21.0	–29.7	44.5	7.8	37.4	0.0	49.0	0.0
M06-HF	13.8	–48.4	18.4	–40.5	42.8	9.4	37.3	0.0	50.6	0.0
TPSSH	16.8	–43.2	17.6	–22.9	41.3	6.9	32.5	0.0	45.2	0.0
					Double-Hybrids					
B2K-PLYP	18.0	–48.6	17.4	–25.3	44.3	9.0	38.6	0.0	49.2	0.0
B2T-PLYP	19.0	–45.4	18.2	–22.8	44.3	9.9	37.4	0.0	49.3	0.0
B2-PLYP	19.0	–43.4	18.0	–20.9	43.9	10.4	36.5	0.0	48.5	0.0
LS1-TPSS	14.1	–55.8	14.7	–29.5	42.2	5.5	38.7	0.0	45.6	0.0
mPW2K-PLYP	17.7	–48.7	17.0	–25.6	44.4	9.2	38.6	0.0	49.0	0.0
mPW2-PLYP	19.4	–44.4	18.5	–22.8	44.8	10.6	37.5	0.0	49.7	0.0
PBE0-DH	16.9	–56.4	18.8	–33.7	42.9	4.2	35.1	0.0	46.1	0.0
revDSD-BLYP	17.3	–48.6	16.7	–25.3	44.6	9.6	39.2	0.0	49.6	0.0
revDSD-PBE	16.7	–51.4	16.7	–26.9	43.4	7.3	38.2	0.0	47.8	0.0
revDSD-PBEP86	18.9	–48.7	18.4	–24.7	44.2	8.2	39.2	0.0	50.0	0.0
					Range-Separated Hybrids					
CAM-B3LYP	23.3	–45.3	22.0	–27.8	46.0	9.3	37.3	0.0	51.7	0.0
CAMY-B3LYP	21.4	–44.4	20.5	–26.5	45.3	9.9	36.1	0.0	49.8	0.0
ωB97	22.9	–56.1	22.5	–35.3	46.4	3.1	40.2	0.0	53.2	0.0
ωB97X	22.4	–52.2	22.1	–32.3	45.9	5.4	38.8	0.0	51.8	0.0
ωB97X-D	22.8	–47.7	22.9	–28.0	44.6	6.3	36.4	0.0	50.3	0.0

Table S43 (continued).

XC	Diels-Alder Reaction		1,3-dipolar Cycloaddition		Electrocyclic Rearrangement		Sigmatropic Rearrangement		Double Group Transfer	
	$\Delta E^{\ddagger}$	ΔE_{rxn}	$\Delta E^{\ddagger}$	ΔE_{rxn}	$\Delta E^{\ddagger}$	ΔE_{rxn}	$\Delta E^{\ddagger}$	ΔE_{rxn}	$\Delta E^{\ddagger}$	ΔE_{rxn}
Dispersion-Corrected										
BP86-D3(BJ)	8.8	–47.2	11.2	–24.7	39.4	8.4	27.7	0.0	34.7	0.0
BLYP-D3(BJ)	13.0	–36.5	13.8	–16.2	42.3	13.9	30.9	0.0	40.8	0.0
PBE-D3(BJ)	8.9	–49.5	11.2	–26.3	38.8	6.8	27.2	0.0	33.6	0.0
OLYP-D3(BJ)	7.6	–50.3	12.1	–25.9	39.0	6.9	27.5	0.0	36.1	0.0
OPBE-D3(BJ)	4.2	–61.3	10.5	–34.2	36.5	0.9	24.2	0.0	29.9	0.0
B3LYP-D3(BJ)	16.0	–42.7	17.2	–23.1	44.5	11.9	34.4	0.0	46.0	0.0
PBE0-D3(BJ)	13.0	–55.4	15.9	–33.2	41.7	5.0	32.0	0.0	41.1	0.0
M06-2X-D3	17.6	–48.8	20.9	–29.7	44.5	7.8	36.5	0.0	48.9	0.0

Table S44. Errors in reaction barriers and reaction energies (kcal mol^{-1}) for the various pericyclic reactions, computed with XC/TZ2P//BP86-D3(BJ)/QZ4P, compared to CCSDT(Q)/CBS//CCSD(T)/cc-pVTZ.

XC	Diels-Alder Reaction		1,3-dipolar Cycloaddition		Electrocyclic Rearrangement		Sigmatropic Rearrangement		Double Group Transfer	
	$\Delta E^{‡}$	ΔE_{rxn}	$\Delta E^{‡}$	ΔE_{rxn}	$\Delta E^{‡}$	ΔE_{rxn}	$\Delta E^{‡}$	ΔE_{rxn}	$\Delta E^{‡}$	ΔE_{rxn}
LDA										
VWN	–18.6	–17.0	–15.0	–12.6	–6.7	–5.5	–14.6	0.0	–28.1	0.0
GGAs										
BP86	–4.4	6.1	–2.8	8.8	–3.5	0.4	–8.6	0.0	–12.1	0.0
BLYP	1.5	18.1	1.1	18.4	–0.4	5.9	–5.0	0.0	–5.0	0.0
BEE	–3.2	5.4	–0.9	9.8	–4.1	–1.4	–8.7	0.0	–11.7	0.0
PW91	–7.0	2.2	–4.8	5.6	–3.8	–0.4	–8.9	0.0	–14.4	0.0
PBE	–6.5	2.1	–4.2	6.0	–4.1	–1.1	–9.2	0.0	–14.3	0.0
PBEsol	–12.6	–8.6	–9.2	–3.9	–6.3	–4.7	–12.7	0.0	–21.7	0.0
RPBE	–0.6	10.4	1.1	14.0	–3.1	0.5	–7.0	0.0	–8.4	0.0
revPBE	–0.9	9.6	1.0	13.4	–3.3	0.1	–7.4	0.0	–8.9	0.0
mPBE	–5.0	4.4	–2.8	8.1	–3.8	–0.5	–8.5	0.0	–12.7	0.0
mPW	–3.9	6.5	–2.0	9.9	–3.2	0.5	–7.9	0.0	–11.2	0.0
HTBS	–5.4	1.3	–2.5	5.8	–5.5	–3.1	–10.7	0.0	–14.7	0.0
OLYP	4.7	12.6	5.9	16.3	–2.5	–0.4	–6.0	0.0	–4.6	0.0
OPBE	0.2	0.3	4.1	7.9	–6.0	–7.3	–9.7	0.0	–11.1	0.0
XLYP	1.8	19.4	1.1	19.2	0.0	6.7	–4.4	0.0	–4.3	0.0
Meta-GGAs										
M06-L	–3.1	2.8	1.2	12.3	0.5	–1.6	–2.5	0.0	–4.1	0.0
MVS	–7.5	–6.7	–0.4	4.3	–0.3	–2.0	–8.4	0.0	–13.4	0.0
TPSS	–4.0	8.2	–2.2	9.9	–2.8	0.1	–5.8	0.0	–7.3	0.0
revTPSS	–5.0	6.0	–3.2	6.8	–3.4	–1.6	–5.3	0.0	–5.4	0.0
Hybrids										
B3LYP	2.9	10.5	3.3	10.4	1.7	3.8	–1.8	0.0	–0.7	0.0
B3LYP*	1.0	9.8	1.5	10.1	0.7	3.5	–3.4	0.0	–3.6	0.0
B1LYP	4.2	10.7	4.5	10.0	2.6	4.1	–0.4	0.0	1.6	0.0
B1PW91	–0.1	–0.6	2.8	2.5	–0.6	–2.2	–3.7	0.0	–4.2	0.0
BHandH	–7.1	–19.0	–3.7	–19.0	1.1	–4.5	–2.6	0.0	–7.8	0.0
BHandHLYP	6.8	3.1	7.9	1.2	5.5	2.3	4.3	0.0	8.1	0.0
KMLYP	–0.9	–12.9	2.3	–13.1	3.4	–3.0	1.2	0.0	–0.1	0.0
O3LYP	–21.3	–27.7	–16.9	–23.6	–5.6	–7.7	–13.5	0.0	–28.4	0.0
OPBE0	1.8	–6.1	6.2	0.0	–2.7	–7.8	–5.1	0.0	–4.9	0.0
PBE0	–3.1	–4.7	0.0	–1.5	–1.1	–3.0	–4.6	0.0	–7.2	0.0
mPW1PW	–1.0	–1.0	1.8	1.7	–0.3	–1.7	–3.5	0.0	–4.6	0.0
mPW1K	1.0	–6.4	4.4	–4.3	1.6	–3.2	–0.3	0.0	0.0	0.0
S12H	–3.3	–4.9	0.9	0.5	–0.2	–3.1	–2.6	0.0	–5.9	0.0
X3LYP	2.1	8.9	2.7	8.6	1.8	3.6	–1.7	0.0	–1.1	0.0
Meta-Hybrids										
M06	–0.5	0.1	3.8	6.6	0.4	–1.1	–1.8	0.0	–1.8	0.0
M06-2X	–2.1	–1.4	2.5	–0.9	1.3	–0.3	–0.5	0.0	–1.5	0.0
M06-HF	–5.2	–1.3	0.8	–12.0	–0.4	0.9	0.5	0.0	0.6	0.0
TPSSH	–2.7	5.1	–0.6	6.7	–1.7	–0.9	–4.3	0.0	–5.0	0.0
Double-Hybrids										
B2K-PLYP	–1.6	–1.2	0.0	3.8	1.4	0.7	–0.8	0.0	–1.2	0.0
B2T-PLYP	–0.7	2.1	0.5	6.3	1.4	1.6	–1.2	0.0	–1.2	0.0
B2-PLYP	–0.6	4.2	0.3	8.2	1.0	2.2	–1.8	0.0	–1.9	0.0
LS1-TPSS	–5.6	–8.5	–2.5	–0.3	–0.5	–2.9	–2.6	0.0	–4.8	0.0
mPW2K-PLYP	–2.0	–1.4	–0.4	3.5	1.5	0.8	–0.7	0.0	–1.4	0.0
mPW2-PLYP	–0.3	3.1	0.6	6.3	1.8	2.3	–0.8	0.0	–0.8	0.0
PBE0-DH	–2.8	–8.9	0.7	–4.4	–0.1	–3.9	–2.5	0.0	–4.3	0.0
revDSD-BLYP	–2.8	–1.8	–1.0	3.4	1.6	1.0	0.1	0.0	–1.0	0.0
revDSD-PBE	–3.5	–4.7	–1.1	1.8	0.4	–1.3	–0.8	0.0	–3.0	0.0
revDSD-PBEP86	–1.3	–2.0	0.6	3.9	1.2	–0.4	0.2	0.0	–0.8	0.0
Range-Separated Hybrids										
CAM-B3LYP	3.7	2.4	3.7	1.1	2.6	0.9	0.5	0.0	1.5	0.0
CAMY-B3LYP	1.8	3.3	2.2	2.4	1.9	1.5	–0.7	0.0	–0.4	0.0
ωB97	3.3	–8.5	4.2	–6.4	3.0	–5.4	3.4	0.0	3.0	0.0
ωB97X	2.8	–4.6	3.8	–3.4	2.5	–3.1	2.0	0.0	1.6	0.0
ωB97X-D	3.2	–0.1	4.6	0.9	1.2	–2.2	–0.4	0.0	0.1	0.0

Table S44 (continued).

XC	Diels-Alder Reaction		1,3-dipolar Cycloaddition		Electrocyclic Rearrangement		Sigmatropic Rearrangement		Double Group Transfer	
	$\Delta E^{\ddagger}$	ΔE_{rxn}	$\Delta E^{\ddagger}$	ΔE_{rxn}	$\Delta E^{\ddagger}$	ΔE_{rxn}	$\Delta E^{\ddagger}$	ΔE_{rxn}	$\Delta E^{\ddagger}$	ΔE_{rxn}
					Dispersion-Corrected					
BP86-D3(BJ)	–10.6	1.2	–7.0	4.9	–3.7	0.6	–9.1	0.0	–15.5	0.0
BLYP-D3(BJ)	–6.4	11.8	–4.4	13.2	–0.9	6.0	–5.8	0.0	–9.4	0.0
PBE-D3(BJ)	–10.5	–1.1	–6.9	3.4	–4.2	–0.9	–9.5	0.0	–16.6	0.0
OLYP-D3(BJ)	–11.7	–1.8	–5.9	4.0	–3.8	–0.6	–9.1	0.0	–13.9	0.0
OPBE-D3(BJ)	–16.0	–13.6	–7.4	–3.8	–7.2	–7.4	–12.4	0.0	–20.2	0.0
B3LYP-D3(BJ)	–3.6	5.3	–1.1	6.1	1.4	3.9	–2.4	0.0	–4.3	0.0
PBE0-D3(BJ)	–6.6	–7.4	–2.4	–3.7	–1.3	–2.9	–4.9	0.0	–9.2	0.0
M06-2X-D3	–2.3	–1.6	2.4	–1.0	1.3	–0.3	–0.5	0.0	–1.6	0.0

Table S45. Mean error (ME), mean absolute error (MAE), maximum unsigned error (MUE) and standard deviation (SD) of reaction barriers and energies (kcal mol^{-1}) for the various pericyclic reactions, computed with XC/QZ4P//BP86-D3(BJ)/QZ4P compared to CCSDT(Q)/CBS//CCSD(T)/cc-pVTZ.

XC	$\Delta E^{\ddagger}$				ΔE_{rxn}				Total			
	ME	MAE	MUE	SD	ME	MAE	MUE	SD	ME	MAE	MUE	SD
					LDA							
VWN	–16.8	16.8	28.1	6.8	–12.3	12.3	17.5	4.8	–15.1	15.1	28.1	6.5
					GGAs							
BP86	–6.4	6.4	12.1	3.5	2.8	2.9	8.1	3.6	–3.0	5.1	12.1	3.9
BLYP	–1.7	2.6	5.0	2.0	13.6	13.6	17.9	5.8	4.0	6.7	17.9	6.6
BEE	–5.9	5.9	11.8	3.9	3.8	5.2	8.9	2.8	–2.3	5.6	11.8	3.5
PW91	–7.9	7.9	14.4	3.7	1.7	2.4	4.8	1.7	–4.3	5.9	14.4	4.1
PBE	–7.2	7.2	11.3	2.7	1.6	2.7	5.1	1.7	–3.9	5.5	11.3	3.2
PBEsol	–12.7	12.7	21.8	5.1	–6.4	6.4	9.3	2.0	–10.4	10.4	21.8	5.2
RPBE	–3.8	4.1	8.5	3.1	7.5	7.6	13.1	5.4	0.4	5.4	13.1	4.5
revPBE	–4.1	4.4	9.0	3.4	6.9	7.3	12.5	4.9	0.0	5.5	12.5	4.3
mPBE	–6.8	6.8	12.8	3.6	3.2	4.1	7.3	2.5	–3.0	5.7	12.8	3.5
mPW	–5.8	5.8	11.3	3.4	4.9	5.0	9.1	3.7	–1.8	5.5	11.3	3.5
HTBS	–8.0	8.0	14.7	4.2	0.5	3.1	4.8	1.8	–4.8	6.1	14.7	4.2
OLYP	–0.8	4.8	6.1	1.1	1.1	6.7	11.7	4.2	–0.1	5.5	11.7	2.9
OPBE	–4.9	6.3	11.2	4.0	–1.0	5.2	8.5	3.2	–3.4	5.9	11.2	3.8
XLYP	–1.3	2.3	4.4	1.7	14.6	14.6	18.8	5.9	4.7	6.9	18.8	7.1
					Meta–GGAs							
M06-L	–1.6	2.3	4.2	1.3	3.6	5.3	11.7	4.5	0.4	3.4	11.7	3.3
MVS	–5.9	5.9	13.4	5.0	–1.7	4.5	6.7	1.7	–4.3	5.4	13.4	4.1
TPSS	–4.6	4.6	7.3	1.8	5.2	5.7	9.1	3.6	–0.9	5.0	9.1	2.7
revTPSS	–4.6	4.6	5.4	0.9	3.0	4.5	6.0	1.6	–1.7	4.6	6.0	1.2
					Hybrids							
B3LYP	1.1	2.0	3.4	1.0	7.8	7.8	10.2	3.2	3.6	4.2	10.2	3.5
B3LYP*	–0.8	1.9	3.5	1.3	7.4	7.4	9.7	3.0	2.3	4.0	9.7	3.4
B1LYP	2.5	2.7	4.6	1.6	7.9	7.9	10.4	3.0	4.5	4.6	10.4	3.4
B1PW91	–1.2	2.3	4.0	1.5	–0.5	1.9	2.8	0.8	–1.0	2.1	4.0	1.3
BHandH	–3.9	4.3	7.6	2.6	–14.3	14.3	19.3	6.7	–7.8	8.0	19.3	6.7
BHandHLYP	6.6	6.6	8.3	1.5	2.0	2.0	3.2	0.9	4.9	4.9	8.3	2.6
KMLYP	1.3	1.5	3.2	1.2	–9.8	9.8	13.3	4.6	–2.9	4.6	13.3	5.0
O3LYP	–17.2	17.2	28.3	7.5	–19.8	19.8	28.1	9.1	–18.2	18.2	28.3	8.2
OPBE0	–1.1	4.2	6.1	1.5	–5.5	5.5	8.8	3.2	–2.8	4.7	8.8	2.4
PBE0	–3.3	3.3	7.1	2.5	–3.5	3.5	5.0	1.2	–3.4	3.4	7.1	2.1
mPW1PW	–1.6	2.3	4.5	1.5	–0.8	1.6	2.2	0.4	–1.3	2.0	4.5	1.2
mPW1K	–3.3	3.3	7.1	2.5	–3.5	3.5	5.0	1.2	–3.4	3.4	7.1	2.1
S12H	–2.3	2.7	6.0	1.9	–3.3	3.3	5.6	2.2	–2.7	2.9	6.0	2.1
X3LYP	0.7	1.8	2.7	0.6	6.6	6.6	8.6	2.5	2.9	3.6	8.6	2.8
					Meta–Hybrids							
M06	0.0	1.6	3.9	1.4	1.6	2.6	6.2	2.6	0.6	1.9	6.2	2.0
M06-2X	0.3	1.5	2.7	0.7	–0.8	0.8	1.1	0.2	–0.2	1.3	2.7	0.7
M06-HF	–1.1	1.5	5.8	2.1	–3.8	4.4	11.6	5.1	–2.1	2.6	11.6	3.8
TPSSH	–3.0	3.0	5.0	1.5	3.0	4.0	6.0	1.8	–0.8	3.4	6.0	1.7
					Double–Hybrids							
B2K-PLYP	–0.2	1.2	1.8	0.4	1.1	1.7	3.6	1.3	0.3	1.4	3.6	0.9
B2T-PLYP	0.0	0.6	0.9	0.3	3.3	3.3	6.1	2.0	1.2	1.6	6.1	1.8
B2-PLYP	–0.5	0.7	1.7	0.5	4.7	4.7	8.0	2.5	1.5	2.2	8.0	2.5
LS1-TPSS	–2.6	3.4	5.5	1.6	–3.9	3.9	8.2	3.1	–3.1	3.6	8.2	2.3
mPW2K-PLYP	–0.3	1.4	1.9	0.3	1.0	1.7	3.3	1.1	0.2	1.5	3.3	0.7
mPW2-PLYP	0.3	0.6	1.4	0.4	3.8	3.8	6.1	1.6	1.6	1.8	6.1	1.9
PBE0-DH	–1.7	1.9	4.1	1.4	–5.9	5.9	8.8	2.0	–3.3	3.4	8.8	2.5
revDSD-BLYP	–0.2	1.6	2.4	0.7	1.3	1.9	3.6	1.2	0.4	1.7	3.6	0.9
revDSD-PBE	–1.1	1.7	2.9	1.0	–1.0	2.3	3.8	1.1	–1.1	1.9	3.8	1.1
revDSD-PBEP86	0.5	0.8	2.4	0.8	1.0	1.8	4.2	1.7	0.7	1.2	4.2	1.3
					Range-Separated Hybrids							
CAM-B3LYP	2.4	2.4	3.7	1.3	1.4	1.4	2.4	0.7	2.0	2.0	3.7	1.2
CAMY-B3LYP	1.0	1.4	2.2	0.7	2.4	2.4	3.3	0.7	1.5	1.8	3.3	0.9
ωB97	3.4	3.4	4.2	0.5	–6.7	6.7	8.5	1.3	–0.4	4.6	8.5	1.8
ωB97X	2.5	2.5	3.8	0.8	–3.7	3.7	4.6	0.6	0.2	3.0	4.6	0.9
ωB97X-D	1.7	1.9	4.6	1.8	–0.4	1.0	2.2	0.9	0.9	1.6	4.6	1.5

Table S45 (continued).

XC	$\Delta E^{\ddagger}$				ΔE_{rxn}				Total			
	ME	MAE	MUE	SD	ME	MAE	MUE	SD	ME	MAE	MUE	SD
					Dispersion–Corrected							
BP86-D3(BJ)	–9.3	9.3	15.5	3.8	1.5	1.6	4.2	1.9	–5.2	6.4	15.5	5.0
BLYP-D3(BJ)	–5.5	5.5	9.4	2.7	9.8	9.8	12.7	3.1	0.2	7.1	12.7	3.5
PBE-D3(BJ)	–9.7	9.7	16.6	4.0	–0.3	2.0	2.6	0.4	–6.2	6.8	16.6	4.9
OLYP-D3(BJ)	–9.2	9.2	14.1	3.6	–0.4	2.4	3.0	0.6	–5.9	6.6	14.1	4.4
OPBE-D3(BJ)	–12.6	12.6	20.3	5.0	–8.8	8.8	13.7	3.5	–11.2	11.2	20.3	4.8
B3LYP-D3(BJ)	–2.0	2.5	4.2	1.3	4.7	4.7	5.8	1.0	0.5	3.3	5.8	1.6
PBE0-D3(BJ)	–4.9	4.9	9.1	2.7	–5.2	5.2	7.8	1.9	–5.0	5.0	9.1	2.4
M06-2X-D3	0.0	1.5	2.6	0.8	–0.9	0.9	1.2	0.2	–0.3	1.2	2.6	0.7

Table S46. Multireference diagnostics for each of the pericyclic stationary points at the CCSD(T)/cc-pVTZ level of theory

Species	T_1 Diagnostic	Largest T_2 Amplitude
**B**	0.011	–0.090
**E**	0.011	–0.120
**MA**	0.018	–0.073
**P**	0.011	–0.083
**Et**	0.008	0.000
**TS-DA**	0.010	–0.078
**P-DA**	0.010	–0.051
**TS-1,3-DC**	0.016	–0.049
**P-1,3-DC**	0.014	–0.030
**TS-SR**	0.011	–0.093
**TS-ER**	0.011	–0.113
**P-ER**	0.010	–0.107
**TS-DGT**	0.009	–0.089

Table S47. Standard Dunning basis raw electronic energies atop the CCSD(T)/cc-pVTZ structures for use in Focal Point analysis

B

	RHF	MP2	CCSD	CCSD(T)	CCSDT	CCSDT(Q)
cc–pVDZ	−154.9288987	−155.4643094	−155.5109513	−155.5326566	−155.5336371	−155.5351984
cc–pVTZ	−154.9714046	−155.6231795	−155.6589972	−155.6913388		
cc–pVQZ	−154.9813853	−155.6734250	−155.7010477	−155.7360870		
cc–pV5Z	−154.9838188	−155.6910929				

E(FC–CCSD(T)/cc–pCVTZ) = −155.6960772
E(AE–CCSD(T)/cc–pCVTZ) = −155.8950888

***trans*–B**

	RHF	MP2	CCSD	CCSD(T)	CCSDT	CCSDT(Q)
cc–pVDZ	−154.9341826	−155.4689579	−155.5154742	−155.5373092	−155.5383244	−155.5398930
cc–pVTZ	−154.9769368	−155.6278093	−155.6635330	−155.6959336		
cc–pVQZ	−154.9869492	−155.6780758	−155.7056171	−155.7407116		
cc–pV5Z	−154.9893903	−155.6957483				

E

	RHF	MP2	CCSD	CCSD(T)	CCSDT	CCSDT(Q)
cc–pVDZ	–78.0398309	–78.3148492	–78.3450186	–78.3548453	–78.3554769	–78.3561073
cc–pVTZ	–78.0635295	–78.3992523	–78.4237557	–78.4388035		
cc–pVQZ	–78.0688081	–78.4251777	–78.4453630	–78.4617267		
cc–pV5Z	–78.0701501	–78.4342943				

E(FC–CCSD(T)/cc–pCVTZ) = –78.44112367
E(AE–CCSD(T)/cc–pCVTZ) = –78.54059175

MA

	RHF	MP2	CCSD	CCSD(T)	CCSDT	CCSDT(Q)
cc–pVDZ	–202.8830963	–203.5330477	–203.5409648	–203.5703363	–203.5702344	–203.5740625
cc–pVTZ	–202.9398678	–203.7289180	–203.7256379	–203.7684651		
cc–pVQZ	–202.9540497	–203.7937466	–203.7821439	–203.8285177		
cc–pV5Z	–202.9573898	–203.8170967				

E(FC–CCSD(T)/cc–pCVTZ) = –203.7769782
E(AE–CCSD(T)/cc–pCVTZ) = –203.9820051

P

	RHF	MP2	CCSD	CCSD(T)	CCSDT	CCSDT(Q)
cc–pVDZ	−193.9675307	−194.6476637	−194.7059797	−194.7328522	−194.7339521	−194.7358514
cc–pVTZ	−194.0201477	−194.8483264	−194.8930656	−194.9332918		
cc–pVQZ	−194.0325070	−194.9115957	−194.9460874	−194.9896902		
cc–pV5Z	−194.0355025	−194.9337951				

E(FC–CCSD(T)/cc–pCVTZ) = –194.9391704
E(AE–CCSD(T)/cc–pCVTZ) = –195.1880501

***trans*–P**

	RHF	MP2	CCSD	CCSD(T)	CCSDT	CCSDT(Q)
cc–pVDZ	−193.9732386	−194.6520861	−194.7104398	−194.7373793	−194.7385396	−194.7404453
cc–pVTZ	−194.0261060	−194.8527622	−194.8975974	−194.9378227		
cc–pVQZ	−194.0385274	−194.9160736	−194.9506829	−194.9942793		
cc–pV5Z	−194.0415550	−194.9382951				

E

	RHF	MP2	CCSD	CCSD(T)	CCSDT	CCSDT(Q)
cc–pVDZ	–79.2349329	–79.5370245	–79.5740384	–79.5823175	–79.5829471	–79.5833399
cc–pVTZ	–79.2597874	–79.6298460	–79.6609679	–79.6744450		
cc–pVQZ	–79.2650239	–79.6573473	–79.6839574	–79.6987107		
cc–pV5Z	–79.2663607	–79.6669257				

E(FC–CCSD(T)/cc–pCVTZ) = –79.6765236
E(AE–CCSD(T)/cc–pCVTZ) = –79.77616799

TS–DA

	RHF	MP2	CCSD	CCSD(T)	CCSDT	CCSDT(Q)
cc–pVDZ	–232.8992400	–233.7594060	–233.8145528	–233.8538137	–233.8546886	–233.8577275
cc–pVTZ	–232.9632774	–234.0059838	–234.0423793	–234.0987534		
cc–pVQZ	–232.9779710	–234.0825452	–234.1060104	–234.1667246		
cc–pV5Z	–232.9815375	–234.1094328				

E(FC–CCSD(T)/cc–pCVTZ) = –234.1056506
E(AE–CCSD(T)/cc–pCVTZ) = –234.4039487

P–DA

	RHF	MP2	CCSD	CCSD(T)	CCSDT	CCSDT(Q)
cc–pVDZ	–233.0367972	–233.8662585	–233.9346806	–233.9655017	–233.9665604	–233.9685235
cc–pVTZ	–233.0975504	–234.1064229	–234.1591461	–234.2058720		
cc–pVQZ	–233.1120132	–234.1825879	–234.2232204	–234.2739459		
cc–pV5Z	–233.1154795	–234.2093095				

E(FC–CCSD(T)/cc–pCVTZ) = –234.2126671
E(AE–CCSD(T)/cc–pCVTZ) = –234.5114888

TS–1,3–DC

	RHF	MP2	CCSD	CCSD(T)	CCSDT	CCSDT(Q)
cc–pVDZ	–280.8602834	–281.8294441	–281.8500866	–281.8966775	–281.8960871	–281.9018519
cc–pVTZ	–280.9348921	–282.1101293	–282.1108629	–282.1775562		
cc–pVQZ	–280.9536608	–282.2014120	–282.1889078	–282.2608781		
cc–pV5Z	–280.9580912	–282.2342253				

E(FC–CCSD(T)/cc–pCVTZ) = –282.1879762
E(AE–CCSD(T)/cc–pCVTZ) = –282.4920985

P–1,3–DC

	RHF	MP2	CCSD	CCSD(T)	CCSDT	CCSDT(Q)
cc–pVDZ	–280.9712756	–281.8865515	–281.9340761	–281.9703206	–281.9706691	–281.9740902
cc–pVTZ	–281.0462135	–282.1665370	–282.1966583	–282.2519795		
cc–pVQZ	–281.0654252	–282.2585138	–282.2760870	–282.3363554		
cc–pV5Z	–281.0700200	–282.2916434				

E(FC–CCSD(T)/cc–pCVTZ) = –282.2620123
E(AE–CCSD(T)/cc–pCVTZ) = –282.5664449

TS–ER

	RHF	MP2	CCSD	CCSD(T)	CCSDT	CCSDT(Q)
cc–pVDZ	–154.8389905	–155.3986728	–155.4374530	–155.4633722	–155.4640989	–155.4661160
cc–pVTZ	–154.8811466	–155.5584944	–155.5851977	–155.6222873		
cc–pVQZ	–154.8910071	–155.6088533	–155.6270821	–155.6669968		
cc–pV5Z	–154.8934213	–155.6266140				

E(FC–CCSD(T)/cc–pCVTZ) = –155.6269212
E(AE–CCSD(T)/cc–pCVTZ) = –155.8256164

P–ER

	RHF	MP2	CCSD	CCSD(T)	CCSDT	CCSDT(Q)
cc–pVDZ	–154.9117217	–155.4552121	–155.4984332	–155.5196265	–155.5203062	–155.5218180
cc–pVTZ	–154.9526202	–155.6130015	–155.6456912	–155.6776100		
cc–pVQZ	–154.9623458	–155.6633752	–155.6880868	–155.7226899		
cc–pV5Z	–154.9646733	–155.6811429				

E(FC–CCSD(T)/cc–pCVTZ) = –155.6822329
E(AE–CCSD(T)/cc–pCVTZ) = –155.8812007

TS–SR

	RHF	MP2	CCSD	CCSD(T)	CCSDT	CCSDT(Q)
cc–pVDZ	–193.8816627	–194.5983275	–194.6415675	–194.6741341	–194.6748017	–194.6773575
cc–pVTZ	–193.9325727	–194.7990793	–194.8275651	–194.8743033		
cc–pVQZ	–193.9448486	–194.8626389	–194.8806682	–194.9309717		
cc–pV5Z	–193.9478418	–194.8848894				

E(FC–CCSD(T)/cc–pCVTZ) = –194.8801141
E(AE–CCSD(T)/cc–pCVTZ) = –195.1288721

TS–DGT

	RHF	MP2	CCSD	CCSD(T)	CCSDT	CCSDT(Q)
cc–pVDZ	–157.1471812	–157.7830069	–157.8302236	–157.8558066	–157.8565883	–157.8585055
cc–pVTZ	–157.1926246	–157.9612959	–157.9948921	–158.0325438		
cc–pVQZ	–157.2026964	–158.0151958	–158.0395703	–158.0802108		
cc–pV5Z	–157.2052153	–158.0337998				

E(FC–CCSD(T)/cc–pCVTZ) = –158.036793
E(AE–CCSD(T)/cc–pCVTZ) = –158.235902

Table S48. Aug′ basis raw electronic energies atop the CCSD(T)/cc-pVTZ structures for use in Focal Point analysis

B

	RHF	MP2	CCSD	CCSD(T)
aug′–cc–pVDZ	–154.9332966	–155.4848185	–155.5297139	–155.5538307
aug′–cc–pVTZ	–154.9726010	–155.6315664	–155.6661443	–155.6994119
aug′–cc–pVQZ	–154.9818063	–155.6773817	–155.7038498	–155.7392358
aug′–cc–pV5Z	–154.9838710	–155.6929894		

***trans*–B**

	RHF	MP2	CCSD	CCSD(T)
aug′–cc–pVDZ	–154.9388329	–155.4896435	–155.5343688	–155.5585730
aug′–cc–pVTZ	–154.9781644	–155.6361878	–155.6706652	–155.7039922
aug′–cc–pVQZ	–154.9873795	–155.6820473	–155.7084341	–155.7438785
aug′–cc–pV5Z	–154.9894482	–155.6976627		

E

	RHF	MP2	CCSD	CCSD(T)
aug′–cc–pVDZ	–78.0425010	–78.3253851	–78.3548073	–78.3657735
aug′–cc–pVTZ	–78.0642344	–78.4031459	–78.4270781	–78.4425588
aug′–cc–pVQZ	–78.0690579	–78.4270457	–78.4466747	–78.4631981
aug′–cc–pV5Z	–78.0701799	–78.4351828		

MA

	RHF	MP2	CCSD	CCSD(T)
aug′–cc–pVDZ	–202.8953823	–203.5679612	–203.5746089	–203.6073716
aug′–cc–pVTZ	–202.9424350	–203.7434969	–203.7387201	–203.7830631
aug′–cc–pVQZ	–202.9548516	–203.8003674	–203.7872330	–203.8341865
aug′–cc–pV5Z	–202.9575278	–203.8202676		

P

	RHF	MP2	CCSD	CCSD(T)
aug′–cc–pVDZ	−193.9725624	−194.6735229	−194.7298726	−194.7597830
aug′–cc–pVTZ	−194.0214524	−194.8588343	−194.9021762	−194.9435512
aug′–cc–pVQZ	−194.0329635	−194.9164543	−194.9495737	−194.9936064
aug′–cc–pV5Z	−194.0355593	−194.9361400		

***trans*–P**

	RHF	MP2	CCSD	CCSD(T)
aug′–cc–pVDZ	–193.9784928	–194.6781347	–194.7344836	–194.7644244
aug′–cc–pVTZ	–194.0275052	–194.8633009	–194.9067348	–194.9481079
aug′–cc–pVQZ	–194.0390147	–194.9209658	–194.9542004	–194.9982289
aug′–cc–pV5Z	–194.0416194	–194.9406599		

Et

	RHF	MP2	CCSD	CCSD(T)
aug′–cc–pVDZ	–79.2362457	–79.5470023	–79.5840179	–79.5933922
aug′–cc–pVTZ	–79.2600623	–79.6333060	–79.6642428	–79.6780935
aug′–cc–pVQZ	–79.2651126	–79.6588848	–79.6851146	–79.7000040
aug′–cc–pV5Z	–79.2663736	–79.6676808		

TS–DA

	RHF	MP2	CCSD	CCSD(T)
aug′–cc–pVDZ	–232.9066197	–233.7960667	–233.8474308	–233.8910377
aug′–cc–pVTZ	–232.9649694	–234.0200174	–234.0544235	–234.1123808
aug′–cc–pVQZ	–232.9785247	–234.0889540	–234.1106322	–234.1719443
aug′–cc–pV5Z	–232.9816192	–234.1124592		

P–DA

	RHF	MP2	CCSD	CCSD(T)
aug′–cc–pVDZ	–233.0430730	–233.8960669	–233.9629866	–233.9972720
aug′–cc–pVTZ	–233.0986939	–234.1187808	–234.1702163	–234.2182312
aug′–cc–pVQZ	–233.1124370	–234.1882681	–234.2274236	–234.2786402
aug′–cc–pV5Z	–233.1155323	–234.2121270		

TS–1,3DC

	RHF	MP2	CCSD	CCSD(T)
aug′–cc–pVDZ	–280.8731801	–281.8809755	–281.8974282	–281.9499069
aug′–cc–pVTZ	–280.9377924	–282.1308842	–282.1291319	–282.1981652
aug′–cc–pVQZ	–280.9545970	–282.2108402	–282.1960750	–282.2689429
aug′–cc–pV5Z	–280.9582268	–282.2386580		

P–1,3DC

	RHF	MP2	CCSD	CCSD(T)
aug′–cc–pVDZ	–280.9874944	–281.9373642	–281.9825142	–282.0240380
aug′–cc–pVTZ	–281.0496008	–282.1869000	–282.2150866	–282.2724401
aug′–cc–pVQZ	–281.0665382	–282.2676834	–282.2832425	–282.3443081
aug′–cc–pV5Z	–281.0701717	–282.2959322		

TS–ER

	RHF	MP2	CCSD	CCSD(T)
aug′–cc–pVDZ	–154.8442958	–155.4206842	–155.4573071	–155.4858028
aug′–cc–pVTZ	–154.8824792	–155.5674822	–155.5928330	–155.6308903
aug′–cc–pVQZ	–154.8914544	–155.6130433	–155.6300545	–155.6703426
aug′–cc–pV5Z	–154.8934775	–155.6286084		

P–ER

	RHF	MP2	CCSD	CCSD(T)
aug′–cc–pVDZ	–154.9159091	–155.4737916	–155.5161264	–155.5396371
aug′–cc–pVTZ	–154.9535162	–155.6210696	–155.6528768	–155.6856475
aug′–cc–pVQZ	–154.9626740	–155.6672442	–155.6909212	–155.7258575
aug′–cc–pV5Z	–154.9647154	–155.6830324		

TS–SR

	RHF	MP2	CCSD	CCSD(T)
aug′–cc–pVDZ	–193.8869737	–194.6249764	–194.6657623	–194.7015552
aug′–cc–pVTZ	–193.9339631	–194.8098697	–194.8368913	–194.8848240
aug′–cc–pVQZ	–193.9453517	–194.8675958	–194.8842420	–194.9349899
aug′–cc–pV5Z	–193.9479093	–194.8872785		

TS–DGT

	RHF	MP2	CCSD	CCSD(T)
aug′–cc–pVDZ	–157.1511377	–157.8061977	–157.8518762	–157.8803342
aug′–cc–pVTZ	–157.1933888	–157.9694211	–158.0021776	–158.0408038
aug′–cc–pVQZ	–157.2029645	–158.0187245	–158.0421527	–158.0831279
aug′–cc–pV5Z	–157.2052578	–158.0355264		
